# Comparative RNA-Seq transcriptome analyses reveal dynamic time-dependent effects of ^56^Fe, ^16^O, and ^28^Si irradiation on the induction of murine hepatocellular carcinoma

**DOI:** 10.1186/s12864-020-06869-4

**Published:** 2020-07-01

**Authors:** Anna M. Nia, Kamil Khanipov, Brooke L. Barnette, Robert L. Ullrich, George Golovko, Mark R. Emmett

**Affiliations:** 1 Biochemistry and Molecular Biology, University of Texas Medical Branch, 301 University Blvd, Galveston, TX 77550 USA; 2 Pharmacology and Toxicology, University of Texas Medical Branch, 301 University Blvd, Galveston, TX 77550 USA; 3 The Radiation Effects Research Foundation (RERF), Hiroshima, Japan

**Keywords:** RNA-Sequencing, Self-organizing maps, Novel transcripts, Carcinogenesis, Tumor microenvironment

## Abstract

**Background:**

One of the health risks posed to astronauts during deep space flights is exposure to high charge, high-energy (HZE) ions (Z > 13), which can lead to the induction of hepatocellular carcinoma (HCC). However, little is known on the molecular mechanisms of HZE irradiation-induced HCC.

**Results:**

We performed comparative RNA-Seq transcriptomic analyses to assess the carcinogenic effects of 600 MeV/n ^56^Fe (0.2 Gy), 1 GeV/n ^16^O (0.2 Gy), and 350 MeV/n ^28^Si (0.2 Gy) ions in a mouse model for irradiation-induced HCC. C3H/HeNCrl mice were subjected to total body irradiation to simulate space environment HZE-irradiation, and liver tissues were extracted at five different time points post-irradiation to investigate the time-dependent carcinogenic response at the transcriptomic level. Our data demonstrated a clear difference in the biological effects of these HZE ions, particularly immunological, such as Acute Phase Response Signaling, B Cell Receptor Signaling, IL-8 Signaling, and ROS Production in Macrophages. Also seen in this study were novel unannotated transcripts that were significantly affected by HZE. To investigate the biological functions of these novel transcripts, we used a machine learning technique known as self-organizing maps (SOMs) to characterize the transcriptome expression profiles of 60 samples (45 HZE-irradiated, 15 non-irradiated control) from liver tissues. A handful of localized modules in the maps emerged as groups of co-regulated and co-expressed transcripts. The functional context of these modules was discovered using overrepresentation analysis. We found that these spots typically contained enriched populations of transcripts related to specific immunological molecular processes (e.g., Acute Phase Response Signaling, B Cell Receptor Signaling, IL-3 Signaling), and RNA Transcription/Expression.

**Conclusions:**

A large number of transcripts were found differentially expressed post-HZE irradiation. These results provide valuable information for uncovering the differences in molecular mechanisms underlying HZE specific induced HCC carcinogenesis. Additionally, a handful of novel differentially expressed unannotated transcripts were discovered for each HZE ion. Taken together, these findings may provide a better understanding of biological mechanisms underlying risks for HCC after HZE irradiation and may also have important implications for the discovery of potential countermeasures against and identification of biomarkers for HZE-induced HCC.

## Background

An important goal for the National Aeronautics and Space Administration (NASA) is to identify the effects of spaceflight-like conditions on irradiation-induced cancer. However, understanding the mechanisms of irradiation-induced cancer is impeded by the fact that there are no quantitative data from human populations exposed to the specific types of irradiation encountered during missions beyond low-earth orbit (LEO) or in deep space. During these missions, astronauts will be continuously exposed to low dose ionizing irradiation (LDR). In particular, high charge, high-energy (HZE) ions such as ^56^Fe, ^16^O, and ^28^Si are the major high linear energy transfer (LET) sources in deep space [1–3]. Previous studies have indicated that irradiation of mice with low dose HZE, specifically ^56^Fe ions, significantly increases the incidences of HCC, but there is a limited understanding of potential mechanisms [4]. Previous studies by multiple investigators have shown that irradiation of mice with HZE particles induces oxidative damage, and micro-environmental changes that are thought to play a role in the carcinogenic processes, yet a detailed analysis of these processes has not been undertaken [2, 4–11]. The main goal of these studies was to establish an association between HZE irradiation and a specific response such as oxidative stress, microenvironmental changes, and/or apoptosis.

The pathogenic process involved in the development of HCC and other cancers following irradiation exposure likely begins with the induction of mutagenic, and/or epigenetic changes and production of oncometabolites that further results in transcriptional alterations leading to a premalignant state. Irradiation can activate and/or inhibit a myriad of transcriptional pathways that are mainly involved in inflammation and oxidative changes that may play a role in the subsequent development of irradiation-related cancers, which involves chronic oxidative stress leading to irradiation-induced tissue injury, and the subsequent development of HCC [7, 11, 12]. The use of RNA-Seq, an approach to transcriptome profiling, which utilizes the deep-sequencing technologies, has become an increasingly common technique to study biological phenomena at the molecular level. This approach generates quantitative data of thousands of different messenger RNAs (mRNAs) with each experiment. To better understand the molecular mechanisms of HZE induced hepatic carcinogenesis, we performed RNA isolation and sequencing of the livers of male C3H/HeNCrl mice. This strain has been shown to be susceptible to the induction of low-dose HZE-induced spontaneous HCC [4]. Low dose irradiation induces micro-environmental changes that lead to carcinogenesis and potentially tumor development. We conducted transcriptomic analyses to identify altered transcript expression in response to different types of HZE irradiation. The results of the present study confirm previous observations of significant differences between ^56^Fe irradiation and non-irradiated control with respect to the induction of HCC [4, 10].

Additionally, the alignment of RNA-Seq reads to the reference set of transcripts usually highlights a small but significant fraction of novel transcripts. Such transcripts are usually unexplored due to their unmappability to the genome sequence and/or the fact that they are missing gene annotations. In recent years, there has been increased attention paid to the unannotated transcript expression data as a potentially valuable resource to identify novel transcripts missing from the existing transcriptome annotations [13–18]. The unannotated transcripts from RNA-Seq in our experiments offered us an opportunity to find novel transcripts that are significantly affected by HZE and potentially associated with irradiation-induced HCC.

To gain biological knowledge about the scope of the cellular processes involved in the irradiation-induced HCC, we analyzed quantitative transcriptional changes in the livers of C3H/HeNCrl mice after irradiation with ^56^Fe, ^16^O, and ^28^Si compared with those from non-irradiated control. These analyses helped us define key molecular components that are driving the HZE induced transcriptional changes leading to HCC as well as functional roles of unannotated transcripts.

## Results

### Differential expression analysis of ^56^Fe reveals dynamic time-dependent changes in inflammatory response at the whole transcriptome level

**Fig. 1:**
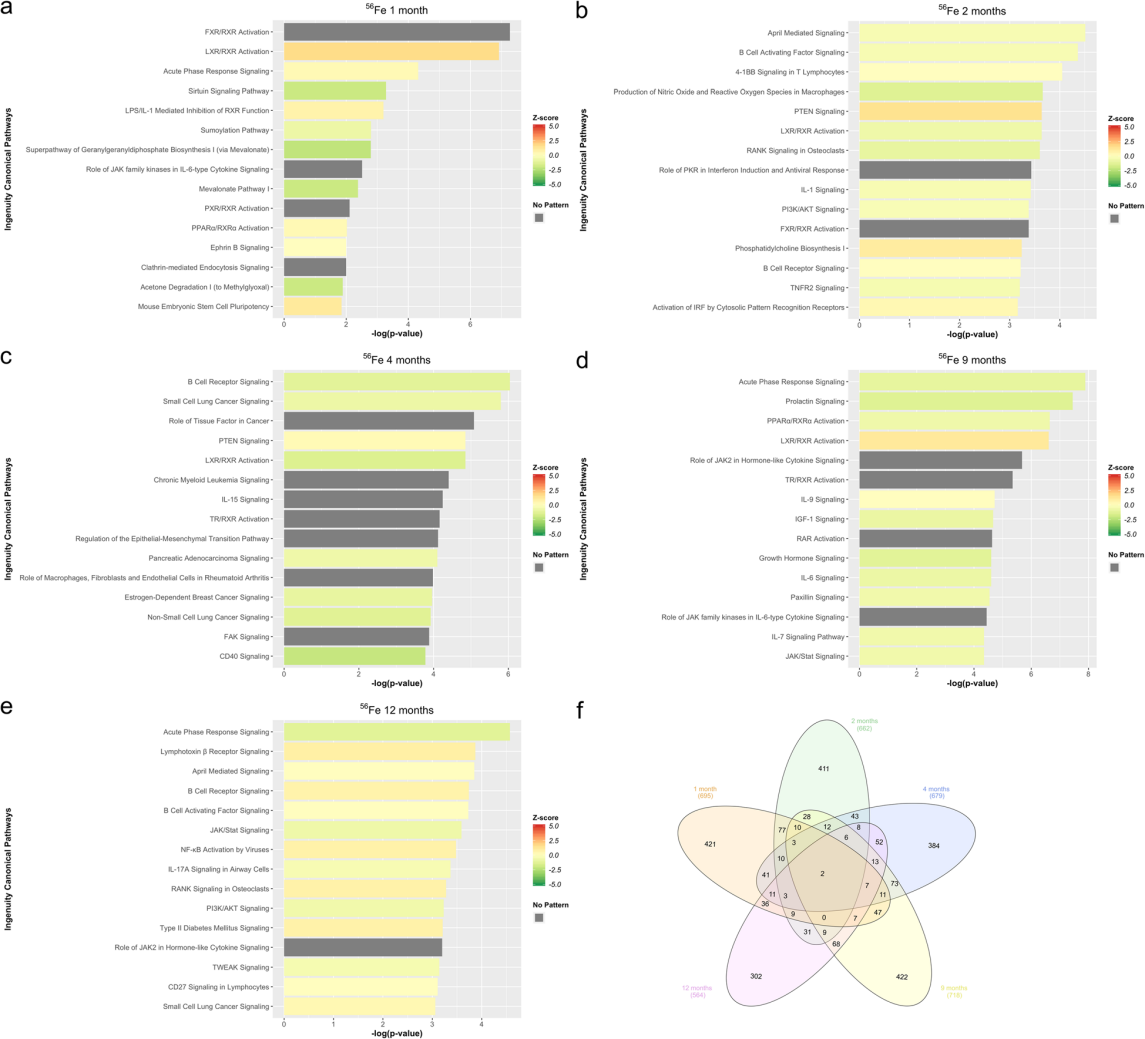
IPA of differentially expressed transcripts in ^56^Fe. **a** Top pathways enrichment analysis at 1 month. **b** Top pathways enrichment analysis at 2 months. **c** Top pathways enrichment analysis at 4 months. **d** Top pathways enrichment analysis at 9 months. **e**Top pathways enrichment analysis at 12 months. **f** The Venn Diagram shows shared and unique differentially expressed transcripts for all time points, in ^56^Fe irradiation compared to control

Transcriptional changes and altered pathways associated with ^56^Fe induced hepatic carcinogenesis were evaluated using differential expression analysis of RNA-Seq data in ^56^Fe irradiated compared to non-irradiated control mice at five different time points (1mo, 2mo, 4mo, 9mo, and 12mo). Table 1 shows the total number of differentially expressed transcripts at each time point. IPA was used to functionally annotate and map the biological processes involving these differentially expressed transcripts (Fig. 1). Inflammatory pathways and their temporal importance in irradiation-induced tissue injury are poorly understood. In this regard, the analyses revealed significant activation of acute-phase response signaling at 1 month, followed by significant inhibition of this pathway at 2, 4, 9, and 12 months. The microenvironment present early after ^56^Fe irradiation is proinflammatory and results in the activation of inflammatory pathways, such as acute phase response signaling. This is a rapid inflammatory response that provides protection against noxious stimuli using non-specific defense mechanisms [19–21]. Tissue inflammation can naturally subside over time, but a significant suppression of inflammatory genes, which we see in our data, is characteristic of induced capillary remodeling and angiogenesis [22]. The prominent inhibition of acute phase response signaling at later time points compared to non-irradiated animals suggests that impaired immune response and regulation are involved in accelerated hepatic carcinogenesis in these mice. Similarly, the peroxisome proliferator-activated receptor α (PPARα), a ligand-activated transcription factor that belongs to the family of nuclear receptors, is significantly affected at 1 month (activated), 2 months (inhibited), 4 months (inhibited), 9 months (inhibited), and 12 months (activated). PPARα has a prominent role in fatty acid oxidation, where it can exert an anti-inflammatory and anti-oxidative effect. Its activation at 1 and 12 months suggest that there is an early inflammatory response that recurs later due to the progression of carcinogenic processes [23–25].

**Table 1:** Differentially Expressed Transcripts. Total DE shows the total number of differentially expressed transcripts (FDR ≤ 0.05 & fold change ≥2) for each HZE ion at 5 different time points

Ion	Time	Total DE	Upregulated	Downregulated
^**56**^**Fe**	**1 mo**	695	304	391
^**56**^**Fe**	**2 mo**	662	300	362
^**56**^**Fe**	**4 mo**	679	325	354
^**56**^**Fe**	**9 mo**	718	374	344
^**56**^**Fe**	**12 mo**	564	304	260
^**16**^**O**	**1 mo**	710	384	326
^**16**^**O**	**2 mo**	615	298	317
^**16**^**O**	**4 mo**	588	328	260
^**16**^**O**	**9 mo**	602	332	270
^**16**^**O**	**12 mo**	796	504	292
^**28**^**Si**	**1 mo**	849	407	442
^**28**^**Si**	**2 mo**	699	319	380
^**28**^**Si**	**4 mo**	902	400	502
^**28**^**Si**	**9 mo**	679	381	298
^**28**^**Si**	**12 mo**	628	328	300

B cell receptor signaling (BCR) is significantly affected at months 2 (directionality unknown), 4 (inhibited), 9 (inhibited), and 12 (activated). Activation of BCR signaling inhibits apoptosis in B cells [26]. This observation is supported in a previous study, which demonstrated that ^56^Fe irradiation increased the incidence of murine acute myeloid leukemia (AML) and HCC [4]. Furthermore, PI3K/AKT signaling is significantly affected at 2 months (inhibited), 4 months (directionality unknown), 9 months (activated), and 12 months (inhibited). AKT has two distinct mechanisms of action. First, it can have an inhibitory role, such as inhibiting apoptosis and allowing for cell survival. Second, it can have an activating role by activating IKK, which in turn leads to NF-κB activation and cell survival [27–29]. The analysis also revealed significant activation of the Liver X receptor (LXR)/Retinoid X Receptor (RXR) pathway at 1 and 9 months accompanied by inhibition at 2- and 4-months post ^56^Fe irradiation. Previous studies have shown LXRs to be key modulators of both lipid metabolism and inflammatory signaling [30], as well as inducers of genes involved in the inhibition of inflammatory pathways [31]. The presence of this complex and coordinated time-dependent interplay between pro- and anti-inflammatory signaling pathways post ^56^Fe irradiation could play a significant role in ^56^Fe irradiated induced hepatic carcinogenesis. A complete list of significant pathways (−log_10_(*p*-value) ≥ 1.3) is provided in Supplemental Tables 1, 2, 3, 4, and 5.

### Identification of dysregulated molecular pathways corresponding to unannotated transcripts associated with ^56^Fe irradiation, using SOM

**Fig. 2:**
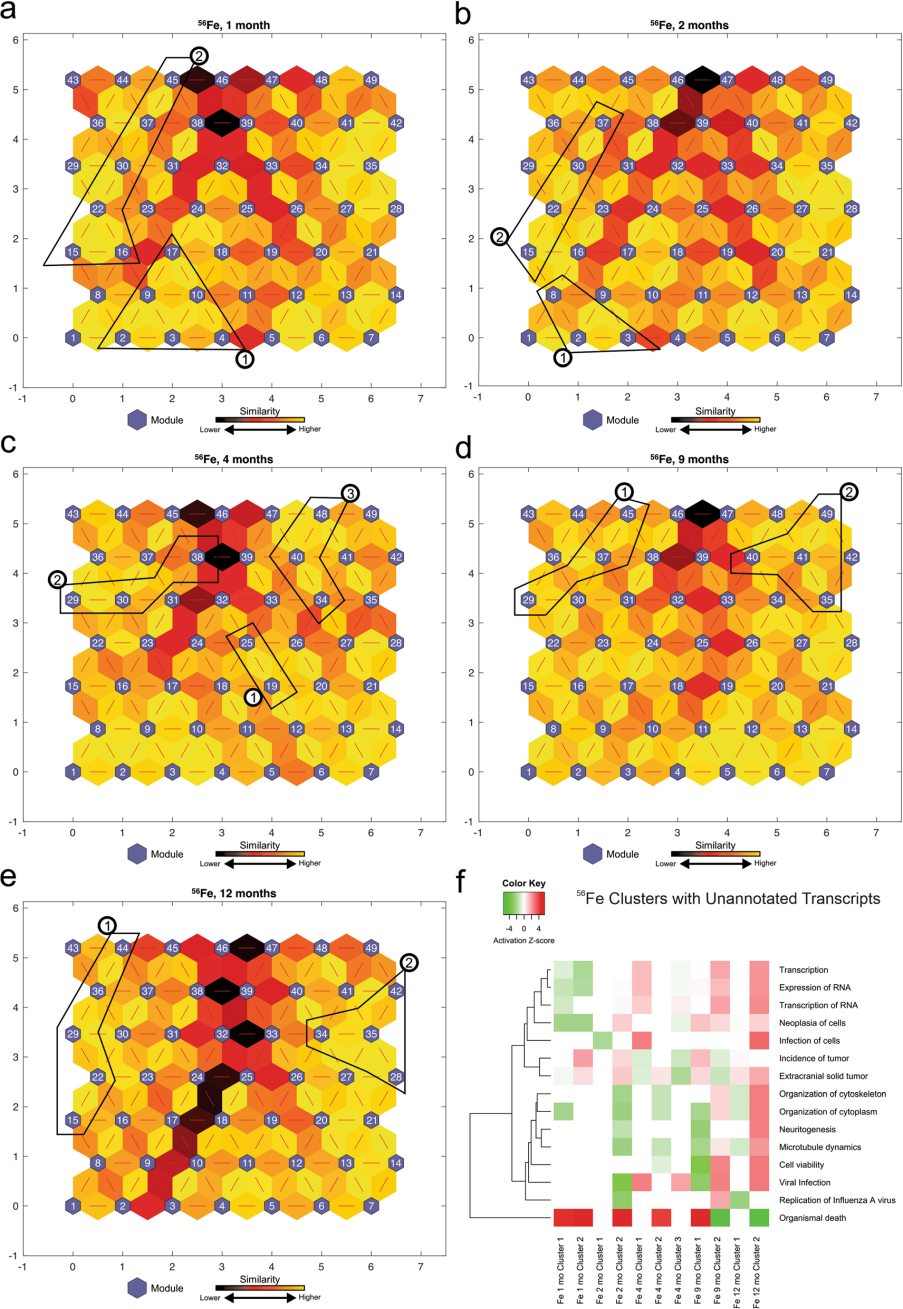
^56^Fe analysis of self-organizing maps for each time point. **a,b,c,d,e** Kohonen Self-Organizing Map (SOM) was applied to the differentially expressed (DE) transcripts obtained from the RNA-Seq data to identify coherent patterns of transcript expression at each time point, as well as patterns within the unannotated transcripts. The SOM clusters transcripts in each module according to log_2_(fold change) of the expression values. SOM clustering analysis demonstrates the distances between correlated transcript groups. The small blue hexagons are modules comprising transcripts with similar log_2_(fold change) expression patterns. The numbers of transcripts in each module are provided in Supplemental Fig. 1. Neighboring modules are connected with a red line. The colors of the lines connecting the modules indicate the similarity between modules: Lighter colors represent higher similarity, and darker colors represent lower similarity. **f** Expression patterns of unannotated transcripts were identified, and the corresponding modules (represented in circled numbers) were further analyzed by IPA. Only the most significant pathways across all clusters are shown with available color-coded activation z-scores. Inhibitory, activation, or unknown directionality z-scores correspond to green, red, and white, respectively. The entries with white color indicate the directionality could not be predicted based on the available data, yet the pathway is significantly identified by pathway analysis. The goal of the IPA downstream effects analysis is to identify functional pathways whose activity is expected to be increased or decreased, given the observed expression changes in a user’s dataset (see Methods)

The above IPA analysis (Fig. 1) resulted in a collection of 67 statistically significant-high-quality unannotated transcripts across all time points from ^56^Fe irradiated mice (Table 2). To characterize the unannotated transcripts, we obtained the log_2_ (fold change) expression values of significantly differentially expressed transcripts from ^56^Fe irradiation compared to non-irradiated control across 5 time points and applied the SOM machine learning algorithm. Next, we identified the modules from SOMs, which contained the majority of unannotated transcripts and combined them to form larger clusters of similar transcription patterns for functional analysis using IPA. We compared the identified 11 clusters across 5 time points and selected the most significant pathways across all clusters (Fig. 2f). The activation z-scores were predicted for some of the clusters based on our observed data and the available literature. The Fe 1-month Clusters have an activated positive z-score for organismal death and an inhibited negative z-score for RNA transcription and cell neoplasia. These observations are in line with our current understanding of early cellular response to irradiation and production of reactive oxygen species at earlier time points and activation of neoplasia at later time points. Clusters of unannotated transcripts show inhibition of pathways involved in RNA expression and transcription at 1 month, and activation of these pathways at 9 and 12 months. A complete list of unannotated transcript ENSMBL IDs with their corresponding module numbers is provided in Supplemental Table 6.

**Table 2:** Number of unannotated transcripts analyzed by IPA

Ion	1 month	2 months	4 months	9 months	12 months	Total
^**56**^**Fe**	16	16	13	8	14	67
^**16**^**O**	24	23	13	13	22	95
^**28**^**Si**	19	14	17	12	19	81

### Differential expression analysis of ^16^O reveals dynamic time-dependent changes in inflammatory response at the whole transcriptome level

**Fig. 3:**
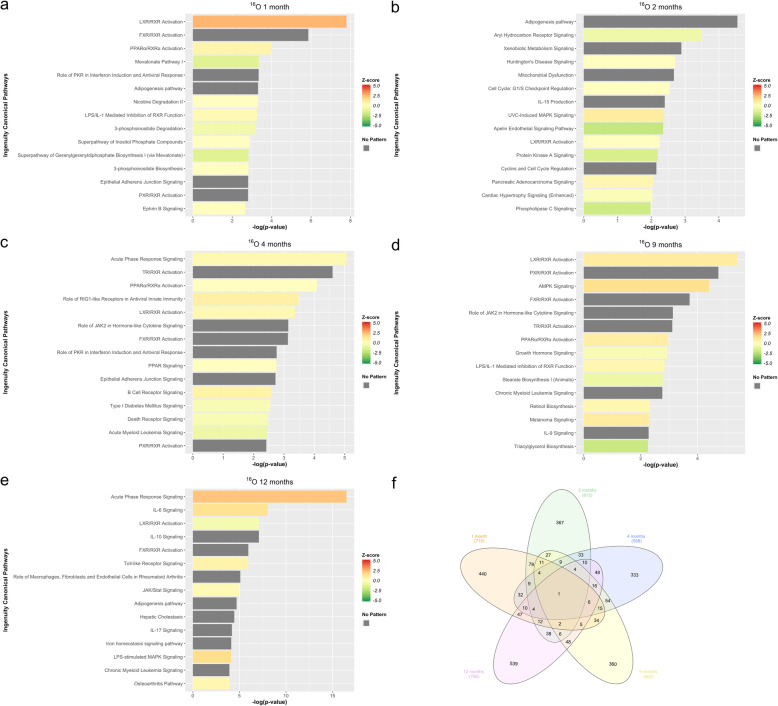
IPA of differentially expressed transcripts in ^16^O. **a** Top pathways enrichment analysis at 1 month. **b** Top pathways enrichment analysis at 2 months. **c** Top pathways enrichment analysis at 4 months. **d** Top pathways enrichment analysis at 9 months. **e**Top pathways enrichment analysis at 12 months. **f** The Venn Diagram shows shared and unique differentially expressed transcripts for all time points, in ^16^O irradiation compared to control

Transcriptional changes and altered pathways associated with proposed ^16^O induced hepatic carcinogenesis were evaluated using differential expression analysis of RNA-Seq data in ^16^O irradiated compared to non-irradiated control mice at 5 different time points (1mo, 2mo, 4mo, 9mo, and 12mo). Table 1 shows the total number of differentially expressed transcripts at each time point. IPA was used to functionally annotate and map the biological processes involving these differentially expressed transcripts (Fig. 3). The analyses revealed that the LXR/RXR pathway is significantly affected at all time points; specifically, at 1 month (activated), 2 months (directionality unknown), 4 months (activated), 9 months (activated), and 12 months (inhibited). These results indicate that ^16^O irradiation shows a time-dependent inflammatory response, similar to that of ^56^Fe. Similarly, PPARα is significantly affected at 1 month (activated), 4 months (directionality unknown), 9 months (activated), and 12 months (activated). This suggests that, even with a time-dependent inflammatory response, ^16^O tend to exert a more potent activation of inflammatory pathways as compared to ^56^Fe. Furthermore, Interleukin 8 (IL-8) signaling is significantly activated at 12 months but inhibited at 2 months. IL-8 is a member of the C-X-C family of chemokines and plays a central role in angiogenesis, tumor growth, and inflammation. IL-8 upregulates the expression of genes involved in tumor growth, angiogenesis, and tumor invasion. IL-8 also enhances cell proliferation by activating cyclin D via a protein kinase B (PKB/Akt) mediated mechanism [32–34].

Our results show activation of LPS/IL-1 mediated inhibition of RXR function pathway at 1, 9, and 12 months. The RXR plays a role in the following cascade of biological events. Binding of the CD14/TRL4/MD2 receptor complex to toxins promotes the secretion of proinflammatory cytokines (IL-1, TNFα) in different cell types, but especially in macrophages. Liver tissue injury downregulates the expression of hepatic specific genes, known as negative hepatic acute phase response (APR). Most of these repressed genes are regulated by retinoid X receptors (RXRs), which dimerizes with LXR. RXRs undergo nuclear export and therefore inhibited in response to proinflammatory cytokines (i.e., IL-1) initiated by the stimuli, and this export leads to impaired lipid metabolism and signaling [19, 35, 36]. The impaired lipid metabolism induced by ^16^O irradiation is furthered demonstrated by the adipogenesis pathway, which was significantly affected at 1, 2, 9, and 12 months (directionality/z-score unknown). Adipogenesis, adipocyte differentiation, is a complicated cellular process that is tightly regulated by a number of transcription factors, lipids, hormones, and signaling pathway molecules [37–39]. In addition, similar to the case with ^56^Fe, BCR is affected at 1 month (directionality unknown), 2 months (inhibited), 4 months (activated), 9 months (inhibited), and 12 months (activated). Activation of BCR at 12 months reduces apoptosis, which could further play a role in hepatic carcinogenesis. This is bolstered by the significant activation of the chronic myeloid leukemia signaling (CML) pathway at all time points, triggered by expression of the BCR gene product. The transcriptional changes in CML involve genes that result in cell proliferation [40–42]. A complete list of statistically significant altered pathways (−log_10_(*p*-value) ≥ 1.3) is provided in Supplemental Tables 7, 8, 9, 10, and 11.

### Identification of dysregulated molecular pathways corresponding to unannotated transcripts associated with ^16^O irradiation, using SOM

**Fig. 4:**
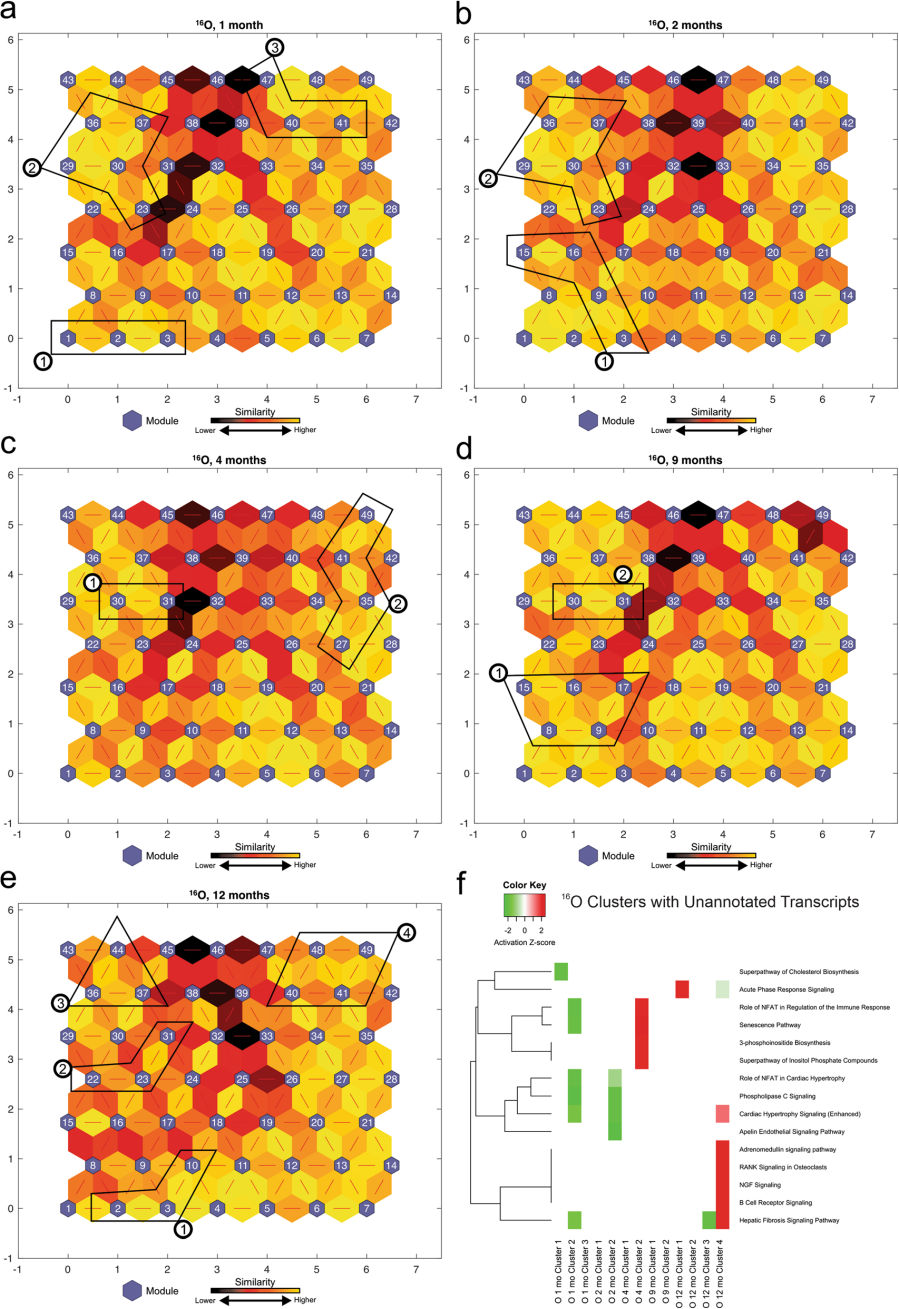
^16^O analysis of self-organizing maps for each time point. **a,b,c,d,e** Kohonen Self-Organizing Map (SOM) was applied to the differentially expressed (DE) transcripts obtained from the RNA-Seq data to identify coherent patterns of transcript expression at each time point, as well as patterns within the unannotated transcripts. The SOM clusters transcripts in each module according to log_2_(fold change) of the expression values. SOM clustering analysis demonstrates the distances between correlated transcript groups. The small blue hexagons are modules comprising transcripts with similar log_2_(fold change) expression patterns. The numbers of transcripts in each module are provided in Supplemental Fig. 2. Neighboring modules are connected with a red line. The colors of the lines connecting the modules indicate the similarity between modules: Lighter colors represent higher similarity, and darker colors represent lower similarity. **f** Expression patterns of unannotated transcripts were identified, and the corresponding modules (represented in circled numbers) were further analyzed by IPA. Only the most significant pathways across all clusters are shown with available color-coded activation z-scores. Inhibitory, activation, or unknown directionality z-scores corresponds to green, red, and white respectively. The entries with white color indicate the directionality could not be predicted based on the available data, yet the pathway is significantly identified by pathway analysis. The goal of the IPA downstream effects analysis is to identify functional pathways whose activity is expected to be increased or decreased, given the observed expression changes in a user’s dataset (see Methods)

The above IPA analyses (Fig. 3) resulted in a collection of 95 statistically significant-high-quality unannotated transcripts across all time points from ^16^O irradiated mice (Table 2). To characterize the unannotated transcripts, we obtained the log_2_(fold change) expression values of differentially expressed transcripts from ^16^O irradiation compared to non-irradiated control across 5 time points and applied the SOM machine learning algorithm. We next identified the modules from SOMs, which contained the majority of unannotated transcripts and combined them to form larger clusters of similar transcription patterns for functional analysis using IPA. We compared the identified 13 clusters across 5 time points using IPA (Fig. 4f). Figure 4f shows the most significant pathways across all clusters. The activation z-scores were predicted for some of the clusters based on our observed data and the available literature. The clusters of unannotated transcripts tended to show inhibitory responses with negative z-scores at 1 and 2 months, and activation at later time points. Even though the directionality could not be determined for some of these pathways, some of the identified significant pathways are similar to those previously observed in Fig. 3 and are involved in immune response (B cell receptor signaling and acute phase response signaling), cholesterol biosynthesis, and the hepatic fibrosis signaling pathway. A complete list of unannotated transcript ENSMBL IDs with their corresponding module numbers is provided in the Supplemental Table 12.

### Differential expression analysis of ^28^Si reveals dynamic time-dependent changes in inflammatory response at the whole transcriptome level

**Fig. 5:**
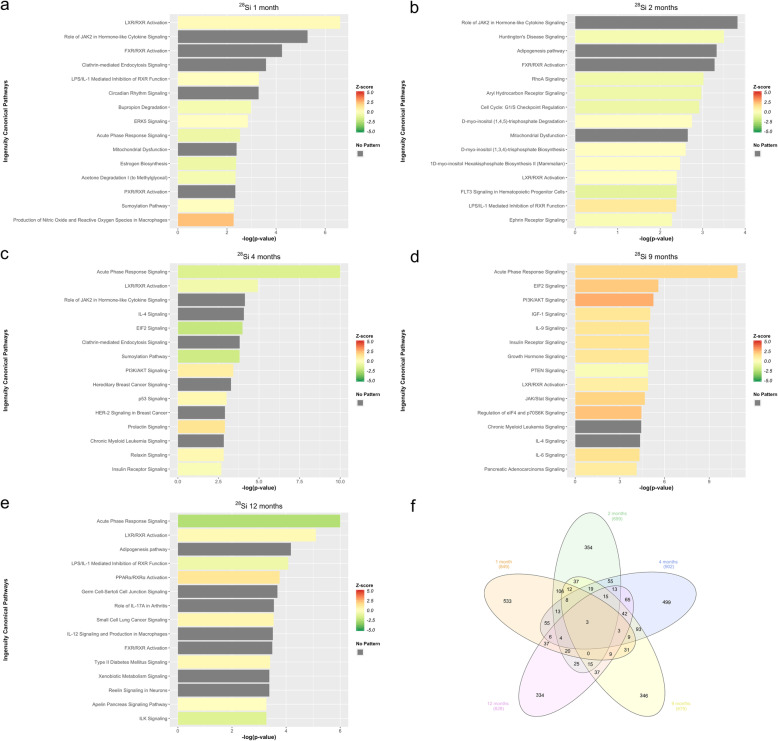
IPA of differentially expressed transcripts in ^28^Si. **a** Top pathways enrichment analysis at 1 month. **b** Top pathways enrichment analysis at 2 months. **c** Top pathways enrichment analysis at 4 months. **d** Top pathways enrichment analysis at 9 months. **e**Top pathways enrichment analysis at 12 months. **f** The Venn Diagram shows shared and unique differentially expressed transcripts for all time points, in ^28^Si irradiation compared to control

Transcriptional changes and altered pathways associated with proposed ^28^Si induced hepatic carcinogenesis were evaluated using differential expression analysis of RNA-Seq data in ^28^Si irradiated compared to non-irradiated control mice at 5 different time points (1mo, 2mo, 4mo, 9mo, and 12mo). Table 1 shows the total number of differentially expressed transcripts at each time point. IPA was used to functionally annotate and map the biological processes involving these differentially expressed transcripts (Fig. 5). The analyses revealed that LXR/RXR is significantly affected at 1 month (activated), 2 months (directionality unknown), 4 months (inhibited), 9 months (activated), and 12 months (activated). The acute phase response signaling pathway demonstrated a different dynamic post ^28^Si irradiation as compared to ^56^Fe. In particular, it was significantly inhibited at 1, 4, and 12 months and activated at 9 months. In addition, IL-8 signaling shows a pattern opposite to that of ^16^O irradiation. An IL-8 signaling pathway is significantly activated at 4 months, while unlike ^16^O irradiation, it is inhibited at 12 months. Furthermore, PI3K/AKT signaling was significantly activated at 1, 4, and 9 months post ^28^Si irradiation. This might suggest that ^28^Si has an earlier cellular survival response compared to ^56^Fe and ^16^O. Additionally, the results show that aryl hydrocarbon receptor signaling is significantly inhibited at 2, 4, 9, and 12 months post ^28^Si irradiation. Aryl hydrocarbon receptor (AHR) is a cytosolic protein associated with chaperone and immunophilin-like protein. Upon ligand activation, AHR dissociates from the complex, translocates into the nucleus and induces transcriptional activation of genes in various signaling pathways involved in cell cycle progression, tumorigenesis, apoptosis, and cell proliferation [43–45].

The analyses revealed that BCR signaling was significantly affected at 1 month (inhibited), 2 months (activated), 4 months (inhibited), 9 months (activated), and 12 months (activated). This is also indicative of a stronger inhibitory apoptosis response later in time after ^28^Si irradiation. In addition, the production of nitric oxide and reactive oxygen species in macrophages were significantly affected at all time points, specifically, at 1 month (activated), 2 months (activated), 9 months (activated), and 12 months (inhibited). The tumoricidal properties of macrophages are dependent on the production of reactive oxygen species (ROS). Production of ROS happens through the activation of the nicotinamide adenine diphosphate oxidase (NADPH oxidase), which is part of the electron transport chain. Factors such as bacterial products and metabolites can activate NADPH oxidase, which will lead to ROS production in macrophages and help defend against noxious stimuli [46–48]. The inhibition of ROS production at 12 months contributes to the carcinogenic process triggered by ^28^Si irradiation. This process is especially pronounced during later time points when the immune response cannot properly regulate apoptosis or control tissue damage. Moreover, Insulin-like growth factor-1 (IGF-1) signaling, which promotes cell proliferation, growth, and survival, is significantly activated at 4, and 9 months. IGF-1 receptor is a transmembrane tyrosine kinase protein that activates many downstream pathways, which in turn induce genes that promote cell growth and differentiation, as well as pathways for cell survival [49–51]. IGF-1 targeted antibodies are currently under phase I clinical investigation as anticancer therapeutic drugs for advanced or refractory solid tumors (NCT03746431). These pathways demonstrate a complex dynamic interplay with different immunological pathways after ^28^Si irradiation, which could contribute to hepatic carcinogenic processes. A complete list of significantly impacted pathways (−log_10_(*p*-value) ≥ 1.3) is provided in Supplemental Tables 13, 14, 15, 16, and 17.

### Identification of dysregulated molecular pathways corresponding to unannotated transcripts associated with ^28^Si irradiation, using SOM

**Fig. 6:**
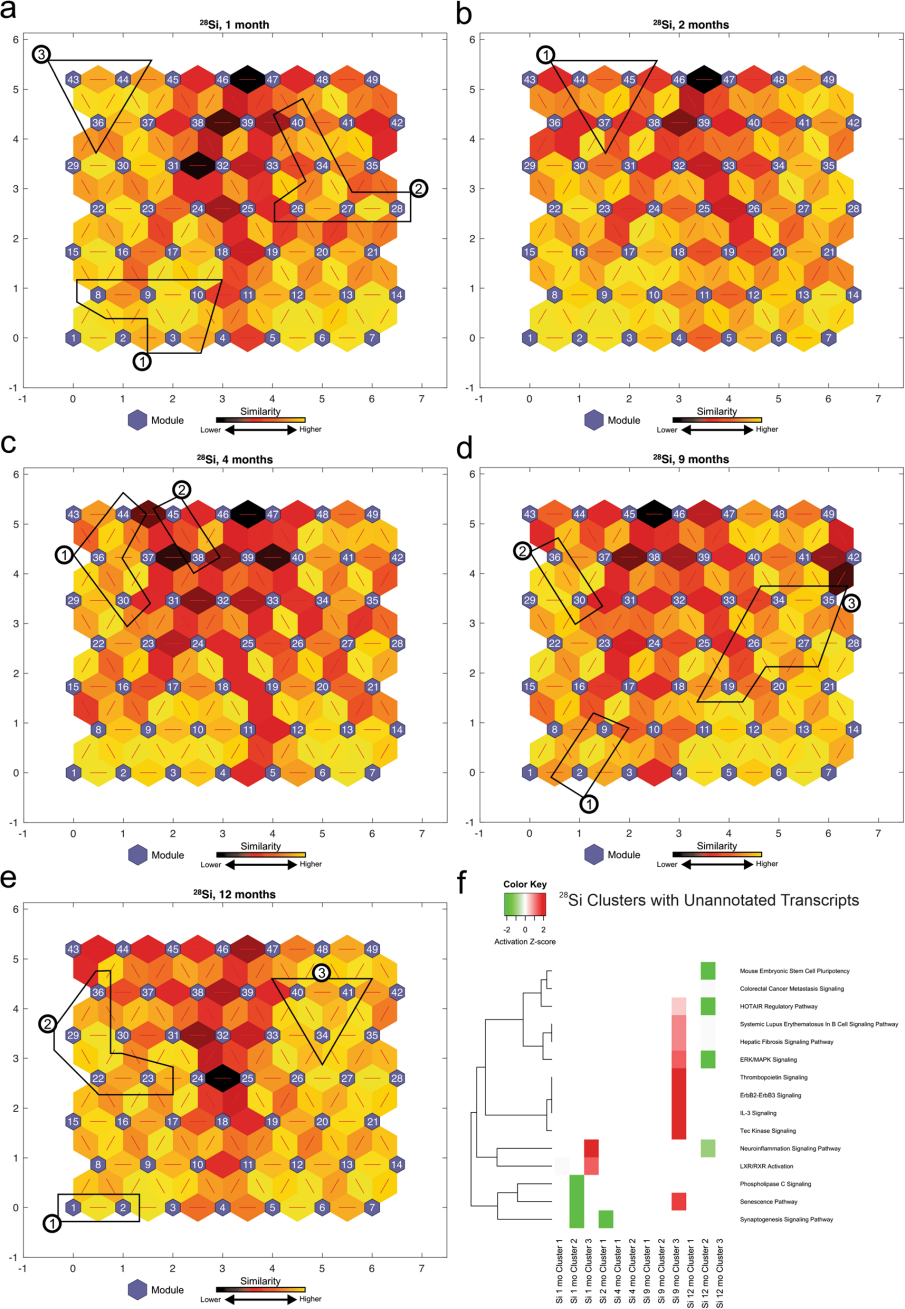
^28^Si analysis of self-organizing maps for each time point. **a,b,c,d,e** Kohonen Self-Organizing Map (SOM) was applied to the differentially expressed (DE) transcripts obtained from the RNA-Seq data to identify coherent patterns of transcript expression at each time point, as well as patterns within the unannotated transcripts. The SOM clusters transcripts in each module according to log_2_(fold change) of the expression values. SOM clustering analysis demonstrates the distances between correlated transcript groups. The small blue hexagons are modules comprising transcripts with similar log_2_(fold change) expression patterns. The numbers of transcripts in each module are provided in Supplemental Fig. 3. Neighboring modules are connected with a red line. The colors of the lines connecting the modules indicate the similarity between modules: Lighter colors represent higher similarity, and darker colors represent lower similarity. **f** Expression patterns of unannotated transcripts were identified, and the corresponding modules (represented in circled numbers) were further analyzed by IPA. Only the most significant pathways across all clusters are shown with available color-coded activation z-scores. Inhibitory, activation, or unknown directionality z-scores corresponds to green, red, and white respectively. The entries with white color indicate the directionality could not be predicted based on the available data, yet the pathway is significantly identified by pathway analysis. The goal of the IPA downstream effects analysis is to identify functional pathways whose activity is expected to be increased or decreased, given the observed expression changes in a user’s dataset (see Methods)

The above IPA analysis (Fig. 5) resulted in a collection of 81 statistically significant-high-quality unannotated transcripts across all time points from ^28^Si irradiated mice (Table 2). To characterize the unannotated transcripts, we obtained the log_2_ (fold change) expression values of significantly differentially expressed transcripts from ^28^Si irradiation compared to non-irradiated control across 5 time points and applied the SOM machine learning algorithm. We next identified the modules from SOMs, which contained the majority of unannotated transcripts and combined them to form larger clusters of similar transcription patterns for functional analysis using IPA. We compared the identified 12 clusters across 5 time points using IPA (Fig. 6f). Figure 6f shows the most significant pathways across all clusters. The activation z-scores were predicted for some of the clusters based on our observed data and the available literature. Even though the directionality could not be determined for some of these pathways, the significant pathways included B cell signaling, hepatic fibrosis signaling, tec kinase signaling, neuroinflammation signaling, LXR/RXR activation, phospholipase C signaling, and the senescence pathway. A complete list of unannotated transcript ENSMBL IDs with their corresponding module numbers is provided in the Supplemental Table 18.

## Discussion

Despite the knowledge that deep spaceflight is associated with multiple carcinogenic processes, the different responses to HZE irradiation are still relatively unexplored. This study was designed to help identify the molecular mechanisms of HZE induced HCC focusing on transcription expression patterns at different time points after irradiation and to elucidate novel unannotated transcripts that are significantly affected by HZE-irradiation. It has been hypothesized that a major driver of HZE induced carcinogenesis occurs through inflammatory responses, reactive oxygen species, and DNA damage [52]. Our results support an association between early proinflammatory response, downstream biomarkers of cytokine activity, and downregulation of such responses at later time points. The exact molecular factors that regulate these responses are not well defined, but HZE-irradiation engenders a complex immune response where directionality (activation/inhibition) cannot be predicted for some pathways.

We observed some significant commonly dysregulated immunological pathways in the HZE-irradiated mice, including PI3K signaling in B lymphocytes, acute phase response signaling, IL-8 signaling, IL-7 signaling, IL-3 signaling, B cell receptor signaling, and PPARα signaling. PI3K was mainly activated at later time points across all HZE ions. PI3K regulates numerous biological functions such as survival, differentiation, proliferation, migration, and metabolism. In the immune system, inhibited PI3K leads to immunodeficiency, whereas activation of this signaling cascade leads to leukemia and autoimmune responses [50, 53, 54]. The acute phase response signaling was activated at 1 month in ^56^Fe but inhibited at this time point for both ^16^O and ^28^Si. This response is triggered by the initiation of irradiation-induced tissue injury, which leads to changes in the concentration of several plasma proteins as a result of significantly altered hepatic metabolism [19–21]. It has been previously shown that ^16^O total body irradiation significantly decreases peripheral blood cell counts in mice as early as 2 weeks post-irradiation, particularly white blood cells (WBC) and platelets (PLT) [5]. This rapid depletion of peripheral WBC can be a potential contributor to an impaired acute phase response in ^16^O and ^28^Si irradiated mice through a similar mechanism. Additionally, IL-8 signaling was activated at 12 months post ^56^Fe and ^16^O irradiation, while it was inhibited in ^28^Si. Given that IL-8 upregulates the expression of genes involved in tumor growth (EGFR, MMP2, MMP9), angiogenesis (VEGF), and cell proliferation through a metalloproteinase dependent pathway [32–34, 55, 56], its activation at 12 months post ^56^Fe and^16^O irradiation is in line with the tumor growth and spontaneous incidences of HCC seen previously [4, 32–34, 56]. It has been previously shown that ^28^Si increases the levels of apoptotic cell death in the heart and bone marrow up to 6 months post-irradiation [8]. This chronic apoptotic response might be associated with the observed IL-8 suppression. Moreover, hepatic nuclear receptor PPARα affects various aspects of energy homeostasis, including cholesterol and fatty acid metabolism [57]. It has been previously reported that mice lacking PPARα accumulate hepatic triglycerides resembling that of nonalcoholic fatty liver disease (NAFLD) [58–61]. On the one hand, significant inhibition of this pathway, as seen in ^56^Fe, ^16^O, and ^28^Si post-irradiation at some of the time points, might indicate that other liver injuries and the consequent liver diseases such as NAFLD can arise as a result of HZE ion exposure. On the other hand, this might indicate that HCC pathogenesis involves some similar/common key players as other liver diseases such as NAFLD.

Nonetheless, as mentioned earlier, the focus of this study was limited to transcriptional changes induced in the liver by ^56^Fe,^16^O, and ^28^Si irradiation at 5 different time points. Hence, it remains unclear how the detected changes reflect the magnitude of carcinogenic processes in the liver. In future studies, it is therefore important to investigate these differences by conducting a comparison between both histologically and quantitively, in addition to measuring the different levels of enzymes/proteins responsible for the indicated pathways. A complete list of comparison analyses with predicted z-scores for significant pathways comparing between all HZE types of irradiated mice across all time points is provided in the Supplemental Table 19.

Moreover, to assess the transcriptional pathways of our novel unannotated transcripts, we examined their activity patterns across five time points utilizing SOMs. To elucidate the biological functions associated with these transcript clusters, we then performed functional pathway analyses (Figs. 2, 4, and 6). The deep mining of biological knowledge from these unannotated transcripts remains challenging due to the incompleteness of genome functional annotation. The SOM machine learning methodology takes advantage of already annotated and studied transcripts and pathways to infer the biological functions of the unannotated transcripts. Future studies should assess the transcriptional and regulatory activity of these unannotated transcripts using different techniques such as histone modifications (H3K4me3 and H3K27ac), which have been associated with activation of transcription and enhancer activity, respectively [62, 63]. Some of these unannotated transcripts may originate from enhancer regions or promotor upstream transcripts and thus play key regulatory roles in controlling gene expression following HZE irradiation since they are significantly affected by irradiation. Additionally, aligning these significant unannotated transcripts to the human genome will help identify those that are conserved in humans. Even though the precise functions of our unannotated transcripts remain to be elucidated, their significant changes post-HZE-irradiation, their similar expression patterns with the annotated genes in specified modules and neighboring modules in the described SOMs, and their functional roles in transcription activity, organismal death, hepatic fibrosis signaling, and LXR/RXR signaling pathways, all provide compelling evidence to support further studies of the roles of these transcripts in the carcinogenic processes of HCC following low-dose HZE irradiation.

## Conclusions

^56^Fe,^16^O, and ^28^Si are all major HZE contributors in the space radiation environment, yet the differences in biological effects (both acute and chronic) of these HZE ions after total body irradiation in mice remain largely unexplored. To understand the molecular mechanisms of HZE-induced HCC, we investigated the effects of ^56^Fe,^16^O, and ^28^Si ions irradiation on transcript expression utilizing RNA-Seq data collected from the livers of mice at five different time points post-irradiation. Our findings revealed an early activation of proinflammatory response along with various cytokine activities, and inhibition of these responses at later time points post-irradiation. Additionally, our results revealed a number of unannotated transcripts that were significantly affected post-low-dose HZE irradiation, and their associations with specific functional pathways. Taken together, these findings provide leads regarding potentially important new transcripts and transcriptional products, which could lead to the identification of novel countermeasures and therapeutic targets. Identification of novel transcriptional products may be accomplished by in silico translation of unannotated transcripts into amino acid sequences, which can be used to search Data Independent Acquisition (DIA) proteomics datasets from similar studies. This will enable the identification of novel transcriptional products.

## Methods

### Animal experiments and sample preparation

C3H/HeNCrl mice purchased from Charles River (Wilmington, MA) were used in this experiment since they have been shown to be a suitable experimental model for liver carcinogenesis. The C3H/HeNCrl strain was used based on previous studies demonstrating that these mice are sensitive to the induction of HCC after exposure to a dose of 0.2 Gy of 600 MeV/n ^56^Fe [4]. It is imperative to conduct tumor induction studies in whole animals to study the microenvironmental effects of HZE exposure and characterize the molecular changes in the irradiated tissues because computer models or cell culture are inadequate based on extensive literature searches. Conducted studies were approved by the institutional animal care and use committees (IACUCs). The power for this study was set at 80%, which determined the number of animals used based on the chi-square test for comparing two proportions, with a two-sided significance level set at 0.05.

A total of 60 8 to 10-week-old male mice were used for this study. The serial sacrifice study consisted of 15 male mice with 3 mice per time point. In particular, five times points which included 30, 60, 120, 270, and 360 days post-exposure. The four groups included three treatments (600 MeV/n ^56^Fe (0.2 Gy), 1 GeV/n ^16^O (0.2 Gy), and 350 MeV/n ^28^Si (0.2 Gy)) and one control (non-irradiated/sham irradiated). The mice were housed at the Brookhaven National Laboratories (BNL) animal facility until irradiation treatment at the NASA Space Radiation Laboratory. Following irradiation, the animals were shipped to the Animal Resources Center at UTMB, quarantined for 1 month, and maintained for the remainder of the experiment. The mice were housed in sterile cages and had free access to food and water. Facilities at both BNL and UTMB used for animal housing are fully AAALAC accredited. Selection of animals for sacrifice at each of the 5 time points and preparation of the left love of livers were performed as previously described [64].

### Acquisition of RNA-Seq data

Total RNA was isolated from the liver slices using RNAqueous™ Total RNA Isolation Kit (ThermoFisher Scientific, Waltham, MA), and rRNA was removed using the Ribo-Zero™ rRNA Removal Kit (Illumina, San Diego, CA). Library preparation and sequencing were performed, as previously described [64]. CLC Genomics Workbench v12.0.3 was used for bioinformatical quality control and mapping of the RNA-Seq data. Sequencing data was initially trimmed using the CLC’s “Trim Reads” module. Reads containing nucleotides below the quality threshold of 0.05 (using the modified Richard Mott algorithm), those with two or more unknown nucleotides or sequencing adapters were trimmed out. Additionally, all reads have been trimmed by 14 bases from the 5′ end of each read. The total number of reads used in analysis varied between 33 and 114 million. A complete list of sample reads information is available in the Supplemental Table 20. Filtered sequencing reads were then processed using the “RNA-Seq Analysis” module. Reads were mapped using a global alignment strategy against the mouse GRCm38 reference genome with 95% length fraction and similarity fraction scores with annotation version GRCm38.97.

### Differential transcript expression analysis

Raw abundance counts of 107,319 mRNAs from 15 non-irradiated control, 15 ^56^Fe irradiated, 15 ^16^O irradiated, and 15 ^28^Si irradiated C3H/HeNCrl male mice liver tissue samples were subjected to differential transcript expression analysis. Differential transcript expression analysis was performed as previously described using edgeR [64–66]. Statistical tests were then conducted at every time point, to compare between ^56^Fe irradiated and non-irradiated control, ^16^O irradiated and non-irradiated control, and ^28^Si irradiated and non-irradiated control samples using a quasi-likelihood negative binomial generalized log-linear model for count data [67–69]. The Benjamini-Hochberg correction was applied, and transcripts with FDR ≤ 0.05 & fold change≥2 (both up and down-regulated) were extracted and utilized in further analyses.

### Functional enrichment analysis

To determine the biological functions of significantly differentially expressed (DE) transcripts, functional enrichment analysis was performed separately for the DE transcripts at each time point using Ingenuity Pathway Analysis (IPA) (QIAGEN Inc., Hilden, Germany) [70]. The most significant functional pathways (−log10(*p-value*) ≥ 1.3) at each time point were then evaluated and reported. A complete list of all identified statistically significant pathways is provided in the Supplemental Tables 1, 2, 3, 4, 5, 7, 8, 9, 10, 11, and 13, 14, 15, 16, 17.

In order to investigate any internal biases associated with specific pathway prediction tools, we ran the same analysis using DAVID (https://david.ncifcrf.gov/) [71, 72]. DAVID provides pathways from KEGG and BIOCARTA databases. The majority of the transcripts for each treatment remained unannotated/unrelated to a pathway. In general, other pathway prediction tools were unable to annotate the transcript expression data as well as that done by IPA, although when annotated, the results obtained by DAVID were contained within the IPA analysis as well. A complete list of DAVID analyses is provided in the Supplemental Tables 21, 22, 23, 24, 25, 26, 27, 28, 29, 30, 31, 32, 33, 34, 35, 36 and 37.

### SOM analysis

Self-Organizing Map (SOM) analysis was performed to identify clusters of transcripts with similar expression patterns and was conducted for every time point analyzing pairwise comparisons of ^56^Fe irradiated and non-irradiated control, ^16^O irradiated and non-irradiated control, and ^28^Si irradiated and non-irradiated control samples. SOMs were created using the algorithm implemented in the MATLAB software Neural Networking Toolbox [www.mathworks.com] version R2018b based on inputs of Log_2_(Fold Change) from the differential transcript expression analyses data. In order to scale network inputs and outputs, we normalized our input matrix so that they had zero mean and unity standard deviation. We then processed the input matrix using principal component analysis (PCA) to reduce dimensionality. The SOM algorithm was then used to cluster the data based on similarity and topology using 100,000 training epochs. The SOM translates the differentially expressed transcriptome profile into a two-dimensional quadratic 7 × 7 pixel map and a color code for similarity values.

Next, we performed functional pathway analysis using IPA (QIAGEN Inc., Hilden, Germany) [70], on selected adjacent modules (clusters selected for IPA analysis are numbered and shown in circles on SOM maps) that contained the reported unannotated transcripts to explore their functionality based on the annotated transcripts contained within those modules (available activation z-scores, shared enriched functions of interest, and similar transcript expression patterns). We identified neighboring modules with high similarity and the most unannotated transcripts. Clusters of modules were grouped visually based on the similarity calculated from the SOM analysis (yellow being the most similar). No specific threshold was applied in the determination of the clusters of modules. The activation z-score is statistically computed by IPA for each functional pathway and is used to infer biological functions and predict implicated functional pathways. The activation z-score is predicted by assessing the consistency of the pattern between the observed gene-regulation pattern and the activation/inhibition pattern given by the network relative to a random pattern. Activation z-score calculations are accomplished independently from associated *p*-values and are based upon the match results from up/down-regulation. Given the observed differential regulation of a transcript in the dataset, the activation state is determined for each specific functional pathway, and the directionality effect is then assigned. If an activation z-score can’t be predicted for a significant pathway based on the available data, and after bias correction, NA (white color) is assigned for that specific pathway [70].

## Supplementary information

**Additional file 1.**

## Data Availability

The data discussed in this publication have been deposited in NCBI’s Gene Expression Omnibus (Nia et al., 2020) and are accessible through GEO Series accession number GSE146254 through https://www.ncbi.nlm.nih.gov/geo/query/acc.cgi?acc=GSE146254. GRCm38.97 reference genome was obtained through ftp://ftp.ensembl.org/pub/release-97/fasta/mus_musculus/. David’s analysis was performed through https://david.ncifcrf.gov/.

## Abbreviations

HZE: High Charge High Energy Ions
IPA: Ingenuity Pathway Analysis
SOM: Self Organizing Map
