# Supplementary material for: Comparative RNA-Seq transcriptome analyses reveal dynamic time-dependent effects of 56Fe, 16O, and 28Si irradiation on the induction of murine hepatocellular carcinoma
Source: BMC Genomics. 2020 Jul 1;21:453. doi: 10.1186/s12864-020-06869-4 (PMC7329445; doi:10.1186/s12864-020-06869-4)
Supplement: Supplementary file 1 — Additional file 1. [file 12864_2020_6869_MOESM1_ESM.docx]

**Supplemental Material:** 37 Supplemental Tables, 3 Supplemental Figures

| **Ingenuity Canonical Pathways** | **-log(p-value)** | **z-score** | **Molecules** |
| --- | --- | --- | --- |
| FXR/RXR Activation | 7.27 | NA | ABCB11,APOA2,BAAT,C4A/C4B,C9,CLU,FASN,FETUB,KNG1,NR0B2,NR1H3,NR1H4,PLTP,SAA1,SDC1,SERPINF1,VTN |
| LXR/RXR Activation | 6.92 | 1.732 | APOA2,C4A/C4B,C9,CLU,FASN,FDFT1,HMGCR,KNG1,NCOR2,NR1H3,NR1H4,PLTP,SAA1,SERPINF1,TLR3,VTN |
| Acute Phase Response Signaling | 4.32 | 0.333 | APOA2,C1R,C4A/C4B,C9,CP,ECSIT,FN1,HNRNPK,HP,IL6ST,MAPK14,MAPK3,SAA1,SERPINF1,TCF3 |
| Sirtuin Signaling Pathway | 3.28 | -2 | ATG13,GLUD1,MAP1LC3A,MAPK3,MAPK7,MT-ATP6,MT-CYB,MT-ND2,MT-ND3,MT-ND4,MT-ND4L,MT-ND5,MT-ND6,NR1H3,NR1H4,PFKFB3,RARB,SIRT5,TUBA4A |
| LPS/IL-1 Mediated Inhibition of RXR Function | 3.2 | 0.632 | ABCB11,ALAS1,ALDH2,CYP2B6,CYP2C8,CYP3A5,CYP4A11,ECSIT,FMO1,FMO2,HMGCS1,NR0B2,NR1H3,NR1H4,PLTP |
| Sumoylation Pathway | 2.8 | -0.816 | RCC1,RFC1,RHOBTB1,SENP5,SENP7,SERBP1,SP3,XIAP,ZNF217 |
| Superpathway of Geranylgeranyldiphosphate Biosynthesis I (via Mevalonate) | 2.79 | -2.236 | FDPS,HADHB,HMGCR,HMGCS1,MVD |
| Role of JAK family kinases in IL-6-type Cytokine Signaling | 2.51 | NA | IL6ST,MAPK14,MAPK3,TYK2 |
| Mevalonate Pathway I | 2.38 | -2 | HADHB,HMGCR,HMGCS1,MVD |
| PXR/RXR Activation | 2.11 | NA | ABCB11,ALAS1,CYP2B6,CYP2C8,CYP3A5,NR0B2 |
| PPARα/RXRα Activation | 2.02 | 0.333 | ADCY9,APOA2,CYP2C8,FASN,GPD2,MAPK14,MAPK3,MED12,NCOR2,NOTUM,NR0B2 |
| Clathrin-mediated Endocytosis Signaling | 2 | NA | AP1B1,AP1G2,APOA2,ARRB1,CD2AP,CLU,MYO1E,NUMB,PICALM,SH3GLB2,TFRC |
| Ephrin B Signaling | 2 | 0 | ABI1,CAP1,CTNNB1,EPHB4,HNRNPK,MAPK3 |
| Acetone Degradation I (to Methylglyoxal) | 1.89 | -2 | CYP2B6,CYP2C8,CYP3A5,CYP4A11 |
| Mouse Embryonic Stem Cell Pluripotency | 1.85 | 1.134 | CTNNB1,IL6ST,MAPK14,MAPK3,TCF3,TYK2,XIAP |
| Complement System | 1.85 | NA | C1R,C4A/C4B,C8A,C9 |
| Superpathway of Cholesterol Biosynthesis | 1.72 | -2.449 | FDFT1,FDPS,HADHB,HMGCR,HMGCS1,MVD |
| α-tocopherol Degradation | 1.71 | NA | CYP4A11,CYP4F3 |
| HIPPO signaling | 1.7 | 1 | AMOT,DLG1,DLG5,MOB1A,PARD3,TEAD1 |
| IL-22 Signaling | 1.68 | NA | MAPK14,MAPK3,TYK2 |
| Endocannabinoid Developing Neuron Pathway | 1.59 | 0.378 | ADCY9,CDKN1B,CREB1,CTNNB1,MAPK14,MAPK3,MAPK7 |
| Lipid Antigen Presentation by CD1 | 1.59 | NA | AP1B1,AP1G2,PSAP |
| Bupropion Degradation | 1.55 | NA | CYP2B6,CYP2C8,CYP3A5 |
| RAR Activation | 1.54 | NA | ADCY9,CYP26A1,DHRS4,MAPK14,NCOR2,PRKD3,RARB,SDR9C7,TNIP1,ZBTB16 |
| Oxidative Phosphorylation | 1.54 | 2.646 | MT-ATP6,MT-CYB,MT-ND2,MT-ND3,MT-ND4,MT-ND4L,MT-ND5 |
| Phenylalanine Degradation I (Aerobic) | 1.54 | NA | PCBD2,QDPR |
| Xenobiotic Metabolism Signaling | 1.53 | NA | ALDH2,CAMK2D,CYP2B6,CYP2C8,CYP3A5,FMO1,FMO2,HDAC4,MAPK14,MAPK3,MAPK7,NCOR2,PRKD3 |
| Systemic Lupus Erythematosus Signaling | 1.49 | NA | C8A,C9,CREM,EFTUD2,HNRNPA2B1,HNRNPC,KNG1,MAPK3,NFAT5,PRPF40B,PRPF8 |
| Endocannabinoid Cancer Inhibition Pathway | 1.47 | -2.828 | ADCY9,CDKN1B,CREB1,CTNNB1,MAPK14,MAPK3,SMPD4,TCF3 |
| UVC-Induced MAPK Signaling | 1.43 | -2 | MAPK14,MAPK3,PRKD3,SMPD4 |
| Gap Junction Signaling | 1.42 | NA | ADCY9,CTNNB1,GJB1,MAPK3,MAPK7,NOTUM,PRKD3,SP3,TUBA4A,TUBB2A |
| Nicotine Degradation II | 1.41 | -1.342 | CYP2B6,CYP2C8,CYP3A5,FMO1,FMO2 |
| Epithelial Adherens Junction Signaling | 1.4 | NA | CTNNB1,EPN2,MYH10,PARD3,RAPGEF1,TCF3,TUBA4A,TUBB2A |
| Tetrapyrrole Biosynthesis II | 1.4 | NA | ALAS1,UROS |
| Glycerol Degradation I | 1.4 | NA | Gk,GPD2 |
| 14-3-3-mediated Signaling | 1.38 | -1.342 | CDKN1B,MAPK3,NOTUM,PRKD3,TSC1,TUBA4A,TUBB2A |
| Triacylglycerol Biosynthesis | 1.38 | -2 | AGPAT3,LPIN1,LPIN3,PLPP5 |
| Apelin Cardiomyocyte Signaling Pathway | 1.35 | -1.633 | MAPK14,MAPK3,MAPK7,NOTUM,PRKD3,SLC9A8 |
| Mitochondrial Dysfunction | 1.34 | NA | GPD2,MT-ATP6,MT-CYB,MT-ND2,MT-ND3,MT-ND4,MT-ND4L,MT-ND5,MT-ND6 |

**Table 1.** Significant pathways for differentially expressed transcripts in ^56^Fe vs. non-irradiated control at 1 month analyzed by IPA.

| **Ingenuity Canonical Pathways** | **-log(p-value)** | **z-score** | **Molecules** |
| --- | --- | --- | --- |
| April Mediated Signaling | 4.51 | -0.378 | CHUK,IKBKB,IKBKG,MAPK14,MAPK9,NFAT5,NFATC1 |
| B Cell Activating Factor Signaling | 4.36 | -0.378 | CHUK,IKBKB,IKBKG,MAPK14,MAPK9,NFAT5,NFATC1 |
| 4-1BB Signaling in T Lymphocytes | 4.05 | 0 | ATF2,CHUK,IKBKB,IKBKG,MAPK14,MAPK9 |
| Production of Nitric Oxide and Reactive Oxygen Species in Macrophages | 3.66 | -1.387 | APOB,APOE,CAT,CHUK,CLU,IKBKB,IKBKG,Map3k7,MAPK14,MAPK9,PIK3CD,PTPA,RHOBTB1,SIRPA |
| LXR/RXR Activation | 3.64 | -0.707 | APOB,APOE,C4A/C4B,CLU,ECHS1,NCOR1,NCOR2,NR1H2,SAA1,TLR3,VTN |
| PTEN Signaling | 3.64 | 1.508 | CDKN1B,CHUK,FGFR2,FGFR3,GSK3A,IKBKB,IKBKG,MAGI1,MAST2,PIK3CD,SYNJ1 |
| RANK Signaling in Osteoclasts | 3.6 | -1 | CHUK,IKBKB,IKBKG,Map3k7,MAPK14,MAPK9,NFATC1,PIK3CD,XIAP |
| Role of PKR in Interferon Induction and Antiviral Response | 3.43 | NA | ATF2,CHUK,IKBKB,IKBKG,MAPK14,TLR3 |
| IL-1 Signaling | 3.42 | -0.378 | ADCY9,CHUK,GNB2,IKBKB,IKBKG,IRAK1,MAPK14,MAPK9,TOLLIP |
| FXR/RXR Activation | 3.38 | NA | APOB,APOE,BAAT,C4A/C4B,CLU,FETUB,LIPC,MAPK9,SAA1,SDC1,VTN |
| PI3K/AKT Signaling | 3.38 | -0.302 | CDKN1B,CHUK,CTNNB1,GSK3A,HSP90AB1,IKBKB,IKBKG,PIK3CD,PTPA,SYNJ1,TSC1 |
| Phosphatidylcholine Biosynthesis I | 3.24 | 1 | CHKA,CHPT1,PCYT1A,PHKA1 |
| B Cell Receptor Signaling | 3.22 | 0 | ATF2,CHUK,GSK3A,IKBKB,IKBKG,Map3k7,MAPK14,MAPK9,NFAT5,NFATC1,PIK3CD,SYNJ1,TCF3 |
| TNFR2 Signaling | 3.2 | -0.447 | CHUK,IKBKB,IKBKG,TBK1,XIAP |
| Activation of IRF by Cytosolic Pattern Recognition Receptors | 3.16 | 0.378 | ADAR,ATF2,CHUK,IKBKB,IKBKG,MAPK9,TBK1 |
| Huntington's Disease Signaling | 3.15 | 0 | ATF2,ATP5F1B,ATP5F1C,ATP5PB,DNM2,GNB2,HDAC5,MAPK9,NCOR1,NCOR2,PIK3CD,PLCB4,PSME1,RASA1,SGK1 |
| Protein Kinase A Signaling | 3.08 | -1.941 | ADCY9,AKAP1,AKAP12,AKAP8,ANAPC5,ATF2,CHUK,CTNNB1,EYA3,FLNA,GNB2,GSK3A,NFAT5,NFATC1,PHKA2,PLCB4,PTPN21,PTPRJ,SIRPA,TCF3,TTN |
| Epithelial Adherens Junction Signaling | 2.94 | NA | ACTR3,BAIAP2,CLIP1,CTNNB1,KEAP1,MAGI1,MET,MYH14,PARD3,RAPGEF1,TCF3 |
| Adipogenesis pathway | 2.86 | NA | CTNNB1,FGFR2,FGFR3,HDAC5,KAT2A,KAT6A,RPS6KA1,SAP130,SENP2,SMAD5 |
| Neuroinflammation Signaling Pathway | 2.85 | -0.728 | ATF2,BIRC6,CHUK,CTNNB1,IKBKB,IKBKG,IRAK1,MAPK14,MAPK6,MAPK9,NFAT5,NFATC1,NOX4,PIK3CD,TBK1,TLR3,XIAP |
| Antioxidant Action of Vitamin C | 2.84 | 0.378 | ABHD3,CHUK,GLRX,IKBKB,IKBKG,MAPK14,MAPK9,PLCB4,TXNRD2 |
| Xenobiotic Metabolism Signaling | 2.81 | NA | ABCC3,CAT,ESD,FMO2,HDAC5,HSP90AB1,KEAP1,Map3k7,MAPK14,MAPK9,NCOR2,NDST1,NRIP1,PIK3CD,PTGES3,PTPA |
| Endocannabinoid Developing Neuron Pathway | 2.76 | 0.333 | ADCY9,ATF2,CDKN1B,CTNNB1,MAPK14,MAPK6,MAPK9,MGLL,PIK3CD |
| Sirtuin Signaling Pathway | 2.71 | 0.832 | ACLY,ATP5F1B,ATP5F1C,ATP5PB,GABARAPL1,GLUD1,KAT2A,MAPK6,MYCN,NDUFA10,NR1H2,PFKFB3,PFKM,PGK1,POLR3D,SCNN1A,STK11 |
| Toll-like Receptor Signaling | 2.65 | -0.816 | CHUK,IKBKB,IKBKG,IRAK1,MAPK14,TLR3,TOLLIP |
| Cdc42 Signaling | 2.64 | 0.707 | ACTR3,ATF2,BAIAP2,CLIP1,DIAPH1,H2-T22,MAPK14,MAPK9,PARD3,RASA1,TNK2 |
| PPARα/RXRα Activation | 2.62 | -1.667 | ACOX1,ADCY9,CHUK,CKAP5,HSP90AB1,IKBKB,IKBKG,MAPK14,MED24,NCOR1,NCOR2,PLCB4 |
| Clathrin-mediated Endocytosis Signaling | 2.61 | NA | ACTR3,AP1B1,AP2A1,APOB,APOE,CLU,DNM2,MET,MYO6,PIK3CD,SH3GLB2,SYNJ1 |
| Assembly of RNA Polymerase III Complex | 2.5 | NA | GTF3A,GTF3C4,POLR3D |
| LPS-stimulated MAPK Signaling | 2.49 | -0.378 | ATF2,CHUK,IKBKB,IKBKG,MAPK14,MAPK9,PIK3CD |
| Apelin Cardiomyocyte Signaling Pathway | 2.49 | -1.414 | ATP2A3,CAT,MAPK14,MAPK6,MAPK9,PIK3CD,PLCB4,SLC9A8 |
| Glucocorticoid Receptor Signaling | 2.48 | NA | CHUK,HSP90AB1,IKBKB,IKBKG,MAPK14,MAPK9,NCOR1,NCOR2,NFAT5,NFATC1,NRIP1,PBRM1,PIK3CD,PTGES3,SGK1,TAF1,TAT |
| NF-κB Signaling | 2.44 | -1.508 | AZI2,CHUK,FGFR2,FGFR3,IKBKB,IKBKG,IRAK1,PIK3CD,TBK1,TLR3,TNIP1 |
| Role of RIG1-like Receptors in Antiviral Innate Immunity | 2.44 | -1.342 | CHUK,IKBKB,IKBKG,TBK1,TRIM25 |
| Acute Phase Response Signaling | 2.41 | -1.667 | C4A/C4B,CHUK,FN1,IKBKB,IKBKG,IRAK1,MAPK14,MAPK9,PIK3CD,SAA1,TCF3 |
| CD40 Signaling | 2.37 | -1.633 | CHUK,IKBKB,IKBKG,MAPK14,MAPK9,PIK3CD |
| IL-17A Signaling in Airway Cells | 2.34 | -1.633 | CHUK,IKBKB,IKBKG,MAPK14,MAPK9,PIK3CD |
| IL-12 Signaling and Production in Macrophages | 2.3 | NA | APOB,APOE,CHUK,CLU,IKBKB,IKBKG,MAPK14,MAPK9,PIK3CD |
| iNOS Signaling | 2.27 | -1.342 | CHUK,IKBKB,IKBKG,IRAK1,MAPK14 |
| Remodeling of Epithelial Adherens Junctions | 2.24 | -1 | ACTR3,CBLL1,CLIP1,CTNNB1,DNM2,MET |
| TNFR1 Signaling | 2.2 | 0.447 | CHUK,IKBKB,IKBKG,MAP4K2,XIAP |
| PI3K Signaling in B Lymphocytes | 2.16 | -0.707 | ATF2,CD81,CHUK,IKBKB,IKBKG,NFAT5,NFATC1,PIK3CD,PLCB4 |
| NGF Signaling | 2.12 | 0.707 | ATF2,CHUK,IKBKB,IKBKG,Map3k7,MAPK9,PIK3CD,RPS6KA1 |
| Role of Osteoblasts, Osteoclasts and Chondrocytes in Rheumatoid Arthritis | 2.12 | NA | CHUK,CTNNB1,IKBKB,IKBKG,MAPK14,MAPK9,NFAT5,NFATC1,PIK3CD,SMAD5,TCF3,XIAP |
| Cardiac Hypertrophy Signaling (Enhanced) | 2.1 | -0.655 | ADCY9,ATF2,ATP2A3,CHUK,CTNNB1,DIAPH1,DLG1,FGFR2,FGFR3,GSK3A,HDAC5,IKBKB,IKBKG,Map3k7,MAPK14,MAPK9,NFAT5,NFATC1,PIK3CD,PKN1,PLCB4 |
| ATM Signaling | 2.09 | 0.447 | ATF2,BRAT1,MAPK14,MAPK9,PTPA,SMC2,TRRAP |
| CD27 Signaling in Lymphocytes | 2.09 | 0.447 | CHUK,IKBKB,IKBKG,Map3k7,MAPK9 |
| RAR Activation | 2.07 | NA | ADCY9,MAPK14,MAPK9,NCOR1,NCOR2,NRIP1,PBRM1,PIK3CD,SMAD5,TNIP1,TRIM24 |
| LPS/IL-1 Mediated Inhibition of RXR Function | 2.07 | 0.378 | ABCC3,ACOX1,ACSL4,ALAS1,APOE,CAT,FMO2,IRAK1,LIPC,MAPK9,NDST1,NR1H2 |
| Pyridoxal 5'-phosphate Salvage Pathway | 2.07 | 0 | GRK6,IRAK1,MAPK6,MAPK9,PKN1,SGK1 |
| Role of Macrophages, Fibroblasts and Endothelial Cells in Rheumatoid Arthritis | 2.05 | NA | ATF2,CHUK,CTNNB1,FN1,IKBKB,IKBKG,IRAK1,MAPK14,MAPK9,NFAT5,NFATC1,PIK3CD,PLCB4,TCF3,TLR3 |
| TWEAK Signaling | 2.05 | -1 | CHUK,IKBKB,IKBKG,XIAP |
| IL-17A Signaling in Fibroblasts | 2.05 | NA | CHUK,IKBKB,IKBKG,MAPK14 |
| CD28 Signaling in T Helper Cells | 2.02 | -0.707 | ACTR3,CHUK,IKBKB,IKBKG,MAPK9,NFAT5,NFATC1,PIK3CD |
| Angiopoietin Signaling | 2.02 | 0.447 | CHUK,IKBKB,IKBKG,PIK3CD,RASA1,TNIP1 |
| Role of IL-17A in Arthritis | 1.99 | NA | ATF2,MAPK14,MAPK9,PIK3CD,RPS6KA1 |
| Chronic Myeloid Leukemia Signaling | 1.95 | NA | CDKN1B,CHUK,HDAC5,IKBKB,IKBKG,PIK3CD,RBL2 |
| 14-3-3-mediated Signaling | 1.94 | -0.707 | CDKN1B,GSK3A,MAPK9,PIK3CD,PLCB4,RPS6KA1,STK11,TSC1 |
| PPAR Signaling | 1.94 | -0.378 | CHUK,HSP90AB1,IKBKB,IKBKG,NCOR1,NCOR2,NRIP1 |
| IL-7 Signaling Pathway | 1.94 | -2.449 | CDKN1B,GSK3A,MAPK14,MET,NFATC1,PIK3CD |
| Apelin Adipocyte Signaling Pathway | 1.91 | 0.816 | ADCY9,CAT,MAPK14,MAPK6,MAPK9,NOX4 |
| Inhibition of Angiogenesis by TSP1 | 1.88 | NA | CD47,MAPK14,MAPK9,SDC1 |
| Role of NFAT in Regulation of the Immune Response | 1.87 | -1 | ATF2,CHUK,GNB2,GSK3A,IKBKB,IKBKG,NFAT5,NFATC1,PIK3CD,PLCB4 |
| Signaling by Rho Family GTPases | 1.86 | -0.707 | ACTR3,ARHGEF12,BAIAP2,CLIP1,GNB2,MAPK9,NOX4,PARD3,PIK3CD,PKN1,RDX,RHOBTB1 |
| Mitochondrial Dysfunction | 1.85 | NA | ATP5F1B,ATP5F1C,ATP5MC2,ATP5PB,CAT,CYB5A,FURIN,MAPK9,NDUFA10,TXNRD2 |
| Induction of Apoptosis by HIV1 | 1.84 | -0.447 | CHUK,IKBKB,IKBKG,MAPK9,XIAP |
| 3-phosphoinositide Degradation | 1.83 | -0.333 | MTM1,PPP1R16B,PPP4R1,PTPA,PTPRJ,RASA1,SET,SIRPA,SYNJ1 |
| NRF2-mediated Oxidative Stress Response | 1.82 | 0.378 | CAT,CDC34,DNAJA3,DNAJB11,GCLM,KEAP1,MAPK14,MAPK9,PIK3CD,SQSTM1 |
| D-myo-inositol-5-phosphate Metabolism | 1.81 | -1 | PLCB4,PPP1R16B,PPP4R1,PTPA,PTPRJ,RASA1,SET,SIRPA,SYNJ1 |
| T Cell Receptor Signaling | 1.81 | NA | CHUK,IKBKB,IKBKG,NFAT5,NFATC1,PIK3CD,RASA1 |
| ILK Signaling | 1.78 | 0.707 | ATF2,CTNNB1,FLNA,FN1,GSK3A,MAPK9,MYH14,PIK3CD,PTPA,RHOBTB1 |
| Glycolysis I | 1.77 | 1 | ENO3,GPI,PFKM,PGK1 |
| Superpathway of Inositol Phosphate Compounds | 1.76 | -1.508 | PIK3CD,PLCB4,PPP1R16B,PPP4R1,PTPA,PTPRJ,RASA1,SEC16A,SET,SIRPA,SYNJ1 |
| FGF Signaling | 1.75 | -1.633 | ATF2,FGFR2,FGFR3,MAPK14,MET,PIK3CD |
| Choline Biosynthesis III | 1.74 | NA | CHPT1,PCYT1A,PHKA1 |
| Salvage Pathways of Pyrimidine Ribonucleotides | 1.73 | -0.378 | AK4,GRK6,IRAK1,MAPK6,MAPK9,PKN1,SGK1 |
| Regulation of IL-2 Expression in Activated and Anergic T Lymphocytes | 1.68 | NA | CHUK,IKBKB,IKBKG,MAPK9,NFAT5,NFATC1 |
| Fatty Acid β-oxidation I | 1.67 | 0 | ACSL4,ECHS1,HADHB,SCP2 |
| Insulin Receptor Signaling | 1.66 | -0.378 | ACLY,GSK3A,PIK3CD,RAPGEF1,SCNN1A,SGK1,SYNJ1,TSC1 |
| IL-8 Signaling | 1.64 | -1.414 | CHUK,GNB2,IKBKB,IKBKG,IRAK1,ITGAV,MAPK9,NOX4,PIK3CD,RHOBTB1 |
| Antiproliferative Role of TOB in T Cell Signaling | 1.64 | NA | CDC34,CDKN1B,RPS6KA1 |
| Factors Promoting Cardiogenesis in Vertebrates | 1.64 | NA | ATF2,CTNNB1,MAPK14,NOX4,SMAD5,TCF3 |
| FAT10 Cancer Signaling Pathway | 1.64 | 0 | CHUK,CTNNB1,IKBKB,IKBKG |
| D-myo-inositol (1,4,5,6)-Tetrakisphosphate Biosynthesis | 1.61 | -0.707 | PPP1R16B,PPP4R1,PTPA,PTPRJ,RASA1,SET,SIRPA,SYNJ1 |
| D-myo-inositol (3,4,5,6)-tetrakisphosphate Biosynthesis | 1.61 | -0.707 | PPP1R16B,PPP4R1,PTPA,PTPRJ,RASA1,SET,SIRPA,SYNJ1 |
| Prostate Cancer Signaling | 1.6 | NA | ATF2,CDKN1B,CHUK,CTNNB1,HSP90AB1,PIK3CD |
| Aryl Hydrocarbon Receptor Signaling | 1.6 | 0.707 | CDKN1B,HSP90AB1,NCOR2,NFIA,NFIX,NRIP1,PTGES3,RBL2 |
| 3-phosphoinositide Biosynthesis | 1.59 | -1 | PIK3CD,PPP1R16B,PPP4R1,PTPA,PTPRJ,RASA1,SET,SIRPA,SYNJ1 |
| Phenylalanine Degradation I (Aerobic) | 1.58 | NA | PAH,QDPR |
| fMLP Signaling in Neutrophils | 1.57 | -1.633 | ACTR3,GNB2,NFAT5,NFATC1,NOX4,PIK3CD,PLCB4 |
| RhoA Signaling | 1.54 | -0.378 | ACTR3,ARHGAP1,ARHGEF12,BAIAP2,PKN1,RDX,TTN |
| Molecular Mechanisms of Cancer | 1.54 | NA | ADCY9,ARHGEF12,CDK13,CDKN1B,CTNNB1,GSK3A,MAPK14,MAPK9,PIK3CD,PLCB4,RAPGEF1,RASA1,RHOBTB1,SMAD5,TCF3,XIAP |
| Apoptosis Signaling | 1.53 | 0.816 | BIRC6,CHUK,IKBKB,IKBKG,RPS6KA1,XIAP |
| Role of p14/p19ARF in Tumor Suppression | 1.52 | NA | PIK3CD,POLR3D,UBTF |
| Small Cell Lung Cancer Signaling | 1.52 | -1.342 | CDKN1B,CHUK,IKBKB,IKBKG,PIK3CD |
| Hypoxia Signaling in the Cardiovascular System | 1.52 | NA | ATF2,BIRC6,CDC34,COPS5,HSP90AB1 |
| Dendritic Cell Maturation | 1.51 | -0.333 | ATF2,CHUK,IKBKB,IKBKG,MAPK14,MAPK9,PIK3CD,PLCB4,TLR3 |
| Mouse Embryonic Stem Cell Pluripotency | 1.45 | -0.816 | CTNNB1,MAPK14,PIK3CD,SMAD5,TCF3,XIAP |
| SAPK/JNK Signaling | 1.45 | 0.447 | ATF2,MAP4K2,MAPK8IP3,MAPK9,NFATC1,PIK3CD |
| Gα12/13 Signaling | 1.44 | 0.378 | CHUK,CTNNB1,IKBKB,IKBKG,MAPK9,PIK3CD,RASA1 |
| BER pathway | 1.44 | NA | LIG3,PNKP |
| Reelin Signaling in Neurons | 1.43 | NA | APOE,ARHGEF12,MAPK8IP3,MAPK9,PIK3CD |
| Transcriptional Regulatory Network in Embryonic Stem Cells | 1.42 | NA | CDYL,KAT6A,SET,TRIM24 |
| Actin Cytoskeleton Signaling | 1.4 | -0.707 | ACTR3,ARHGEF12,BAIAP2,DIAPH1,FLNA,FN1,MYH14,PIK3CD,RDX,TTN |
| Lymphotoxin β Receptor Signaling | 1.39 | -1 | CHUK,IKBKB,IKBKG,PIK3CD |
| Sertoli Cell-Sertoli Cell Junction Signaling | 1.39 | NA | ATF2,CTNNB1,DLG1,EPB41,GSK3A,KEAP1,Map3k7,MAPK14,MAPK9 |
| Gαq Signaling | 1.37 | -1.633 | CHUK,GNB2,IKBKB,IKBKG,NFATC1,PIK3CD,PLCB4,RHOBTB1 |
| Estrogen Receptor Signaling | 1.36 | NA | MED15,MED24,NCOR1,NCOR2,NRIP1,TAF1,TRRAP |
| PEDF Signaling | 1.35 | -1.342 | CHUK,IKBKB,IKBKG,MAPK14,PIK3CD |
| DNA Methylation and Transcriptional Repression Signaling | 1.34 | NA | ARID4B,MTA1,SAP130 |
| AMPK Signaling | 1.34 | 0 | AK4,ATF2,MAPK14,PBRM1,PFKFB3,PFKM,PIK3CD,PTPA,STK11,TSC1 |
| NF-κB Activation by Viruses | 1.33 | -1.342 | CHUK,IKBKB,IKBKG,ITGAV,PIK3CD |
| Human Embryonic Stem Cell Pluripotency | 1.32 | NA | CTNNB1,FGFR2,FGFR3,GSK3A,PIK3CD,SMAD5,TCF3 |
| HER-2 Signaling in Breast Cancer | 1.31 | NA | CDKN1B,GSK3A,PARD3,PIK3CD,TSC1 |
| Valine Degradation I | 1.31 | NA | BCKDHB,ECHS1,HADHB |
| Type I Diabetes Mellitus Signaling | 1.31 | -0.816 | CHUK,IKBKB,IKBKG,IRAK1,MAPK14,MAPK9 |

**Table 2.** Significant pathways for differentially expressed transcripts in ^56^Fe vs. non-irradiated control at 2 months analyzed by IPA.

| **Ingenuity Canonical Pathways** | **-log(p-value)** | **z-score** | **Molecules** |
| --- | --- | --- | --- |
| B Cell Receptor Signaling | 6.04 | -1.213 | AKT3,ATF2,BCL6,Calm1 (includes others),CREB1,EGR1,FCGR2A,IKBKG,INPP5B,MAP3K13,MAPK14,MAPK3,NFAT5,PIK3C2G,PIK3CA,SYK,SYNJ1,SYNJ2 |
| Small Cell Lung Cancer Signaling | 5.8 | -0.816 | AKT3,BIRC2,IKBKG,PA2G4,PIAS3,PIK3C2G,PIK3CA,RXRA,RXRB,SIN3A,TRAF3 |
| Role of Tissue Factor in Cancer | 5.08 | NA | AKT3,ARRB1,EGFR,EGR1,ITGA3,ITGAV,JAK2,MAPK14,MAPK3,PIK3C2G,PIK3CA,STAT5A,STAT5B |
| LXR/RXR Activation | 4.85 | -1.508 | APOE,C4A/C4B,CYP7A1,HMGCR,NR1H2,NR1H3,NR1H4,RXRA,RXRB,SAA1,SCD,SERPINF1,TLR3 |
| PTEN Signaling | 4.85 | 0.277 | AKT3,EGFR,FGFR3,IKBKG,INPP5B,ITGA3,MAGI1,MAPK3,MAST2,PIK3CA,SYNJ1,SYNJ2,TGFBR1 |
| Chronic Myeloid Leukemia Signaling | 4.4 | NA | AKT3,IKBKG,MAPK3,MDM2,PA2G4,PIK3C2G,PIK3CA,SIN3A,STAT5A,STAT5B,TGFBR1 |
| IL-15 Signaling | 4.24 | NA | AKT3,JAK2,MAPK14,MAPK3,PIK3C2G,PIK3CA,STAT5A,STAT5B,SYK |
| TR/RXR Activation | 4.16 | NA | AKT3,CYP7A1,G6PC,HP,MDM2,PIK3C2G,PIK3CA,RXRA,RXRB,THRA |
| Regulation of the Epithelial-Mesenchymal Transition Pathway | 4.12 | NA | ADAM17,AKT3,APC,DVL2,EGFR,EGR1,FGF21,FGFR3,FZD6,JAK2,MAPK3,PIK3C2G,PIK3CA,TGFBR1,ZEB2 |
| Pancreatic Adenocarcinoma Signaling | 4.1 | -0.707 | AKT3,EGFR,JAK2,MAPK3,MDM2,PA2G4,PIK3C2G,PIK3CA,RALBP1,SIN3A,TGFBR1 |
| Role of Macrophages, Fibroblasts and Endothelial Cells in Rheumatoid Arthritis | 3.98 | NA | AKT3,APC,ATF2,C5AR1,Calm1 (includes others),CEBPA,CREB1,CSNK1A1,DAAM1,FN1,FZD6,IKBKG,JAK2,MAPK14,MAPK3,NFAT5,PIK3C2G,PIK3CA,TLR3,TRAF3 |
| Estrogen-Dependent Breast Cancer Signaling | 3.96 | -1 | AKT3,ATF2,CREB1,EGFR,MAPK3,PIK3C2G,PIK3CA,STAT5A,STAT5B |
| Non-Small Cell Lung Cancer Signaling | 3.92 | -1.342 | AKT3,EGFR,MAPK3,PA2G4,PIK3C2G,PIK3CA,RXRA,RXRB,SIN3A |
| FAK Signaling | 3.88 | NA | AKT3,ASAP1,CAPNS1,EGFR,ITGA3,MAPK3,PIK3C2G,PIK3CA,PXN,TNS1 |
| CD40 Signaling | 3.78 | -2.121 | IKBKG,MAPK14,MAPK3,PIK3C2G,PIK3CA,TANK,TNFAIP3,TRAF3 |
| FLT3 Signaling in Hematopoietic Progenitor Cells | 3.75 | -1 | AKT3,ATF2,CREB1,MAPK14,MAPK3,PIK3C2G,PIK3CA,STAT5A,STAT5B |
| Colorectal Cancer Metastasis Signaling | 3.71 | 0.277 | ADCY6,AKT3,APC,ARRB1,EGFR,FZD6,GNB1,GNB2,GRK2,JAK2,MAPK3,PIK3C2G,PIK3CA,RHOBTB1,RHOT1,TGFBR1,TLR3 |
| IL-22 Signaling | 3.63 | -0.447 | AKT3,MAPK14,MAPK3,STAT5A,STAT5B |
| SPINK1 General Cancer Pathway | 3.6 | 0 | AKT3,EGFR,JAK2,MAPK3,Mt1,Mt2,PIK3C2G,PIK3CA |
| FGF Signaling | 3.56 | -1.667 | AKT3,ATF2,CREB1,FGF21,FGFR3,MAPK14,MAPK3,PIK3C2G,PIK3CA |
| Role of JAK family kinases in IL-6-type Cytokine Signaling | 3.54 | NA | JAK2,MAPK14,MAPK3,STAT5A,STAT5B |
| RANK Signaling in Osteoclasts | 3.52 | -1 | AKT3,BIRC2,Calm1 (includes others),IKBKG,MAP3K13,MAPK14,MAPK3,PIK3C2G,PIK3CA |
| Lymphotoxin β Receptor Signaling | 3.46 | -0.816 | AKT3,BIRC2,IKBKG,MAPK3,PIK3C2G,PIK3CA,TRAF3 |
| Growth Hormone Signaling | 3.35 | 1.134 | CEBPA,JAK2,MAPK3,ONECUT1,PIK3C2G,PIK3CA,STAT5A,STAT5B |
| Prostate Cancer Signaling | 3.31 | NA | AKT3,ATF2,CREB1,MAPK3,MDM2,PA2G4,PIK3C2G,PIK3CA,SIN3A |
| FXR/RXR Activation | 3.29 | NA | AKT3,APOE,C4A/C4B,CYP7A1,FETUB,G6PC,NR1H3,NR1H4,RXRA,SAA1,SERPINF1 |
| p53 Signaling | 3.21 | 0.816 | AKT3,ATR,COQ8A,GADD45G,MAPK14,MDM2,PIK3C2G,PIK3CA,PML |
| Role of NFAT in Regulation of the Immune Response | 3.19 | -0.905 | AKT3,ATF2,Calm1 (includes others),CSNK1A1,FCGR2A,GNB1,GNB2,IKBKG,MAPK3,NFAT5,PIK3C2G,PIK3CA,SYK |
| JAK/Stat Signaling | 3.16 | 0 | AKT3,JAK2,MAPK3,PIAS3,PIK3C2G,PIK3CA,STAT5A,STAT5B |
| IL-7 Signaling Pathway | 3.16 | -0.378 | AKT3,BCL6,MAPK14,MAPK3,PIK3C2G,PIK3CA,STAT5A,STAT5B |
| TNFR2 Signaling | 3.16 | 1 | BIRC2,IKBKG,TANK,TBK1,TNFAIP3 |
| Neuroinflammation Signaling Pathway | 3.15 | -1.414 | ACVR2B,AKT3,APP,ATF2,BIRC2,CREB1,IKBKG,JAK2,MAPK14,MAPK3,NFAT5,PIK3C2G,PIK3CA,SYK,TBK1,TGFBR1,TLR3,TRAF3 |
| Neuregulin Signaling | 3.12 | 0 | ADAM17,AKT3,EGFR,ERBB4,ERRFI1,ITGA3,MAPK3,STAT5A,STAT5B |
| IL-2 Signaling | 3.1 | -0.378 | AKT3,MAPK3,PIK3C2G,PIK3CA,STAT5A,STAT5B,SYK |
| PEDF Signaling | 3.09 | -0.707 | AKT3,IKBKG,MAPK14,MAPK3,PIK3C2G,PIK3CA,SERPINF1,TCF12 |
| Mouse Embryonic Stem Cell Pluripotency | 3.05 | -1.667 | AKT3,APC,DVL2,FZD6,JAK2,MAPK14,MAPK3,PIK3C2G,PIK3CA |
| Production of Nitric Oxide and Reactive Oxygen Species in Macrophages | 3.04 | 0 | AKT3,APOE,IKBKG,JAK2,MAP3K13,MAPK14,MAPK3,PIK3C2G,PIK3CA,PPP1R3C,RHOBTB1,RHOT1,SIRPA |
| Phagosome Formation | 3.03 | NA | C5AR1,FCGR2A,FN1,ITGA3,PIK3C2G,PIK3CA,RHOBTB1,RHOT1,SYK,TLR3 |
| IL-17A Signaling in Airway Cells | 2.98 | -1.134 | AKT3,IKBKG,JAK2,MAPK14,MAPK3,PIK3C2G,PIK3CA |
| Cardiac Hypertrophy Signaling (Enhanced) | 2.94 | 0 | ACVR2B,ADCY6,AKT3,ATF2,Calm1 (includes others),DVL2,FGF21,FGFR3,FZD6,GNB1,IKBKG,IL17RB,ITGA3,JAK2,MAP3K13,MAPK14,MAPK3,NFAT5,PDE1A,PDE4B,PDK1,PIK3C2G,PIK3CA,TGFBR1 |
| Germ Cell-Sertoli Cell Junction Signaling | 2.93 | NA | EPN2,ITGA3,KEAP1,MAP3K13,MAPK14,MAPK3,PIK3C2G,PIK3CA,PXN,RHOBTB1,RHOT1,TGFBR1 |
| IL-4 Signaling | 2.92 | NA | AKT3,INPP5B,JAK2,NFAT5,PIK3C2G,PIK3CA,SYNJ1,SYNJ2 |
| Protein Ubiquitination Pathway | 2.91 | NA | ANAPC5,BIRC2,DNAJB2,DNAJC12,HSPH1,MDM2,NEDD4L,PAN2,UBE4A,USP19,USP2,USP21,USP34,USP36,USP45,USP8 |
| Role of JAK2 in Hormone-like Cytokine Signaling | 2.9 | NA | JAK2,SH2B1,SIRPA,STAT5A,STAT5B |
| Role of Osteoblasts, Osteoclasts and Chondrocytes in Rheumatoid Arthritis | 2.9 | NA | ADAM17,AKT3,APC,BIRC2,Calm1 (includes others),CSNK1A1,FZD6,IKBKG,ITGA3,MAPK14,MAPK3,NFAT5,PIK3C2G,PIK3CA |
| Role of JAK1 and JAK3 in γc Cytokine Signaling | 2.86 | NA | JAK2,MAPK3,PIK3C2G,PIK3CA,STAT5A,STAT5B,SYK |
| Epithelial Adherens Junction Signaling | 2.85 | NA | ACVR2B,AKT3,APC,BAIAP2,EGFR,EPN2,KEAP1,MAGI1,MYH11,PARD3,TGFBR1 |
| NF-κB Signaling | 2.85 | -0.905 | AKT3,EGFR,FGFR3,IKBKG,PIK3C2G,PIK3CA,TANK,TBK1,TGFBR1,TLR3,TNFAIP3,TRAF3 |
| PXR/RXR Activation | 2.83 | NA | AKT3,ALAS1,CES3,CYP7A1,G6PC,RXRA,SCD |
| Amyloid Processing | 2.81 | -1 | AKT3,APP,CAPNS1,CSNK1A1,MAPK14,MAPK3 |
| Acute Phase Response Signaling | 2.81 | -1.414 | AKT3,C4A/C4B,FN1,HP,IKBKG,JAK2,MAPK14,MAPK3,PIK3CA,SAA1,Saa3,SERPINF1 |
| Ephrin Receptor Signaling | 2.77 | -0.302 | ABI1,AKT3,ATF2,CREB1,EPHB4,GNB1,GNB2,ITGA3,JAK2,MAPK3,PIK3C2G,PXN |
| Relaxin Signaling | 2.76 | 0 | ADCY6,AKT3,CREB1,GNB1,GNB2,MAPK3,NPR2,PDE1A,PDE4B,PIK3C2G,PIK3CA |
| Acute Myeloid Leukemia Signaling | 2.76 | -0.707 | AKT3,CEBPA,MAPK3,PIK3C2G,PIK3CA,PML,STAT5A,STAT5B |
| PI3K/AKT Signaling | 2.73 | 0 | AKT3,IKBKG,INPP5B,ITGA3,JAK2,MAPK3,MDM2,PIK3CA,SYNJ1,SYNJ2 |
| Endocannabinoid Developing Neuron Pathway | 2.69 | -1.667 | ADCY6,AKT3,ATF2,CREB1,GNB1,MAPK14,MAPK3,PIK3C2G,PIK3CA |
| Glioma Signaling | 2.64 | -1.633 | AKT3,Calm1 (includes others),EGFR,MAPK3,MDM2,PA2G4,PIK3C2G,PIK3CA,SIN3A |
| Ovarian Cancer Signaling | 2.64 | -0.816 | AKT3,APC,ARRB1,EGFR,FZD6,MAPK3,PA2G4,PIK3C2G,PIK3CA,SIN3A |
| Gαq Signaling | 2.64 | -0.333 | AKT3,Calm1 (includes others),GNB1,GNB2,GRK2,IKBKG,MAPK3,PIK3C2G,PIK3CA,RHOBTB1,RHOT1 |
| Integrin Signaling | 2.64 | 0.302 | AKT3,ASAP1,CAPNS1,GIT1,ITGA3,ITGAV,MAPK3,PIK3C2G,PIK3CA,PXN,RHOBTB1,RHOT1,TNK2 |
| Huntington's Disease Signaling | 2.6 | 0 | AKT3,ATF2,ATP5F1C,CAPNS1,CREB1,EGFR,GNB1,GNB2,MAPK3,NAPA,PIK3C2G,PIK3CA,SIN3A,ZDHHC17 |
| EGF Signaling | 2.53 | -1.633 | AKT3,EGFR,MAPK14,MAPK3,PIK3C2G,PIK3CA |
| Role of PKR in Interferon Induction and Antiviral Response | 2.53 | NA | ATF2,IKBKG,MAPK14,TLR3,TRAF3 |
| Erythropoietin Signaling | 2.52 | NA | AKT3,JAK2,MAPK3,PIK3C2G,PIK3CA,STAT5A,STAT5B |
| IL-3 Signaling | 2.52 | 0.378 | AKT3,JAK2,MAPK3,PIK3C2G,PIK3CA,STAT5A,STAT5B |
| Fc Epsilon RI Signaling | 2.52 | -1.667 | AKT3,INPP5B,MAPK14,MAPK3,PIK3C2G,PIK3CA,SYK,SYNJ1,SYNJ2 |
| Cardiac Hypertrophy Signaling | 2.51 | 0 | ADCY6,ATF2,Calm1 (includes others),CREB1,GNB1,GNB2,MAP3K13,MAPK14,MAPK3,PIK3C2G,PIK3CA,RHOBTB1,RHOT1,TGFBR1 |
| Natural Killer Cell Signaling | 2.49 | NA | AKT3,FCGR2A,INPP5B,MAPK3,PIK3C2G,PIK3CA,SYK,SYNJ1,SYNJ2 |
| IL-17 Signaling | 2.49 | NA | AKT3,ATF2,JAK2,MAPK14,MAPK3,PIK3C2G,PIK3CA |
| Systemic Lupus Erythematosus In B Cell Signaling Pathway | 2.45 | -1.291 | AKT3,Calm1 (includes others),FCGR2A,INPP5B,JAK2,MAPK3,NFAT5,PIK3C2G,PIK3CA,SYK,SYNJ1,SYNJ2,TBK1,TLR3,TRAF3 |
| CXCR4 Signaling | 2.44 | 0.333 | ADCY6,AKT3,EGR1,GNB1,GNB2,MAPK3,PIK3C2G,PIK3CA,PXN,RHOBTB1,RHOT1 |
| LPS-stimulated MAPK Signaling | 2.43 | -1.134 | ATF2,CREB1,IKBKG,MAPK14,MAPK3,PIK3C2G,PIK3CA |
| NF-κB Activation by Viruses | 2.4 | -0.378 | AKT3,IKBKG,ITGA3,ITGAV,MAPK3,PIK3C2G,PIK3CA |
| Docosahexaenoic Acid (DHA) Signaling | 2.39 | NA | AKT3,APP,PIK3C2G,PIK3CA,SERPINF1 |
| Molecular Mechanisms of Cancer | 2.37 | NA | ADCY6,AKT3,APC,ATR,BIRC2,FZD6,ITGA3,JAK2,MAPK14,MAPK3,MDM2,PA2G4,PIK3C2G,PIK3CA,RALBP1,RHOBTB1,RHOT1,SIN3A,TGFBR1 |
| GNRH Signaling | 2.37 | 0 | ADCY6,ATF2,Calm1 (includes others),CREB1,EGFR,EGR1,GNB1,MAP3K13,MAPK14,MAPK3,PXN |
| Cancer Drug Resistance By Drug Efflux | 2.32 | NA | ABCC10,AKT3,MAPK3,MDM2,PDK1,PIK3CA |
| Dendritic Cell Maturation | 2.28 | -0.905 | AKT3,ATF2,CREB1,FCGR2A,IKBKG,JAK2,MAPK14,MAPK3,PIK3C2G,PIK3CA,TLR3 |
| Thrombopoietin Signaling | 2.25 | 0.816 | JAK2,MAPK3,PIK3C2G,PIK3CA,STAT5A,STAT5B |
| Thrombin Signaling | 2.22 | -1.667 | ADCY6,AKT3,CREB1,EGFR,GNB1,GNB2,MAPK14,MAPK3,PIK3C2G,PIK3CA,RHOBTB1,RHOT1 |
| P2Y Purigenic Receptor Signaling Pathway | 2.21 | -0.707 | ADCY6,AKT3,ATF2,CREB1,GNB1,GNB2,MAPK3,PIK3C2G,PIK3CA |
| HGF Signaling | 2.19 | 0 | AKT3,ATF2,ITGA3,MAP3K13,MAPK3,PIK3C2G,PIK3CA,PXN |
| ErbB4 Signaling | 2.16 | -0.447 | ADAM17,ERBB4,MAPK3,PIK3C2G,PIK3CA,YAP1 |
| PDGF Signaling | 2.16 | 0.378 | INPP5B,JAK2,MAPK3,PIK3C2G,PIK3CA,SYNJ1,SYNJ2 |
| TNFR1 Signaling | 2.15 | 0 | BIRC2,IKBKG,MADD,TANK,TNFAIP3 |
| 4-1BB Signaling in T Lymphocytes | 2.15 | -1 | ATF2,IKBKG,MAPK14,MAPK3 |
| GM-CSF Signaling | 2.13 | 0 | AKT3,JAK2,MAPK3,PIK3C2G,PIK3CA,STAT5B |
| Phosphatidylcholine Biosynthesis I | 2.13 | NA | CHKA,PCYT1A,PHKA1 |
| ILK Signaling | 2.13 | -0.632 | AKT3,ATF2,CREB1,FN1,MAPK3,MYH11,PIK3C2G,PIK3CA,PXN,RHOBTB1,RHOT1 |
| Tec Kinase Signaling | 2.12 | 1.89 | GNB1,GNB2,ITGA3,JAK2,PIK3C2G,PIK3CA,RHOBTB1,RHOT1,STAT5A,STAT5B |
| IL-9 Signaling | 2.1 | 1 | PIK3C2G,PIK3CA,STAT5A,STAT5B |
| PI3K Signaling in B Lymphocytes | 2.09 | -2.333 | AKT3,ATF2,Calm1 (includes others),CREB1,IKBKG,MAPK3,NFAT5,PIK3CA,SYK |
| White Adipose Tissue Browning Pathway | 2.09 | -1 | ADCY6,ATF2,CREB1,FGF21,FGFR3,MAPK14,RXRA,RXRB,THRA |
| Melanoma Signaling | 2.08 | -0.447 | AKT3,MAPK3,MDM2,PIK3C2G,PIK3CA |
| UVB-Induced MAPK Signaling | 2.08 | -1.342 | EGFR,MAPK14,MAPK3,PIK3C2G,PIK3CA |
| Actin Cytoskeleton Signaling | 2.08 | 0 | APC,BAIAP2,CYFIP1,FGF21,FN1,GIT1,ITGA3,MAPK3,MYH11,PIK3C2G,PIK3CA,PXN |
| Glioblastoma Multiforme Signaling | 2.07 | -0.333 | AKT3,APC,EGFR,FZD6,MAPK3,MDM2,PIK3C2G,PIK3CA,RHOBTB1,RHOT1 |
| NGF Signaling | 2.06 | -0.707 | AKT3,ATF2,CREB1,IKBKG,MAP3K13,MAPK3,PIK3C2G,PIK3CA |
| Insulin Receptor Signaling | 2.06 | -0.333 | AKT3,INPP5B,JAK2,MAPK3,PIK3C2G,PIK3CA,PPP1R3C,SYNJ1,SYNJ2 |
| Ephrin B Signaling | 2.05 | -1 | ABI1,EPHB4,GNB1,GNB2,MAPK3,PXN |
| ATM Signaling | 2.04 | -0.447 | ATF2,ATR,CREB1,GADD45G,MAPK14,MDM2,TRRAP |
| Bladder Cancer Signaling | 2.04 | NA | EGFR,FGF21,FGFR3,MAPK3,MDM2,PA2G4,SIN3A |
| Glioma Invasiveness Signaling | 2.02 | -0.447 | ITGAV,MAPK3,PIK3C2G,PIK3CA,RHOBTB1,RHOT1 |
| Circadian Rhythm Signaling | 2.01 | NA | ATF2,CREB1,NR1D1,PER3 |
| IL-17A Signaling in Fibroblasts | 2.01 | NA | IKBKG,LCN2,MAPK14,MAPK3 |
| RAR Activation | 2 | NA | ADCY6,AKT3,JAK2,MAPK14,PIK3CA,PML,PRMT1,RXRA,RXRB,STAT5A,STAT5B |
| TREM1 Signaling | 1.99 | 0 | AKT3,JAK2,MAPK3,STAT5A,STAT5B,TLR3 |
| IL-8 Signaling | 1.97 | -0.333 | AKT3,EGFR,GNB1,GNB2,IKBKG,ITGAV,MAPK3,PIK3C2G,PIK3CA,RHOBTB1,RHOT1 |
| Angiopoietin Signaling | 1.97 | 0 | AKT3,IKBKG,PIK3C2G,PIK3CA,STAT5A,STAT5B |
| Opioid Signaling Pathway | 1.96 | -1.941 | ADCY6,AKT3,ARRB1,ATF2,Calm1 (includes others),CREB1,GNB1,GRK2,GRK6,MAPK3,PDE1A,PDK1,PIK3C2G |
| Role of IL-17A in Arthritis | 1.95 | NA | ATF2,MAPK14,MAPK3,PIK3C2G,PIK3CA |
| Leptin Signaling in Obesity | 1.94 | -0.447 | ADCY6,AKT3,JAK2,MAPK3,PIK3C2G,PIK3CA |
| Protein Kinase A Signaling | 1.93 | -0.5 | ADCY6,ANAPC5,ATF2,Calm1 (includes others),CREB1,GNB1,GNB2,MAPK3,MTMR3,NFAT5,PDE1A,PDE4B,PPP1R3C,PTPN21,PTPRG,PXN,SIRPA,TGFBR1 |
| T Cell Exhaustion Signaling Pathway | 1.92 | 0.333 | ACVR2B,AKT3,BCL6,JAK2,MAPK3,NFAT5,PDK1,PIK3C2G,PIK3CA,TGFBR1 |
| Phospholipase C Signaling | 1.89 | -0.632 | ADCY6,ATF2,Calm1 (includes others),CREB1,FCGR2A,GNB1,GNB2,ITGA3,MAPK3,NFAT5,RHOBTB1,RHOT1,SYK |
| Antiproliferative Role of Somatostatin Receptor 2 | 1.86 | 0 | GNB1,GNB2,MAPK3,NPR2,PIK3C2G,PIK3CA |
| April Mediated Signaling | 1.85 | -1 | IKBKG,MAPK14,NFAT5,TRAF3 |
| PD-1, PD-L1 cancer immunotherapy pathway | 1.82 | -0.378 | AKT3,JAK2,PIK3C2G,PIK3CA,STAT5A,STAT5B,YAP1 |
| D-myo-inositol (1,4,5)-trisphosphate Degradation | 1.81 | NA | INPP5B,SYNJ1,SYNJ2 |
| PCP pathway | 1.8 | -2.236 | ATF2,DAAM1,DVL2,FZD6,LGR4 |
| Prolactin Signaling | 1.79 | 0.816 | JAK2,MAPK3,PIK3C2G,PIK3CA,STAT5A,STAT5B |
| HER-2 Signaling in Breast Cancer | 1.79 | NA | AKT3,EGFR,MDM2,PARD3,PIK3C2G,PIK3CA |
| Regulation of eIF4 and p70S6K Signaling | 1.78 | -0.447 | AKT3,EIF4G1,EIF4G3,ITGA3,MAPK14,MAPK3,PIK3C2G,PIK3CA,RPS19 |
| B Cell Activating Factor Signaling | 1.77 | -1 | IKBKG,MAPK14,NFAT5,TRAF3 |
| IL-12 Signaling and Production in Macrophages | 1.75 | NA | AKT3,APOE,IKBKG,MAPK14,MAPK3,PIK3C2G,PIK3CA,RXRA |
| STAT3 Pathway | 1.75 | -0.707 | EGFR,FGFR3,IL17RB,JAK2,MAPK14,MAPK3,PIAS3,TGFBR1 |
| Activation of IRF by Cytosolic Pattern Recognition Receptors | 1.74 | 0.447 | ATF2,IKBKG,TANK,TBK1,TRAF3 |
| Gαs Signaling | 1.74 | -0.816 | ADCY6,ATF2,CREB1,GNB1,GNB2,MAPK3,RAPGEF4 |
| Th2 Pathway | 1.74 | 1.414 | ACVR2B,IL17RB,JAK2,PIK3C2G,PIK3CA,STAT5A,STAT5B,TGFBR1 |
| Role of NFAT in Cardiac Hypertrophy | 1.72 | -0.333 | ADCY6,AKT3,Calm1 (includes others),CSNK1A1,GNB1,GNB2,MAPK14,MAPK3,PIK3C2G,PIK3CA,TGFBR1 |
| D-myo-inositol (1,3,4)-trisphosphate Biosynthesis | 1.71 | NA | INPP5B,SYNJ1,SYNJ2 |
| Oncostatin M Signaling | 1.7 | 1 | JAK2,MAPK3,STAT5A,STAT5B |
| ErbB2-ErbB3 Signaling | 1.69 | 0.447 | MAPK3,PIK3C2G,PIK3CA,STAT5A,STAT5B |
| Wnt/Ca+ pathway | 1.69 | -2.236 | ATF2,CREB1,DVL2,FZD6,NFAT5 |
| Autophagy | 1.69 | NA | ATG13,BECN1,CTSH,NBR1,SQSTM1 |
| PPARα/RXRα Activation | 1.69 | -0.333 | ACVR2B,ADCY6,CKAP5,IKBKG,JAK2,MAPK14,MAPK3,RXRA,STAT5B,TGFBR1 |
| Rac Signaling | 1.68 | 0 | BAIAP2,CYFIP1,ITGA3,MAPK3,PARD3,PIK3C2G,PIK3CA |
| Role of RIG1-like Receptors in Antiviral Innate Immunity | 1.67 | 1 | IKBKG,TANK,TBK1,TRAF3 |
| IL-23 Signaling Pathway | 1.67 | 0 | AKT3,JAK2,PIK3C2G,PIK3CA |
| IL-17A Signaling in Gastric Cells | 1.66 | NA | EGFR,MAPK14,MAPK3 |
| Regulation of Cellular Mechanics by Calpain Protease | 1.64 | 0 | CAPNS1,EGFR,ITGA3,MAPK3,PXN |
| LPS/IL-1 Mediated Inhibition of RXR Function | 1.62 | 0 | ACSL1,ACSL4,ALAS1,APOE,CYP7A1,FMO1,FMO2,NR1H2,NR1H3,NR1H4,RXRA |
| 1D-myo-inositol Hexakisphosphate Biosynthesis II (Mammalian) | 1.62 | NA | INPP5B,SYNJ1,SYNJ2 |
| FAT10 Cancer Signaling Pathway | 1.61 | 0 | ACVR2B,AKT3,IKBKG,TGFBR1 |
| Factors Promoting Cardiogenesis in Vertebrates | 1.6 | NA | ACVR2B,APC,ATF2,FZD6,MAPK14,TGFBR1 |
| Role of NANOG in Mammalian Embryonic Stem Cell Pluripotency | 1.59 | -0.447 | AKT3,APC,FZD6,JAK2,MAPK3,PIK3C2G,PIK3CA |
| Wnt/β-catenin Signaling | 1.59 | 0 | ACVR2B,AKT3,APC,CSNK1A1,DVL2,FZD6,MDM2,SOX6,TGFBR1 |
| ErbB Signaling | 1.58 | -0.816 | EGFR,ERBB4,MAPK14,MAPK3,PIK3C2G,PIK3CA |
| IL-15 Production | 1.56 | NA | EGFR,EPHB4,ERBB4,FGFR3,JAK2,SYK,TNK2 |
| Sphingosine-1-phosphate Signaling | 1.56 | -0.816 | ADCY6,AKT3,MAPK3,PIK3C2G,PIK3CA,RHOBTB1,RHOT1 |
| Adrenomedullin signaling pathway | 1.55 | -0.632 | ADCY6,AKT3,Calm1 (includes others),MAPK14,MAPK3,NPR2,PIK3C2G,PIK3CA,RXRA,RXRB |
| iNOS Signaling | 1.55 | NA | Calm1 (includes others),IKBKG,JAK2,MAPK14 |
| Melanocyte Development and Pigmentation Signaling | 1.54 | -0.816 | ADCY6,ATF2,CREB1,MAPK3,PIK3C2G,PIK3CA |
| Endocannabinoid Cancer Inhibition Pathway | 1.53 | 0 | ADCY6,AKT3,ATF2,CREB1,MAPK14,MAPK3,PIK3C2G,PIK3CA |
| fMLP Signaling in Neutrophils | 1.52 | -0.816 | Calm1 (includes others),GNB1,GNB2,MAPK3,NFAT5,PIK3C2G,PIK3CA |
| Cholecystokinin/Gastrin-mediated Signaling | 1.51 | -0.816 | ATF2,EGFR,MAPK14,MAPK3,PXN,RHOBTB1,RHOT1 |
| Role of p14/p19ARF in Tumor Suppression | 1.49 | NA | MDM2,PIK3C2G,PIK3CA |
| CD28 Signaling in T Helper Cells | 1.49 | -1.134 | AKT3,Calm1 (includes others),IKBKG,NFAT5,PIK3C2G,PIK3CA,SYK |
| IL-6 Signaling | 1.49 | -0.378 | AKT3,IKBKG,JAK2,MAPK14,MAPK3,PIK3C2G,PIK3CA |
| Cleavage and Polyadenylation of Pre-mRNA | 1.48 | NA | CPSF1,PAPOLA |
| PAK Signaling | 1.48 | 0.816 | GIT1,ITGA3,MAPK3,PIK3C2G,PIK3CA,PXN |
| UVA-Induced MAPK Signaling | 1.48 | -1 | EGFR,MAPK14,MAPK3,PARP16,PIK3C2G,PIK3CA |
| Fcγ Receptor-mediated Phagocytosis in Macrophages and Monocytes | 1.46 | 0 | AKT3,FCGR2A,MAPK3,PIK3C2G,PXN,SYK |
| UVC-Induced MAPK Signaling | 1.46 | NA | ATR,EGFR,MAPK14,MAPK3 |
| Renin-Angiotensin Signaling | 1.46 | -0.816 | ADCY6,ATF2,JAK2,MAPK14,MAPK3,PIK3C2G,PIK3CA |
| Signaling by Rho Family GTPases | 1.44 | 0.378 | BAIAP2,CYFIP1,GNB1,GNB2,ITGA3,MAPK3,PARD3,PIK3C2G,PIK3CA,RHOBTB1,RHOT1 |
| CREB Signaling in Neurons | 1.43 | -1.414 | ADCY6,AKT3,ATF2,Calm1 (includes others),CREB1,GNB1,GNB2,MAPK3,PIK3C2G,PIK3CA |
| CCR3 Signaling in Eosinophils | 1.43 | -1 | Calm1 (includes others),GNB1,GNB2,MAPK14,MAPK3,PIK3C2G,PIK3CA |
| mTOR Signaling | 1.42 | 0 | AKT3,ATG13,EIF4G1,EIF4G3,MAPK3,PIK3C2G,PIK3CA,RHOBTB1,RHOT1,RPS19 |
| BER pathway | 1.42 | NA | LIG1,LIG3 |
| SAPK/JNK Signaling | 1.41 | 0 | ATF2,GNB1,MAP3K13,MAP4K5,PIK3C2G,PIK3CA |
| Glucocorticoid Receptor Signaling | 1.4 | NA | AKT3,CEBPA,CREB1,IKBKG,JAK2,MAPK14,MAPK3,NFAT5,PIK3C2G,PIK3CA,STAT5A,STAT5B,TAT,TGFBR1 |
| G-Protein Coupled Receptor Signaling | 1.4 | NA | ADCY6,AKT3,ATF2,CREB1,GRK2,IKBKG,MAPK3,PDE1A,PDE4B,PIK3C2G,PIK3CA,RAPGEF4 |
| Reelin Signaling in Neurons | 1.39 | NA | APOE,APP,ITGA3,PIK3C2G,PIK3CA |
| PPAR Signaling | 1.39 | 0 | IKBKG,MAPK3,NR1H3,RXRA,STAT5A,STAT5B |
| Superpathway of D-myo-inositol (1,4,5)-trisphosphate Metabolism | 1.38 | NA | INPP5B,SYNJ1,SYNJ2 |
| NRF2-mediated Oxidative Stress Response | 1.38 | -0.816 | DNAJB2,FMO1,KEAP1,MAPK14,MAPK3,PIK3C2G,PIK3CA,PRDX1,SQSTM1 |
| Agrin Interactions at Neuromuscular Junction | 1.37 | 0.447 | EGFR,ERBB4,ITGA3,MAPK3,PXN |
| GDNF Family Ligand-Receptor Interactions | 1.37 | -1.342 | CREB1,GFRA1,MAPK3,PIK3C2G,PIK3CA |
| Amyotrophic Lateral Sclerosis Signaling | 1.36 | 1.342 | AKT3,BIRC2,CAPNS1,GPX1,PIK3C2G,PIK3CA |
| IGF-1 Signaling | 1.36 | 0 | AKT3,JAK2,MAPK3,PIK3C2G,PIK3CA,PXN |
| Adipogenesis pathway | 1.34 | NA | CEBPA,FGFR3,FZD6,LPIN1,NOCT,SIN3A,STAT5B |
| Neurotrophin/TRK Signaling | 1.33 | -1.342 | ATF2,CREB1,MAPK3,PIK3C2G,PIK3CA |
| G Protein Signaling Mediated by Tubby | 1.32 | NA | GNB1,GNB2,JAK2 |
| Paxillin Signaling | 1.31 | 0.447 | ITGA3,ITGAV,MAPK14,PIK3C2G,PIK3CA,PXN |
| Sumoylation Pathway | 1.31 | NA | CEBPA,MDM2,PML,RANGAP1,RHOBTB1,RHOT1 |
| Clathrin-mediated Endocytosis Signaling | 1.31 | NA | APOE,ARRB1,CD2AP,FGF21,MDM2,MYO6,PIK3C2G,PIK3CA,SYNJ1 |
| EIF2 Signaling | 1.3 | -1.633 | AKT3,EIF4G1,EIF4G3,MAPK3,PIK3C2G,PIK3CA,PTBP1,RPL37,RPL38,RPS19 |
| Xenobiotic Metabolism Signaling | 1.3 | NA | CES3,ESD,FMO1,FMO2,KEAP1,MAP3K13,MAPK14,MAPK3,PIK3C2G,PIK3CA,RXRA,UGT2B28 |
| ERK/MAPK Signaling | 1.3 | -1 | ATF2,CREB1,ITGA3,MAPK3,PIK3C2G,PIK3CA,PPP1R3C,PXN,RAPGEF4 |

**Table 3.** Significant pathways for differentially expressed transcripts in ^56^Fe vs. non-irradiated control at 4 months analyzed by IPA.

| **Ingenuity Canonical Pathways** | **-log(p-value)** | **z-score** | **Molecules** |
| --- | --- | --- | --- |
| Acute Phase Response Signaling | 7.89 | -1.069 | C1R,C4A/C4B,GRB2,HMOX2,HP,HRAS,IL1R1,IL33,MAPK14,MAPK9,NR3C1,PIK3CD,SAA1,SERPINA1,SERPINF1,SOCS1,SOCS2,SOCS3,STAT3,TCF3,TF |
| Prolactin Signaling | 7.45 | -1.387 | FYN,GRB2,HRAS,NR3C1,PIK3C2G,PIK3C3,PIK3CD,PRKCB,PRLR,SOCS1,SOCS2,SOCS3,STAT3,STAT5A |
| PPARα/RXRα Activation | 6.65 | -0.688 | ADCY3,ADCY7,AIP,CD36,CYP2C18,CYP2C8,FASN,GHR,GRB2,HELZ2,HRAS,HSP90AB1,IL1R1,LPL,MAPK14,MEF2C,PRKAA1,PRKACA,PRKCB,RXRA |
| LXR/RXR Activation | 6.61 | 1.155 | ACACA,APOA4,C4A/C4B,CD36,FASN,IL1R1,IL33,LPL,PON3,RXRA,RXRB,SAA1,SCD,SERPINA1,SERPINF1,TF |
| Role of JAK2 in Hormone-like Cytokine Signaling | 5.68 | NA | GHR,PRLR,SIRPA,SOCS1,SOCS2,SOCS3,STAT3,STAT5A |
| TR/RXR Activation | 5.35 | NA | ACACA,COL6A3,FASN,HP,ME1,PIK3C2G,PIK3C3,PIK3CD,RXRA,RXRB,TBL1XR1,THRSP |
| IL-9 Signaling | 4.72 | 0 | PIK3C2G,PIK3C3,PIK3CD,SOCS2,SOCS3,STAT3,STAT5A |
| IGF-1 Signaling | 4.67 | -0.905 | GRB2,HRAS,IGFBP2,PIK3C2G,PIK3C3,PIK3CD,PRKACA,PXN,SOCS1,SOCS2,SOCS3,STAT3 |
| RAR Activation | 4.63 | NA | ADCY3,ADCY7,GTF2H1,MAPK14,MAPK9,PBRM1,PIK3CD,PML,PRKACA,PRKCB,RDH16,RXRA,RXRB,SMARCA2,SMARCA4,STAT5A,TNIP1 |
| IL-6 Signaling | 4.6 | -0.832 | GRB2,HRAS,IL1R1,IL33,MAPK14,MAPK9,MCL1,PIK3C2G,PIK3C3,PIK3CD,SOCS1,SOCS3,STAT3 |
| Growth Hormone Signaling | 4.6 | -1.265 | GHR,PIK3C2G,PIK3C3,PIK3CD,PRKCB,SOCS1,SOCS2,SOCS3,STAT3,STAT5A |
| Paxillin Signaling | 4.54 | -0.632 | ACTN4,DOCK1,GRB2,HRAS,ITGAV,ITGB2,MAPK14,MAPK9,PIK3C2G,PIK3C3,PIK3CD,PXN |
| Role of JAK family kinases in IL-6-type Cytokine Signaling | 4.44 | NA | MAPK14,MAPK9,SOCS1,SOCS3,STAT3,STAT5A |
| JAK/Stat Signaling | 4.35 | -0.632 | GRB2,HRAS,PIK3C2G,PIK3C3,PIK3CD,SOCS1,SOCS2,SOCS3,STAT3,STAT5A |
| IL-7 Signaling Pathway | 4.35 | -0.632 | FYN,GRB2,GSK3A,MAPK14,MCL1,PIK3C2G,PIK3C3,PIK3CD,SOCS1,STAT5A |
| HGF Signaling | 4.35 | -0.302 | DOCK1,ELF4,GRB2,HRAS,Map3k7,MAPK9,PIK3C2G,PIK3C3,PIK3CD,PRKCB,PXN,STAT3 |
| FXR/RXR Activation | 4.19 | NA | APOA4,C4A/C4B,FASN,IL33,LPL,MAPK9,PKLR,PON3,RXRA,SAA1,SERPINA1,SERPINF1,TF |
| HER-2 Signaling in Breast Cancer | 4.17 | NA | GRB2,GSK3A,HRAS,ITGB2,PARD3,PIK3C2G,PIK3C3,PIK3CD,PRKCB,TSC1 |
| Role of JAK1 and JAK3 in γc Cytokine Signaling | 4.12 | NA | GRB2,HRAS,PIK3C2G,PIK3C3,PIK3CD,SOCS1,SOCS3,STAT3,STAT5A |
| PXR/RXR Activation | 4.07 | NA | ALAS1,ALDH3A2,CES3,CYP2A6 (includes others),CYP2C8,NR3C1,PRKACA,RXRA,SCD |
| Xenobiotic Metabolism Signaling | 4.06 | NA | AIP,ALDH3A2,CAMK2G,CES3,CYP2C8,ESD,FMO1,GSTM5,HDAC5,HRAS,HSP90AB1,Map3k7,MAPK14,MAPK9,MGST1,PIK3C2G,PIK3C3,PIK3CD,PRKCB,RXRA |
| Apelin Endothelial Signaling Pathway | 4.02 | -0.577 | ADCY3,ADCY7,GNAI2,HDAC5,HRAS,MAPK9,MEF2C,PIK3C2G,PIK3C3,PIK3CD,PRKAA1,PRKCB |
| Adrenomedullin signaling pathway | 3.97 | 0 | ADCY3,ADCY7,CFH,GRB2,GSK3A,GUCY2C,HRAS,IL33,MAPK14,MAPK9,PIK3C2G,PIK3C3,PIK3CD,PRKACA,RXRA,RXRB |
| Insulin Receptor Signaling | 3.93 | -2.111 | ACLY,FYN,GRB2,GSK3A,HRAS,PIK3C2G,PIK3C3,PIK3CD,PRKACA,SOCS3,SYNJ1,TRIP10,TSC1 |
| Renin-Angiotensin Signaling | 3.89 | -1 | ADCY3,ADCY7,GRB2,HRAS,MAPK14,MAPK9,PIK3C2G,PIK3C3,PIK3CD,PRKACA,PRKCB,STAT3 |
| Acute Myeloid Leukemia Signaling | 3.83 | 0 | GRB2,HRAS,KIT,PIK3C2G,PIK3C3,PIK3CD,PML,STAT3,STAT5A,TCF3 |
| B Cell Receptor Signaling | 3.82 | -1.387 | CAMK2G,GRB2,GSK3A,HRAS,Map3k7,MAPK14,MAPK9,MEF2C,PIK3C2G,PIK3C3,PIK3CD,PRKCB,PTPRC,SYNJ1,TCF3 |
| Leptin Signaling in Obesity | 3.7 | 0 | ADCY3,ADCY7,GRB2,PIK3C2G,PIK3C3,PIK3CD,PRKACA,SOCS3,STAT3 |
| Erythropoietin Signaling | 3.66 | NA | GRB2,HRAS,PIK3C2G,PIK3C3,PIK3CD,PRKCB,SOCS1,SOCS3,STAT5A |
| Endocannabinoid Developing Neuron Pathway | 3.63 | -0.632 | ADCY3,ADCY7,GNAI2,HRAS,MAPK14,MAPK9,PIK3C2G,PIK3C3,PIK3CD,PRKACA,STAT3 |
| Role of NFAT in Cardiac Hypertrophy | 3.6 | -0.775 | ADCY3,ADCY7,CACNA1A,CAMK2G,GNAI2,GRB2,HDAC5,HRAS,MAPK14,MAPK9,MEF2C,PIK3C2G,PIK3C3,PIK3CD,PRKACA,PRKCB |
| Apelin Adipocyte Signaling Pathway | 3.58 | -0.333 | ADCY3,ADCY7,GNAI2,GPX1,MAPK14,MAPK9,MGST1,PRKAA1,PRKACA |
| ErbB2-ErbB3 Signaling | 3.55 | 0 | GRB2,GSK3A,HRAS,PIK3C2G,PIK3C3,PIK3CD,STAT3,STAT5A |
| IL-22 Signaling | 3.47 | -0.447 | MAPK14,MAPK9,SOCS3,STAT3,STAT5A |
| Thrombopoietin Signaling | 3.45 | 0 | GRB2,HRAS,PIK3C2G,PIK3C3,PIK3CD,PRKCB,STAT3,STAT5A |
| Type II Diabetes Mellitus Signaling | 3.43 | -2.121 | ACSL1,CACNA1A,CD36,MAPK9,PIK3C2G,PIK3C3,PIK3CD,PKLR,PRKAA1,PRKCB,SOCS1,SOCS2,SOCS3 |
| LPS/IL-1 Mediated Inhibition of RXR Function | 3.43 | 0.447 | ACSL1,ALAS1,ALDH3A2,Cyp2a12/Cyp2a22,CYP2A6 (includes others),CYP2C8,CYP4A11,FABP2,FMO1,GSTM5,IL1R1,IL33,MAPK9,MGST1,RXRA,XPO1 |
| IL-4 Signaling | 3.34 | NA | GRB2,HLA-A,HRAS,NR3C1,PIK3C2G,PIK3C3,PIK3CD,SOCS1,SYNJ1 |
| FGF Signaling | 3.31 | -1 | FGFR2,FGFR3,GRB2,HRAS,MAPK14,PIK3C2G,PIK3C3,PIK3CD,STAT3 |
| GM-CSF Signaling | 3.28 | -0.707 | CAMK2G,GRB2,HRAS,PIK3C2G,PIK3C3,PIK3CD,PRKCB,STAT3 |
| 14-3-3-mediated Signaling | 3.23 | -1.265 | GRB2,GSK3A,HRAS,MAPK9,PIK3C2G,PIK3C3,PIK3CD,PRKCB,STK11,TSC1,TUBB2A |
| Axonal Guidance Signaling | 3.22 | NA | ABLIM1,ADAM17,ADAM23,ADAM9,ARPC1A,DOCK1,EPHB4,FYN,GIT1,GNAI2,GRB2,HERC2,HRAS,NRP2,PIK3C2G,PIK3C3,PIK3CD,PLXNB1,PRKACA,PRKCB,PXN,RTN4,SEMA4B,SEMA4C,SEMA6D,TUBB2A |
| Clathrin-mediated Endocytosis Signaling | 3.17 | NA | APOA4,ARPC1A,EPS15,GRB2,ITGB2,MYO6,PICALM,PIK3C2G,PIK3C3,PIK3CD,SERPINA1,SH3GL1,SYNJ1,TF |
| Germ Cell-Sertoli Cell Junction Signaling | 3.14 | NA | ACTN4,CDH1,HRAS,Map3k7,MAPK14,MAPK9,PIK3C2G,PIK3C3,PIK3CD,PXN,RHOBTB1,SORBS1,TUBB2A |
| Macropinocytosis Signaling | 3.08 | -1.633 | ABI1,ACTN4,HRAS,ITGB2,PIK3C2G,PIK3C3,PIK3CD,PRKCB |
| EGF Signaling | 3.07 | -0.378 | GRB2,HRAS,MAPK14,PIK3C2G,PIK3C3,PIK3CD,STAT3 |
| Adipogenesis pathway | 3.06 | NA | EZH2,FGFR2,FGFR3,GTF2H1,HDAC5,Kat6b,KAT7,KMT2B,LPL,SETDB1,TBL1XR1 |
| GNRH Signaling | 3.05 | -0.577 | ADCY3,ADCY7,CACNA1A,CAMK2G,GNAI2,GRB2,HRAS,Map3k7,MAPK14,MAPK9,PRKACA,PRKCB,PXN |
| NF-κB Signaling | 3.05 | -1.387 | FGFR2,FGFR3,GHR,HRAS,IL1R1,IL33,PIK3C2G,PIK3C3,PIK3CD,PRKACA,PRKCB,TNFAIP3,TNIP1 |
| STAT3 Pathway | 3.04 | -2.53 | FGFR2,FGFR3,GHR,HRAS,IL1R1,MAPK14,MAPK9,SOCS1,SOCS2,SOCS3,STAT3 |
| Melanocyte Development and Pigmentation Signaling | 3.03 | -0.333 | ADCY3,ADCY7,GRB2,HRAS,KIT,PIK3C2G,PIK3C3,PIK3CD,PRKACA |
| Leukocyte Extravasation Signaling | 3 | -1.941 | ACTN4,ARHGAP4,CLDN12,GNAI2,ITGB2,MAPK14,MAPK9,PIK3C2G,PIK3C3,PIK3CD,PRKCB,PXN,RAPGEF4,RASGRP1 |
| FcγRIIB Signaling in B Lymphocytes | 2.97 | -0.378 | CACNA1A,DOK1,GRB2,HRAS,MAPK9,PIK3C2G,PIK3C3,PIK3CD |
| IL-3 Signaling | 2.97 | 0 | GRB2,HRAS,PIK3C2G,PIK3C3,PIK3CD,PRKCB,STAT3,STAT5A |
| Non-Small Cell Lung Cancer Signaling | 2.97 | -0.447 | CDK4,GRB2,HRAS,PIK3C2G,PIK3C3,PIK3CD,RXRA,RXRB |
| Apelin Pancreas Signaling Pathway | 2.97 | 0 | MAPK9,PIK3C2G,PIK3C3,PIK3CD,PRKAA1,PRKACA |
| p53 Signaling | 2.97 | 1.342 | ATR,CDK4,COQ8A,GADD45G,MAPK14,PIK3C2G,PIK3C3,PIK3CD,PML |
| G-Protein Coupled Receptor Signaling | 2.94 | NA | ADCY3,ADCY7,CAMK2G,FYN,GNAI2,GRB2,GRK2,HRAS,PIK3C2G,PIK3C3,PIK3CD,PRKACA,PRKCB,RAPGEF4,RASGRP1,RGS16,STAT3 |
| IL-2 Signaling | 2.9 | -0.378 | GRB2,HRAS,PIK3C2G,PIK3C3,PIK3CD,SOCS1,STAT5A |
| Hereditary Breast Cancer Signaling | 2.89 | NA | ATR,CDK4,GADD45G,HDAC5,HRAS,PBRM1,PIK3C2G,PIK3C3,PIK3CD,SMARCA2,SMARCA4 |
| Fc Epsilon RI Signaling | 2.83 | -1.897 | FYN,GRB2,HRAS,MAPK14,MAPK9,PIK3C2G,PIK3C3,PIK3CD,PRKCB,SYNJ1 |
| FLT3 Signaling in Hematopoietic Progenitor Cells | 2.83 | 0 | GRB2,HRAS,MAPK14,PIK3C2G,PIK3C3,PIK3CD,STAT3,STAT5A |
| CD40 Signaling | 2.82 | -1.134 | MAPK14,MAPK9,PIK3C2G,PIK3C3,PIK3CD,STAT3,TNFAIP3 |
| Chronic Myeloid Leukemia Signaling | 2.82 | NA | CDK4,GRB2,HDAC5,HRAS,PIK3C2G,PIK3C3,PIK3CD,RBL2,STAT5A |
| NRF2-mediated Oxidative Stress Response | 2.81 | -1.134 | DNAJB2,FMO1,GSTM5,HERPUD1,HRAS,MAPK14,MAPK9,MGST1,PIK3C2G,PIK3C3,PIK3CD,PRKCB,SQSTM1 |
| Integrin Signaling | 2.78 | -1.155 | ACTN4,ARPC1A,DOCK1,FYN,GIT1,GRB2,HRAS,ITGAV,ITGB2,PIK3C2G,PIK3C3,PIK3CD,PXN,RHOBTB1 |
| VEGF Signaling | 2.73 | 0 | ACTN4,EIF2S3,GRB2,HRAS,PIK3C2G,PIK3C3,PIK3CD,PRKCB,PXN |
| Aryl Hydrocarbon Receptor Signaling | 2.72 | -1.414 | AIP,ALDH3A2,ATR,CDK4,GSTM5,HSP90AB1,MGST1,RBL2,RXRA,RXRB,SMARCA4 |
| eNOS Signaling | 2.71 | -0.707 | ADCY3,ADCY7,AQP4,AQP8,CHRNA4,HSP90AB1,PIK3C2G,PIK3C3,PIK3CD,PRKAA1,PRKACA,PRKCB |
| Telomerase Signaling | 2.7 | 0 | ELF4,GRB2,HDAC5,HRAS,HSP90AB1,PIK3C2G,PIK3C3,PIK3CD,TERT |
| PTEN Signaling | 2.7 | 1.897 | FGFR2,FGFR3,GHR,GRB2,GSK3A,HRAS,MAGI1,MAST2,PIK3CD,SYNJ1 |
| Endocannabinoid Cancer Inhibition Pathway | 2.69 | 0.302 | ADCY3,ADCY7,CDH1,GNAI2,MAPK14,PIK3C2G,PIK3C3,PIK3CD,PRKAA1,PRKACA,TCF3 |
| Molecular Mechanisms of Cancer | 2.67 | NA | ADCY3,ADCY7,ATR,CAMK2G,CDH1,CDK4,FYN,GNAI2,GRB2,GSK3A,HRAS,MAPK14,MAPK9,PIK3C2G,PIK3C3,PIK3CD,PRKACA,PRKCB,RASGRP1,RHOBTB1,TCF3 |
| ERK/MAPK Signaling | 2.67 | -0.277 | DOCK1,ELF4,FYN,GRB2,HRAS,PIK3C2G,PIK3C3,PIK3CD,PRKACA,PRKCB,PXN,RAPGEF4,STAT3 |
| CXCR4 Signaling | 2.64 | -0.905 | ADCY3,ADCY7,DOCK1,GNAI2,HRAS,MAPK9,PIK3C2G,PIK3C3,PIK3CD,PRKCB,PXN,RHOBTB1 |
| Melanoma Signaling | 2.64 | -0.447 | CDH1,CDK4,HRAS,PIK3C2G,PIK3C3,PIK3CD |
| UVB-Induced MAPK Signaling | 2.64 | -1.633 | MAPK14,MAPK9,PIK3C2G,PIK3C3,PIK3CD,PRKCB |
| ErbB4 Signaling | 2.63 | -1.134 | ADAM17,GRB2,HRAS,PIK3C2G,PIK3C3,PIK3CD,PRKCB |
| Cardiac Hypertrophy Signaling | 2.61 | 0 | ADCY3,ADCY7,CACNA1A,GNAI2,GRB2,HRAS,Map3k7,MAPK14,MAPK9,MEF2C,PIK3C2G,PIK3C3,PIK3CD,PRKACA,RHOBTB1 |
| Acetone Degradation I (to Methylglyoxal) | 2.58 | -1.342 | CYP2A6 (includes others),CYP2C18,CYP2C8,CYP4A11,POR |
| Nitric Oxide Signaling in the Cardiovascular System | 2.56 | 1 | CACNA1A,GUCY2C,HSP90AB1,PIK3C2G,PIK3C3,PIK3CD,PRKAA1,PRKACA,PRKCB |
| IL-15 Signaling | 2.56 | NA | HRAS,MAPK14,PIK3C2G,PIK3C3,PIK3CD,STAT3,STAT5A |
| PDGF Signaling | 2.55 | 0 | GRB2,HRAS,PIK3C2G,PIK3C3,PIK3CD,PRKCB,STAT3,SYNJ1 |
| Virus Entry via Endocytic Pathways | 2.54 | NA | FLNA,FYN,HLA-A,HRAS,ITGB2,PIK3C2G,PIK3C3,PIK3CD,PRKCB |
| Complement System | 2.53 | NA | C1R,C4A/C4B,C6,CFH,ITGB2 |
| AMPK Signaling | 2.53 | 0.632 | ACACA,CHRNA4,FASN,MAPK14,PBRM1,PIK3C2G,PIK3C3,PIK3CD,PRKAA1,PRKACA,SMARCA2,SMARCA4,STK11,TSC1 |
| Breast Cancer Regulation by Stathmin1 | 2.5 | NA | ADCY3,ADCY7,CAMK2G,GNAI2,GRB2,HRAS,PIK3C2G,PIK3C3,PIK3CD,PRKACA,PRKCB,TUBB2A,UHMK1 |
| ErbB Signaling | 2.49 | -1.414 | GRB2,HRAS,MAPK14,MAPK9,PIK3C2G,PIK3C3,PIK3CD,PRKCB |
| Ephrin Receptor Signaling | 2.48 | -0.905 | ABI1,ARPC1A,DOK1,EPHB4,FYN,GNAI2,GRB2,HRAS,PIK3C2G,PXN,SORBS1,STAT3 |
| Inhibition of Angiogenesis by TSP1 | 2.48 | -2.236 | CD36,CD47,FYN,MAPK14,MAPK9 |
| Glioma Signaling | 2.41 | -1.134 | CAMK2G,CDK4,GRB2,HRAS,PIK3C2G,PIK3C3,PIK3CD,PRKCB,RBL2 |
| Angiopoietin Signaling | 2.4 | 1 | GRB2,HRAS,PIK3C2G,PIK3C3,PIK3CD,STAT5A,TNIP1 |
| Glucocorticoid Receptor Signaling | 2.38 | NA | BAG1,GRB2,GTF2H1,HRAS,HSP90AB1,MAPK14,MAPK9,NR3C1,PBRM1,PIK3C2G,PIK3C3,PIK3CD,PRKAA1,PRKACA,SMARCA2,SMARCA4,STAT3,STAT5A |
| FAK Signaling | 2.38 | NA | DOCK1,FYN,GRB2,HRAS,PIK3C2G,PIK3C3,PIK3CD,PXN |
| HMGB1 Signaling | 2.38 | -1 | HRAS,IL1R1,IL33,Kat6b,KAT7,MAPK14,MAPK9,PIK3C2G,PIK3C3,PIK3CD,RHOBTB1 |
| Reelin Signaling in Neurons | 2.37 | NA | FYN,ITGB2,MAPK8IP3,MAPK9,PIK3C2G,PIK3C3,PIK3CD |
| Stearate Biosynthesis I (Animals) | 2.36 | -1.633 | ACOT1,ACOT2,ACSL1,CYP4A11,ELOVL6,FASN |
| White Adipose Tissue Browning Pathway | 2.35 | 0.632 | ADCY3,ADCY7,CACNA1A,FGFR2,FGFR3,MAPK14,PRKAA1,PRKACA,RXRA,RXRB |
| PAK Signaling | 2.35 | -1.414 | GIT1,GRB2,HRAS,MAPK9,PIK3C2G,PIK3C3,PIK3CD,PXN |
| Tec Kinase Signaling | 2.34 | -0.333 | FYN,GNAI2,GTF2I,MAPK9,PIK3C2G,PIK3C3,PIK3CD,PRKCB,RHOBTB1,STAT3,STAT5A |
| GDNF Family Ligand-Receptor Interactions | 2.34 | -1.134 | DOK1,GRB2,HRAS,MAPK9,PIK3C2G,PIK3C3,PIK3CD |
| CNTF Signaling | 2.33 | 0 | GRB2,HRAS,PIK3C2G,PIK3C3,PIK3CD,STAT3 |
| ILK Signaling | 2.3 | -0.632 | ACTN4,CDH1,DOCK1,FLNA,GSK3A,ITGB2,MAPK9,PIK3C2G,PIK3C3,PIK3CD,PXN,RHOBTB1 |
| Regulation of the Epithelial-Mesenchymal Transition Pathway | 2.3 | NA | ADAM17,CDH1,FGFR2,FGFR3,GRB2,HRAS,PIK3C2G,PIK3C3,PIK3CD,STAT3,TCF3,ZEB2 |
| Bupropion Degradation | 2.3 | -1 | CYP2A6 (includes others),CYP2C18,CYP2C8,POR |
| Th1 Pathway | 2.29 | -0.707 | GRB2,HLA-A,ITGB2,PIK3C2G,PIK3C3,PIK3CD,SOCS1,SOCS3,STAT3 |
| Sertoli Cell-Sertoli Cell Junction Signaling | 2.28 | NA | ACTN4,CDH1,CLDN12,EPB41,GSK3A,HRAS,Map3k7,MAPK14,MAPK9,PRKACA,SORBS1,TUBB2A |
| Cholecystokinin/Gastrin-mediated Signaling | 2.27 | -1.414 | GRB2,HRAS,IL33,MAPK14,MAPK9,MEF2C,PRKCB,PXN,RHOBTB1 |
| Endometrial Cancer Signaling | 2.25 | -0.816 | CDH1,GRB2,HRAS,PIK3C2G,PIK3C3,PIK3CD |
| CD28 Signaling in T Helper Cells | 2.25 | -1.414 | ARPC1A,FYN,GRB2,HLA-A,MAPK9,PIK3C2G,PIK3C3,PIK3CD,PTPRC |
| Docosahexaenoic Acid (DHA) Signaling | 2.25 | NA | GSK3A,PIK3C2G,PIK3C3,PIK3CD,SERPINF1 |
| IL-23 Signaling Pathway | 2.25 | -0.447 | PIK3C2G,PIK3C3,PIK3CD,SOCS3,STAT3 |
| Mouse Embryonic Stem Cell Pluripotency | 2.25 | 0 | GRB2,HRAS,MAPK14,PIK3C2G,PIK3C3,PIK3CD,STAT3,TCF3 |
| SAPK/JNK Signaling | 2.25 | -1.134 | GRB2,HRAS,MAP4K5,MAPK8IP3,MAPK9,PIK3C2G,PIK3C3,PIK3CD |
| LPS-stimulated MAPK Signaling | 2.25 | -1.89 | HRAS,MAPK14,MAPK9,PIK3C2G,PIK3C3,PIK3CD,PRKCB |
| Th1 and Th2 Activation Pathway | 2.22 | NA | GRB2,HLA-A,IL33,ITGB2,PIK3C2G,PIK3C3,PIK3CD,SOCS1,SOCS3,STAT3,STAT5A |
| PPAR Signaling | 2.22 | -0.707 | AIP,GRB2,HRAS,HSP90AB1,IL1R1,IL33,RXRA,STAT5A |
| Apelin Cardiomyocyte Signaling Pathway | 2.22 | -1.414 | GNAI2,MAPK14,MAPK9,PIK3C2G,PIK3C3,PIK3CD,PRKCB,SLC9A6 |
| NF-κB Activation by Viruses | 2.22 | -1.89 | HRAS,ITGAV,ITGB2,PIK3C2G,PIK3C3,PIK3CD,PRKCB |
| Cardiac Hypertrophy Signaling (Enhanced) | 2.19 | -0.853 | ADCY3,ADCY7,CACNA1A,CAMK2G,FGFR2,FGFR3,GHR,GNAI2,GSK3A,HDAC5,HRAS,IL1R1,IL33,Map3k7,MAPK14,MAPK9,MEF2C,PIK3C2G,PIK3C3,PIK3CD,PRKACA,PRKCB,STAT3 |
| Protein Ubiquitination Pathway | 2.18 | NA | ANAPC5,BAG1,BIRC6,DNAJB2,HLA-A,HSP90AB1,PAN2,PSMD13,PSMD2,UBE2M,UBE4B,USO1,USP19,USP24,USP36 |
| Endothelin-1 Signaling | 2.16 | -1.155 | ADCY3,ADCY7,GNAI2,GRB2,GUCY2C,HRAS,MAPK14,MAPK9,PIK3C2G,PIK3C3,PIK3CD,PRKCB |
| Myc Mediated Apoptosis Signaling | 2.15 | NA | GRB2,HRAS,MAPK9,PIK3C2G,PIK3C3,PIK3CD |
| IL-17A Signaling in Airway Cells | 2.12 | -0.816 | MAPK14,MAPK9,PIK3C2G,PIK3C3,PIK3CD,STAT3 |
| Epithelial Adherens Junction Signaling | 2.11 | NA | ACTN4,ARPC1A,CDH1,CLIP1,HRAS,MAGI1,PARD3,SORBS1,TCF3,TUBB2A |
| T Cell Receptor Signaling | 2.08 | NA | FYN,GRB2,HRAS,PIK3C2G,PIK3C3,PIK3CD,PTPRC,RASGRP1 |
| Colorectal Cancer Metastasis Signaling | 2.08 | 0 | ADCY3,ADCY7,CDH1,GRB2,GRK2,HRAS,MAPK9,PIK3C2G,PIK3C3,PIK3CD,PRKACA,RHOBTB1,STAT3,TCF3 |
| PKCθ Signaling in T Lymphocytes | 2.04 | -1 | CACNA1A,CAMK2G,FYN,GRB2,HLA-A,HRAS,Map3k7,PIK3C2G,PIK3C3,PIK3CD |
| SPINK1 General Cancer Pathway | 2.03 | -0.816 | HRAS,Mt2,PIK3C2G,PIK3C3,PIK3CD,STAT3 |
| VEGF Family Ligand-Receptor Interactions | 2.03 | -0.816 | GRB2,HRAS,NRP2,PIK3C2G,PIK3C3,PIK3CD,PRKCB |
| IL-12 Signaling and Production in Macrophages | 2.02 | NA | APOA4,MAPK14,MAPK9,PIK3C2G,PIK3C3,PIK3CD,PRKCB,RXRA,SERPINA1 |
| Gap Junction Signaling | 2 | NA | ADCY3,ADCY7,GNAI2,GRB2,GUCY2C,HRAS,PIK3C2G,PIK3C3,PIK3CD,PRKACA,PRKCB,TUBB2A |
| Regulation of eIF4 and p70S6K Signaling | 2 | -0.447 | EIF2S3,EIF4G1,EIF4G3,GRB2,HRAS,MAPK14,PABPC1,PIK3C2G,PIK3C3,PIK3CD |
| HOTAIR Regulatory Pathway | 2 | -0.632 | ATXN1,CDH1,EZH2,KMT2A,PIK3C2G,PIK3C3,PIK3CD,SETDB1,STAT3,TCF3 |
| P2Y Purigenic Receptor Signaling Pathway | 2 | -1 | ADCY3,ADCY7,GNAI2,HRAS,PIK3C2G,PIK3C3,PIK3CD,PRKACA,PRKCB |
| Th2 Pathway | 2 | 0.816 | GRB2,HLA-A,IL33,ITGB2,PIK3C2G,PIK3C3,PIK3CD,SOCS3,STAT5A |
| Role of NFAT in Regulation of the Immune Response | 1.98 | -0.632 | FYN,GNAI2,GRB2,GSK3A,HLA-A,HRAS,MEF2C,PIK3C2G,PIK3C3,PIK3CD,XPO1 |
| Retinol Biosynthesis | 1.98 | 0.447 | CES3,DDHD2,ESD,LPL,PNPLA5 |
| UVC-Induced MAPK Signaling | 1.98 | -2 | ATR,HRAS,MAPK14,MAPK9,PRKCB |
| Thrombin Signaling | 1.96 | -0.905 | ADCY3,ADCY7,CAMK2G,GNAI2,GRB2,HRAS,MAPK14,PIK3C2G,PIK3C3,PIK3CD,PRKCB,RHOBTB1 |
| CREB Signaling in Neurons | 1.94 | -0.302 | ADCY3,ADCY7,CACNA1A,CAMK2G,GNAI2,GRB2,HRAS,PIK3C2G,PIK3C3,PIK3CD,PRKACA,PRKCB |
| Thyroid Cancer Signaling | 1.94 | NA | CDH1,HRAS,RXRA,RXRB,TCF3 |
| IL-1 Signaling | 1.93 | 0 | ADCY3,ADCY7,GNAI2,IL1R1,MAPK14,MAPK9,PRKACA |
| mTOR Signaling | 1.93 | -1.667 | ATG13,EIF4G1,EIF4G3,HRAS,PIK3C2G,PIK3C3,PIK3CD,PRKAA1,PRKCB,RHOBTB1,STK11,TSC1 |
| GADD45 Signaling | 1.9 | NA | ATR,CDK4,GADD45G |
| Small Cell Lung Cancer Signaling | 1.89 | 0 | CDK4,PIK3C2G,PIK3C3,PIK3CD,RXRA,RXRB |
| Production of Nitric Oxide and Reactive Oxygen Species in Macrophages | 1.87 | -0.632 | APOA4,Map3k7,MAPK14,MAPK9,PIK3C2G,PIK3C3,PIK3CD,PRKCB,RHOBTB1,SERPINA1,SIRPA |
| Caveolar-mediated Endocytosis Signaling | 1.86 | NA | ARCN1,FLNA,FYN,HLA-A,ITGAV,ITGB2 |
| ATM Signaling | 1.86 | -0.447 | ATR,GADD45G,HERC2,MAPK14,MAPK9,TLK2,TRRAP |
| Glioma Invasiveness Signaling | 1.86 | -1.342 | HRAS,ITGAV,PIK3C2G,PIK3C3,PIK3CD,RHOBTB1 |
| Superpathway of Inositol Phosphate Compounds | 1.83 | 0 | INPP5E,PI4KB,PIK3C2G,PIK3C3,PIK3CD,PPP1R16B,PPP4C,PTPRC,SEC16A,SIRPA,SOCS3,SYNJ1 |
| Role of Tissue Factor in Cancer | 1.82 | NA | FYN,HRAS,ITGAV,MAPK14,PIK3C2G,PIK3C3,PIK3CD,STAT5A |
| Role of IL-17A in Arthritis | 1.81 | NA | MAPK14,MAPK9,PIK3C2G,PIK3C3,PIK3CD |
| Natural Killer Cell Signaling | 1.79 | NA | FYN,GRB2,HRAS,PIK3C2G,PIK3C3,PIK3CD,PRKCB,SYNJ1 |
| Estrogen-Dependent Breast Cancer Signaling | 1.79 | -0.447 | HRAS,PIK3C2G,PIK3C3,PIK3CD,STAT5A,TERT |
| Agrin Interactions at Neuromuscular Junction | 1.76 | -1.342 | AGRN,HRAS,ITGB2,MAPK9,PKLR,PXN |
| Triacylglycerol Degradation | 1.75 | -0.447 | Ces2e,CES3,DDHD2,LPL,PNPLA5 |
| IL-8 Signaling | 1.74 | -1.667 | CDH1,GNAI2,HRAS,ITGAV,ITGB2,MAPK9,PIK3C2G,PIK3C3,PIK3CD,PRKCB,RHOBTB1 |
| IL-17 Signaling | 1.74 | NA | HRAS,MAPK14,MAPK9,PIK3C2G,PIK3C3,PIK3CD |
| D-myo-inositol (1,4,5)-trisphosphate Degradation | 1.72 | NA | INPP5E,SEC16A,SYNJ1 |
| 3-phosphoinositide Biosynthesis | 1.72 | 0 | PI4KB,PIK3C2G,PIK3C3,PIK3CD,PPP1R16B,PPP4C,PTPRC,SIRPA,SOCS3,SYNJ1 |
| Opioid Signaling Pathway | 1.7 | -0.832 | ADCY3,ADCY7,CACNA1A,CAMK2G,FYN,GNAI2,GRK2,GRK6,HRAS,PIK3C2G,PRKACA,PRKCB,RGS16 |
| PEDF Signaling | 1.69 | -1.633 | HRAS,MAPK14,PIK3C2G,PIK3C3,PIK3CD,SERPINF1 |
| Systemic Lupus Erythematosus Signaling | 1.68 | NA | C6,CD2BP2,GRB2,HLA-A,HRAS,IL33,PIK3C2G,PIK3C3,PIK3CD,PRPF3,PRPF40B,PTPRC |
| Corticotropin Releasing Hormone Signaling | 1.68 | 0.333 | ADCY3,ADCY7,CACNA1A,GNAI2,GUCY2C,MAPK14,MEF2C,PRKACA,PRKCB |
| α-tocopherol Degradation | 1.66 | NA | CYP4A11,CYP4F12 |
| Gα12/13 Signaling | 1.66 | -1.414 | CDH1,HRAS,MAPK9,MEF2C,PIK3C2G,PIK3C3,PIK3CD,PXN |
| Synaptogenesis Signaling Pathway | 1.66 | -0.775 | ADCY3,ADCY7,ARPC1A,CAMK2G,CDH1,EPHB4,FYN,GRB2,HRAS,MAPK14,PIK3C2G,PIK3C3,PIK3CD,PRKACA,RASGRP1 |
| PD-1, PD-L1 cancer immunotherapy pathway | 1.65 | 1.134 | CBLB,HLA-A,PIK3C2G,PIK3C3,PIK3CD,RASGRP1,STAT5A |
| Phagosome Maturation | 1.65 | NA | CANX,Dync1i2,DYNC2H1,HLA-A,M6PR,PIK3C3,TCIRG1,TUBB2A,VPS39 |
| Chemokine Signaling | 1.64 | -1.633 | CAMK2G,GNAI2,HRAS,MAPK14,PIK3C2G,PRKCB |
| D-myo-inositol (1,3,4)-trisphosphate Biosynthesis | 1.62 | NA | INPP5E,SEC16A,SYNJ1 |
| BMP signaling pathway | 1.6 | -0.816 | CHRD,GRB2,HRAS,MAPK14,MAPK9,PRKACA |
| Oncostatin M Signaling | 1.59 | 1 | GRB2,HRAS,STAT3,STAT5A |
| Maturity Onset Diabetes of Young (MODY) Signaling | 1.57 | NA | CACNA1A,FABP2,PKLR |
| Pancreatic Adenocarcinoma Signaling | 1.57 | -0.378 | CDK4,GRB2,MAPK9,PIK3C2G,PIK3C3,PIK3CD,STAT3 |
| 2-ketoglutarate Dehydrogenase Complex | 1.57 | NA | DLST,OGDH |
| Acetate Conversion to Acetyl-CoA | 1.57 | NA | ACSL1,ACSS3 |
| Autophagy | 1.56 | NA | ATG13,NBR1,PIK3C3,SQSTM1,VPS39 |
| PI3K/AKT Signaling | 1.56 | 0.378 | GRB2,GSK3A,HRAS,HSP90AB1,MCL1,PIK3CD,SYNJ1,TSC1 |
| CDK5 Signaling | 1.55 | 1.134 | ADCY3,ADCY7,CACNA1A,HRAS,MAPK14,MAPK9,PRKACA |
| Type I Diabetes Mellitus Signaling | 1.55 | -1.633 | HLA-A,IL1R1,MAPK14,MAPK9,SOCS1,SOCS2,SOCS3 |
| CTLA4 Signaling in Cytotoxic T Lymphocytes | 1.53 | NA | FYN,GRB2,HLA-A,PIK3C2G,PIK3C3,PIK3CD |
| RANK Signaling in Osteoclasts | 1.53 | -0.816 | Map3k7,MAPK14,MAPK9,PIK3C2G,PIK3C3,PIK3CD |
| 1D-myo-inositol Hexakisphosphate Biosynthesis II (Mammalian) | 1.53 | NA | INPP5E,SEC16A,SYNJ1 |
| Salvage Pathways of Pyrimidine Ribonucleotides | 1.52 | 0.378 | APOBEC1,CDADC1,CDK4,DMPK,GRK6,MAPK9,PRKAA1 |
| Regulation of Cellular Mechanics by Calpain Protease | 1.51 | NA | ACTN4,CDK4,GRB2,HRAS,PXN |
| Cell Cycle: G1/S Checkpoint Regulation | 1.51 | -1 | ATR,CDK4,FBXL5,HDAC5,RBL2 |
| iCOS-iCOSL Signaling in T Helper Cells | 1.48 | -0.816 | CAMK2G,GRB2,HLA-A,PIK3C2G,PIK3C3,PIK3CD,PTPRC |
| Actin Cytoskeleton Signaling | 1.48 | 0 | ACTN4,ARPC1A,DOCK1,FLNA,GIT1,GRB2,HRAS,PIK3C2G,PIK3C3,PIK3CD,PXN |
| Remodeling of Epithelial Adherens Junctions | 1.46 | NA | ACTN4,ARPC1A,CDH1,CLIP1,TUBB2A |
| Glioblastoma Multiforme Signaling | 1.45 | -0.378 | CDK4,GRB2,HRAS,PIK3C2G,PIK3C3,PIK3CD,RHOBTB1,TCF3,TSC1 |
| GPCR-Mediated Nutrient Sensing in Enteroendocrine Cells | 1.43 | -0.378 | ADCY3,ADCY7,CACNA1A,GNAI2,PRKACA,PRKCB,RAPGEF4 |
| EIF2 Signaling | 1.43 | 0.378 | EIF2S3,EIF4G1,EIF4G3,GRB2,HRAS,PABPC1,PIK3C2G,PIK3C3,PIK3CD,RPL18A,RPL38 |
| NGF Signaling | 1.42 | -0.378 | GRB2,HRAS,Map3k7,MAPK9,PIK3C2G,PIK3C3,PIK3CD |
| Prostate Cancer Signaling | 1.41 | NA | GRB2,HRAS,HSP90AB1,PIK3C2G,PIK3C3,PIK3CD |
| Ephrin A Signaling | 1.41 | NA | FYN,PIK3C2G,PIK3C3,PIK3CD |
| Role of p14/p19ARF in Tumor Suppression | 1.41 | NA | PIK3C2G,PIK3C3,PIK3CD |
| Sphingosine-1-phosphate Signaling | 1.4 | 0 | ADCY3,ADCY7,GNAI2,PIK3C2G,PIK3C3,PIK3CD,RHOBTB1 |
| TGF-β Signaling | 1.4 | -1 | GRB2,HRAS,MAPK14,MAPK9,RNF111,TFE3 |
| Role of Macrophages, Fibroblasts and Endothelial Cells in Rheumatoid Arthritis | 1.38 | NA | CAMK2G,HRAS,IL1R1,IL33,MAPK14,MAPK9,PIK3C2G,PIK3C3,PIK3CD,PRKCB,SOCS1,SOCS3,STAT3,TCF3 |
| IL-10 Signaling | 1.37 | NA | IL1R1,IL33,MAPK14,SOCS3,STAT3 |
| fMLP Signaling in Neutrophils | 1.37 | -1.134 | ARPC1A,GNAI2,HRAS,PIK3C2G,PIK3C3,PIK3CD,PRKCB |
| Ephrin B Signaling | 1.35 | -0.447 | ABI1,EPHB4,GNAI2,HRAS,PXN |
| GP6 Signaling Pathway | 1.35 | -1.134 | COL6A3,FYN,GSK3A,PIK3C2G,PIK3C3,PIK3CD,PRKCB |
| UVA-Induced MAPK Signaling | 1.34 | -1 | HRAS,MAPK14,MAPK9,PIK3C2G,PIK3C3,PIK3CD |
| Phagosome Formation | 1.34 | NA | ITGB2,MARCO,PIK3C2G,PIK3C3,PIK3CD,PRKCB,RHOBTB1 |
| Oxidative Ethanol Degradation III | 1.33 | NA | ACSL1,ACSS3,ALDH3A2 |
| Pyridoxal 5'-phosphate Salvage Pathway | 1.33 | 1.342 | CDK4,DMPK,GRK6,MAPK9,PRKAA1 |
| Nicotine Degradation II | 1.33 | -0.447 | CYP2A6 (includes others),CYP2C18,CYP2C8,FMO1,POR |
| Estrogen Biosynthesis | 1.33 | -1 | CYP2A6 (includes others),CYP2C18,CYP2C8,POR |
| Fcγ Receptor-mediated Phagocytosis in Macrophages and Monocytes | 1.32 | -1.633 | ARPC1A,DOCK1,FYN,PIK3C2G,PRKCB,PXN |
| Neuregulin Signaling | 1.31 | -0.447 | ADAM17,GRB2,HRAS,HSP90AB1,PRKCB,STAT5A |
| Superpathway of D-myo-inositol (1,4,5)-trisphosphate Metabolism | 1.3 | NA | INPP5E,SEC16A,SYNJ1 |

**Table 4.** Significant pathways for differentially expressed transcripts in ^56^Fe vs. non-irradiated control at 9 months analyzed by IPA.

| **Ingenuity Canonical Pathways** | **-log(p-value)** | **z-score** | **Molecules** |
| --- | --- | --- | --- |
| Acute Phase Response Signaling | 4.57 | -1.265 | AKT3,C1R,IKBKB,IKBKG,JAK2,MAPK9,NFKB1,NR3C1,PIK3CD,SAA1,SOCS1,SOCS3,TCF3,TF |
| Lymphotoxin β Receptor Signaling | 3.87 | 0.816 | AKT3,BCL2L1,IKBKB,IKBKG,NFKB1,PIK3CD,TRAF3 |
| April Mediated Signaling | 3.85 | 0 | IKBKB,IKBKG,MAPK9,NFAT5,NFKB1,TRAF3 |
| B Cell Receptor Signaling | 3.74 | 0.632 | AKT3,BCL2L1,FCGR2A,IKBKB,IKBKG,MAP3K13,MAPK9,NFAT5,NFKB1,PIK3CD,PTPRC,SYNJ1,TCF3 |
| B Cell Activating Factor Signaling | 3.73 | 0 | IKBKB,IKBKG,MAPK9,NFAT5,NFKB1,TRAF3 |
| JAK/Stat Signaling | 3.59 | -0.707 | AKT3,BCL2L1,JAK2,NFKB1,PIK3CD,PTPN1,SOCS1,SOCS3 |
| NF-κB Activation by Viruses | 3.48 | 0.707 | AKT3,IKBKB,IKBKG,ITGA3,ITGAL,NFKB1,PIK3CD,PRKD3 |
| IL-17A Signaling in Airway Cells | 3.37 | -0.378 | AKT3,IKBKB,IKBKG,JAK2,MAPK9,NFKB1,PIK3CD |
| RANK Signaling in Osteoclasts | 3.28 | 0.707 | AKT3,IKBKB,IKBKG,MAP3K13,MAPK9,NFKB1,PIK3CD,XIAP |
| PI3K/AKT Signaling | 3.23 | -0.632 | AKT3,BCL2L1,IKBKB,IKBKG,ITGA3,JAK2,NFKB1,PIK3CD,SYNJ1,TSC1 |
| Type II Diabetes Mellitus Signaling | 3.21 | 0.707 | AKT3,IKBKB,IKBKG,MAPK9,NFKB1,PIK3CD,PRKAA1,PRKD3,SMPD4,SOCS1,SOCS3 |
| Role of JAK2 in Hormone-like Cytokine Signaling | 3.2 | NA | JAK2,PTPN1,SH2B1,SOCS1,SOCS3 |
| TWEAK Signaling | 3.14 | -0.447 | IKBKB,IKBKG,NFKB1,TRAF3,XIAP |
| CD27 Signaling in Lymphocytes | 3.11 | 0 | BCL2L1,IKBKB,IKBKG,MAP3K13,MAPK9,NFKB1 |
| Small Cell Lung Cancer Signaling | 3.06 | 0.378 | AKT3,BCL2L1,IKBKB,IKBKG,NFKB1,PIK3CD,TRAF3 |
| Role of Osteoblasts, Osteoclasts and Chondrocytes in Rheumatoid Arthritis | 3.03 | NA | ADAM17,AKT3,APC,AXIN1,IKBKB,IKBKG,ITGA3,MAPK9,NFAT5,NFKB1,PIK3CD,TCF3,XIAP |
| p53 Signaling | 3 | 0.378 | AKT3,BCL2L1,COQ8A,GADD45G,MDM4,PIK3CD,PML,STAG1 |
| IL-6 Signaling | 2.92 | -0.333 | AKT3,IKBKB,IKBKG,JAK2,MAPK9,NFKB1,PIK3CD,SOCS1,SOCS3 |
| Erythropoietin Signaling | 2.9 | NA | AKT3,JAK2,NFKB1,PIK3CD,PRKD3,SOCS1,SOCS3 |
| Chronic Myeloid Leukemia Signaling | 2.86 | NA | AKT3,BCL2L1,HDAC10,HDAC6,IKBKB,IKBKG,NFKB1,PIK3CD |
| PTEN Signaling | 2.84 | -0.333 | AKT3,BCL2L1,FGFR2,IKBKB,IKBKG,ITGA3,NFKB1,PIK3CD,SYNJ1 |
| Induction of Apoptosis by HIV1 | 2.79 | 0 | BCL2L1,IKBKB,IKBKG,MAPK9,NFKB1,XIAP |
| Role of JAK family kinases in IL-6-type Cytokine Signaling | 2.79 | NA | JAK2,MAPK9,SOCS1,SOCS3 |
| Activation of IRF by Cytosolic Pattern Recognition Receptors | 2.71 | -0.816 | DDX58,IKBKB,IKBKG,MAPK9,NFKB1,TRAF3 |
| Role of RIG1-like Receptors in Antiviral Innate Immunity | 2.68 | 0.447 | DDX58,IKBKB,IKBKG,NFKB1,TRAF3 |
| IL-23 Signaling Pathway | 2.68 | 0.447 | AKT3,JAK2,NFKB1,PIK3CD,SOCS3 |
| IL-4 Signaling | 2.65 | NA | AKT3,JAK2,NFAT5,NR3C1,PIK3CD,SOCS1,SYNJ1 |
| Glucocorticoid Receptor Signaling | 2.65 | NA | AKT3,BAG1,BCL2L1,ERCC3,GTF2A1,IKBKB,IKBKG,JAK2,MAPK9,NFAT5,NFKB1,NR3C1,PIK3CD,POLR2E,PRKAA1,TAF1 |
| CD40 Signaling | 2.64 | -0.816 | IKBKB,IKBKG,MAPK9,NFKB1,PIK3CD,TRAF3 |
| Production of Nitric Oxide and Reactive Oxygen Species in Macrophages | 2.6 | 0.302 | AKT3,IKBKB,IKBKG,JAK2,MAP3K13,MAPK9,NFKB1,PIK3CD,PPARA,PPP1R10,PRKD3 |
| FAT10 Cancer Signaling Pathway | 2.59 | 0.447 | ACVR1,AKT3,IKBKB,IKBKG,NFKB1 |
| Role of Macrophages, Fibroblasts and Endothelial Cells in Rheumatoid Arthritis | 2.55 | NA | AKT3,APC,AXIN1,IKBKB,IKBKG,JAK2,MAPK9,NFAT5,NFKB1,PIK3CD,PRKD3,SOCS1,SOCS3,TCF3,TRAF3 |
| TNFR2 Signaling | 2.49 | 1 | IKBKB,IKBKG,NFKB1,XIAP |
| RAR Activation | 2.48 | NA | ADCY4,AKR1C3,AKT3,ERCC3,JAK2,MAPK9,NFKB1,PIK3CD,PML,PRKD3,SDR9C7 |
| NGF Signaling | 2.44 | 0.707 | AKT3,IKBKB,IKBKG,MAP3K13,MAPK9,NFKB1,PIK3CD,SMPD4 |
| Leukocyte Extravasation Signaling | 2.43 | 0 | CLDN12,FER,ITGA3,ITGAL,MAPK9,PIK3CD,PRKD3,PXN,RAPGEF4,SIPA1,TEC |
| 4-1BB Signaling in T Lymphocytes | 2.38 | 0 | IKBKB,IKBKG,MAPK9,NFKB1 |
| Apelin Endothelial Signaling Pathway | 2.38 | 1.414 | ADCY4,AKT3,ARNT,MAPK9,NFKB1,PIK3CD,PRKAA1,PRKD3 |
| Apoptosis Signaling | 2.34 | 0.378 | AIFM1,BCL2L1,CAPNS1,IKBKB,IKBKG,NFKB1,XIAP |
| CD28 Signaling in T Helper Cells | 2.34 | 1.414 | AKT3,IKBKB,IKBKG,MAPK9,NFAT5,NFKB1,PIK3CD,PTPRC |
| Reelin Signaling in Neurons | 2.25 | NA | ARHGEF2,ITGA3,ITGAL,MAPK8IP3,MAPK9,PIK3CD |
| Mouse Embryonic Stem Cell Pluripotency | 2.24 | 1.134 | AKT3,APC,AXIN1,JAK2,PIK3CD,TCF3,XIAP |
| SAPK/JNK Signaling | 2.24 | 0 | GNB1,MAP3K13,MAP4K5,MAPK8IP3,MAPK9,MINK1,PIK3CD |
| IL-17A Signaling in Fibroblasts | 2.24 | NA | IKBKB,IKBKG,LCN2,NFKB1 |
| Gα12/13 Signaling | 2.21 | 0.707 | AKT3,IKBKB,IKBKG,MAPK9,NFKB1,PIK3CD,PXN,TEC |
| Amyotrophic Lateral Sclerosis Signaling | 2.18 | 1.633 | AKT3,BCL2L1,CAPNS1,GPX1,GRIK5,PIK3CD,XIAP |
| LPS-stimulated MAPK Signaling | 2.14 | 0 | IKBKB,IKBKG,MAPK9,NFKB1,PIK3CD,PRKD3 |
| PEDF Signaling | 2.14 | 1.633 | AKT3,BCL2L1,IKBKB,IKBKG,NFKB1,PIK3CD |
| Sertoli Cell-Sertoli Cell Junction Signaling | 2.12 | NA | AKT3,AXIN1,CLDN12,EPB41,EPN2,ITGA3,MAP3K13,MAPK9,TJP2,TJP3 |
| PPARα/RXRα Activation | 2.11 | 0.707 | ACVR1,ADCY4,CKAP5,HELZ2,IKBKB,IKBKG,JAK2,NFKB1,PPARA,PRKAA1 |
| Colorectal Cancer Metastasis Signaling | 2.1 | 0.577 | ADCY4,AKT3,APC,ARRB1,AXIN1,BCL2L1,GNB1,JAK2,MAPK9,NFKB1,PIK3CD,TCF3 |
| Prolactin Signaling | 2.09 | -0.447 | JAK2,NR3C1,PIK3CD,PRKD3,SOCS1,SOCS3 |
| T Cell Receptor Signaling | 2.09 | NA | IKBKB,IKBKG,NFAT5,NFKB1,PIK3CD,PTPRC,TEC |
| Pancreatic Adenocarcinoma Signaling | 2.07 | 0.378 | AKT3,BCL2L1,JAK2,MAPK9,NFKB1,PIK3CD,PLD2 |
| Cardiac Hypertrophy Signaling (Enhanced) | 2.07 | 1.147 | ACVR1,ADCY4,AKT3,DIAPH1,FGFR2,GNB1,HDAC10,HDAC6,IKBKB,IKBKG,ITGA3,JAK2,MAP3K13,MAPK9,NFAT5,NFKB1,PIK3CD,PRKD3,RCAN1 |
| Type I Diabetes Mellitus Signaling | 2.05 | -1.134 | IKBKB,IKBKG,JAK2,MAPK9,NFKB1,SOCS1,SOCS3 |
| HGF Signaling | 2.01 | 0.378 | AKT3,ITGA3,MAP3K13,MAPK9,PIK3CD,PRKD3,PXN |
| Role of PKR in Interferon Induction and Antiviral Response | 2 | NA | IKBKB,IKBKG,NFKB1,TRAF3 |
| IL-8 Signaling | 1.99 | 1 | AKT3,BCL2L1,GNB1,IKBKB,IKBKG,MAPK9,NFKB1,PIK3CD,PLD2,PRKD3 |
| iCOS-iCOSL Signaling in T Helper Cells | 1.97 | 1.633 | AKT3,IKBKB,IKBKG,NFAT5,NFKB1,PIK3CD,PTPRC |
| Insulin Receptor Signaling | 1.96 | -0.378 | AKT3,JAK2,PIK3CD,PPP1R10,PTPN1,SOCS3,SYNJ1,TSC1 |
| IL-22 Signaling | 1.89 | NA | AKT3,MAPK9,SOCS3 |
| Role of JAK1, JAK2 and TYK2 in Interferon Signaling | 1.89 | NA | JAK2,NFKB1,SOCS1 |
| Huntington's Disease Signaling | 1.88 | 1.134 | AKT3,BCL2L1,CAPNS1,DNM2,GNB1,HDAC10,HDAC6,MAPK9,PIK3CD,POLR2E,PRKD3 |
| IL-15 Production | 1.88 | NA | AATK,DYRK1A,FER,FGFR2,JAK2,NFKB1,TEC |
| IL-1 Signaling | 1.87 | 0 | ADCY4,GNB1,IKBKB,IKBKG,MAPK9,NFKB1 |
| Apelin Pancreas Signaling Pathway | 1.85 | 1 | MAPK9,NFKB1,PIK3CD,PRKAA1 |
| Role of NFAT in Regulation of the Immune Response | 1.78 | 2.121 | AKT3,FCGR2A,GNB1,IKBKB,IKBKG,NFAT5,NFKB1,PIK3CD,RCAN1 |
| IL-15 Signaling | 1.77 | NA | AKT3,BCL2L1,JAK2,NFKB1,PIK3CD |
| iNOS Signaling | 1.76 | 0 | IKBKB,IKBKG,JAK2,NFKB1 |
| IL-10 Signaling | 1.75 | NA | FCGR2A,IKBKB,IKBKG,NFKB1,SOCS3 |
| Fcγ Receptor-mediated Phagocytosis in Macrophages and Monocytes | 1.74 | 1.633 | AKT3,FCGR2A,PIP5K1A,PLD2,PRKD3,PXN |
| Sirtuin Signaling Pathway | 1.72 | -0.632 | ADAM10,ATG13,CYC1,GADD45G,GLUD1,LDHA,NDUFAF1,NDUFV1,NFKB1,NR1H2,PPARA,PRKAA1,SIRT7 |
| HOTAIR Regulatory Pathway | 1.71 | 0 | AEBP2,AKT3,NFKB1,PIK3CD,SETDB1,STK38,TCF3,XIAP |
| Regulation of the Epithelial-Mesenchymal Transition Pathway | 1.7 | NA | ADAM17,AKT3,APC,AXIN1,FGFR2,JAK2,NFKB1,PIK3CD,TCF3 |
| Caveolar-mediated Endocytosis Signaling | 1.7 | NA | DNM2,FLOT1,ITGA3,ITGAL,PTPN1 |
| Growth Hormone Signaling | 1.7 | -0.447 | JAK2,PIK3CD,PRKD3,SOCS1,SOCS3 |
| TNFR1 Signaling | 1.7 | 1 | IKBKB,IKBKG,NFKB1,XIAP |
| Assembly of RNA Polymerase II Complex | 1.7 | NA | ERCC3,GTF2A1,POLR2E,TAF1 |
| Molecular Mechanisms of Cancer | 1.68 | NA | ADCY4,AKT3,APC,ARHGEF2,AXIN1,BCL2L1,CDK19,ITGA3,JAK2,MAPK9,NFKB1,PIK3CD,PRKD3,TCF3,XIAP |
| Phenylalanine Degradation I (Aerobic) | 1.68 | NA | PCBD2,QDPR |
| Angiopoietin Signaling | 1.66 | 1 | AKT3,IKBKB,IKBKG,NFKB1,PIK3CD |
| Gαq Signaling | 1.65 | 2.121 | AKT3,GNB1,IKBKB,IKBKG,NFKB1,PIK3CD,PLD2,PRKD3 |
| Adipogenesis pathway | 1.64 | NA | ERCC3,FGFR2,HDAC10,HDAC6,PPIP5K1,SETDB1,TBL1XR1 |
| Leptin Signaling in Obesity | 1.64 | 0 | ADCY4,AKT3,JAK2,PIK3CD,SOCS3 |
| Phospholipase C Signaling | 1.63 | 1.667 | ADCY4,ARHGEF2,FCGR2A,GNB1,HDAC10,HDAC6,ITGA3,NFAT5,NFKB1,PLD2,PRKD3 |
| IGF-1 Signaling | 1.63 | -0.816 | AKT3,JAK2,PIK3CD,PXN,SOCS1,SOCS3 |
| Hepatic Cholestasis | 1.63 | NA | ADCY4,IKBKB,IKBKG,MAPK9,NFKB1,PPARA,PPRC1,PRKD3,TJP2 |
| IL-12 Signaling and Production in Macrophages | 1.63 | NA | AKT3,IKBKB,IKBKG,MAPK9,NFKB1,PIK3CD,PRKD3 |
| Tec Kinase Signaling | 1.63 | 0.378 | GNB1,ITGA3,JAK2,MAPK9,NFKB1,PIK3CD,PRKD3,TEC |
| Agrin Interactions at Neuromuscular Junction | 1.61 | -1.342 | AGRN,ITGA3,ITGAL,MAPK9,PXN |
| FXR/RXR Activation | 1.61 | NA | AKT3,FETUB,LIPC,MAPK9,PPARA,SAA1,TF |
| Tight Junction Signaling | 1.61 | NA | AKT3,ARHGEF2,CASK,CLDN12,EPB41,NFKB1,TJP2,TJP3 |
| IL-17 Signaling | 1.6 | NA | AKT3,JAK2,MAPK9,NFKB1,PIK3CD |
| Sumoylation Pathway | 1.58 | NA | MAPK9,NFKB1,NR3C1,PML,RNF4,XIAP |
| Human Embryonic Stem Cell Pluripotency | 1.57 | NA | ACVR1,AKT3,APC,AXIN1,FGFR2,PIK3CD,TCF3 |
| tRNA Charging | 1.55 | 1.342 | FARSA,IARS,LARS2,Qars,VARS |
| PI3K Signaling in B Lymphocytes | 1.52 | 1.89 | AKT3,IKBKB,IKBKG,NFAT5,NFKB1,PIK3CD,PTPRC |
| Germ Cell-Sertoli Cell Junction Signaling | 1.52 | NA | AXIN1,EPN2,FER,ITGA3,MAP3K13,MAPK9,PIK3CD,PXN |
| Virus Entry via Endocytic Pathways | 1.52 | NA | AP1G2,DNM2,ITGA3,ITGAL,PIK3CD,PRKD3 |
| Antioxidant Action of Vitamin C | 1.52 | 0 | IKBKB,IKBKG,JAK2,MAPK9,NFKB1,PLD2 |
| IL-9 Signaling | 1.51 | NA | NFKB1,PIK3CD,SOCS3 |
| mTOR Signaling | 1.43 | 0.378 | AKT3,ATG13,EIF4G1,PIK3CD,PLD2,PRKAA1,PRKD3,RPS19,TSC1 |
| MIF-mediated Glucocorticoid Regulation | 1.42 | NA | CD74,NFKB1,NR3C1 |
| Dendritic Cell Maturation | 1.41 | 0.707 | AKT3,FCGR2A,IKBKB,IKBKG,JAK2,MAPK9,NFKB1,PIK3CD |
| TR/RXR Activation | 1.39 | NA | AKR1C3,AKT3,COL6A3,PIK3CD,TBL1XR1 |
| Regulation of IL-2 Expression in Activated and Anergic T Lymphocytes | 1.39 | NA | IKBKB,IKBKG,MAPK9,NFAT5,NFKB1 |
| Role of NFAT in Cardiac Hypertrophy | 1.38 | 1.89 | ADCY4,AKT3,GNB1,HDAC10,HDAC6,MAPK9,PIK3CD,PRKD3,RCAN1 |
| Role of Tissue Factor in Cancer | 1.38 | NA | AKT3,ARRB1,BCL2L1,ITGA3,JAK2,PIK3CD |
| Acute Myeloid Leukemia Signaling | 1.37 | 2.236 | AKT3,NFKB1,PIK3CD,PML,TCF3 |
| Factors Promoting Cardiogenesis in Vertebrates | 1.35 | NA | ACVR1,APC,AXIN1,PRKD3,TCF3 |
| GP6 Signaling Pathway | 1.35 | 1.633 | ADAM10,AKT3,COL4A5,COL6A3,PIK3CD,PRKD3 |
| Renin-Angiotensin Signaling | 1.31 | 0.447 | ADCY4,JAK2,MAPK9,NFKB1,PIK3CD,PRKD3 |
| LPS/IL-1 Mediated Inhibition of RXR Function | 1.3 | 0 | CYP2A6 (includes others),CYP4A11,FMO1,FMO2,LIPC,MAPK9,NR1H2,PPARA,PPARGC1B |

**Table 5.** Significant pathways for differentially expressed transcripts in ^56^Fe vs. non-irradiated control at 12 months analyzed by IPA.

| **^56^Fe, 1 month** | | **^56^Fe, 2 months** | | **^56^Fe, 4 months** | | **^56^Fe, 9 months** | | **^56^Fe, 12 months** | |
| --- | --- | --- | --- | --- | --- | --- | --- | --- | --- |
| **Module** | **Transcript Ensemble ID** | **Module** | **Transcript Ensemble ID** | **Module** | **Transcript Ensemble ID** | **Module** | **Transcript Ensemble ID** | **Module** | **Transcript Ensemble ID** |
| 10 | ENSMUST00000237602 | 13 | ENSMUST00000237025 | 19 | ENSMUST00000236950 | 18 | ENSMUST00000235957 | 15 | ENSMUST00000236171 |
| 15 | ENSMUST00000237742 | 15 | ENSMUST00000236006 | 25 | ENSMUST00000237098 | 29 | ENSMUST00000236217 | 22 | ENSMUST00000236392 |
| 16 | ENSMUST00000236727 | 19 | ENSMUST00000236824 | 29 | ENSMUST00000235915 | 35 | ENSMUST00000235927 | 28 | ENSMUST00000236217 |
| 17 | ENSMUST00000236267 | 23 | ENSMUST00000236209 | 29 | ENSMUST00000236006 | 37 | ENSMUST00000236006 | 29 | ENSMUST00000235411 |
| 19 | ENSMUST00000236403 | 28 | ENSMUST00000236925 | 30 | ENSMUST00000235764 | 40 | ENSMUST00000237358 | 2 | ENSMUST00000235318 |
| 20 | ENSMUST00000238288 | 2 | ENSMUST00000235947 | 34 | ENSMUST00000237854 | 40 | ENSMUST00000237862 | 32 | ENSMUST00000235947 |
| 27 | ENSMUST00000237170 | 30 | ENSMUST00000236873 | 38 | ENSMUST00000235411 | 45 | ENSMUST00000236547 | 34 | ENSMUST00000237874 |
| 2 | ENSMUST00000238201 | 30 | ENSMUST00000237472 | 40 | ENSMUST00000237874 | 49 | ENSMUST00000238729 | 35 | ENSMUST00000235620 |
| 2 | ENSMUST00000236186 | 30 | ENSMUST00000237823 | 48 | ENSMUST00000236209 |  |  | 35 | ENSMUST00000236330 |
| 30 | ENSMUST00000237167 | 33 | ENSMUST00000237749 | 48 | ENSMUST00000236046 |  |  | 42 | ENSMUST00000235135 |
| 45 | ENSMUST00000236268 | 37 | ENSMUST00000235620 | 48 | ENSMUST00000235207 |  |  | 42 | ENSMUST00000238368 |
| 45 | ENSMUST00000238271 | 38 | ENSMUST00000238677 | 48 | ENSMUST00000236850 |  |  | 44 | ENSMUST00000238729 |
| 48 | ENSMUST00000237472 | 3 | ENSMUST00000235318 | 48 | ENSMUST00000236687 |  |  | 44 | ENSMUST00000236006 |
| 4 | ENSMUST00000235304 | 42 | ENSMUST00000237742 |  |  |  |  | 46 | ENSMUST00000238731 |
| 4 | ENSMUST00000235231 | 6 | ENSMUST00000236480 |  |  |  |  |  |  |
| 9 | ENSMUST00000235929 | 8 | ENSMUST00000238176 |  |  |  |  |  |  |

**Table 6.** Unannotated differentially expressed transcripts (by IPA) in ^56^Fe experiments at all time points. Each unannotated ENSEMBLE transcript ID is listed with the corresponding module number in the SOM Figure 2.

| **Ingenuity Canonical Pathways** | **-log(p-value)** | **z-score** | **Molecules** |
| --- | --- | --- | --- |
| LXR/RXR Activation | 7.8 | 2.53 | ACACA,APOA2,C4A/C4B,C9,CD36,CLU,FASN,FDFT1,HMGCR,KNG1,MLXIPL,NCOR2,NR1H3,PLTP,SAA1,SCD,SERPINF1 |
| FXR/RXR Activation | 5.86 | NA | APOA2,C4A/C4B,C9,CLU,FASN,FETUB,KNG1,MLXIPL,NR0B2,NR1H3,PLTP,SAA1,SCARB1,SDC1,SERPINF1 |
| PPARα/RXRα Activation | 4.01 | 0.577 | APOA2,CD36,CKAP5,Cyp2c70,CYP2C8,FASN,GPD2,GRB2,IKBKB,IKBKG,MED23,NCOR2,NR0B2,PLCE1,TGFBR1 |
| Mevalonate Pathway I | 3.35 | -1.342 | ACAT2,HADHB,HMGCR,HMGCS1,MVD |
| Role of PKR in Interferon Induction and Antiviral Response | 3.34 | NA | ATF2,CASP8,IKBKB,IKBKG,STAT1,TRAF3 |
| Nicotine Degradation II | 3.31 | 0 | CYP2A6 (includes others),CYP2C8,CYP3A5,FMO1,FMO2,FMO4,INMT,UGT2B28 |
| Adipogenesis pathway | 3.31 | NA | CEBPA,CTNNB1,EZH2,FGFR2,FGFR3,HDAC10,LPIN1,NOCT,PPIP5K1,RPS6KA1,SMAD5 |
| LPS/IL-1 Mediated Inhibition of RXR Function | 3.26 | 0.333 | ALAS1,CYP2A6 (includes others),CYP2C8,CYP3A5,CYP4A11,Cyp4a14,FMO1,FMO2,FMO4,HMGCS1,NR0B2,NR1H3,NR1I3,PLTP,SCARB1 |
| 3-phosphoinositide Degradation | 3.18 | -0.577 | CA3,INPP4A,MTMR3,PPIP5K1,Ppp1cc,PPP1R16B,PTPA,PTPRF,PTPRJ,SET,SIRPA,SYNJ1 |
| Superpathway of Inositol Phosphate Compounds | 2.9 | 0 | CA3,PI4K2B,PIP5K1A,PLCE1,PPIP5K1,Ppp1cc,PPP1R16B,PTPA,PTPRF,PTPRJ,SEC16A,SET,SIRPA,SYNJ1 |
| 3-phosphoinositide Biosynthesis | 2.82 | 0 | CA3,PI4K2B,PIP5K1A,PPIP5K1,Ppp1cc,PPP1R16B,PTPA,PTPRF,PTPRJ,SET,SIRPA,SYNJ1 |
| Superpathway of Geranylgeranyldiphosphate Biosynthesis I (via Mevalonate) | 2.82 | -1.342 | ACAT2,HADHB,HMGCR,HMGCS1,MVD |
| Epithelial Adherens Junction Signaling | 2.81 | NA | ACTN4,BAIAP2,CLIP1,CTNNB1,EPN2,MYH10,PARD3,TCF3,TGFBR1,TUBA4A,TUBB2A |
| PXR/RXR Activation | 2.8 | NA | ALAS1,CYP2A6 (includes others),CYP2C8,CYP3A5,NR0B2,NR1I3,SCD |
| Ephrin B Signaling | 2.66 | 0 | ABI1,AXIN1,CAP1,CTNNB1,EPHB4,GNB2,HNRNPK |
| D-myo-inositol-5-phosphate Metabolism | 2.64 | -0.302 | CA3,PLCE1,PPIP5K1,Ppp1cc,PPP1R16B,PTPA,PTPRF,PTPRJ,SET,SIRPA,SYNJ1 |
| Inhibition of Angiogenesis by TSP1 | 2.6 | NA | CD36,CD47,HSPG2,SDC1,TGFBR1 |
| D-myo-inositol (1,4,5,6)-Tetrakisphosphate Biosynthesis | 2.45 | 0 | CA3,PPIP5K1,Ppp1cc,PPP1R16B,PTPA,PTPRF,PTPRJ,SET,SIRPA,SYNJ1 |
| D-myo-inositol (3,4,5,6)-tetrakisphosphate Biosynthesis | 2.45 | 0 | CA3,PPIP5K1,Ppp1cc,PPP1R16B,PTPA,PTPRF,PTPRJ,SET,SIRPA,SYNJ1 |
| RAR Activation | 2.39 | NA | AKR1C4,CARM1,CYP26A1,DHRS4,NCOR2,PBRM1,PML,PRKD3,RARB,SDR9C7,SMAD5,TNIP1 |
| Role of RIG1-like Receptors in Antiviral Innate Immunity | 2.37 | 0.447 | CASP8,IKBKB,IKBKG,TRAF3,TRIM25 |
| Superpathway of Cholesterol Biosynthesis | 2.32 | -1.89 | ACAT2,FDFT1,HADHB,HMGCR,HMGCS1,MVD,TM7SF2 |
| Acute Phase Response Signaling | 2.29 | -1.342 | APOA2,C1R,C4A/C4B,C9,GRB2,HNRNPK,IKBKB,IKBKG,SAA1,SERPINF1,TCF3 |
| HIPPO signaling | 2.29 | 0 | DLG1,ITCH,MOB1A,PARD3,Ppp1cc,PTPA,SMAD5 |
| Regulation of IL-2 Expression in Activated and Anergic T Lymphocytes | 2.16 | NA | Calm1 (includes others),GRB2,IKBKB,IKBKG,NFATC1,PPP3CB,TGFBR1 |
| B Cell Receptor Signaling | 2.14 | 0 | ATF2,BCL6,Calm1 (includes others),GRB2,IKBKB,IKBKG,MAP3K13,NFATC1,PPP3CB,SYNJ1,TCF3 |
| Phosphatidylcholine Biosynthesis I | 2.12 | NA | CHPT1,PCYT1A,PHKA1 |
| Factors Promoting Cardiogenesis in Vertebrates | 2.11 | NA | ATF2,AXIN1,CTNNB1,PRKD3,SMAD5,TCF3,TGFBR1 |
| Sertoli Cell-Sertoli Cell Junction Signaling | 2.07 | NA | ACTN4,ATF2,AXIN1,CTNNB1,DLG1,EPN2,MAP3K13,SPTAN1,TJP3,TUBA4A,TUBB2A |
| Cardiac Hypertrophy Signaling | 2.06 | -0.905 | ADRA1B,ATF2,Calm1 (includes others),EIF2B4,GNB2,GRB2,MAP3K13,MEF2A,PLCE1,PPP3CB,RHOBTB1,RPS6KA1,TGFBR1 |
| TWEAK Signaling | 1.99 | -1 | CASP8,IKBKB,IKBKG,TRAF3 |
| Ketogenesis | 1.98 | NA | ACAT2,HADHB,HMGCS1 |
| Pregnenolone Biosynthesis | 1.91 | NA | CYP26A1,CYP26B1,CYP4A11 |
| Cell Cycle Regulation by BTG Family Proteins | 1.91 | NA | E2F3,E2F5,NOCT,PTPA |
| Acetone Degradation I (to Methylglyoxal) | 1.91 | -1 | CYP2A6 (includes others),CYP2C8,CYP3A5,CYP4A11 |
| Breast Cancer Regulation by Stathmin1 | 1.89 | NA | ARHGEF12,Calm1 (includes others),E2F3,E2F5,GNB2,GRB2,PRKD3,PTPA,TUBA4A,TUBB2A,UHMK1 |
| Chronic Myeloid Leukemia Signaling | 1.88 | NA | E2F3,E2F5,GRB2,HDAC10,IKBKB,IKBKG,TGFBR1 |
| SAPK/JNK Signaling | 1.88 | 0 | ATF2,GRB2,HNRNPK,MAP3K13,MAPK8IP3,MINK1,NFATC1 |
| PTEN Signaling | 1.87 | 0 | BCL2L11,FGFR2,FGFR3,GRB2,IKBKB,IKBKG,SYNJ1,TGFBR1 |
| Complement System | 1.87 | NA | C1R,C4A/C4B,C8A,C9 |
| April Mediated Signaling | 1.83 | -1 | IKBKB,IKBKG,NFATC1,TRAF3 |
| B Cell Activating Factor Signaling | 1.76 | -1 | IKBKB,IKBKG,NFATC1,TRAF3 |
| Activation of IRF by Cytosolic Pattern Recognition Receptors | 1.72 | 0.447 | ATF2,IKBKB,IKBKG,STAT1,TRAF3 |
| α-tocopherol Degradation | 1.72 | NA | CYP4A11,CYP4F3 |
| Choline Biosynthesis III | 1.7 | NA | CHPT1,PCYT1A,PHKA1 |
| Gαq Signaling | 1.69 | -1.134 | ADRA1B,Calm1 (includes others),GNB2,IKBKB,IKBKG,NFATC1,PPP3CB,PRKD3,RHOBTB1 |
| Production of Nitric Oxide and Reactive Oxygen Species in Macrophages | 1.67 | 1 | APOA2,CLU,IKBKB,IKBKG,MAP3K13,PRKD3,PTPA,RHOBTB1,SIRPA,STAT1 |
| RANK Signaling in Osteoclasts | 1.66 | 0 | Calm1 (includes others),IKBKB,IKBKG,MAP3K13,NFATC1,PPP3CB |
| Histidine Degradation VI | 1.65 | NA | CYP26A1,CYP26B1,CYP4A11 |
| Tetrahydrofolate Salvage from 5,10-methenyltetrahydrofolate | 1.63 | NA | GART,MTHFD1L |
| Glioblastoma Multiforme Signaling | 1.61 | -0.447 | AXIN1,CTNNB1,E2F3,E2F5,GRB2,PLCE1,RHOBTB1,TCF3,TSC1 |
| Protein Kinase A Signaling | 1.6 | -1.069 | ATF2,Calm1 (includes others),CTNNB1,EYA3,GNB2,MTMR3,MYH10,NFATC1,PLCE1,Ppp1cc,PPP3CB,PRKD3,PTPRF,PTPRJ,SIRPA,TCF3,TGFBR1 |
| Role of Macrophages, Fibroblasts and Endothelial Cells in Rheumatoid Arthritis | 1.59 | NA | ATF2,AXIN1,Calm1 (includes others),CEBPA,CTNNB1,IKBKB,IKBKG,NFATC1,PLCE1,PPP3CB,PRKD3,PROK1,TCF3,TRAF3 |
| FAT10 Cancer Signaling Pathway | 1.59 | 0 | CTNNB1,IKBKB,IKBKG,TGFBR1 |
| Insulin Receptor Signaling | 1.58 | 0 | ACLY,EIF2B4,GRB2,Ppp1cc,PTPRF,SYNJ1,TRIP10,TSC1 |
| Remodeling of Epithelial Adherens Junctions | 1.57 | NA | ACTN4,CLIP1,CTNNB1,TUBA4A,TUBB2A |
| Xenobiotic Metabolism Signaling | 1.57 | NA | ARNT,CYP2C8,CYP3A5,ESD,FMO1,FMO2,FMO4,MAP3K13,NCOR2,NR1I3,PRKD3,PTPA,UGT2B28 |
| Bupropion Degradation | 1.56 | NA | CYP2A6 (includes others),CYP2C8,CYP3A5 |
| NGF Signaling | 1.55 | 0.378 | ATF2,GRB2,IKBKB,IKBKG,MAP3K13,RPS6KA1,SMPD4 |
| Phenylalanine Degradation I (Aerobic) | 1.55 | NA | PCBD2,QDPR |
| Germ Cell-Sertoli Cell Junction Signaling | 1.54 | NA | ACTN4,AXIN1,CTNNB1,EPN2,MAP3K13,RHOBTB1,TGFBR1,TUBA4A,TUBB2A |
| IL-15 Production | 1.53 | NA | AXL,EPHB4,FGFR2,FGFR3,KIT,STAT1,TNK2 |
| iNOS Signaling | 1.53 | NA | Calm1 (includes others),IKBKB,IKBKG,STAT1 |
| TGF-β Signaling | 1.52 | NA | GRB2,RNF111,SMAD5,TFE3,TGFBR1,ZFYVE9 |
| Phospholipase C Signaling | 1.51 | -1.265 | ARHGEF12,ATF2,Calm1 (includes others),GNB2,GRB2,HDAC10,MEF2A,NFATC1,PLCE1,PPP3CB,PRKD3,RHOBTB1 |
| NF-κB Signaling | 1.49 | -1 | AZI2,CASP8,FGFR2,FGFR3,IKBKB,IKBKG,TGFBR1,TNIP1,TRAF3 |
| TNFR1 Signaling | 1.47 | 0 | CASP8,IKBKB,IKBKG,MADD |
| Apoptosis Signaling | 1.46 | 1.633 | BCL2L11,CASP8,IKBKB,IKBKG,RPS6KA1,SPTAN1 |
| Estrogen Biosynthesis | 1.42 | 0 | AKR1C4,CYP2A6 (includes others),CYP2C8,CYP3A5 |
| 14-3-3-mediated Signaling | 1.4 | -1.342 | GRB2,PLCE1,PRKD3,RPS6KA1,TSC1,TUBA4A,TUBB2A |
| Integrin Signaling | 1.4 | -0.333 | ACTN4,ARF5,ASAP1,GRB2,ITGA7,ITGAL,ITGAV,RHOBTB1,TLN2,TNK2 |
| CD27 Signaling in Lymphocytes | 1.39 | 0 | CASP8,IKBKB,IKBKG,MAP3K13 |
| mTOR Signaling | 1.39 | -0.447 | DGKZ,EIF4G3,MT-RNR1,MT-RNR2,PRKD3,PROK1,PTPA,RHOBTB1,RPS6KA1,TSC1 |
| Triacylglycerol Biosynthesis | 1.39 | 0 | AGPAT3,LPIN1,PLPP5,TAZ |
| Mouse Embryonic Stem Cell Pluripotency | 1.39 | 0 | AXIN1,CTNNB1,GRB2,LIFR,SMAD5,TCF3 |
| Role of NFAT in Regulation of the Immune Response | 1.39 | -0.707 | ATF2,Calm1 (includes others),GNB2,GRB2,IKBKB,IKBKG,MEF2A,NFATC1,PPP3CB |
| PPAR Signaling | 1.37 | 0.816 | GRB2,IKBKB,IKBKG,NCOR2,NR0B2,NR1H3 |
| 4-1BB Signaling in T Lymphocytes | 1.37 | NA | ATF2,IKBKB,IKBKG |
| Ubiquinol-10 Biosynthesis (Eukaryotic) | 1.37 | NA | CYP26A1,CYP26B1,CYP4A11 |
| Unfolded protein response | 1.32 | NA | CEBPA,INSIG1,P4HB,SYVN1 |
| Cyclins and Cell Cycle Regulation | 1.32 | NA | E2F3,E2F5,FBXL5,HDAC10,PTPA |
| Role of JAK2 in Hormone-like Cytokine Signaling | 1.3 | NA | SH2B2,SIRPA,STAT1 |

**Table 7.** Significant pathways for differentially expressed transcripts in ^16^O vs. non-irradiated control at 1 month analyzed by IPA.

| **Ingenuity Canonical Pathways** | **-log(p-value)** | **z-score** | **Molecules** |
| --- | --- | --- | --- |
| Adipogenesis pathway | 4.55 | NA | ATG7,CTNNB1,EZH2,FGFR2,HDAC10,HDAC5,LPIN1,NOCT,PPIP5K1,SAP130,SIN3A,TBL1XR1 |
| Aryl Hydrocarbon Receptor Signaling | 3.5 | -0.707 | ALDH2,ARNT,ATR,CDKN1B,GSTM4,HSP90AB1,MGST1,NCOR2,NFIA,NFKB1,RXRB |
| Xenobiotic Metabolism Signaling | 2.89 | NA | ALDH2,ANKRA2,ARNT,GSTM4,HDAC5,HSP90AB1,KEAP1,MAP3K13,Map3k7,MAPK9,MGST1,NCOR2,NFKB1,NR1I3,PRKD3 |
| Huntington's Disease Signaling | 2.71 | 0 | ATF2,ATP5PB,DNM2,EGFR,GNB2,HDAC10,HDAC5,MAPK9,NCOR1,NCOR2,PRKD3,SGK1,SIN3A |
| Mitochondrial Dysfunction | 2.67 | NA | ATP5MC2,ATP5PB,CYB5A,CYC1,MAPK9,MT-ATP6,MT-ND4L,NCSTN,NDUFAF1,PINK1,TXNRD2 |
| Cell Cycle: G1/S Checkpoint Regulation | 2.55 | 0 | ATR,CDKN1B,FBXL5,HDAC10,HDAC5,SIN3A |
| IL-15 Production | 2.4 | NA | CLK2,EGFR,EPHB4,FES,FGFR2,NFKB1,TEK,TNK2 |
| UVC-Induced MAPK Signaling | 2.38 | 1 | ATR,EGFR,MAPK9,PRKD3,SMPD2 |
| Apelin Endothelial Signaling Pathway | 2.35 | -2.121 | ARNT,Calm1 (includes others),HDAC5,MAPK9,MEF2A,NFKB1,PRKD3,TEK |
| LXR/RXR Activation | 2.25 | 0 | APOE,C4A/C4B,CD36,NCOR1,NCOR2,NFKB1,RXRB,TLR3 |
| Protein Kinase A Signaling | 2.19 | -1.667 | AKAP1,AKAP13,ANAPC5,ATF2,Calm1 (includes others),CTNNB1,EYA3,FLNA,GNB2,NAPEPLD,NFKB1,PHKA2,PRKD3,PTPRC,SIRPA,TCF3,TTN |
| Cyclins and Cell Cycle Regulation | 2.15 | NA | ATR,CDKN1B,FBXL5,HDAC10,HDAC5,SIN3A |
| Pancreatic Adenocarcinoma Signaling | 2.05 | 0.447 | CDKN1B,EGFR,MAPK9,NAPEPLD,NFKB1,PROK1,SIN3A |
| Cardiac Hypertrophy Signaling (Enhanced) | 2.03 | -0.229 | ADRA1B,AKAP13,ATF2,Calm1 (includes others),CTNNB1,DIAPH1,FGFR2,HDAC10,HDAC5,ITGA3,MAP3K13,Map3k7,MAPK9,MEF2A,MKNK1,NAPEPLD,NFKB1,PKN1,PRKD3 |
| Phospholipase C Signaling | 1.98 | -1.897 | ARHGEF2,ATF2,Calm1 (includes others),GNB2,HDAC10,HDAC5,ITGA3,MEF2A,MPRIP,NAPEPLD,NFKB1,PRKD3 |
| RANK Signaling in Osteoclasts | 1.95 | -0.816 | Calm1 (includes others),MAP3K13,Map3k7,MAPK9,NFKB1,XIAP |
| α-tocopherol Degradation | 1.84 | NA | CYP4F12,CYP4F3 |
| Prostate Cancer Signaling | 1.83 | NA | ATF2,CDKN1B,CTNNB1,HSP90AB1,NFKB1,SIN3A |
| IL-17A Signaling in Gastric Cells | 1.83 | NA | EGFR,MAPK9,NFKB1 |
| mTOR Signaling | 1.79 | -1 | EIF4A2,EIF4G1,NAPEPLD,PRKD3,PROK1,RPS11,RPS13,RPS14,RPTOR,TSC1 |
| ATM Signaling | 1.79 | 0 | ATF2,ATR,HERC2,MAPK9,MDM4,SMC2 |
| RhoA Signaling | 1.79 | -0.378 | ACTR3,ARHGAP12,BAIAP2,MPRIP,PKN1,RAPGEF6,TTN |
| Epithelial Adherens Junction Signaling | 1.78 | NA | ACTR3,BAIAP2,CTNNB1,EGFR,KEAP1,MYH14,SSX2IP,TCF3 |
| PTEN Signaling | 1.74 | -0.378 | BCAR1,CDKN1B,EGFR,FGFR2,INPP5D,ITGA3,NFKB1 |
| NRF2-mediated Oxidative Stress Response | 1.72 | 0.447 | DNAJB11,DNAJC1,EPHX1,GSTM4,HERPUD1,KEAP1,MAPK9,MGST1,PRKD3 |
| B Cell Receptor Signaling | 1.72 | -1.134 | ATF2,Calm1 (includes others),INPP5D,MAP3K13,Map3k7,MAPK9,NFKB1,PTPRC,TCF3 |
| Actin Cytoskeleton Signaling | 1.71 | 0 | ACTR3,BAIAP2,BCAR1,DIAPH1,FLNA,FN1,ITGA3,MPRIP,MYH14,TTN |
| Sirtuin Signaling Pathway | 1.69 | -1.667 | ACLY,ATG7,ATP5PB,CYC1,HSF1,MT-ATP6,MT-ND4L,NDUFAF1,NFKB1,PFKFB3,PGK1,POLR3D,RPTOR |
| Regulation of eIF4 and p70S6K Signaling | 1.69 | NA | EIF2S2,EIF4A2,EIF4G1,ITGA3,MKNK1,RPS11,RPS13,RPS14 |
| HOTAIR Regulatory Pathway | 1.69 | -0.707 | ATG7,CTNNB1,EZH2,HSF1,JARID2,NFKB1,TCF3,XIAP |
| Caveolar-mediated Endocytosis Signaling | 1.69 | NA | DNM2,EGFR,FLNA,FLOT2,ITGA3 |
| Pyridoxal 5'-phosphate Salvage Pathway | 1.69 | 1.342 | DMPK,GRK6,MAPK9,PKN1,SGK1 |
| Sertoli Cell-Sertoli Cell Junction Signaling | 1.67 | NA | ATF2,BCAR1,CTNNB1,EPB41,ITGA3,KEAP1,MAP3K13,Map3k7,MAPK9 |
| EIF2 Signaling | 1.66 | 0 | EIF2S2,EIF4A2,EIF4G1,NOX4,RPL10A,RPL19,RPS11,RPS13,RPS14,XIAP |
| Reelin Signaling in Neurons | 1.62 | NA | APOE,ARHGEF2,ITGA3,MAPK8IP3,MAPK9 |
| TNFR2 Signaling | 1.61 | NA | NFKB1,TBK1,XIAP |
| CD27 Signaling in Lymphocytes | 1.6 | 0 | MAP3K13,Map3k7,MAPK9,NFKB1 |
| VDR/RXR Activation | 1.6 | 0 | CDKN1B,NCOR1,NCOR2,PRKD3,RXRB |
| PI3K/AKT Signaling | 1.59 | 1.134 | CDKN1B,CTNNB1,HSP90AB1,INPP5D,ITGA3,NFKB1,TSC1 |
| RAR Activation | 1.58 | NA | AKR1C4,MAPK9,NCOR1,NCOR2,NFKB1,PBRM1,PRKD3,RXRB,TNIP1 |
| Cdc42 Signaling | 1.58 | 0 | ACTR3,ATF2,BAIAP2,DIAPH1,ITGA3,MAPK9,MPRIP,TNK2 |
| IL-8 Signaling | 1.55 | -0.378 | EGFR,GNB2,MAPK9,NAPEPLD,NFKB1,NOX4,PRKD3,PROK1,TEK |
| 4-1BB Signaling in T Lymphocytes | 1.54 | NA | ATF2,MAPK9,NFKB1 |
| Assembly of RNA Polymerase III Complex | 1.53 | NA | GTF3C4,POLR3D |
| HGF Signaling | 1.48 | 0 | ATF2,ITGA3,MAP3K13,Map3k7,MAPK9,PRKD3 |
| Salvage Pathways of Pyrimidine Ribonucleotides | 1.48 | 0.816 | AK4,DMPK,GRK6,MAPK9,PKN1,SGK1 |
| GNRH Signaling | 1.45 | 0.378 | ATF2,Calm1 (includes others),EGFR,MAP3K13,Map3k7,MAPK9,NFKB1,PRKD3 |
| Induction of Apoptosis by HIV1 | 1.41 | 1 | MAPK9,NFKB1,SLC25A3,XIAP |
| Endocannabinoid Cancer Inhibition Pathway | 1.41 | 0.378 | ATF2,CDKN1B,CTNNB1,PROK1,RPTOR,SMPD2,TCF3 |
| Oxidative Phosphorylation | 1.4 | -0.816 | ATP5MC2,ATP5PB,CYB5A,CYC1,MT-ATP6,MT-ND4L |
| Ephrin Receptor Signaling | 1.4 | 0 | ABI1,ACTR3,ATF2,BCAR1,EPHB4,GNB2,ITGA3,PROK1 |
| NGF Signaling | 1.39 | 0 | ATF2,MAP3K13,Map3k7,MAPK9,NFKB1,SMPD2 |
| TR/RXR Activation | 1.37 | NA | NCOR1,NCOR2,RCAN2,RXRB,TBL1XR1 |
| Activation of IRF by Cytosolic Pattern Recognition Receptors | 1.37 | 0 | ATF2,MAPK9,NFKB1,TBK1 |
| fMLP Signaling in Neutrophils | 1.35 | -2.236 | ACTR3,Calm1 (includes others),GNB2,NFKB1,NOX4,PRKD3 |
| Factors Promoting Cardiogenesis in Vertebrates | 1.34 | NA | ATF2,CTNNB1,NOX4,PRKD3,TCF3 |
| Colorectal Cancer Metastasis Signaling | 1.33 | 1.414 | ARRB1,CTNNB1,EGFR,GNB2,MAPK9,MSH3,NFKB1,PROK1,TCF3,TLR3 |
| Cholecystokinin/Gastrin-mediated Signaling | 1.33 | 0 | ATF2,BCAR1,EGFR,MAPK9,MEF2A,PRKD3 |
| Autophagy | 1.32 | NA | ATG7,CTSS,NBR1,VPS33B |
| Inhibition of Angiogenesis by TSP1 | 1.32 | NA | CD36,MAPK9,SDC1 |
| Phosphatidylcholine Biosynthesis I | 1.32 | NA | CHKA,PHKA1 |

**Table 8.** Significant pathways for differentially expressed transcripts in ^16^O vs. non-irradiated control at 2 months analyzed by IPA.

| **Ingenuity Canonical Pathways** | **-log(p-value)** | **z-score** | **Molecules** |
| --- | --- | --- | --- |
| Acute Phase Response Signaling | 5.06 | 0.378 | AHSG,C1R,C2,FN1,IKBKB,IKBKG,IL1R1,ITIH2,ITIH3,MAP2K3,NR3C1,SAA1,SOCS2,TCF3,TF |
| TR/RXR Activation | 4.6 | NA | F10,FASN,G6PC,MDM2,ME1,PIK3C2G,RXRB,SREBF1,TBL1XR1,THRSP |
| PPARα/RXRα Activation | 4.08 | 0 | ADCY6,ADCY9,AIP,CKAP5,FASN,HELZ2,HSP90AA1,IKBKB,IKBKG,IL1R1,MAP2K3,MAP4K4,SLC27A1,TGFBR1 |
| Role of RIG1-like Receptors in Antiviral Innate Immunity | 3.48 | 0.816 | CASP8,IKBKB,IKBKG,TBK1,TRAF3,TRIM25 |
| LXR/RXR Activation | 3.36 | 0.378 | AHSG,APOE,FASN,IL1R1,NR1H2,RXRB,SAA1,SCD,SREBF1,TF |
| Role of JAK2 in Hormone-like Cytokine Signaling | 3.14 | NA | SH2B1,SH2B3,SIRPA,SOCS2,STAT5A |
| FXR/RXR Activation | 3.13 | NA | AHSG,APOE,FASN,G6PC,LIPC,NR5A2,PKLR,SAA1,SREBF1,TF |
| Role of PKR in Interferon Induction and Antiviral Response | 2.76 | NA | CASP8,IKBKB,IKBKG,MAP2K3,TRAF3 |
| PPAR Signaling | 2.75 | 0 | AIP,HSP90AA1,IKBKB,IKBKG,IL1R1,MAP4K4,PDGFRA,STAT5A |
| Epithelial Adherens Junction Signaling | 2.72 | NA | AFDN,CLIP1,JUP,KEAP1,MET,MYH10,MYH11,PARD3,TCF3,TGFBR1 |
| B Cell Receptor Signaling | 2.57 | 0.707 | FCGR2A,IKBKB,IKBKG,INPP5K,MAP2K3,Map3k7,NFAT5,PIK3C2G,PTPRC,SYNJ1,TCF3 |
| Type I Diabetes Mellitus Signaling | 2.55 | -0.378 | CASP8,HLA-A,IFNGR1,IKBKB,IKBKG,IL1R1,MAP2K3,SOCS2 |
| Death Receptor Signaling | 2.47 | -0.378 | CASP8,IKBKB,IKBKG,LMNA,MAP4K4,SPTAN1,TBK1 |
| Acute Myeloid Leukemia Signaling | 2.44 | -0.816 | CEBPA,JUP,MAP2K3,PIK3C2G,PML,STAT5A,TCF3 |
| PXR/RXR Activation | 2.42 | NA | ALAS1,G6PC,GSTM1,NR1I3,NR3C1,SCD |
| IL-1 Signaling | 2.39 | 0 | ADCY6,ADCY9,GNB1,IKBKB,IKBKG,IL1R1,MAP2K3 |
| Cardiac β-adrenergic Signaling | 2.33 | -0.378 | ADCY6,ADCY9,AKAP13,ATP2A3,GNB1,PDE1A,PDE4B,PPP1R10,PPP1R3C |
| IL-10 Signaling | 2.33 | NA | FCGR2A,IKBKB,IKBKG,IL1R1,MAP2K3,MAP4K4 |
| NF-κB Signaling | 2.27 | 0 | CASP8,IKBKB,IKBKG,IL1R1,MAP4K4,PDGFRA,PIK3C2G,TBK1,TGFBR1,TRAF3 |
| Apoptosis Signaling | 2.27 | -0.816 | CAPNS1,CASP8,IKBKB,IKBKG,LMNA,MAP4K4,SPTAN1 |
| CD27 Signaling in Lymphocytes | 2.27 | 0.447 | CASP8,IKBKB,IKBKG,MAP2K3,Map3k7 |
| TWEAK Signaling | 2.19 | -1 | CASP8,IKBKB,IKBKG,TRAF3 |
| Production of Nitric Oxide and Reactive Oxygen Species in Macrophages | 2.04 | 0 | APOE,IFNGR1,IKBKB,IKBKG,Map3k7,PIK3C2G,PPP1R10,PPP1R3C,RHOT1,SIRPA |
| April Mediated Signaling | 2.03 | 1 | IKBKB,IKBKG,NFAT5,TRAF3 |
| LPS/IL-1 Mediated Inhibition of RXR Function | 1.97 | -1.134 | ALAS1,APOE,FMO1,GSTM1,IL1R1,LIPC,NR1H2,NR1I3,NR5A2,SLC27A1,SREBF1 |
| IL-4 Signaling | 1.96 | NA | HLA-A,INPP5K,NFAT5,NR3C1,PIK3C2G,SYNJ1 |
| Activation of IRF by Cytosolic Pattern Recognition Receptors | 1.95 | 0.447 | IKBKB,IKBKG,STAT2,TBK1,TRAF3 |
| B Cell Activating Factor Signaling | 1.95 | 1 | IKBKB,IKBKG,NFAT5,TRAF3 |
| Cellular Effects of Sildenafil (Viagra) | 1.91 | NA | ADCY6,ADCY9,GUCY2C,MPRIP,MYH10,MYH11,PDE1A,PDE4B |
| CD40 Signaling | 1.9 | -0.447 | IKBKB,IKBKG,MAP2K3,PIK3C2G,TRAF3 |
| Insulin Receptor Signaling | 1.89 | 1.134 | ACLY,INPP5K,PIK3C2G,PPP1R10,PPP1R3C,PTPRF,SYNJ1,TSC1 |
| Sphingosine-1-phosphate Signaling | 1.82 | 1.134 | ADCY6,ADCY9,CASP8,PDGFRA,PIK3C2G,RHOT1,SMPD4 |
| Protein Kinase A Signaling | 1.79 | 0.535 | ADCY6,ADCY9,AKAP13,GNB1,MYH10,NFAT5,PDE1A,PDE4B,PPP1R10,PPP1R3C,PTPN21,PTPRC,PTPRF,SIRPA,TCF3,TGFBR1 |
| Role of Oct4 in Mammalian Embryonic Stem Cell Pluripotency | 1.78 | NA | JARID2,NR5A2,RXRB,TDRD7 |
| p53 Signaling | 1.73 | 0.447 | ATR,MDM2,MDM4,PIK3C2G,PML,STAG1 |
| PTEN Signaling | 1.69 | -1.134 | IKBKB,IKBKG,INPP5K,MAST2,PDGFRA,SYNJ1,TGFBR1 |
| Osteoarthritis Pathway | 1.69 | -0.632 | ACVRL1,CASP8,FN1,IL1R1,LRP1,MTF1,PTH1R,SIK3,TCF3,TGFBR1 |
| Cell Cycle: G2/M DNA Damage Checkpoint Regulation | 1.69 | 0 | ATR,FBXL5,MDM2,MDM4 |
| Small Cell Lung Cancer Signaling | 1.68 | 1 | IKBKB,IKBKG,PIK3C2G,RXRB,TRAF3 |
| Relaxin Signaling | 1.67 | 0.816 | ADCY6,ADCY9,GNB1,GUCY2C,NPR2,PDE1A,PDE4B,PIK3C2G |
| NRF2-mediated Oxidative Stress Response | 1.67 | NA | DNAJB12,DNAJC1,DNAJC5,DNAJC8,FMO1,GSTM1,KEAP1,MAP2K3,PIK3C2G |
| Hepatic Fibrosis / Hepatic Stellate Cell Activation | 1.67 | NA | COL4A1,FN1,IFNGR1,IL1R1,MET,MYH10,MYH11,PDGFRA,TGFBR1 |
| TNFR1 Signaling | 1.66 | 0 | CASP8,IKBKB,IKBKG,MADD |
| Phenylalanine Degradation I (Aerobic) | 1.66 | NA | PCBD2,QDPR |
| Growth Hormone Signaling | 1.65 | 2.236 | CEBPA,PIK3C2G,RPS6KC1,SOCS2,STAT5A |
| HOTAIR Regulatory Pathway | 1.64 | 0 | DNMT3B,JARID2,MDM2,MET,PIK3C2G,SETDB1,STK38,TCF3 |
| Chronic Myeloid Leukemia Signaling | 1.63 | NA | IKBKB,IKBKG,MDM2,PIK3C2G,STAT5A,TGFBR1 |
| Clathrin-mediated Endocytosis Signaling | 1.59 | NA | APOE,CD2AP,MDM2,MET,PICALM,PIK3C2G,PIP5K1C,SYNJ1,TF |
| TNFR2 Signaling | 1.59 | NA | IKBKB,IKBKG,TBK1 |
| CDP-diacylglycerol Biosynthesis I | 1.55 | NA | AGPAT3,CDS2,GPAM |
| PI3K/AKT Signaling | 1.55 | 1.134 | HSP90AA1,IKBKB,IKBKG,INPP5K,MDM2,SYNJ1,TSC1 |
| Tight Junction Signaling | 1.55 | NA | AFDN,CEBPA,CPSF1,MYH10,MYH11,PARD6A,SPTAN1,TGFBR1 |
| Systemic Lupus Erythematosus Signaling | 1.53 | NA | FCGR2A,HLA-A,HNRNPA2B1,HNRNPC,NFAT5,PIK3C2G,PRPF38B,PRPF40B,PRPF6,PTPRC |
| Sumoylation Pathway | 1.53 | -2 | CEBPA,MDM2,NR3C1,PML,RFC1,RHOT1 |
| Lymphotoxin β Receptor Signaling | 1.52 | 1 | IKBKB,IKBKG,PIK3C2G,TRAF3 |
| Assembly of RNA Polymerase III Complex | 1.52 | NA | BDP1,GTF3C1 |
| BER pathway | 1.52 | NA | LIG1,LIG3 |
| PEDF Signaling | 1.51 | 1.342 | CASP8,IKBKB,IKBKG,PIK3C2G,TCF12 |
| IL-9 Signaling | 1.48 | NA | PIK3C2G,SOCS2,STAT5A |
| HER-2 Signaling in Breast Cancer | 1.47 | NA | MDM2,PARD3,PARD6A,PIK3C2G,TSC1 |
| Germ Cell-Sertoli Cell Junction Signaling | 1.46 | NA | AFDN,JUP,KEAP1,MAP2K3,Map3k7,PIK3C2G,RHOT1,TGFBR1 |
| HIPPO signaling | 1.43 | NA | PARD3,PPP1R10,PPP1R3C,WWTR1,YAP1 |
| IL-17A Signaling in Fibroblasts | 1.42 | NA | IKBKB,IKBKG,LCN2 |
| iCOS-iCOSL Signaling in T Helper Cells | 1.41 | 2.236 | HLA-A,IKBKB,IKBKG,NFAT5,PIK3C2G,PTPRC |
| Systemic Lupus Erythematosus In B Cell Signaling Pathway | 1.41 | -0.302 | Eda,FCGR2A,IFNGR1,INPP5K,MAP4K4,NFAT5,PIK3C2G,STAT2,SYNJ1,TBK1,TRAF3 |
| Telomere Extension by Telomerase | 1.4 | NA | HNRNPA2B1,TERF2 |
| Cardiac Hypertrophy Signaling (Enhanced) | 1.39 | 0.5 | ADCY6,ADCY9,AKAP13,ATP2A3,Eda,GNB1,IKBKB,IKBKG,IL1R1,MAP2K3,Map3k7,MKNK1,NFAT5,PDE1A,PDE4B,PIK3C2G,TGFBR1 |
| Aryl Hydrocarbon Receptor Signaling | 1.38 | 0.816 | AIP,ATR,GSTM1,HSP90AA1,MDM2,NFIA,RXRB |
| Induction of Apoptosis by HIV1 | 1.38 | -1 | CASP8,IKBKB,IKBKG,SLC25A3 |
| Role of Macrophages, Fibroblasts and Endothelial Cells in Rheumatoid Arthritis | 1.38 | NA | CEBPA,CSNK1A1,FN1,IKBKB,IKBKG,IL1R1,LRP1,MAP2K3,NFAT5,PIK3C2G,TCF3,TRAF3 |
| Endocannabinoid Cancer Inhibition Pathway | 1.37 | -0.378 | ADCY6,ADCY9,CASP8,MAP2K3,PIK3C2G,SMPD4,TCF3 |
| Phosphatidylglycerol Biosynthesis II (Non-plastidic) | 1.36 | NA | AGPAT3,CDS2,GPAM |
| NGF Signaling | 1.35 | 1.633 | IKBKB,IKBKG,Map3k7,PIK3C2G,RPS6KC1,SMPD4 |
| Iron homeostasis signaling pathway | 1.34 | NA | ATP6AP1,FBXL5,LRP1,NUBP2,PDGFRA,STAT5A,TF |
| Dopamine Receptor Signaling | 1.34 | NA | ADCY6,ADCY9,PPP1R10,PPP1R3C,QDPR |
| Role of NFAT in Regulation of the Immune Response | 1.32 | 2.646 | CSNK1A1,FCGR2A,GNB1,HLA-A,IKBKB,IKBKG,NFAT5,PIK3C2G |
| Phosphatidylcholine Biosynthesis I | 1.3 | NA | CHKA,PHKA1 |

**Table 9.** Significant pathways for differentially expressed transcripts in ^16^O vs. non-irradiated control at 4 months analyzed by IPA.

| **Ingenuity Canonical Pathways** | **-log(p-value)** | **z-score** | **Molecules** |
| --- | --- | --- | --- |
| LXR/RXR Activation | 5.4 | 1 | ACACA,C4A/C4B,CD36,FASN,ITIH4,LPL,PLTP,PON3,SAA1,SCD,SERPINA1,TF,TLR3 |
| PXR/RXR Activation | 4.73 | NA | ALAS1,ALDH3A2,CES3,CYP2A6 (includes others),CYP2C8,NR0B2,PRKACA,SCD,SLCO1B3 |
| AMPK Signaling | 4.4 | 1.604 | ACACA,ACACB,ADRA2B,ARID1A,CHRNA4,FASN,PFKFB3,PFKM,PIK3C2G,PIK3CA,PPM1B,PRKAA1,PRKACA,RAB6A,STK11,TSC2 |
| FXR/RXR Activation | 3.72 | NA | C4A/C4B,FASN,ITIH4,LPL,NR0B2,PLTP,PON3,SAA1,SERPINA1,SLCO1B3,TF |
| Role of JAK2 in Hormone-like Cytokine Signaling | 3.13 | NA | GHR,SIRPA,SOCS2,STAT5A,TYK2 |
| TR/RXR Activation | 3.11 | NA | ACACA,FASN,MDM2,ME1,NCOA4,PIK3C2G,PIK3CA,THRSP |
| PPARα/RXRα Activation | 2.96 | 0.905 | ADCY3,CD36,CKAP5,CYP2C8,FASN,GHR,LPL,MAP4K4,MEF2C,NR0B2,PRKAA1,PRKACA |
| Growth Hormone Signaling | 2.94 | -0.378 | CEBPA,GHR,PIK3C2G,PIK3CA,RPS6KA1,SOCS2,STAT5A |
| LPS/IL-1 Mediated Inhibition of RXR Function | 2.84 | 0.447 | ACSL1,ALAS1,ALDH3A2,Cyp2a12/Cyp2a22,CYP2A6 (includes others),CYP2C8,CYP4A11,FMO1,GSTP1,NR0B2,PLTP,SLCO1B3,XPO1 |
| Stearate Biosynthesis I (Animals) | 2.78 | -0.816 | ACOT1,ACSL1,CYP4A11,ELOVL1,ELOVL6,FASN |
| Chronic Myeloid Leukemia Signaling | 2.76 | NA | CDK4,CDKN1B,HDAC5,MDM2,PIK3C2G,PIK3CA,RBL2,STAT5A |
| Retinol Biosynthesis | 2.33 | 0.447 | CES3,ESD,LPL,PNPLA3,PNPLA5 |
| Melanoma Signaling | 2.29 | 1 | CDH1,CDK4,MDM2,PIK3C2G,PIK3CA |
| IL-9 Signaling | 2.28 | NA | PIK3C2G,PIK3CA,SOCS2,STAT5A |
| Triacylglycerol Biosynthesis | 2.26 | -2 | AGPAT3,ELOVL1,ELOVL6,PLPP2,PNPLA3 |
| IL-7 Signaling Pathway | 2.12 | -0.447 | BCL6,CDKN1B,MET,PIK3C2G,PIK3CA,STAT5A |
| Triacylglycerol Degradation | 2.09 | 0.447 | CES3,LPL,PNPLA3,PNPLA5,TARS2 |
| B Cell Receptor Signaling | 2.08 | -1.265 | BCL6,CAMK2D,CAMK2G,GAB1,Map3k7,MEF2C,PIK3C2G,PIK3CA,SYK,SYNJ1 |
| Complement System | 2.06 | NA | C4A/C4B,C6,C8G,CFH |
| Adipogenesis pathway | 2.05 | NA | CEBPA,GTF2H1,HDAC5,Kat6b,KAT7,LPL,RBBP7,RPS6KA1 |
| CNTF Signaling | 2.03 | 0.447 | LIFR,PIK3C2G,PIK3CA,RPS6KA1,TYK2 |
| HER-2 Signaling in Breast Cancer | 2.02 | NA | CDKN1B,MDM2,PARD3,PIK3C2G,PIK3CA,TSC2 |
| Xenobiotic Metabolism Signaling | 1.98 | NA | ALDH3A2,CAMK2D,CAMK2G,CES3,CYP2C8,ESD,FMO1,GSTP1,HDAC5,Map3k7,PIK3C2G,PIK3CA,UGT2B28 |
| HGF Signaling | 1.93 | 0 | DOCK1,ELF2,GAB1,Map3k7,MET,PIK3C2G,PIK3CA |
| cAMP-mediated signaling | 1.93 | -1.667 | ADCY3,ADRA2B,AKAP12,AKAP13,CAMK2D,CAMK2G,CREM,LPAR1,PDE5A,PRKACA,RPS6KA1 |
| RAR Activation | 1.92 | NA | ADCY3,ARID1A,CARM1,GTF2H1,PIK3CA,PML,PRKACA,RDH16,STAT5A,TAF4 |
| Systemic Lupus Erythematosus Signaling | 1.89 | NA | C6,C8G,CD2BP2,CD72,CREM,HLA-A,HNRNPA2B1,PIK3C2G,PIK3CA,PRPF3,PRPF40B |
| ErbB2-ErbB3 Signaling | 1.89 | 1 | CDKN1B,PIK3C2G,PIK3CA,STAT5A,TYK2 |
| Insulin Receptor Signaling | 1.88 | -1.414 | ACLY,GAB1,PIK3C2G,PIK3CA,PPP1R10,PRKACA,SYNJ1,TSC2 |
| Glioma Signaling | 1.86 | -1 | CAMK2D,CAMK2G,CDK4,MDM2,PIK3C2G,PIK3CA,RBL2 |
| Breast Cancer Regulation by Stathmin1 | 1.85 | NA | ADCY3,CAMK2D,CAMK2G,CDKN1B,GNB1,PIK3C2G,PIK3CA,PPP1R10,PRKACA,UHMK1 |
| Cell Cycle: G1/S Checkpoint Regulation | 1.84 | -0.447 | CDK4,CDKN1B,HDAC5,MDM2,RBL2 |
| α-tocopherol Degradation | 1.82 | NA | CYP4A11,CYP4F12 |
| Aryl Hydrocarbon Receptor Signaling | 1.81 | 0 | ALDH3A2,CDK4,CDKN1B,GSTP1,MDM2,NEDD8,NR0B2,RBL2 |
| Apelin Pancreas Signaling Pathway | 1.8 | 0 | PIK3C2G,PIK3CA,PRKAA1,PRKACA |
| GM-CSF Signaling | 1.74 | -0.447 | CAMK2D,CAMK2G,PIK3C2G,PIK3CA,RACK1 |
| Acetate Conversion to Acetyl-CoA | 1.73 | NA | ACSL1,ACSS3 |
| p53 Signaling | 1.72 | 0.447 | CDK4,COQ8A,MDM2,PIK3C2G,PIK3CA,PML |
| FAK Signaling | 1.72 | NA | ASAP1,CAPNS1,DOCK1,PIK3C2G,PIK3CA,TNS1 |
| IL-15 Signaling | 1.71 | NA | PIK3C2G,PIK3CA,STAT5A,SYK,TYK2 |
| Role of p14/p19ARF in Tumor Suppression | 1.62 | NA | MDM2,PIK3C2G,PIK3CA |
| Sirtuin Signaling Pathway | 1.61 | 0.905 | ACLY,ATG13,ATP5F1B,ATP5PF,CDH1,MT-ATP6,NDUFA10,NDUFA4,PFKFB3,PFKM,PRKAA1,SIRT4,STK11 |
| Biotin-carboxyl Carrier Protein Assembly | 1.58 | NA | ACACA,ACACB |
| PD-1, PD-L1 cancer immunotherapy pathway | 1.55 | 0 | CDKN1B,HLA-A,PIK3C2G,PIK3CA,STAT5A,TYK2 |
| CDP-diacylglycerol Biosynthesis I | 1.55 | NA | AGPAT3,CDS2,PNPLA3 |
| Oxidative Ethanol Degradation III | 1.55 | NA | ACSL1,ACSS3,ALDH3A2 |
| PI3K/AKT Signaling | 1.54 | 0.816 | CDKN1B,GAB1,MDM2,PIK3CA,SYNJ1,TSC2,TYK2 |
| JAK/Stat Signaling | 1.54 | 0.447 | PIK3C2G,PIK3CA,SOCS2,STAT5A,TYK2 |
| Antiproliferative Role of Somatostatin Receptor 2 | 1.52 | -1 | CDKN1B,GNB1,GUCY2C,PIK3C2G,PIK3CA |
| Apelin Adipocyte Signaling Pathway | 1.52 | 1.342 | ADCY3,GPX1,GSTP1,PRKAA1,PRKACA |
| Pancreatic Adenocarcinoma Signaling | 1.48 | -0.447 | CDK4,CDKN1B,MDM2,PIK3C2G,PIK3CA,TYK2 |
| eNOS Signaling | 1.48 | -0.816 | ADCY3,AQP8,CHRNA4,LPAR1,PIK3C2G,PIK3CA,PRKAA1,PRKACA |
| Nitric Oxide Signaling in the Cardiovascular System | 1.47 | 1.633 | GUCY2C,PDE5A,PIK3C2G,PIK3CA,PRKAA1,PRKACA |
| Ethanol Degradation IV | 1.44 | NA | ACSL1,ACSS3,ALDH3A2 |
| HIPPO signaling | 1.42 | 1 | AMOT,ITCH,PARD3,PPP1R10,WWTR1 |
| Endocannabinoid Developing Neuron Pathway | 1.4 | -0.447 | ADCY3,CDKN1B,GNB1,PIK3C2G,PIK3CA,PRKACA |
| IL-4 Signaling | 1.4 | NA | HLA-A,PIK3C2G,PIK3CA,SYNJ1,TYK2 |
| Cardiac β-adrenergic Signaling | 1.39 | -1 | ADCY3,AKAP12,AKAP13,GNB1,PDE5A,PPP1R10,PRKACA |
| Acute Phase Response Signaling | 1.37 | NA | C4A/C4B,ITIH3,ITIH4,PIK3CA,SAA1,SERPINA1,SOCS2,TF |
| Oxidative Phosphorylation | 1.36 | 1.633 | ATP5F1B,ATP5PF,MT-ATP6,NDUFA10,NDUFA4,SURF1 |
| Endocannabinoid Cancer Inhibition Pathway | 1.36 | 1.134 | ADCY3,CDH1,CDKN1B,PIK3C2G,PIK3CA,PRKAA1,PRKACA |
| mTOR Signaling | 1.35 | -1.89 | ATG13,EIF4G1,EIF4G3,PIK3C2G,PIK3CA,PRKAA1,RPS6KA1,STK11,TSC2 |
| Phosphatidylglycerol Biosynthesis II (Non-plastidic) | 1.35 | NA | AGPAT3,CDS2,PNPLA3 |
| Acetone Degradation I (to Methylglyoxal) | 1.35 | NA | CYP2A6 (includes others),CYP2C8,CYP4A11 |
| IL-2 Signaling | 1.33 | 0 | PIK3C2G,PIK3CA,STAT5A,SYK |
| Acute Myeloid Leukemia Signaling | 1.32 | -0.447 | CEBPA,PIK3C2G,PIK3CA,PML,STAT5A |
| Apelin Endothelial Signaling Pathway | 1.3 | 0.816 | ADCY3,HDAC5,MEF2C,PIK3C2G,PIK3CA,PRKAA1 |
| Role of NFAT in Cardiac Hypertrophy | 1.3 | -0.707 | ADCY3,CAMK2D,CAMK2G,GNB1,HDAC5,MEF2C,PIK3C2G,PIK3CA,PRKACA |

**Table 10.** Significant pathways for differentially expressed transcripts in ^16^O vs. non-irradiated control at 9 months analyzed by IPA.

| **Ingenuity Canonical Pathways** | **-log(p-value)** | **z-score** | **Molecules** |
| --- | --- | --- | --- |
| Acute Phase Response Signaling | 16.5 | 2.2 | A2M,AKT3,APOA2,CP,FGA,FN1,HMOX1,HP,HPX,IKBKG,IL18,IL1R1,IL1RN,IL33,ITIH3,ITIH4,JAK2,JUN,LBP,MAP2K6,MAPK9,NFKB1,OSMR,PIK3CD,SAA1,Saa3,SERPINA3,SERPINE1,SOCS1,SOCS3,TCF3,TF |
| IL-6 Signaling | 8.05 | 1.5 | A2M,AKT3,CD14,IKBKG,IL18,IL1R1,IL1RN,IL33,JAK2,JUN,LBP,MAP2K6,MAPK9,NFKB1,PIK3C3,PIK3CD,SOCS1,SOCS3 |
| LXR/RXR Activation | 7.1 | -0.5 | APOA2,CD14,FGA,HPX,IL18,IL1R1,IL1RN,IL33,ITIH4,LBP,NCOR1,NFKB1,NR1H2,NR1H4,RXRA,SAA1,TF |
| IL-10 Signaling | 7.09 | NA | CD14,FCGR2A,HMOX1,IKBKG,IL18,IL1R1,IL1RN,IL33,JUN,LBP,MAP2K6,NFKB1,SOCS3 |
| FXR/RXR Activation | 5.95 | NA | ABCB11,AKT3,APOA2,FETUB,FGA,HPX,IL18,IL1RN,IL33,ITIH4,LIPC,MAPK9,NR1H4,RXRA,SAA1,TF |
| Toll-like Receptor Signaling | 5.94 | 0.632 | CD14,IKBKG,IL18,IL1RN,IL33,IRAK2,JUN,LBP,MAP2K6,NFKB1,TLR2,TNFAIP3 |
| Role of Macrophages, Fibroblasts and Endothelial Cells in Rheumatoid Arthritis | 5.09 | NA | AKT3,ATF2,AXIN1,CEBPD,FN1,IKBKG,IL17RA,IL18,IL1R1,IL1RN,IL33,IRAK2,JAK2,JUN,MAP2K6,MAPK9,NFAT5,NFKB1,PIK3C3,PIK3CD,SOCS1,SOCS3,TCF3,TLR2 |
| JAK/Stat Signaling | 4.96 | 0.302 | AKT3,BCL2L1,CDKN1A,JAK2,JUN,NFKB1,PIK3C3,PIK3CD,PTPN1,SOCS1,SOCS3 |
| Adipogenesis pathway | 4.69 | NA | CEBPD,ERCC3,EZH2,FGFR2,FGFR3,HDAC3,HDAC6,HDAC7,KDM1A,KMT2B,PPIP5K1,SAP130,SETDB1,SOX9 |
| Hepatic Cholestasis | 4.45 | NA | ABCB11,ADCY1,ADCY4,CD14,IKBKG,IL18,IL1R1,IL1RN,IL33,IRAK2,JUN,LBP,MAPK9,NFKB1,NR1H4,PPRC1,RXRA |
| IL-17 Signaling | 4.2 | NA | AKT3,ATF2,IL17RA,JAK2,JUN,MAP2K6,MAPK9,NFKB1,PIK3C3,PIK3CD |
| Iron homeostasis signaling pathway | 4.12 | NA | ARNT,ATP6AP1,ATP6V0A4,CP,FBXL5,HMOX1,HP,HPX,JAK2,SLC11A2,SLC39A14,TCIRG1,TF,TFR2 |
| LPS-stimulated MAPK Signaling | 4.1 | 1.667 | ATF2,CD14,IKBKG,JUN,LBP,MAP2K6,MAPK9,NFKB1,PIK3C3,PIK3CD |
| Chronic Myeloid Leukemia Signaling | 3.92 | NA | AKT3,BCL2L1,CDKN1A,CDKN1B,HDAC3,HDAC6,HDAC7,IKBKG,NFKB1,PIK3C3,PIK3CD |
| Osteoarthritis Pathway | 3.91 | 0.5 | ACVRL1,ATF2,DDIT4,DDR2,FGFR3,FN1,H19,HDAC3,IL1R1,MTF1,MYBBP1A,NFKB1,PRKAA1,SLC39A8,SOX9,TCF3,TLR2 |
| NF-κB Signaling | 3.9 | 0.775 | AKT3,FGFR2,FGFR3,IKBKG,IL18,IL1R1,IL1RN,IL33,MAP2K6,NFKB1,PIK3C3,PIK3CD,TBK1,TLR2,TNFAIP3 |
| TNFR2 Signaling | 3.86 | 1.342 | IKBKG,JUN,NFKB1,TBK1,TNFAIP3,XIAP |
| RANK Signaling in Osteoclasts | 3.8 | 1.897 | AKT3,IKBKG,JUN,MAP2K6,MAP3K13,MAPK9,NFKB1,PIK3C3,PIK3CD,XIAP |
| Dendritic Cell Maturation | 3.77 | 2.324 | AKT3,ATF2,DDR2,FCGR2A,HLA-A,IKBKG,IL18,IL1RN,IL33,JAK2,MAPK9,NFKB1,PIK3C3,PIK3CD,TLR2 |
| TR/RXR Activation | 3.72 | NA | AKT3,COL6A3,FGA,HDAC3,HP,NCOR1,PIK3C3,PIK3CD,RXRA,SYT12 |
| Glucocorticoid Receptor Signaling | 3.69 | NA | A2M,AKT3,BAG1,BCL2L1,CDKN1A,ERCC3,FKBP5,IKBKG,IL1RN,JAK2,JUN,MAPK9,NCOR1,NFAT5,NFKB1,PIK3C3,PIK3CD,PRKAA1,SERPINE1,SMARCA2,SMARCA4,TAF1 |
| B Cell Receptor Signaling | 3.62 | 1.941 | AKT3,ATF2,BCL2L1,FCGR2A,IKBKG,JUN,MAP2K6,MAP3K13,MAPK9,NFAT5,NFKB1,PIK3C3,PIK3CD,SYNJ1,TCF3 |
| IL-1 Signaling | 3.6 | 1.134 | ADCY1,ADCY4,GNB1,IKBKG,IL1R1,IRAK2,JUN,MAP2K6,MAPK9,NFKB1 |
| Activation of IRF by Cytosolic Pattern Recognition Receptors | 3.51 | 0.707 | ATF2,DDX58,IKBKG,JUN,MAPK9,MAVS,NFKB1,TBK1 |
| iNOS Signaling | 3.51 | 0.816 | CD14,IKBKG,IRAK2,JAK2,JUN,LBP,NFKB1 |
| IL-17A Signaling in Fibroblasts | 3.47 | NA | CEBPD,IKBKG,IL17RA,JUN,LCN2,NFKB1 |
| CD40 Signaling | 3.42 | 0.707 | IKBKG,JUN,MAP2K6,MAPK9,NFKB1,PIK3C3,PIK3CD,TNFAIP3 |
| IL-17A Signaling in Airway Cells | 3.37 | 1.414 | AKT3,IKBKG,IL17RA,JAK2,MAPK9,NFKB1,PIK3C3,PIK3CD |
| RAR Activation | 3.35 | NA | ADCY1,ADCY4,AKT3,CSF2RB,ERCC3,JAK2,JUN,MAPK9,NCOR1,NFKB1,PIK3CD,RXRA,SMARCA2,SMARCA4,ZBTB16 |
| IL-15 Production | 3.31 | NA | AATK,CLK4,DDR2,EPHB4,FGFR2,FGFR3,JAK2,MAP2K6,NFKB1,ROR1,TEC |
| HOTAIR Regulatory Pathway | 3.3 | -0.832 | AKT3,CDKN1A,DNMT3B,EZH2,HSF1,KDM1A,KMT2A,NFKB1,PIK3C3,PIK3CD,SETDB1,TCF3,XIAP |
| Role of JAK family kinases in IL-6-type Cytokine Signaling | 3.3 | NA | JAK2,MAPK9,OSMR,SOCS1,SOCS3 |
| Role of Osteoblasts, Osteoclasts and Chondrocytes in Rheumatoid Arthritis | 3.29 | NA | AKT3,AXIN1,IKBKG,IL18,IL1R1,IL1RN,IL33,JUN,MAP2K6,MAPK9,NFAT5,NFKB1,PIK3C3,PIK3CD,TCF3,XIAP |
| SAPK/JNK Signaling | 3.28 | 1.667 | ATF2,DUSP8,GNB1,JUN,MAP3K13,MAPK8IP3,MAPK9,MINK1,PIK3C3,PIK3CD |
| Hereditary Breast Cancer Signaling | 3.27 | NA | AKT3,CDKN1A,GADD45G,HDAC3,HDAC6,HDAC7,MSH2,PIK3C3,PIK3CD,RAD50,SMARCA2,SMARCA4 |
| CD27 Signaling in Lymphocytes | 3.24 | 1.633 | BCL2L1,IKBKG,JUN,MAP2K6,MAP3K13,MAPK9,NFKB1 |
| LPS/IL-1 Mediated Inhibition of RXR Function | 3.22 | 1.155 | ABCB11,ABCC4,CD14,IL18,IL1R1,IL1RN,IL33,JUN,LBP,LIPC,MAPK9,NR1H2,NR1H4,PPARGC1B,RXRA,SLC27A1 |
| Cardiac Hypertrophy Signaling (Enhanced) | 3.2 | 1.961 | ADCY1,ADCY4,AKT3,ATF2,CSF2RB,DIAPH1,FGFR2,FGFR3,GNB1,HDAC3,HDAC6,HDAC7,IKBKG,IL17RA,IL18,IL1R1,IL33,JAK2,JUN,MAP2K6,MAP3K13,MAPK9,NFAT5,NFKB1,PIK3C3,PIK3CD,RCAN1 |
| PTEN Signaling | 3.1 | 0.302 | AKT3,BCL2L1,CDKN1A,CDKN1B,FGFR2,FGFR3,IKBKG,MAST2,NFKB1,PIK3CD,SYNJ1 |
| Role of IL-17A in Arthritis | 3.1 | NA | ATF2,IL17RA,MAP2K6,MAPK9,NFKB1,PIK3C3,PIK3CD |
| Small Cell Lung Cancer Signaling | 3.04 | 1.89 | AKT3,BCL2L1,CDKN1B,IKBKG,NFKB1,PIK3C3,PIK3CD,RXRA |
| Pancreatic Adenocarcinoma Signaling | 3.03 | 2.333 | AKT3,BCL2L1,CDKN1A,CDKN1B,HMOX1,JAK2,MAPK9,NFKB1,PIK3C3,PIK3CD |
| PPARα/RXRα Activation | 3.01 | 0.277 | ADCY1,ADCY4,APOA2,CKAP5,IKBKG,IL1R1,JAK2,JUN,MAP2K6,NCOR1,NFKB1,PRKAA1,RXRA,SLC27A1 |
| IL-23 Signaling Pathway | 2.92 | 0.816 | AKT3,JAK2,NFKB1,PIK3C3,PIK3CD,SOCS3 |
| IL-12 Signaling and Production in Macrophages | 2.89 | NA | AKT3,APOA2,IKBKG,IL18,JUN,MAPK9,NFKB1,PIK3C3,PIK3CD,RXRA,TLR2 |
| Endocannabinoid Developing Neuron Pathway | 2.88 | 0.632 | ADCY1,ADCY4,AKT3,ATF2,CDKN1B,GNB1,MAP2K6,MAPK9,PIK3C3,PIK3CD |
| Erythropoietin Signaling | 2.85 | NA | AKT3,JAK2,JUN,NFKB1,PIK3C3,PIK3CD,SOCS1,SOCS3 |
| Adrenomedullin signaling pathway | 2.8 | 2.138 | ADCY1,ADCY4,AKT3,ARNT,IL18,IL1RN,IL33,MAP2K6,MAPK9,NFKB1,NPR2,PIK3C3,PIK3CD,RXRA |
| Molecular Mechanisms of Cancer | 2.79 | NA | ADCY1,ADCY4,AKT3,ARHGEF1,ARHGEF2,AXIN1,BCL2L1,CDK10,CDK9,CDKN1A,CDKN1B,CTNND1,FNBP1,JAK2,JUN,MAP2K6,MAPK9,NFKB1,PIK3C3,PIK3CD,TCF3,XIAP |
| 4-1BB Signaling in T Lymphocytes | 2.79 | 2.236 | ATF2,IKBKG,JUN,MAPK9,NFKB1 |
| HMGB1 Signaling | 2.7 | 0.632 | AKT3,FNBP1,IL18,IL1R1,IL33,JUN,MAP2K6,MAPK9,NFKB1,PIK3C3,PIK3CD,SERPINE1 |
| Th1 Pathway | 2.69 | 0 | HLA-A,IL18,JAK2,MAP2K6,NFIL3,NFKB1,PIK3C3,PIK3CD,SOCS1,SOCS3 |
| Apelin Endothelial Signaling Pathway | 2.69 | 1.897 | ADCY1,ADCY4,AKT3,ARNT,JUN,MAPK9,NFKB1,PIK3C3,PIK3CD,PRKAA1 |
| Colorectal Cancer Metastasis Signaling | 2.68 | 2.324 | ADCY1,ADCY4,AKT3,AXIN1,BCL2L1,FNBP1,GNB1,JAK2,JUN,MAPK9,MSH2,NFKB1,PIK3C3,PIK3CD,TCF3,TLR2 |
| Role of JAK2 in Hormone-like Cytokine Signaling | 2.67 | NA | JAK2,PTPN1,SH2B1,SOCS1,SOCS3 |
| PPAR Signaling | 2.66 | -1 | IKBKG,IL18,IL1R1,IL1RN,IL33,JUN,NCOR1,NFKB1,RXRA |
| IGF-1 Signaling | 2.6 | 0.378 | AKT3,IGFBP1,JAK2,JUN,PIK3C3,PIK3CD,SOCS1,SOCS3,YWHAZ |
| IL-4 Signaling | 2.58 | NA | AKT3,HLA-A,JAK2,NFAT5,PIK3C3,PIK3CD,SOCS1,SYNJ1 |
| Aryl Hydrocarbon Receptor Signaling | 2.57 | 0.302 | ARNT,CDKN1A,CDKN1B,JUN,NFIA,NFIC,NFIX,NFKB1,RXRA,SMARCA4,TGM2 |
| Sertoli Cell-Sertoli Cell Junction Signaling | 2.57 | NA | A2M,AKT3,ATF2,AXIN1,CLDN12,CLDN14,EPB41,EPN2,JUN,MAP3K13,MAPK9,MYO7A,SPTAN1 |
| Neuroinflammation Signaling Pathway | 2.56 | 2.357 | AKT3,ATF2,HLA-A,HMOX1,IKBKG,IL18,IL1R1,IRAK2,JAK2,JUN,MAPK9,NFAT5,NFKB1,PIK3C3,PIK3CD,TBK1,TLR2,XIAP |
| Endocannabinoid Cancer Inhibition Pathway | 2.55 | -1.508 | ADCY1,ADCY4,AKT3,ATF2,CDKN1A,CDKN1B,MAP2K6,PIK3C3,PIK3CD,PRKAA1,TCF3 |
| Th1 and Th2 Activation Pathway | 2.53 | NA | HLA-A,IL18,IL33,JAK2,JUN,MAP2K6,NFIL3,NFKB1,PIK3C3,PIK3CD,SOCS1,SOCS3 |
| Huntington's Disease Signaling | 2.53 | 1.414 | AKT3,ATF2,BCL2L1,CAPNS1,DNM2,GNB1,HDAC3,HDAC6,HDAC7,JUN,MAPK9,NCOR1,PIK3C3,PIK3CD,TGM2 |
| Type I Diabetes Mellitus Signaling | 2.44 | 0 | HLA-A,IKBKG,IL1R1,JAK2,MAP2K6,MAPK9,NFKB1,SOCS1,SOCS3 |
| Lymphotoxin β Receptor Signaling | 2.42 | 2.236 | AKT3,BCL2L1,IKBKG,NFKB1,PIK3C3,PIK3CD |
| Choline Biosynthesis III | 2.42 | 0 | CHPT1,HMOX1,PCYT1A,PHKA1 |
| April Mediated Signaling | 2.4 | 1.342 | IKBKG,JUN,MAPK9,NFAT5,NFKB1 |
| STAT3 Pathway | 2.37 | -1.134 | CDKN1A,CSF2RB,FGFR2,FGFR3,IL17RA,IL1R1,JAK2,MAPK9,SOCS1,SOCS3 |
| IL-17A Signaling in Gastric Cells | 2.35 | NA | IL17RA,JUN,MAPK9,NFKB1 |
| P2Y Purigenic Receptor Signaling Pathway | 2.35 | 3.162 | ADCY1,ADCY4,AKT3,ATF2,GNB1,JUN,NFKB1,P2RY2,PIK3C3,PIK3CD |
| PI3K/AKT Signaling | 2.35 | 1.265 | AKT3,BCL2L1,CDKN1A,CDKN1B,IKBKG,JAK2,NFKB1,PIK3CD,SYNJ1,YWHAZ |
| B Cell Activating Factor Signaling | 2.3 | 1.342 | IKBKG,JUN,MAPK9,NFAT5,NFKB1 |
| p53 Signaling | 2.27 | -1.134 | AKT3,BCL2L1,CDKN1A,COQ8A,GADD45G,JUN,PIK3C3,PIK3CD |
| Leptin Signaling in Obesity | 2.26 | 0.447 | ADCY1,ADCY4,AKT3,JAK2,PIK3C3,PIK3CD,SOCS3 |
| Role of NFAT in Regulation of the Immune Response | 2.26 | 2.111 | AKT3,ATF2,FCGR2A,GNB1,HLA-A,IKBKG,JUN,NFAT5,NFKB1,PIK3C3,PIK3CD,RCAN1 |
| VDR/RXR Activation | 2.23 | 1.342 | CD14,CDKN1A,CDKN1B,IGFBP1,NCOR1,RXRA,THBD |
| Induction of Apoptosis by HIV1 | 2.2 | 0 | BCL2L1,IKBKG,MAPK9,NFKB1,SLC25A3,XIAP |
| Phospholipase C Signaling | 2.19 | 0.577 | ADCY1,ADCY4,ARHGEF1,ARHGEF2,ATF2,FCGR2A,FNBP1,GNB1,HDAC3,HDAC6,HDAC7,HMOX1,NFAT5,NFKB1,TGM2 |
| Tight Junction Signaling | 2.18 | NA | AKT3,ARHGEF2,CLDN12,CLDN14,CPSF1,EPB41,HSF1,JUN,NFKB1,PATJ,SPTAN1 |
| Role of RIG1-like Receptors in Antiviral Innate Immunity | 2.17 | 0.447 | DDX58,IKBKG,MAVS,NFKB1,TBK1 |
| PEDF Signaling | 2.15 | 1.134 | AKT3,BCL2L1,IKBKG,NFKB1,PIK3C3,PIK3CD,TCF12 |
| Production of Nitric Oxide and Reactive Oxygen Species in Macrophages | 2.14 | 1.732 | AKT3,APOA2,FNBP1,IKBKG,JAK2,JUN,MAP3K13,MAPK9,NFKB1,PIK3C3,PIK3CD,TLR2 |
| CD28 Signaling in T Helper Cells | 2.13 | 2.121 | AKT3,HLA-A,IKBKG,JUN,MAPK9,NFAT5,NFKB1,PIK3C3,PIK3CD |
| MIF Regulation of Innate Immunity | 2.13 | 2.236 | CD14,CD74,JUN,MAPK9,NFKB1 |
| Apelin Pancreas Signaling Pathway | 2.13 | 0.447 | MAPK9,NFKB1,PIK3C3,PIK3CD,PRKAA1 |
| Role of p14/p19ARF in Tumor Suppression | 2.12 | -2 | PIK3C3,PIK3CD,POLR3D,UBTF |
| Renin-Angiotensin Signaling | 2.09 | 1.89 | ADCY1,ADCY4,ATF2,JAK2,JUN,MAPK9,NFKB1,PIK3C3,PIK3CD |
| Germ Cell-Sertoli Cell Junction Signaling | 2.05 | NA | A2M,AXIN1,CTNND1,EPN2,FNBP1,MAP2K6,MAP3K13,MAPK9,MYO7A,PIK3C3,PIK3CD |
| Cell Cycle: G1/S Checkpoint Regulation | 2 | 0.447 | CDKN1A,CDKN1B,FBXL5,HDAC3,HDAC6,HDAC7 |
| Gα12/13 Signaling | 2 | 2.333 | AKT3,ARHGEF1,IKBKG,JUN,MAPK9,NFKB1,PIK3C3,PIK3CD,TEC |
| Sumoylation Pathway | 2 | 0 | DNMT3A,FNBP1,JUN,KDM1A,MAPK9,NFKB1,SP3,XIAP |
| IL-8 Signaling | 1.99 | 2.111 | AKT3,BCL2L1,FNBP1,GNB1,HMOX1,IKBKG,IRAK2,JUN,MAPK9,NFKB1,PIK3C3,PIK3CD |
| Phosphatidylcholine Biosynthesis I | 1.98 | NA | CHPT1,PCYT1A,PHKA1 |
| FGF Signaling | 1.98 | 0.378 | AKT3,ATF2,FGFR2,FGFR3,MAP2K6,PIK3C3,PIK3CD |
| SPINK1 General Cancer Pathway | 1.94 | 0 | AKT3,JAK2,Mt1,Mt2,PIK3C3,PIK3CD |
| TNFR1 Signaling | 1.94 | 2 | IKBKG,JUN,NFKB1,TNFAIP3,XIAP |
| IL-9 Signaling | 1.92 | 1 | NFKB1,PIK3C3,PIK3CD,SOCS3 |
| HGF Signaling | 1.89 | 2.121 | AKT3,ATF2,CDKN1A,JUN,MAP3K13,MAPK9,PIK3C3,PIK3CD |
| Acute Myeloid Leukemia Signaling | 1.89 | 1.134 | AKT3,CSF2RB,MAP2K6,NFKB1,PIK3C3,PIK3CD,TCF3 |
| GM-CSF Signaling | 1.89 | 1.633 | AKT3,BCL2L1,CSF2RB,JAK2,PIK3C3,PIK3CD |
| DNA Methylation and Transcriptional Repression Signaling | 1.87 | NA | DNMT3A,DNMT3B,MTA1,SAP130 |
| IL-15 Signaling | 1.86 | NA | AKT3,BCL2L1,JAK2,NFKB1,PIK3C3,PIK3CD |
| Type II Diabetes Mellitus Signaling | 1.84 | 1.89 | AKT3,IKBKG,MAPK9,NFKB1,PIK3C3,PIK3CD,PRKAA1,SLC27A1,SOCS1,SOCS3 |
| Coagulation System | 1.83 | 0 | A2M,FGA,SERPINE1,THBD |
| Prostate Cancer Signaling | 1.81 | NA | AKT3,ATF2,CDKN1A,CDKN1B,NFKB1,PIK3C3,PIK3CD |
| Hepatic Fibrosis / Hepatic Stellate Cell Activation | 1.8 | NA | A2M,CD14,COL4A1,COL4A5,COL6A3,FGFR2,FN1,IL1R1,LBP,NFKB1,SERPINE1 |
| p38 MAPK Signaling | 1.8 | 0.707 | ATF2,IL18,IL1R1,IL1RN,IL33,IRAK2,MAP2K6,TIFA |
| Growth Hormone Signaling | 1.78 | 0 | A2M,JAK2,PIK3C3,PIK3CD,SOCS1,SOCS3 |
| NGF Signaling | 1.76 | 2.828 | AKT3,ATF2,IKBKG,MAP3K13,MAPK9,NFKB1,PIK3C3,PIK3CD |
| ILK Signaling | 1.76 | 2.111 | AKT3,ATF2,FLNA,FN1,FNBP1,JUN,MAP2K6,MAPK9,NFKB1,PIK3C3,PIK3CD |
| TREM1 Signaling | 1.75 | 0.816 | AKT3,IL18,JAK2,NFKB1,NLRP12,TLR2 |
| Role of NFAT in Cardiac Hypertrophy | 1.72 | 1.667 | ADCY1,ADCY4,AKT3,GNB1,HDAC3,HDAC6,HDAC7,MAP2K6,MAPK9,PIK3C3,PIK3CD,RCAN1 |
| Reelin Signaling in Neurons | 1.7 | NA | ARHGEF1,ARHGEF2,MAPK8IP3,MAPK9,PIK3C3,PIK3CD |
| Estrogen-Dependent Breast Cancer Signaling | 1.7 | 2.449 | AKT3,ATF2,JUN,NFKB1,PIK3C3,PIK3CD |
| Cholecystokinin/Gastrin-mediated Signaling | 1.68 | 0.707 | ATF2,FNBP1,IL18,IL1RN,IL33,JUN,MAP2K6,MAPK9 |
| GP6 Signaling Pathway | 1.68 | 2.121 | ADAM10,AKT3,COL4A1,COL4A5,COL6A3,FGA,PIK3C3,PIK3CD |
| IL-3 Signaling | 1.68 | 1.633 | AKT3,CSF2RB,JAK2,JUN,PIK3C3,PIK3CD |
| Inhibition of Angiogenesis by TSP1 | 1.67 | 1 | AKT3,CD47,JUN,MAPK9 |
| Role of BRCA1 in DNA Damage Response | 1.65 | NA | BABAM2,CDKN1A,MSH2,RAD50,SMARCA2,SMARCA4 |
| IL-7 Signaling Pathway | 1.65 | 0 | AKT3,CDKN1B,JUN,PIK3C3,PIK3CD,SOCS1 |
| MSP-RON Signaling Pathway | 1.65 | NA | CSF2RB,JAK2,PIK3C3,PIK3CD,TLR2 |
| Systemic Lupus Erythematosus In B Cell Signaling Pathway | 1.65 | 0 | AKT3,BCL2L1,FCGR2A,IL18,IL33,JAK2,JUN,MAVS,NFAT5,NFKB1,PIK3C3,PIK3CD,SYNJ1,TBK1 |
| Mouse Embryonic Stem Cell Pluripotency | 1.64 | 1.134 | AKT3,AXIN1,JAK2,PIK3C3,PIK3CD,TCF3,XIAP |
| Antiproliferative Role of Somatostatin Receptor 2 | 1.63 | 0.447 | CDKN1A,CDKN1B,GNB1,NPR2,PIK3C3,PIK3CD |
| Cyclins and Cell Cycle Regulation | 1.63 | NA | CDKN1A,CDKN1B,FBXL5,HDAC3,HDAC6,HDAC7 |
| Apelin Cardiac Fibroblast Signaling Pathway | 1.62 | NA | AKT3,PRKAA1,SERPINE1 |
| Role of PKR in Interferon Induction and Antiviral Response | 1.6 | NA | ATF2,IKBKG,MAP2K6,NFKB1 |
| Role of Pattern Recognition Receptors in Recognition of Bacteria and Viruses | 1.59 | 1.89 | DDX58,IL18,IL33,MAPK9,MAVS,NFKB1,PIK3C3,PIK3CD,TLR2 |
| Amyotrophic Lateral Sclerosis Signaling | 1.58 | 0.816 | AKT3,BCL2L1,CAPNS1,GRIK5,PIK3C3,PIK3CD,XIAP |
| IL-22 Signaling | 1.57 | NA | AKT3,MAPK9,SOCS3 |
| Role of JAK1, JAK2 and TYK2 in Interferon Signaling | 1.57 | NA | JAK2,NFKB1,SOCS1 |
| Prolactin Signaling | 1.56 | 0 | JAK2,JUN,PIK3C3,PIK3CD,SOCS1,SOCS3 |
| HER-2 Signaling in Breast Cancer | 1.56 | NA | AKT3,CDKN1A,CDKN1B,PARD3,PIK3C3,PIK3CD |
| Telomerase Signaling | 1.56 | NA | AKT3,CDKN1A,HDAC3,HDAC6,HDAC7,PIK3C3,PIK3CD |
| PD-1, PD-L1 cancer immunotherapy pathway | 1.56 | -1.134 | AKT3,BCL2L1,CDKN1B,HLA-A,JAK2,PIK3C3,PIK3CD |
| IL-2 Signaling | 1.54 | 1 | AKT3,JUN,PIK3C3,PIK3CD,SOCS1 |
| Relaxin Signaling | 1.51 | 2.333 | ADCY1,ADCY4,AKT3,GNB1,JUN,NFKB1,NPR2,PIK3C3,PIK3CD |
| T Cell Receptor Signaling | 1.5 | NA | IKBKG,JUN,NFAT5,NFKB1,PIK3C3,PIK3CD,TEC |
| Docosahexaenoic Acid (DHA) Signaling | 1.5 | NA | AKT3,BCL2L1,PIK3C3,PIK3CD |
| Myc Mediated Apoptosis Signaling | 1.49 | NA | AKT3,MAPK9,PIK3C3,PIK3CD,YWHAZ |
| Wnt/Ca+ pathway | 1.49 | 1.342 | ATF2,AXIN1,NFAT5,NFKB1,ROR1 |
| Th2 Pathway | 1.46 | 1.342 | HLA-A,IL33,JAK2,JUN,NFKB1,PIK3C3,PIK3CD,SOCS3 |
| mTOR Signaling | 1.45 | 0.378 | AKT3,ATG13,DDIT4,EIF4G1,FNBP1,HMOX1,PIK3C3,PIK3CD,PRKAA1,RPS14,RPS19 |
| NRF2-mediated Oxidative Stress Response | 1.44 | 1.633 | ABCC4,DNAJB2,FKBP5,HMOX1,JUN,JUNB,MAP2K6,MAPK9,PIK3C3,PIK3CD |
| Altered T Cell and B Cell Signaling in Rheumatoid Arthritis | 1.43 | NA | HLA-A,IL18,IL1RN,IL33,NFKB1,TLR2 |
| Rac Signaling | 1.43 | 1.633 | ABI2,JUN,NFKB1,PARD3,PIK3C3,PIK3CD,PIP5K1C |
| HIF1α Signaling | 1.41 | NA | AKT3,ARNT,JUN,LDHA,MAPK9,PIK3C3,PIK3CD |
| Death Receptor Signaling | 1.41 | 0.816 | IKBKG,NFKB1,SPTAN1,TBK1,TIPARP,XIAP |
| Regulation of the Epithelial-Mesenchymal Transition Pathway | 1.4 | NA | AKT3,AXIN1,FGFR2,FGFR3,JAK2,MAP2K6,NFKB1,PIK3C3,PIK3CD,TCF3 |
| iCOS-iCOSL Signaling in T Helper Cells | 1.4 | 1.89 | AKT3,HLA-A,IKBKG,NFAT5,NFKB1,PIK3C3,PIK3CD |
| Role of JAK1 and JAK3 in γc Cytokine Signaling | 1.39 | NA | JAK2,PIK3C3,PIK3CD,SOCS1,SOCS3 |
| Tec Kinase Signaling | 1.39 | 0.707 | FNBP1,GNB1,GTF2I,JAK2,MAPK9,NFKB1,PIK3C3,PIK3CD,TEC |
| Th17 Activation Pathway | 1.38 | 0 | IL1R1,IRAK2,JAK2,NFAT5,NFKB1,SOCS3 |
| PI3K Signaling in B Lymphocytes | 1.37 | 2.121 | AKT3,ATF2,ATF5,IKBKG,JUN,NFAT5,NFKB1,PIK3CD |
| White Adipose Tissue Browning Pathway | 1.37 | 1.414 | ADCY1,ADCY4,ATF2,FGFR2,FGFR3,LDHA,PRKAA1,RXRA |
| Actin Cytoskeleton Signaling | 1.36 | 0.707 | ABI2,ARHGEF1,CD14,DIAPH1,FLNA,FN1,GIT1,LBP,PIK3C3,PIK3CD,PIP5K1C |
| Graft-versus-Host Disease Signaling | 1.35 | NA | HLA-A,IL18,IL1RN,IL33 |
| Cell Cycle: G2/M DNA Damage Checkpoint Regulation | 1.35 | 1 | CDKN1A,FBXL5,TRIP12,YWHAZ |
| Assembly of RNA Polymerase III Complex | 1.32 | NA | GTF3C1,POLR3D |
| TGF-β Signaling | 1.32 | 1.633 | INHBB,JUN,MAP2K6,MAPK9,SERPINE1,SKI |
| ATM Signaling | 1.3 | 2 | ATF2,CDKN1A,GADD45G,JUN,MAPK9,RAD50 |

**Table 11.** Significant pathways for differentially expressed transcripts in ^16^O vs. non-irradiated control at 12 months analyzed by IPA.

| **^16^O, 1 month** | | **^16^O, 2 months** | | **^16^O, 4 months** | | **^16^O, 9 months** | | **^16^O, 12 months** | |
| --- | --- | --- | --- | --- | --- | --- | --- | --- | --- |
| **Module** | **Transcript Ensemble ID** | **Module** | **Transcript Ensemble ID** | **Module** | **Transcript Ensemble ID** | **Module** | **Transcript Ensemble ID** | **Module** | **Transcript Ensemble ID** |
| 19 | ENSMUST00000237265 | 11 | ENSMUST00000235160 | 17 | ENSMUST00000237369 | 14 | ENSMUST00000235927 | 10 | ENSMUST00000237369 |
| 1 | ENSMUST00000235180 | 15 | ENSMUST00000237442 | 1 | ENSMUST00000237170 | 15 | ENSMUST00000237814 | 10 | ENSMUST00000238098 |
| 23 | ENSMUST00000236215 | 16 | ENSMUST00000235764 | 27 | ENSMUST00000237874 | 16 | ENSMUST00000237749 | 10 | ENSMUST00000235828 |
| 25 | ENSMUST00000235160 | 17 | ENSMUST00000236950 | 30 | ENSMUST00000237472 | 17 | ENSMUST00000235957 | 12 | ENSMUST00000237042 |
| 28 | ENSMUST00000236591 | 17 | ENSMUST00000238418 | 31 | ENSMUST00000235764 | 21 | ENSMUST00000236736 | 15 | ENSMUST00000236504 |
| 29 | ENSMUST00000235927 | 23 | ENSMUST00000237060 | 35 | ENSMUST00000236046 | 30 | ENSMUST00000238222 | 15 | ENSMUST00000238021 |
| 29 | ENSMUST00000236925 | 23 | ENSMUST00000235620 | 35 | ENSMUST00000236687 | 31 | ENSMUST00000237125 | 22 | ENSMUST00000238288 |
| 2 | ENSMUST00000235304 | 26 | ENSMUST00000238151 | 39 | ENSMUST00000236950 | 31 | ENSMUST00000236330 | 23 | ENSMUST00000235318 |
| 2 | ENSMUST00000236824 | 28 | ENSMUST00000236925 | 41 | ENSMUST00000236209 | 35 | ENSMUST00000238729 | 23 | ENSMUST00000238267 |
| 2 | ENSMUST00000235231 | 29 | ENSMUST00000236873 | 45 | ENSMUST00000237798 | 40 | ENSMUST00000236898 | 2 | ENSMUST00000238677 |
| 36 | ENSMUST00000236950 | 29 | ENSMUST00000237472 | 47 | ENSMUST00000236030 | 45 | ENSMUST00000235558 | 31 | ENSMUST00000236414 |
| 37 | ENSMUST00000235647 | 30 | ENSMUST00000236303 | 49 | ENSMUST00000237854 | 8 | ENSMUST00000236824 | 32 | ENSMUST00000237170 |
| 3 | ENSMUST00000235957 | 30 | ENSMUST00000237854 | 4 | ENSMUST00000238288 | 9 | ENSMUST00000236950 | 34 | ENSMUST00000235648 |
| 3 | ENSMUST00000237185 | 36 | ENSMUST00000236186 |  |  |  |  | 36 | ENSMUST00000235411 |
| 3 | ENSMUST00000237146 | 37 | ENSMUST00000237305 |  |  |  |  | 37 | ENSMUST00000238331 |
| 40 | ENSMUST00000237170 | 37 | ENSMUST00000235518 |  |  |  |  | 37 | ENSMUST00000237823 |
| 40 | ENSMUST00000235620 | 3 | ENSMUST00000238288 |  |  |  |  | 3 | ENSMUST00000236873 |
| 40 | ENSMUST00000235633 | 3 | ENSMUST00000237603 |  |  |  |  | 40 | ENSMUST00000238368 |
| 41 | ENSMUST00000236195 | 46 | ENSMUST00000237103 |  |  |  |  | 41 | ENSMUST00000235135 |
| 45 | ENSMUST00000236567 | 49 | ENSMUST00000237742 |  |  |  |  | 44 | ENSMUST00000236006 |
| 47 | ENSMUST00000236873 | 7 | ENSMUST00000237749 |  |  |  |  | 48 | ENSMUST00000238306 |
| 47 | ENSMUST00000236414 | 7 | ENSMUST00000236480 |  |  |  |  | 49 | ENSMUST00000235620 |
| 47 | ENSMUST00000237587 | 9 | ENSMUST00000235318 |  |  |  |  |  |  |
| 7 | ENSMUST00000238288 |  |  |  |  |  |  |  |  |

**Table 12.** Unmapped differentially expressed transcripts (by IPA) in ^16^O experiments at all time points. Each unmapped ensemble transcript ID is listed with the corresponding module number in SOM Figure 4.

| **Ingenuity Canonical Pathways** | **-log(p-value)** | **z-score** | **Molecules** |
| --- | --- | --- | --- |
| LXR/RXR Activation | 6.61 | 0.258 | APOA2,APOA5,APOB,APOE,CD36,CLU,CYP7A1,FASN,HMGCR,IRF3,KNG1,LDLR,NFKB1,NR1H2,PLTP,SERPINF1,TF |
| Role of JAK2 in Hormone-like Cytokine Signaling | 5.28 | NA | SH2B1,SH2B2,SH2B3,SHC1,SIRPA,SOCS2,STAT1,TYK2 |
| FXR/RXR Activation | 4.24 | NA | APOA2,APOB,APOE,CLU,CYP7A1,FASN,FETUB,G6PC3,KNG1,LIPC,PLTP,SCARB1,SERPINF1,TF |
| Clathrin-mediated Endocytosis Signaling | 3.59 | NA | ACTR3,AP1B1,APOA2,APOB,APOE,CD2AP,CLU,DNM2,F2,LDLR,MET,NUMB,PICALM,PROK1,RAB7A,TF |
| LPS/IL-1 Mediated Inhibition of RXR Function | 3.3 | 0 | ACSL3,ALAS1,APOE,CAT,CYP2B6,CYP2C8,CYP7A1,FABP5,FMO1,FMO2,LIPC,NDST1,NDST2,NR1H2,NR1I3,PLTP,SCARB1 |
| Circadian Rhythm Signaling | 3.29 | NA | ARNTL,ATF2,CREB1,PER1,PER2,PER3 |
| Bupropion Degradation | 2.98 | -0.447 | CYP2B6,CYP2C8,CYP2E1,CYP2F1,POR |
| ERK5 Signaling | 2.85 | 0 | ATF2,CREB1,GAB1,MAPK7,RPS6KC1,WNK1,YWHAG,YWHAQ |
| Acute Phase Response Signaling | 2.53 | -0.816 | APOA2,C1R,F2,HNRNPK,IKBKG,ITIH3,MAPK14,NFKB1,NR3C1,SERPINF1,SHC1,SOCS2,TF |
| Mitochondrial Dysfunction | 2.39 | NA | ATP5F1B,ATP5MC2,ATP5PB,ATPAF1,CAT,COX6B1,CYB5A,GPX4,NDUFA4,PARK7,PDHA1,PINK1,VDAC2 |
| Estrogen Biosynthesis | 2.37 | -0.816 | CYP2B6,CYP2C8,CYP2E1,CYP2F1,HSD17B12,POR |
| Acetone Degradation I (to Methylglyoxal) | 2.35 | -0.447 | CYP2B6,CYP2C8,CYP2E1,CYP2F1,POR |
| PXR/RXR Activation | 2.34 | NA | ALAS1,CYP2B6,CYP2C8,CYP7A1,IGFBP1,NR1I3,NR3C1 |
| Sumoylation Pathway | 2.29 | 0 | CEBPA,NFKB1,NR3C1,RHOBTB1,RHOT1,RPA1,SENP5,SP3,XIAP |
| Production of Nitric Oxide and Reactive Oxygen Species in Macrophages | 2.27 | 2.309 | APOA2,APOB,APOE,CAT,CLU,IKBKG,MAPK14,NFKB1,RHOBTB1,RHOT1,SIRPA,STAT1,TYK2 |
| Ephrin B Signaling | 2.2 | 1.342 | ABI1,CAP1,CTNNB1,EPHB4,GNB1,GNB2,HNRNPK |
| Nicotine Degradation II | 2.17 | -0.378 | CYP2B6,CYP2C8,CYP2E1,CYP2F1,FMO1,FMO2,POR |
| Actin Cytoskeleton Signaling | 2.16 | 0.905 | ACTN4,ACTR3,ARHGEF12,CYFIP1,F2,FLNA,GIT1,KNG1,MYH10,MYH14,PFN1,RDX,SHC1,TLN2 |
| Role of PKR in Interferon Induction and Antiviral Response | 2.16 | NA | ATF2,IKBKG,MAPK14,NFKB1,STAT1 |
| Adipogenesis pathway | 2.16 | NA | ARNTL,CEBPA,CTNNB1,EZH2,FGFR2,KMT2B,LPIN1,NOCT,PER2,RPS6KC1 |
| PI3K/AKT Signaling | 2.11 | 1.667 | CTNNB1,GAB1,IKBKG,NFKB1,SHC1,SYNJ2,TSC1,TYK2,YWHAG,YWHAQ |
| ATM Signaling | 2.09 | 0 | ATF2,BRAT1,CREB1,GADD45G,HERC2,MAPK14,TDP1,USP7 |
| NGF Signaling | 2.03 | 0.333 | ATF2,CREB1,GAB1,IKBKG,MAPK7,NFKB1,RPS6KC1,SHC1,SMPD4 |
| IL-15 Production | 2 | NA | EPHB4,FGFR2,IRF3,KIT,MET,NFKB1,STAT1,TNK2,TYK2 |
| SAPK/JNK Signaling | 1.94 | 0.378 | ATF2,GAB1,GNB1,HNRNPK,MAP4K3,MAPK8IP3,MINK1,SHC1 |
| mTOR Signaling | 1.9 | 0.378 | DGKZ,EIF4A2,EIF4G1,EIF4G2,FKBP1A,PLD2,PROK1,RHOBTB1,RHOT1,RPS14,RPS6KC1,STK11,TSC1 |
| iNOS Signaling | 1.87 | 1.342 | IKBKG,MAPK14,NFKB1,STAT1,TYK2 |
| HIPPO signaling | 1.86 | 0.447 | AMOT,DLG1,ITCH,PARD3,Ppp1cc,YWHAG,YWHAQ |
| 4-1BB Signaling in T Lymphocytes | 1.85 | 0 | ATF2,IKBKG,MAPK14,NFKB1 |
| Cell Cycle: G2/M DNA Damage Checkpoint Regulation | 1.84 | NA | FBXL5,HIPK2,TRIP12,YWHAG,YWHAQ |
| Remodeling of Epithelial Adherens Junctions | 1.79 | -1 | ACTN4,ACTR3,CTNNB1,DNM2,MET,RAB7A |
| DNA Methylation and Transcriptional Repression Signaling | 1.76 | NA | CHD4,H3F3A/H3F3B,MECP2,MTA1 |
| TR/RXR Activation | 1.74 | NA | APOA5,CYP7A1,FASN,GPS2,LDLR,SCARB1,THRSP |
| IL-12 Signaling and Production in Macrophages | 1.7 | NA | APOA2,APOB,APOE,CLU,IKBKG,MAPK14,NFKB1,RAB7A,STAT1 |
| Estrogen Receptor Signaling | 1.68 | NA | G6PC3,H3F3A/H3F3B,IGFBP1,MED23,NR3C1,RBFOX2,SHC1,TAF10,TAF6 |
| Protein Kinase A Signaling | 1.67 | -1.069 | ADCY9,AKAP1,ANAPC5,ATF2,CREB1,CTNNB1,FLNA,GNB1,GNB2,H3F3A/H3F3B,MYH10,NFAT5,NFKB1,Ppp1cc,PTCH1,PTPRC,PTPRJ,SIRPA,YWHAG,YWHAQ |
| Hypoxia Signaling in the Cardiovascular System | 1.65 | NA | ARNT,ATF2,CDC34,CREB1,P4HB,UBE2D3 |
| Melanocyte Development and Pigmentation Signaling | 1.62 | 0 | ADCY9,ATF2,CREB1,KIT,RPS6KC1,SH2B2,SHC1 |
| p38 MAPK Signaling | 1.62 | -0.378 | ATF2,CREB1,H3F3A/H3F3B,HMGN1,MAPK14,PLA2G6,RPS6KC1,STAT1 |
| Unfolded protein response | 1.61 | NA | CD82,CEBPA,HSPA5,P4HB,SYVN1 |
| B Cell Receptor Signaling | 1.58 | -0.632 | ATF2,BCL6,CREB1,GAB1,IKBKG,MAPK14,NFAT5,NFKB1,PTPRC,SHC1,SYNJ2 |
| April Mediated Signaling | 1.56 | 0 | IKBKG,MAPK14,NFAT5,NFKB1 |
| ILK Signaling | 1.53 | 0.632 | ACTN4,ATF2,CREB1,CTNNB1,FLNA,MYH10,MYH14,NFKB1,PROK1,RHOBTB1,RHOT1 |
| PPARα/RXRα Activation | 1.51 | -1 | ADCY9,APOA2,CD36,CKAP5,CYP2C8,FASN,IKBKG,MAPK14,MED23,NFKB1,SHC1 |
| Role of BRCA1 in DNA Damage Response | 1.5 | NA | BABAM2,E2F8,RBL2,RPA1,SMARCA2,STAT1 |
| B Cell Activating Factor Signaling | 1.49 | 0 | IKBKG,MAPK14,NFAT5,NFKB1 |
| Atherosclerosis Signaling | 1.49 | NA | ABHD3,APOA2,APOB,APOE,CD36,CLU,NFKB1,PLA2G6 |
| IL-22 Signaling | 1.48 | NA | MAPK14,STAT1,TYK2 |
| Role of JAK1, JAK2 and TYK2 in Interferon Signaling | 1.48 | NA | NFKB1,STAT1,TYK2 |
| D-myo-inositol (1,3,4)-trisphosphate Biosynthesis | 1.48 | NA | IPMK,SEC16A,SYNJ2 |
| Apelin Adipocyte Signaling Pathway | 1.48 | -0.816 | ADCY9,CAT,GPX1,GPX4,MAPK14,MAPK7 |
| ERK/MAPK Signaling | 1.48 | 0.333 | ATF2,CREB1,ELF1,ELF4,H3F3A/H3F3B,PLA2G6,SHC1,STAT1,TLN2,YWHAG,YWHAQ |
| Nicotine Degradation III | 1.46 | -0.447 | CYP2B6,CYP2C8,CYP2E1,CYP2F1,POR |
| PCP pathway | 1.46 | 0.447 | ATF2,DAAM1,LGR4,PFN1,PRICKLE1 |
| tRNA Charging | 1.46 | -2.449 | FARSA,FARSB,LARS2,MARS,Qars,TARS |
| Role of JAK family kinases in IL-6-type Cytokine Signaling | 1.44 | NA | MAPK14,STAT1,TYK2 |
| Activation of IRF by Cytosolic Pattern Recognition Receptors | 1.41 | 0.447 | ATF2,IKBKG,IRF3,NFKB1,STAT1 |
| 1D-myo-inositol Hexakisphosphate Biosynthesis II (Mammalian) | 1.39 | NA | IPMK,SEC16A,SYNJ2 |
| Epithelial Adherens Junction Signaling | 1.38 | NA | ACTN4,ACTR3,CTNNB1,EPN2,MET,MYH10,MYH14,PARD3,SSX2IP |
| BMP signaling pathway | 1.37 | 0.816 | ATF2,CHRD,CREB1,MAPK14,NFKB1,XIAP |
| Autophagy | 1.36 | NA | CTSH,CTSS,CTSV,MAP1LC3A,NBR1 |
| BAG2 Signaling Pathway | 1.36 | 0 | HSPA5,MAPK14,NFKB1,PINK1 |
| Mevalonate Pathway I | 1.35 | NA | HADHB,HMGCR,IDI1 |
| FGF Signaling | 1.33 | -0.816 | ATF2,CREB1,FGFR2,GAB1,MAPK14,MET |
| Pancreatic Adenocarcinoma Signaling | 1.33 | 1.633 | CYP2E1,E2F8,NFKB1,PLD2,PROK1,STAT1,TYK2 |

**Table 13.** Significant pathways for differentially expressed transcripts in ^28^Si vs. non-irradiated control at 1 month analyzed by IPA.

| **Ingenuity Canonical Pathways** | **-log(p-value)** | **z-score** | **Molecules** |
| --- | --- | --- | --- |
| Role of JAK2 in Hormone-like Cytokine Signaling | 3.82 | NA | SH2B1,SH2B2,SH2B3,SHC1,SIRPA,STAT1 |
| Huntington's Disease Signaling | 3.5 | -0.447 | ATF2,ATP5F1B,ATP5PB,GNB1,GNB2,GPAA1,HDAC5,HDAC6,MAPK9,NCOR1,NCOR2,PDPK1,SGK1,SHC1,SIN3A,TCERG1 |
| Adipogenesis pathway | 3.33 | NA | CEBPA,EZH2,FGFR2,HDAC5,HDAC6,KAT2A,KMT2B,PPIP5K1,RPS6KA1,SIN3A,TBL1XR1 |
| FXR/RXR Activation | 3.28 | NA | APOA2,BAAT,FETUB,LIPC,MAPK9,NR0B2,PKLR,SAA1,SCARB1,SERPINF1,SLC22A7 |
| RhoA Signaling | 3.02 | -0.632 | ACTR3,ARHGEF1,BAIAP2,CDC42EP4,MPRIP,PFN1,RAPGEF6,SEPT2,SEPT9,TTN |
| Aryl Hydrocarbon Receptor Signaling | 2.97 | -0.707 | ALDH2,ATR,CDKN1B,HSP90AB1,MGST1,NCOR2,NFIA,NFIX,NQO2,NR0B2,RBL2 |
| Cell Cycle: G1/S Checkpoint Regulation | 2.92 | -0.816 | ATR,CDKN1B,E2F3,HDAC5,HDAC6,RBL2,SIN3A |
| D-myo-inositol (1,4,5)-trisphosphate Degradation | 2.75 | 0 | INPP5A,INPP5D,SEC16A,SYNJ2 |
| Mitochondrial Dysfunction | 2.65 | NA | ATP5F1B,ATP5PB,CAT,COX6B1,CYB5A,CYC1,GPX4,MAPK9,MT-ND4L,NDUFAF1,NDUFV1,PINK1 |
| D-myo-inositol (1,3,4)-trisphosphate Biosynthesis | 2.6 | 0 | INPP5A,INPP5D,SEC16A,SYNJ2 |
| 1D-myo-inositol Hexakisphosphate Biosynthesis II (Mammalian) | 2.47 | 0 | INPP5A,INPP5D,SEC16A,SYNJ2 |
| FLT3 Signaling in Hematopoietic Progenitor Cells | 2.39 | -1.134 | ATF2,INPP5D,PDPK1,RPS6KA1,SHC1,STAT1,STAT2 |
| LXR/RXR Activation | 2.39 | 0 | APOA2,APOA5,CD36,ECHS1,NCOR1,NCOR2,NR1H2,SAA1,SERPINF1 |
| LPS/IL-1 Mediated Inhibition of RXR Function | 2.38 | 1.134 | ACOX2,ACSL3,ACSL4,ALAS1,ALDH2,CAT,LIPC,MAPK9,MGST1,NDST2,NR0B2,NR1H2,SCARB1 |
| Ephrin Receptor Signaling | 2.28 | -0.333 | ACTR3,ATF2,BCAR1,EPHB4,GNB1,GNB2,GRIN3A,PROK1,PXN,RAPGEF1,SHC1 |
| Sirtuin Signaling Pathway | 2.2 | -0.277 | ACLY,ATP5F1B,ATP5PB,CYC1,GABPB1,GABPB2,GLUD1,KAT2A,MT-ND4L,NDUFAF1,NDUFV1,NR1H2,PFKFB3,PFKM,POLR3D,STK11 |
| Superpathway of D-myo-inositol (1,4,5)-trisphosphate Metabolism | 2.14 | 0 | INPP5A,INPP5D,SEC16A,SYNJ2 |
| Actin Cytoskeleton Signaling | 2.06 | -1.265 | ACTR3,ARHGEF1,BAIAP2,BCAR1,DIAPH1,FLNA,MPRIP,MYH14,PFN1,PXN,SHC1,TTN |
| NGF Signaling | 2.04 | 0 | ATF2,MAP3K13,Map3k7,MAPK9,PDPK1,RPS6KA1,SHC1,SMPD4 |
| SAPK/JNK Signaling | 1.89 | 0.816 | ATF2,GNB1,HNRNPK,MAP3K13,MAPK8IP3,MAPK9,SHC1 |
| Role of BRCA1 in DNA Damage Response | 1.88 | NA | ATR,E2F3,PBRM1,RBL2,SMARCC1,STAT1 |
| Cyclins and Cell Cycle Regulation | 1.85 | NA | ATR,CDKN1B,E2F3,HDAC5,HDAC6,SIN3A |
| Calcium Signaling | 1.8 | 0.816 | ATF2,ATP2A2,CACNA1A,CALR,GRIA3,GRIN3A,HDAC5,HDAC6,MICU1,MYH14,NFAT5 |
| Signaling by Rho Family GTPases | 1.77 | 0.333 | ACTR3,ARHGEF1,ARHGEF10,ARHGEF3,BAIAP2,CDC42EP4,GNB1,GNB2,MAPK9,PARD3,SEPT2,SEPT9 |
| B Cell Receptor Signaling | 1.74 | 0.333 | ATF2,INPP5D,MAP3K13,Map3k7,MAPK9,NFAT5,PDPK1,SHC1,SYNJ2,TCF3 |
| α-tocopherol Degradation | 1.72 | NA | CYP4F12,CYP4F3 |
| Estrogen Receptor Signaling | 1.72 | NA | MED12,NCOR1,NCOR2,NR0B2,SHC1,TAF10,TAF6,TRRAP |
| Glutamate Receptor Signaling | 1.71 | NA | GNB1,GRIA3,GRIN3A,GRM8,SLC38A1 |
| γ-linolenate Biosynthesis II (Animals) | 1.71 | NA | ACSL3,ACSL4,CYB5A |
| Glucocorticoid Receptor Signaling | 1.68 | NA | CEBPA,FKBP5,HSP90AB1,MAPK9,NCOR1,NCOR2,NFAT5,PBRM1,SGK1,SHC1,SMARCC1,STAT1,TAF10,TAF6,TAT |
| PPARα/RXRα Activation | 1.67 | 0 | ADCY4,APOA2,CD36,CKAP5,HSP90AB1,MED12,NCOR1,NCOR2,NR0B2,SHC1 |
| Cdc42 Signaling | 1.65 | 0 | ACTR3,ATF2,BAIAP2,CDC42BPA,DIAPH1,MAPK9,MPRIP,PARD3,TNK2 |
| Protein Kinase A Signaling | 1.62 | -0.302 | ADCY4,ADD1,AKAP1,ANAPC5,ATF2,EYA3,FLNA,GNB1,GNB2,NFAT5,PPP1R10,PTPN4,PTPRJ,PXN,SIRPA,TCF3,TTN |
| RAR Activation | 1.59 | NA | ADCY4,AKR1C4,MAPK9,NCOR1,NCOR2,PBRM1,PDPK1,PML,SMARCC1,TNIP1 |
| Insulin Receptor Signaling | 1.59 | -0.707 | ACLY,INPP5D,PDPK1,PPP1R10,RAPGEF1,SGK1,SHC1,SYNJ2 |
| Oxidative Phosphorylation | 1.58 | -1.134 | ATP5F1B,ATP5PB,COX6B1,CYB5A,CYC1,MT-ND4L,NDUFV1 |
| Phospholipase C Signaling | 1.53 | 0.632 | ADCY4,ARHGEF1,ARHGEF10,ARHGEF3,ATF2,GNB1,GNB2,HDAC5,HDAC6,MPRIP,NFAT5,SHC1 |
| Breast Cancer Regulation by Stathmin1 | 1.53 | NA | ADCY4,ARHGEF1,ARHGEF10,ARHGEF3,CDKN1B,E2F3,GNB1,GNB2,PPP1R10,SHC1 |
| ATM Signaling | 1.51 | 1.342 | ATF2,ATR,HERC2,MAPK9,SMC2,TRRAP |
| Ephrin B Signaling | 1.47 | NA | EPHB4,GNB1,GNB2,HNRNPK,PXN |
| PTEN Signaling | 1.43 | 0.378 | BCAR1,CDKN1B,FGFR2,INPP5D,PDPK1,SHC1,SYNJ2 |
| Integrin Signaling | 1.42 | -1.667 | ACTR3,BCAR1,ITGA8,MPRIP,PFN1,PXN,RAPGEF1,SHC1,TNK2,TTN |
| Phagosome Maturation | 1.42 | NA | ATP6AP1,CALR,CTSH,CTSV,Dync1i2,DYNC1LI2,GPAA1,VPS33B |
| Assembly of RNA Polymerase III Complex | 1.41 | NA | GTF3C1,POLR3D |
| Chronic Myeloid Leukemia Signaling | 1.4 | NA | CDKN1B,E2F3,HDAC5,HDAC6,RBL2,SIN3A |
| Reelin Signaling in Neurons | 1.38 | NA | ARHGEF1,ARHGEF10,ARHGEF3,MAPK8IP3,MAPK9 |
| FcγRIIB Signaling in B Lymphocytes | 1.36 | -1 | CACNA1A,INPP5D,MAPK9,PDPK1,SHC1 |
| Amyotrophic Lateral Sclerosis Signaling | 1.35 | 1.342 | CACNA1A,CAT,GRIA3,GRIN3A,PROK1,XIAP |
| 3-phosphoinositide Degradation | 1.35 | 0 | INPP4A,INPP5D,PPIP5K1,PPP1R16B,PTPRJ,SIRPA,SYNJ2,UBLCP1 |
| Type II Diabetes Mellitus Signaling | 1.34 | 1 | ACSL3,ACSL4,CACNA1A,CD36,MAPK9,PDPK1,PKLR,SMPD4 |
| Apelin Adipocyte Signaling Pathway | 1.33 | -0.447 | ADCY4,CAT,GPX4,MAPK9,MGST1 |
| Production of Nitric Oxide and Reactive Oxygen Species in Macrophages | 1.32 | 0.333 | APOA2,CAT,IRF1,MAP3K13,Map3k7,MAPK9,PPP1R10,SIRPA,STAT1 |
| Sertoli Cell-Sertoli Cell Junction Signaling | 1.32 | NA | ATF2,BCAR1,CLDN12,EPB41,MAP3K13,Map3k7,MAPK9,SPTAN1,TJP3 |
| DNA Methylation and Transcriptional Repression Signaling | 1.31 | NA | ARID4B,CHD4,SIN3A |
| Sumoylation Pathway | 1.3 | 0 | CEBPA,MAPK9,PML,RCC1,SENP6,XIAP |

**Table 14.** Significant pathways for differentially expressed transcripts in ^28^Si vs. non-irradiated control at 2 months analyzed by IPA.

| **Ingenuity Canonical Pathways** | **-log(p-value)** | **z-score** | **Molecules** |
| --- | --- | --- | --- |
| Acute Phase Response Signaling | 10 | -1.5 | APOA2,CFB,CP,F2,FGA,FN1,GRB2,HNRNPK,HP,IKBKB,IL1R1,ITIH2,ITIH3,ITIH4,JAK2,LBP,NR3C1,PIK3CD,SAA1,SAA2-SAA4,Saa3,SERPINA1,SERPINA3,SOCS2,SOCS3,TF |
| LXR/RXR Activation | 4.93 | -0.577 | ABCG8,APOA2,APOA5,FGA,IL1R1,ITIH4,LBP,LPL,NCOR2,NR1H2,NR1H3,SAA1,SERPINA1,TF,TLR3 |
| Role of JAK2 in Hormone-like Cytokine Signaling | 4.13 | NA | JAK2,SH2B1,SH2B3,SIRPA,SOCS2,SOCS3,STAT5A |
| IL-4 Signaling | 4.07 | NA | GRB2,HLA-A,INPP5B,INPP5D,JAK2,NFAT5,NR3C1,PIK3C2G,PIK3CD,SYNJ1,SYNJ2 |
| EIF2 Signaling | 3.98 | -2.111 | ACTB,EIF4G1,EIF4G3,GRB2,HSPA5,MT-TM,PIK3C2G,PIK3CD,PTBP1,RPL10A,RPL11,RPL12,RPL18A,RPL37,RPL38,RPS14,RPS15A,RPS19,XIAP |
| Clathrin-mediated Endocytosis Signaling | 3.81 | NA | ACTB,AP1G2,APOA2,ARRB1,CD2AP,F2,GRB2,MDM2,MET,PICALM,PIK3C2G,PIK3CD,PIP5K1C,RAB11A,SERPINA1,SYNJ1,TF |
| Sumoylation Pathway | 3.8 | -1.89 | CEBPA,DNMT3A,MDM2,NR3C1,PML,RANGAP1,RCC1,RFC1,RHOBTB1,RHOT1,SENP6,XIAP |
| PI3K/AKT Signaling | 3.42 | 0.832 | CDKN1B,GRB2,IKBKB,INPP5B,INPP5D,ITGA3,JAK2,MDM2,PIK3CD,SYNJ1,SYNJ2,TSC1,YWHAG |
| Hereditary Breast Cancer Signaling | 3.27 | NA | ACTB,ARID1A,ATR,BRCA2,FANCM,GADD45G,HDAC10,HDAC6,PIK3C2G,PIK3CD,RAD50,RFC1,SMARCA2 |
| p53 Signaling | 3 | 0.378 | ATR,COQ8A,GADD45G,MDM2,PIDD1,PIK3C2G,PIK3CD,PML,ST13,STAG1 |
| Prolactin Signaling | 2.9 | 1.414 | GRB2,JAK2,NR3C1,PIK3C2G,PIK3CD,PRKCE,SOCS2,SOCS3,STAT5A |
| HER-2 Signaling in Breast Cancer | 2.9 | NA | CDKN1B,GRB2,MDM2,PARD3,PARD6A,PIK3C2G,PIK3CD,PRKCE,TSC1 |
| Chronic Myeloid Leukemia Signaling | 2.83 | NA | CDKN1B,GRB2,HDAC10,HDAC6,IKBKB,MDM2,PIK3C2G,PIK3CD,STAT5A,TGFBR1 |
| Relaxin Signaling | 2.82 | 0.378 | ADCY3,ADCY6,GNAI2,GNB1,GNG12,GUCY2C,NPR2,PDE1A,PDE4B,PDE4C,PDE5A,PIK3C2G,PIK3CD |
| Insulin Receptor Signaling | 2.68 | -0.302 | ACLY,GRB10,GRB2,INPP5B,INPP5D,JAK2,PIK3C2G,PIK3CD,SOCS3,SYNJ1,SYNJ2,TSC1 |
| TR/RXR Activation | 2.66 | NA | APOA5,DIO1,FGA,HP,MDM2,ME1,NCOR2,PIK3C2G,PIK3CD |
| PTEN Signaling | 2.63 | 0.302 | CDKN1B,FGFR3,GRB2,IKBKB,INPP5B,INPP5D,ITGA3,PIK3CD,SYNJ1,SYNJ2,TGFBR1 |
| Growth Hormone Signaling | 2.63 | 2.121 | CEBPA,JAK2,PIK3C2G,PIK3CD,PRKCE,SOCS2,SOCS3,STAT5A |
| Tec Kinase Signaling | 2.63 | 1 | ACTB,GNAI2,GNB1,GNG12,GTF2I,ITGA3,JAK2,PIK3C2G,PIK3CD,PRKCE,RHOBTB1,RHOT1,STAT5A |
| Pancreatic Adenocarcinoma Signaling | 2.59 | -0.378 | BRCA2,CDKN1B,GRB2,JAK2,MDM2,PIK3C2G,PIK3CD,PLD1,RALBP1,TGFBR1 |
| Nitric Oxide Signaling in the Cardiovascular System | 2.56 | 0.632 | ATP2A2,ATP2A3,Calm1 (includes others),GUCY2C,ITPR1,PDE1A,PDE5A,PIK3C2G,PIK3CD,PRKCE |
| IL-9 Signaling | 2.47 | 1 | PIK3C2G,PIK3CD,SOCS2,SOCS3,STAT5A |
| Adipogenesis pathway | 2.45 | NA | ARNTL,ATG7,CEBPA,FGFR3,HDAC10,HDAC6,KAT6A,LPL,NOCT,PPIP5K1,SETDB1 |
| Role of BRCA1 in DNA Damage Response | 2.45 | NA | ACTB,ARID1A,ATR,BRCA2,FANCM,RAD50,RFC1,SMARCA2 |
| JAK/Stat Signaling | 2.45 | 0.707 | GRB2,JAK2,PIAS3,PIK3C2G,PIK3CD,SOCS2,SOCS3,STAT5A |
| Cardiac Hypertrophy Signaling (Enhanced) | 2.36 | 0.962 | ACVR2B,ADCY3,ADCY6,ATF2,ATP2A2,ATP2A3,Calm1 (includes others),DIAPH1,FGFR3,GNAI2,GNB1,HDAC10,HDAC6,IKBKB,IL1R1,ITGA3,ITPR1,JAK2,NFAT5,PDE1A,PDE4B,PDE4C,PDE5A,PIK3C2G,PIK3CD,PRKCE,TGFBR1 |
| Phospholipase C Signaling | 2.36 | -0.302 | ADCY3,ADCY6,ATF2,Calm1 (includes others),FCGR2A,GNB1,GNG12,GRB2,HDAC10,HDAC6,ITGA3,ITPR1,NFAT5,PLD1,PRKCE,RHOBTB1,RHOT1 |
| D-myo-inositol (1,4,5)-trisphosphate Degradation | 2.35 | 0 | INPP5B,INPP5D,SYNJ1,SYNJ2 |
| Cell Cycle: G1/S Checkpoint Regulation | 2.31 | 1.633 | ATR,CDKN1B,FBXL5,HDAC10,HDAC6,MDM2,RPL11 |
| Ephrin Receptor Signaling | 2.29 | 0.905 | ABI1,ADAM10,ATF2,GNAI2,GNB1,GNG12,GRB2,ITGA3,ITSN1,JAK2,MAP4K4,PIK3C2G,SORBS1 |
| Breast Cancer Regulation by Stathmin1 | 2.25 | NA | ADCY3,ADCY6,Calm1 (includes others),CDKN1B,GNAI2,GNB1,GNG12,GRB2,ITPR1,PIK3C2G,PIK3CD,PRKCE,TUBB2A,UHMK1 |
| Natural Killer Cell Signaling | 2.25 | NA | FCGR2A,GRB2,INPP5B,INPP5D,LAIR1,PIK3C2G,PIK3CD,PRKCE,SYNJ1,SYNJ2 |
| Role of JAK1 and JAK3 in γc Cytokine Signaling | 2.24 | NA | FES,GRB2,JAK2,PIK3C2G,PIK3CD,SOCS3,STAT5A |
| Phagosome Formation | 2.22 | NA | C5AR1,FCGR2A,FN1,ITGA3,PIK3C2G,PIK3CD,PRKCE,RHOBTB1,RHOT1,TLR3 |
| D-myo-inositol (1,3,4)-trisphosphate Biosynthesis | 2.21 | 0 | INPP5B,INPP5D,SYNJ1,SYNJ2 |
| Role of NFAT in Regulation of the Immune Response | 2.21 | 0 | ATF2,Calm1 (includes others),FCGR2A,GNAI2,GNB1,GNG12,GRB2,HLA-A,IKBKB,ITPR1,NFAT5,PIK3C2G,PIK3CD |
| Renin-Angiotensin Signaling | 2.17 | 0 | ADCY3,ADCY6,ATF2,GRB2,ITPR1,JAK2,PIK3C2G,PIK3CD,PRKCE,SHF |
| Protein Ubiquitination Pathway | 2.17 | NA | BAG1,CUL2,DNAJB2,DNAJC12,DNAJC21,DNAJC5,HLA-A,HSPA5,MDM2,PAN2,UBE2M,UBE4A,USP15,USP19,USP34,USP45,XIAP |
| NRF2-mediated Oxidative Stress Response | 2.15 | NA | ACTB,DNAJB2,DNAJC21,DNAJC5,FKBP5,FMO1,GSTM4,Gstm6,KEAP1,MGST1,PIK3C2G,PIK3CD,PRKCE |
| Aryl Hydrocarbon Receptor Signaling | 2.13 | -1.134 | ALDH2,ATR,CDKN1B,GSTM4,Gstm6,MDM2,MGST1,NCOR2,NEDD8,NFIA,TRIP11 |
| Systemic Lupus Erythematosus In B Cell Signaling Pathway | 2.13 | -1.213 | Calm1 (includes others),FCGR2A,GRB2,INPP5B,INPP5D,IRF5,JAK2,MAP4K4,NFAT5,PIK3C2G,PIK3CD,PRKCE,SHF,SYNJ1,SYNJ2,TBK1,TLR3 |
| Thrombin Signaling | 2.11 | 1.508 | ADCY3,ADCY6,F2,GNAI2,GNB1,GNG12,GRB2,IKBKB,ITPR1,PIK3C2G,PIK3CD,PRKCE,RHOBTB1,RHOT1 |
| PDGF Signaling | 2.08 | 0 | GRB2,INPP5B,INPP5D,JAK2,PIK3C2G,PIK3CD,SYNJ1,SYNJ2 |
| Lipid Antigen Presentation by CD1 | 2.08 | NA | AP1G2,CALR,CANX,PSAP |
| 1D-myo-inositol Hexakisphosphate Biosynthesis II (Mammalian) | 2.08 | 0 | INPP5B,INPP5D,SYNJ1,SYNJ2 |
| Iron homeostasis signaling pathway | 2.07 | NA | ATP6V0A1,CP,FBXL5,HBA1/HBA2,HP,JAK2,MMS19,NUBP2,SLC39A14,STAT5A,TF |
| DNA Double-Strand Break Repair by Homologous Recombination | 2.07 | NA | BRCA2,LIG1,RAD50 |
| Calcium Transport I | 2.07 | NA | ATP2A2,ATP2A3,ATP2C1 |
| PPARα/RXRα Activation | 2.06 | 0 | ACVR2B,ADCY3,ADCY6,APOA2,CKAP5,GRB2,IKBKB,IL1R1,JAK2,LPL,MAP4K4,NCOR2,TGFBR1 |
| Caveolar-mediated Endocytosis Signaling | 2.04 | NA | ACTB,ARCN1,FLNA,FLOT2,HLA-A,ITGA3,ITSN1 |
| Virus Entry via Endocytic Pathways | 2.04 | NA | ACTB,AP1G2,FLNA,HLA-A,ITGA3,ITSN1,PIK3C2G,PIK3CD,PRKCE |
| Germ Cell-Sertoli Cell Junction Signaling | 2.03 | NA | ACTB,CTNND1,EPN2,ITGA3,KEAP1,PIK3C2G,PIK3CD,RHOBTB1,RHOT1,SORBS1,TGFBR1,TUBB2A |
| Epithelial Adherens Junction Signaling | 2.01 | NA | ACTB,ACVR2B,CLIP1,CTNND1,EPN2,KEAP1,MET,PARD3,SORBS1,TGFBR1,TUBB2A |
| Huntington's Disease Signaling | 2 | 0 | ATF2,ATP5PF,CAPNS1,DNAJC5,GNB1,GNG12,GRB2,HDAC10,HDAC6,HSPA5,ITPR1,NCOR2,PIK3C2G,PIK3CD,PRKCE |
| Role of NFAT in Cardiac Hypertrophy | 2 | 0.302 | ADCY3,ADCY6,Calm1 (includes others),GNAI2,GNB1,GNG12,GRB2,HDAC10,HDAC6,ITPR1,PIK3C2G,PIK3CD,PRKCE,TGFBR1 |
| iCOS-iCOSL Signaling in T Helper Cells | 1.97 | 0 | Calm1 (includes others),GRB2,HLA-A,IKBKB,INPP5D,ITPR1,NFAT5,PIK3C2G,PIK3CD |
| Actin Cytoskeleton Signaling | 1.96 | -0.905 | ACTB,DIAPH1,F2,FLNA,FN1,GIT1,GNG12,GRB2,ITGA3,LBP,PIK3C2G,PIK3CD,PIP5K1C,TTN |
| FXR/RXR Activation | 1.95 | NA | ABCG8,APOA2,FGA,ITIH4,LPL,NR1H3,PKLR,SAA1,SERPINA1,TF |
| Leptin Signaling in Obesity | 1.95 | 0.447 | ADCY3,ADCY6,GRB2,JAK2,PIK3C2G,PIK3CD,SOCS3 |
| ATM Signaling | 1.95 | -1 | ATF2,ATR,BRAT1,GADD45G,HERC2,HP1BP3,MDM2,RAD50 |
| Glucocorticoid Receptor Signaling | 1.92 | NA | ACTB,ARID1A,BAG1,CEBPA,CREBZF,FKBP5,GRB2,HSPA5,IKBKB,JAK2,NCOR2,NFAT5,NR3C1,PIK3C2G,PIK3CD,SMARCA2,STAT5A,TAT,TGFBR1 |
| Erythropoietin Signaling | 1.92 | NA | GRB2,JAK2,PIK3C2G,PIK3CD,PRKCE,SOCS3,STAT5A |
| IL-3 Signaling | 1.92 | 1.134 | GRB2,INPP5D,JAK2,PIK3C2G,PIK3CD,PRKCE,STAT5A |
| 3-phosphoinositide Degradation | 1.88 | 0.302 | CA3,INPP5B,INPP5D,MTM1,MTMR4,PPIP5K1,SET,SIRPA,SOCS3,SYNJ1,SYNJ2 |
| LPS/IL-1 Mediated Inhibition of RXR Function | 1.87 | 0 | ABCG8,ACOX2,ALAS1,ALDH2,FMO1,GSTM4,Gstm6,HMGCS2,HS3ST6,IL1R1,LBP,MGST1,NR1H2,NR1H3 |
| Antiproliferative Role of Somatostatin Receptor 2 | 1.87 | 0 | CDKN1B,GNB1,GNG12,GUCY2C,NPR2,PIK3C2G,PIK3CD |
| TNFR2 Signaling | 1.86 | NA | IKBKB,TBK1,TNFAIP3,XIAP |
| Gαq Signaling | 1.82 | 1 | Calm1 (includes others),GNB1,GNG12,IKBKB,ITPR1,PIK3C2G,PIK3CD,PLD1,PRKCE,RHOBTB1,RHOT1 |
| fMLP Signaling in Neutrophils | 1.81 | 0.707 | Calm1 (includes others),GNAI2,GNB1,GNG12,ITPR1,NFAT5,PIK3C2G,PIK3CD,PRKCE |
| Cellular Effects of Sildenafil (Viagra) | 1.81 | NA | ACTB,ADCY3,ADCY6,Calm1 (includes others),GUCY2C,ITPR1,PDE1A,PDE4B,PDE4C,PDE5A |
| Apelin Cardiomyocyte Signaling Pathway | 1.78 | 1.414 | ATP2A2,ATP2A3,GNAI2,ITPR1,PIK3C2G,PIK3CD,PRKCE,SLC9A8 |
| RhoA Signaling | 1.77 | -2.121 | ACTB,ARHGAP1,ARHGAP12,NRP2,PIP5K1C,PLD1,RAPGEF6,RHPN2,TTN |
| B Cell Receptor Signaling | 1.77 | -0.905 | ATF2,Calm1 (includes others),FCGR2A,GRB2,IKBKB,INPP5B,INPP5D,NFAT5,PIK3C2G,PIK3CD,SYNJ1,SYNJ2 |
| IL-6 Signaling | 1.77 | 0 | GRB2,IKBKB,IL1R1,JAK2,LBP,MAP4K4,PIK3C2G,PIK3CD,SOCS3 |
| Superpathway of D-myo-inositol (1,4,5)-trisphosphate Metabolism | 1.77 | 0 | INPP5B,INPP5D,SYNJ1,SYNJ2 |
| α-Adrenergic Signaling | 1.76 | 1 | ADCY3,ADCY6,Calm1 (includes others),GNAI2,GNB1,GNG12,ITPR1,PRKCE |
| Thrombopoietin Signaling | 1.74 | 0.816 | GRB2,JAK2,PIK3C2G,PIK3CD,PRKCE,STAT5A |
| Cardiac β-adrenergic Signaling | 1.74 | 0 | ADCY3,ADCY6,ATP2A2,ATP2A3,GNB1,GNG12,PDE1A,PDE4B,PDE4C,PDE5A |
| IGF-1 Signaling | 1.74 | 0.378 | GRB10,GRB2,JAK2,PIK3C2G,PIK3CD,SOCS2,SOCS3,YWHAG |
| Lysine Degradation II | 1.69 | NA | AADAT,AASDH,AASDHPPT |
| FGF Signaling | 1.69 | -1.134 | ATF2,FGFR3,GRB2,ITPR1,MET,PIK3C2G,PIK3CD |
| Calcium-induced T Lymphocyte Apoptosis | 1.65 | 0.816 | ATP2A2,ATP2A3,Calm1 (includes others),HLA-A,ITPR1,PRKCE |
| CXCR4 Signaling | 1.65 | 1.667 | ADCY3,ADCY6,GNAI2,GNB1,GNG12,ITPR1,PIK3C2G,PIK3CD,PRKCE,RHOBTB1,RHOT1 |
| Signaling by Rho Family GTPases | 1.64 | 0 | ACTB,CLIP1,GNAI2,GNB1,GNG12,ITGA3,PARD3,PARD6A,PIK3C2G,PIK3CD,PIP5K1C,PLD1,RHOBTB1,RHOT1 |
| Superpathway of Inositol Phosphate Compounds | 1.62 | -0.277 | CA3,INPP5B,INPP5D,MTMR4,PIK3C2G,PIK3CD,PIP5K1C,PPIP5K1,SET,SIRPA,SOCS3,SYNJ1,SYNJ2 |
| Formaldehyde Oxidation II (Glutathione-dependent) | 1.62 | NA | ADH5,ESD |
| Synaptogenesis Signaling Pathway | 1.61 | 0 | ADCY3,ADCY6,AP1G2,ATF2,Calm1 (includes others),CTNND1,DNAJC5,GRB2,ITPR1,ITSN1,NRXN1,PIK3C2G,PIK3CD,PRKCE,SHF,STXBP1,STXBP6 |
| 3-phosphoinositide Biosynthesis | 1.61 | 0.302 | CA3,INPP5B,MTMR4,PIK3C2G,PIK3CD,PIP5K1C,PPIP5K1,SET,SIRPA,SOCS3,SYNJ1 |
| Endothelin-1 Signaling | 1.6 | 0.577 | ADCY3,ADCY6,GNAI2,GRB2,GUCY2C,ITPR1,PIK3C2G,PIK3CD,PLD1,PRKCE,PTGS1,SHF |
| IL-8 Signaling | 1.57 | 1.265 | GNAI2,GNB1,GNG12,IKBKB,MAP4K4,PIK3C2G,PIK3CD,PLD1,PRKCE,RHOBTB1,RHOT1,TEK |
| IL-10 Signaling | 1.57 | NA | FCGR2A,IKBKB,IL1R1,LBP,MAP4K4,SOCS3 |
| Sirtuin Signaling Pathway | 1.55 | 0 | ACLY,ACSS2,ADAM10,ARNTL,ATG7,ATP5PF,BECN1,GADD45G,GLUD1,HMGCS2,MT-ATP6,NDUFA4,NDUFAF1,NDUFV1,NR1H2,NR1H3,VDAC2 |
| IL-1 Signaling | 1.55 | NA | ADCY3,ADCY6,GNAI2,GNB1,GNG12,IKBKB,IL1R1 |
| Ephrin B Signaling | 1.55 | 2 | ABI1,GNAI2,GNB1,GNG12,HNRNPK,ITSN1 |
| P2Y Purigenic Receptor Signaling Pathway | 1.54 | 0 | ADCY3,ADCY6,ATF2,GNAI2,GNB1,GNG12,PIK3C2G,PIK3CD,PRKCE |
| Th2 Pathway | 1.54 | 0 | ACVR2B,GRB2,HLA-A,JAK2,PIK3C2G,PIK3CD,SOCS3,STAT5A,TGFBR1 |
| Endocannabinoid Developing Neuron Pathway | 1.53 | 0 | ADCY3,ADCY6,ATF2,CDKN1B,GNAI2,GNB1,PIK3C2G,PIK3CD |
| HOTAIR Regulatory Pathway | 1.52 | -1.265 | ATG7,JARID2,KMT2A,MDM2,MET,PIK3C2G,PIK3CD,SETDB1,SUZ12,XIAP |
| Angiopoietin Signaling | 1.47 | 0 | GRB2,IKBKB,PIK3C2G,PIK3CD,STAT5A,TEK |
| Oxidative Phosphorylation | 1.47 | -2.121 | ATP5PF,COX6B1,COX7A2,COX7B,MT-ATP6,NDUFA4,NDUFV1,SURF1 |
| FAK Signaling | 1.46 | NA | ACTB,CAPNS1,GRB2,ITGA3,PIK3C2G,PIK3CD,TNS1 |
| Gap Junction Signaling | 1.46 | NA | ACTB,ADCY3,ADCY6,GNAI2,GRB2,GUCY2C,ITPR1,NPR2,PIK3C2G,PIK3CD,PRKCE,TUBB2A |
| Mitochondrial Dysfunction | 1.46 | NA | ATP5PF,COX6B1,COX7A2,COX7B,MT-ATP6,NDUFA4,NDUFAF1,NDUFV1,OGDH,SURF1,VDAC2 |
| Protein Kinase A Signaling | 1.45 | -0.243 | ADCY3,ADCY6,ATF2,Calm1 (includes others),FLNA,GNAI2,GNB1,GNG12,ITPR1,NFAT5,NTN1,PDE1A,PDE4B,PDE4C,PDE5A,PRKCE,SIRPA,TGFBR1,TTN,YWHAG |
| Sperm Motility | 1.45 | 0.816 | Calm1 (includes others),ERBB4,FES,FGFR3,ITPR1,JAK2,MET,PDE1A,PDE4B,PDE4C,PRKCE,TEK,TNK2 |
| Sphingosine-1-phosphate Signaling | 1.44 | 1.134 | ADCY3,ADCY6,GNAI2,PIK3C2G,PIK3CD,RHOBTB1,RHOT1,SMPD4 |
| Fcγ Receptor-mediated Phagocytosis in Macrophages and Monocytes | 1.42 | -0.378 | ACTB,FCGR2A,INPP5D,PIK3C2G,PLD1,PRKCE,RAB11A |
| Choline Biosynthesis III | 1.42 | NA | CHPT1,PHKA1,PLD1 |
| Role of Tissue Factor in Cancer | 1.42 | NA | ARRB1,F2,FGA,ITGA3,JAK2,PIK3C2G,PIK3CD,STAT5A |
| CREB Signaling in Neurons | 1.41 | 0 | ADCY3,ADCY6,ATF2,Calm1 (includes others),GNAI2,GNB1,GNG12,GRB2,ITPR1,PIK3C2G,PIK3CD,PRKCE |
| Integrin Signaling | 1.41 | 0.632 | ACTB,ARF4,CAPNS1,GIT1,GRB2,ITGA3,PIK3C2G,PIK3CD,RHOBTB1,RHOT1,TNK2,TTN |
| Aldosterone Signaling in Epithelial Cells | 1.41 | 0.447 | DNAJB2,DNAJC12,DNAJC21,DNAJC5,HSPA5,ITPR1,PIK3C2G,PIK3CD,PIP5K1C,PRKCE |
| Neuregulin Signaling | 1.4 | 0.816 | CDKN1B,ERBB4,ERRFI1,GRB2,ITGA3,PRKCE,STAT5A |
| IL-7 Signaling Pathway | 1.4 | -0.816 | CDKN1B,GRB2,MET,PIK3C2G,PIK3CD,STAT5A |
| Semaphorin Signaling in Neurons | 1.4 | NA | ARHGAP1,FES,MET,RHOBTB1,RHOT1 |
| Fc Epsilon RI Signaling | 1.4 | 0 | GRB2,INPP5B,INPP5D,PIK3C2G,PIK3CD,PRKCE,SYNJ1,SYNJ2 |
| Apelin Endothelial Signaling Pathway | 1.4 | 0.707 | ADCY3,ADCY6,Calm1 (includes others),GNAI2,PIK3C2G,PIK3CD,PRKCE,TEK |
| mTOR Signaling | 1.4 | 0.816 | EIF4G1,EIF4G3,PIK3C2G,PIK3CD,PLD1,PRKCE,RHOBTB1,RHOT1,RPS14,RPS15A,RPS19,TSC1 |
| GP6 Signaling Pathway | 1.38 | 0 | ADAM10,Calm1 (includes others),COL27A1,FGA,ITPR1,PIK3C2G,PIK3CD,PRKCE |
| Role of JAK family kinases in IL-6-type Cytokine Signaling | 1.37 | NA | JAK2,SOCS3,STAT5A |
| Lysine Degradation V | 1.37 | NA | AADAT,AASDH,AASDHPPT |
| CD28 Signaling in T Helper Cells | 1.37 | -0.378 | Calm1 (includes others),GRB2,HLA-A,IKBKB,ITPR1,NFAT5,PIK3C2G,PIK3CD |
| SAPK/JNK Signaling | 1.37 | -0.378 | ATF2,GNB1,GRB2,HNRNPK,MAP4K4,PIK3C2G,PIK3CD |
| LPS-stimulated MAPK Signaling | 1.36 | 0.447 | ATF2,IKBKB,LBP,PIK3C2G,PIK3CD,PRKCE |
| PPAR Signaling | 1.35 | 1.134 | GRB2,IKBKB,IL1R1,MAP4K4,NCOR2,NR1H3,STAT5A |
| FLT3 Signaling in Hematopoietic Progenitor Cells | 1.34 | 0 | ATF2,GRB2,INPP5D,PIK3C2G,PIK3CD,STAT5A |
| IL-23 Signaling Pathway | 1.32 | 1 | JAK2,PIK3C2G,PIK3CD,SOCS3 |
| Molecular Mechanisms of Cancer | 1.32 | NA | ADCY3,ADCY6,ATR,CDK9,CDKN1B,CTNND1,GNAI2,GRB2,ITGA3,JAK2,MDM2,PIK3C2G,PIK3CD,PRKCE,RALBP1,RHOBTB1,RHOT1,TGFBR1,XIAP |
| 14-3-3-mediated Signaling | 1.3 | -0.378 | CDKN1B,GRB2,PIK3C2G,PIK3CD,PRKCE,TSC1,TUBB2A,YWHAG |
| CCR3 Signaling in Eosinophils | 1.3 | NA | Calm1 (includes others),GNAI2,GNB1,GNG12,ITPR1,PIK3C2G,PIK3CD,PRKCE |

**Table 15.** Significant pathways for differentially expressed transcripts in ^28^Si vs. non-irradiated control at 4 months analyzed by IPA.

| **Ingenuity Canonical Pathways** | **-log(p-value)** | **z-score** | **Molecules** |
| --- | --- | --- | --- |
| Acute Phase Response Signaling | 10.9 | 1.807 | A2M,AKT3,APCS,APOA2,C1R,C4A/C4B,GRB2,HPX,IL1R1,IL33,ITIH3,ITIH4,JAK2,MAPK9,NR3C1,PIK3CA,PIK3CD,SAA1,Saa3,SERPINA3,SOCS2,SOCS3,STAT3,TF |
| EIF2 Signaling | 5.58 | 2.121 | AKT3,EIF2S3,EIF4G1,EIF4G3,EIF5,GRB2,MT-RNR1,MT-RNR2,MT-TM,MYCN,PABPC1,PIK3CA,PIK3CD,RPL10A,RPL18A,RPL38,RPS14,RPS15A,RPS3 |
| PI3K/AKT Signaling | 5.25 | 2.673 | AKT3,CDKN1B,GRB2,GSK3A,HSP90AB1,INPPL1,ITGA3,JAK2,MDM2,PIK3CA,PIK3CD,SYNJ1,SYNJ2,TSC1 |
| IGF-1 Signaling | 5.04 | 1.265 | AKT3,GRB10,GRB2,IGFBP1,IGFBP2,JAK2,PIK3CA,PIK3CD,PXN,SOCS2,SOCS3,STAT3 |
| Insulin Receptor Signaling | 4.97 | 1.387 | ACLY,AKT3,GRB10,GRB2,GSK3A,INPPL1,JAK2,PIK3CA,PIK3CD,PPP1R10,SOCS3,SYNJ1,SYNJ2,TSC1 |
| IL-9 Signaling | 4.97 | 1.342 | CISH,PIK3CA,PIK3CD,SOCS2,SOCS3,STAT3,STAT5A |
| Growth Hormone Signaling | 4.93 | 1.265 | A2M,CEBPA,JAK2,PIK3CA,PIK3CD,RPS6KA1,SOCS2,SOCS3,STAT3,STAT5A |
| LXR/RXR Activation | 4.89 | 0.832 | APOA2,C4A/C4B,CD14,CD36,HPX,IL1R1,IL33,ITIH4,NR1H2,PON3,RXRB,SAA1,TF |
| PTEN Signaling | 4.89 | -0.277 | AKT3,CDKN1B,FGFR3,GRB2,GSK3A,INPPL1,ITGA3,MAGI1,MAST2,PIK3CA,PIK3CD,SYNJ1,SYNJ2 |
| JAK/Stat Signaling | 4.68 | 1.897 | AKT3,CISH,GRB2,JAK2,PIK3CA,PIK3CD,SOCS2,SOCS3,STAT3,STAT5A |
| Regulation of eIF4 and p70S6K Signaling | 4.45 | 2.236 | AKT3,EIF2S3,EIF4G1,EIF4G3,GRB2,ITGA3,MT-RNR1,MT-RNR2,PABPC1,PIK3CA,PIK3CD,RPS14,RPS15A,RPS3 |
| Chronic Myeloid Leukemia Signaling | 4.43 | NA | AKT3,CDKN1B,E2F3,GRB2,HDAC5,HDAC6,MDM2,PIK3CA,PIK3CD,RBL2,STAT5A |
| IL-4 Signaling | 4.36 | NA | AKT3,GRB2,INPPL1,JAK2,NFAT5,NR3C1,PIK3CA,PIK3CD,SYNJ1,SYNJ2 |
| IL-6 Signaling | 4.32 | 1.508 | A2M,AKT3,CD14,GRB2,IL1R1,IL33,JAK2,MAPK9,PIK3CA,PIK3CD,SOCS3,STAT3 |
| Pancreatic Adenocarcinoma Signaling | 4.13 | 1 | AKT3,CDKN1B,E2F3,GRB2,JAK2,MAPK9,MDM2,PIK3CA,PIK3CD,PLD1,STAT3 |
| HGF Signaling | 4.03 | 1.897 | AKT3,DOCK1,ELF4,GRB2,ITGA3,Map3k7,MAPK9,PIK3CA,PIK3CD,PXN,STAT3 |
| Role of JAK2 in Hormone-like Cytokine Signaling | 3.85 | NA | JAK2,SIRPA,SOCS2,SOCS3,STAT3,STAT5A |
| Prolactin Signaling | 3.74 | 1.414 | GRB2,JAK2,NR3C1,PIK3CA,PIK3CD,SOCS2,SOCS3,STAT3,STAT5A |
| HER-2 Signaling in Breast Cancer | 3.74 | NA | AKT3,CDKN1B,GRB2,GSK3A,MDM2,PARD3,PIK3CA,PIK3CD,TSC1 |
| IL-22 Signaling | 3.65 | 0.447 | AKT3,MAPK9,SOCS3,STAT3,STAT5A |
| Role of JAK family kinases in IL-6-type Cytokine Signaling | 3.56 | NA | JAK2,MAPK9,SOCS3,STAT3,STAT5A |
| GM-CSF Signaling | 3.53 | 2.121 | AKT3,CAMK2G,CISH,GRB2,JAK2,PIK3CA,PIK3CD,STAT3 |
| Acute Myeloid Leukemia Signaling | 3.44 | 1 | AKT3,ARAF,CEBPA,GRB2,PIK3CA,PIK3CD,PML,STAT3,STAT5A |
| HOTAIR Regulatory Pathway | 3.28 | 0 | AKT3,ATXN1,CDH1,DZIP3,KMT2A,MDM2,PIK3CA,PIK3CD,SETDB1,STAT3,STK38,SUZ12 |
| CNTF Signaling | 3.25 | 1.89 | GRB2,JAK2,LIFR,PIK3CA,PIK3CD,RPS6KA1,STAT3 |
| IL-23 Signaling Pathway | 3.22 | 1.633 | AKT3,JAK2,PIK3CA,PIK3CD,SOCS3,STAT3 |
| mTOR Signaling | 3.12 | 0.378 | AKT3,EIF4G1,EIF4G3,MT-RNR1,MT-RNR2,PIK3CA,PIK3CD,PLD1,RPS14,RPS15A,RPS3,RPS6KA1,STK11,TSC1 |
| ErbB2-ErbB3 Signaling | 3.04 | 1.633 | CDKN1B,GRB2,GSK3A,PIK3CA,PIK3CD,STAT3,STAT5A |
| Cancer Drug Resistance By Drug Efflux | 3.04 | NA | ABCC10,AKT3,ARAF,MDM2,PDK1,PIK3CA,PIK3CD |
| Telomerase Signaling | 2.96 | 2.646 | AKT3,ELF4,GRB2,HDAC5,HDAC6,HSP90AB1,PIK3CA,PIK3CD,TEP1 |
| 14-3-3-mediated Signaling | 2.95 | 0 | AKT3,CDKN1B,GRB2,GSK3A,MAPK9,PIK3CA,PIK3CD,RPS6KA1,STK11,TSC1 |
| Paxillin Signaling | 2.91 | 1 | ACTN4,DOCK1,GIT2,GRB2,ITGA3,MAPK9,PIK3CA,PIK3CD,PXN |
| Role of JAK1 and JAK3 in γc Cytokine Signaling | 2.89 | NA | GRB2,JAK2,PIK3CA,PIK3CD,SOCS3,STAT3,STAT5A |
| SPINK1 General Cancer Pathway | 2.89 | 1.134 | AKT3,JAK2,Mt1,Mt2,PIK3CA,PIK3CD,STAT3 |
| LPS/IL-1 Mediated Inhibition of RXR Function | 2.88 | 0.816 | ACSL1,ALDH3A2,CD14,CYP2A6 (includes others),CYP2B6,CYP2C8,FMO1,Gstm3,IL1R1,IL33,MAPK9,MGST1,NR1H2,SLC27A1 |
| Adrenomedullin signaling pathway | 2.85 | 1.387 | AKT3,ARAF,CFH,GRB2,GSK3A,GUCY1B1,GUCY2C,IL33,ITPR1,MAPK9,PIK3CA,PIK3CD,RXRB |
| PXR/RXR Activation | 2.85 | NA | AKT3,ALDH3A2,CYP2A6 (includes others),CYP2B6,CYP2C8,IGFBP1,NR3C1 |
| TR/RXR Activation | 2.81 | NA | AKR1C3,AKT3,COL6A3,MDM2,PIK3CA,PIK3CD,RXRB,TBL1XR1 |
| Adipogenesis pathway | 2.81 | NA | CEBPA,FGFR3,GTF2H1,HDAC5,HDAC6,Kat6b,KAT7,RPS6KA1,SETDB1,TBL1XR1 |
| Systemic Lupus Erythematosus Signaling | 2.79 | NA | AKT3,C6,C8B,CD2BP2,CD72,CREM,GRB2,HNRNPA2B1,IL33,NFAT5,PIK3CA,PIK3CD,PRPF3,PRPF40B |
| PDGF Signaling | 2.78 | 1.414 | GRB2,INPPL1,JAK2,PIK3CA,PIK3CD,STAT3,SYNJ1,SYNJ2 |
| D-myo-inositol (1,4,5)-trisphosphate Degradation | 2.77 | 0 | INPPL1,SEC16A,SYNJ1,SYNJ2 |
| FXR/RXR Activation | 2.76 | NA | AKT3,APOA2,C4A/C4B,HPX,IL33,ITIH4,MAPK9,PON3,SAA1,TF |
| Complement System | 2.69 | NA | C1R,C4A/C4B,C6,C8B,CFH |
| B Cell Receptor Signaling | 2.66 | 1.155 | AKT3,CAMK2G,GRB2,GSK3A,INPPL1,Map3k7,MAPK9,NFAT5,PIK3CA,PIK3CD,SYNJ1,SYNJ2 |
| Inhibition of Angiogenesis by TSP1 | 2.64 | -1 | AKT3,CD36,CD47,GUCY1B1,MAPK9 |
| D-myo-inositol (1,3,4)-trisphosphate Biosynthesis | 2.63 | 0 | INPPL1,SEC16A,SYNJ1,SYNJ2 |
| FAK Signaling | 2.61 | NA | AKT3,DOCK1,GIT2,GRB2,ITGA3,PIK3CA,PIK3CD,PXN |
| Leptin Signaling in Obesity | 2.58 | 1.89 | AKT3,GRB2,JAK2,PIK3CA,PIK3CD,SOCS3,STAT3 |
| EGF Signaling | 2.55 | 2.449 | AKT3,GRB2,ITPR1,PIK3CA,PIK3CD,STAT3 |
| Erythropoietin Signaling | 2.55 | NA | AKT3,GRB2,JAK2,PIK3CA,PIK3CD,SOCS3,STAT5A |
| IL-3 Signaling | 2.55 | 2.646 | AKT3,GRB2,JAK2,PIK3CA,PIK3CD,STAT3,STAT5A |
| ERK/MAPK Signaling | 2.53 | 1.508 | ARAF,DOCK1,ELF4,GRB2,ITGA3,MYCN,PIK3CA,PIK3CD,PPP1R10,PXN,RPS6KA1,STAT3 |
| IL-7 Signaling Pathway | 2.51 | 1.134 | AKT3,CDKN1B,GRB2,GSK3A,PIK3CA,PIK3CD,STAT5A |
| Antiproliferative Role of TOB in T Cell Signaling | 2.49 | NA | CDKN1B,PABPC1,PABPC4,RPS6KA1 |
| 1D-myo-inositol Hexakisphosphate Biosynthesis II (Mammalian) | 2.49 | 0 | INPPL1,SEC16A,SYNJ1,SYNJ2 |
| Aryl Hydrocarbon Receptor Signaling | 2.49 | -1.89 | AIP,ALDH3A2,CDKN1B,Gstm3,HSP90AB1,MDM2,MGST1,NFIB,RBL2,RXRB |
| Mouse Embryonic Stem Cell Pluripotency | 2.47 | 2.121 | AKT3,APC,GRB2,JAK2,LIFR,PIK3CA,PIK3CD,STAT3 |
| FLT3 Signaling in Hematopoietic Progenitor Cells | 2.42 | 1.89 | AKT3,GRB2,PIK3CA,PIK3CD,RPS6KA1,STAT3,STAT5A |
| Docosahexaenoic Acid (DHA) Signaling | 2.41 | NA | AKT3,BID,GSK3A,PIK3CA,PIK3CD |
| Glucocorticoid Receptor Signaling | 2.41 | NA | A2M,AKT3,ANXA1,BAG1,CEBPA,GRB2,GTF2H1,HSP90AB1,JAK2,MAPK9,NFAT5,NR3C1,PIK3CA,PIK3CD,STAT3,STAT5A,TAT |
| Xenobiotic Metabolism Signaling | 2.34 | NA | AIP,ALDH3A2,CAMK2G,CYP2B6,CYP2C8,ESD,FMO1,Gstm3,HDAC5,HSP90AB1,Map3k7,MAPK9,MGST1,PIK3CA,PIK3CD |
| Myc Mediated Apoptosis Signaling | 2.34 | NA | AKT3,BID,GRB2,MAPK9,PIK3CA,PIK3CD |
| IL-17A Signaling in Airway Cells | 2.3 | 1.633 | AKT3,JAK2,MAPK9,PIK3CA,PIK3CD,STAT3 |
| FGF Signaling | 2.28 | 1.89 | AKT3,FGFR3,GRB2,ITPR1,PIK3CA,PIK3CD,STAT3 |
| Thrombopoietin Signaling | 2.27 | 2.449 | GRB2,JAK2,PIK3CA,PIK3CD,STAT3,STAT5A |
| Cell Cycle: G1/S Checkpoint Regulation | 2.27 | 0 | CDKN1B,E2F3,HDAC5,HDAC6,MDM2,RBL2 |
| Glutamine Degradation I | 2.26 | NA | GLS,GLS2 |
| Superpathway of D-myo-inositol (1,4,5)-trisphosphate Metabolism | 2.16 | 0 | INPPL1,SEC16A,SYNJ1,SYNJ2 |
| ILK Signaling | 2.15 | 1.265 | ACTN4,AKT3,CDH1,DOCK1,FLNA,GSK3A,MAPK9,MYH10,PIK3CA,PIK3CD,PXN |
| Regulation of the Epithelial-Mesenchymal Transition Pathway | 2.15 | NA | AKT3,APC,ARAF,CDH1,FGFR3,GRB2,JAK2,PIK3CA,PIK3CD,STAT3,ZEB2 |
| Sertoli Cell-Sertoli Cell Junction Signaling | 2.14 | NA | A2M,ACTN4,AKT3,CDH1,EPB41,EPN2,GSK3A,GUCY1B1,ITGA3,Map3k7,MAPK9 |
| IL-15 Signaling | 2.12 | NA | AKT3,JAK2,PIK3CA,PIK3CD,STAT3,STAT5A |
| Glioma Signaling | 2.12 | 1.342 | AKT3,CAMK2G,E2F3,GRB2,MDM2,PIK3CA,PIK3CD,RBL2 |
| PPARα/RXRα Activation | 2.12 | -2.333 | AIP,APOA2,CD36,CKAP5,CYP2C8,GRB2,HSP90AB1,IL1R1,JAK2,MED12,SLC27A1 |
| Actin Cytoskeleton Signaling | 2.11 | 1.667 | ACTN4,APC,CD14,DOCK1,FLNA,GRB2,ITGA3,MYH10,NCKAP1,PIK3CA,PIK3CD,PXN |
| Prostate Cancer Signaling | 2.1 | NA | AKT3,CDKN1B,GRB2,HSP90AB1,MDM2,PIK3CA,PIK3CD |
| Clathrin-mediated Endocytosis Signaling | 2.1 | NA | APOA2,EPS15,GAK,GRB2,MDM2,PICALM,PIK3CA,PIK3CD,SH3GL1,SYNJ1,TF |
| Role of NANOG in Mammalian Embryonic Stem Cell Pluripotency | 2.1 | 2.646 | AKT3,APC,GRB2,JAK2,LIFR,PIK3CA,PIK3CD,STAT3 |
| Melanoma Signaling | 2.1 | 2 | AKT3,CDH1,MDM2,PIK3CA,PIK3CD |
| Estrogen Biosynthesis | 2.1 | -1.342 | AKR1C3,CYP2A6 (includes others),CYP2B6,CYP2C8,HSD17B10 |
| Glioblastoma Multiforme Signaling | 2.09 | 2.333 | AKT3,APC,CDKN1B,E2F3,GRB2,ITPR1,MDM2,PIK3CA,PIK3CD,TSC1 |
| Small Cell Lung Cancer Signaling | 2.06 | 1.342 | AKT3,BID,CDKN1B,PIK3CA,PIK3CD,RXRB |
| ATM Signaling | 2.06 | -1.633 | BID,HERC2,MAPK9,MDM2,MDM4,SMC2,TRRAP |
| p53 Signaling | 2.03 | -0.378 | AKT3,COQ8A,MDM2,MDM4,PIK3CA,PIK3CD,PML |
| Fc Epsilon RI Signaling | 2.02 | 1.414 | AKT3,GRB2,INPPL1,MAPK9,PIK3CA,PIK3CD,SYNJ1,SYNJ2 |
| TREM1 Signaling | 2.01 | 2.449 | AKT3,GRB2,JAK2,STAT3,STAT5A,TLR1 |
| Germ Cell-Sertoli Cell Junction Signaling | 2.01 | NA | A2M,ACTN4,CDH1,EPN2,ITGA3,Map3k7,MAPK9,PIK3CA,PIK3CD,PXN |
| Non-Small Cell Lung Cancer Signaling | 1.93 | 2.236 | AKT3,GRB2,ITPR1,PIK3CA,PIK3CD,RXRB |
| Epithelial Adherens Junction Signaling | 1.9 | NA | ACTN4,AKT3,APC,CDH1,CLIP1,EPN2,MAGI1,MYH10,PARD3 |
| Phagosome Maturation | 1.87 | NA | ATP6V0A1,ATP6V0C,CANX,CTSC,CTSH,Dync1i2,M6PR,PRDX1,VPS39 |
| VEGF Signaling | 1.86 | 1.633 | ACTN4,AKT3,EIF2S3,GRB2,PIK3CA,PIK3CD,PXN |
| Endometrial Cancer Signaling | 1.79 | 1.342 | AKT3,CDH1,GRB2,PIK3CA,PIK3CD |
| NRF2-mediated Oxidative Stress Response | 1.78 | NA | DNAJB2,DNAJC21,DNAJC5,FMO1,Gstm3,MAPK9,MGST1,PIK3CA,PIK3CD,PRDX1 |
| STAT3 Pathway | 1.77 | -0.378 | CISH,FGFR3,IL1R1,JAK2,MAPK9,SOCS2,SOCS3,STAT3 |
| Type II Diabetes Mellitus Signaling | 1.77 | 0.447 | ACSL1,AKT3,CD36,MAPK9,PIK3CA,PIK3CD,SLC27A1,SOCS2,SOCS3 |
| IL-2 Signaling | 1.76 | 2.236 | AKT3,GRB2,PIK3CA,PIK3CD,STAT5A |
| Estrogen Receptor Signaling | 1.76 | NA | GRB2,GTF2H1,IGFBP1,MED12,MED13L,MED15,NR3C1,TRRAP |
| Nitric Oxide Signaling in the Cardiovascular System | 1.74 | 2.646 | AKT3,GUCY1B1,GUCY2C,HSP90AB1,ITPR1,PIK3CA,PIK3CD |
| Oncostatin M Signaling | 1.72 | 2 | GRB2,JAK2,STAT3,STAT5A |
| CD40 Signaling | 1.71 | 0.447 | MAPK9,PIK3CA,PIK3CD,STAT3,TNFAIP3 |
| Aldosterone Signaling in Epithelial Cells | 1.69 | NA | DNAJB2,DNAJC12,DNAJC2,DNAJC21,DNAJC5,HSP90AB1,ITPR1,PIK3CA,PIK3CD |
| iCOS-iCOSL Signaling in T Helper Cells | 1.66 | 1.633 | AKT3,CAMK2G,GRB2,ITPR1,NFAT5,PIK3CA,PIK3CD |
| Endocannabinoid Developing Neuron Pathway | 1.66 | 0.378 | AKT3,ARAF,CDKN1B,MAPK9,PIK3CA,PIK3CD,STAT3 |
| Role of Macrophages, Fibroblasts and Endothelial Cells in Rheumatoid Arthritis | 1.66 | NA | AKT3,APC,CAMK2G,CEBPA,IL1R1,IL33,JAK2,MAPK9,NFAT5,PIK3CA,PIK3CD,SOCS3,STAT3,TLR1 |
| Regulation of Cellular Mechanics by Calpain Protease | 1.65 | NA | ACTN4,CDKN1B,GRB2,ITGA3,PXN |
| 2-ketoglutarate Dehydrogenase Complex | 1.64 | NA | DLST,OGDH |
| RAR Activation | 1.63 | NA | AKR1C3,AKT3,GTF2H1,JAK2,MAPK9,PIK3CA,PIK3CD,PML,RXRB,STAT5A |
| Th17 Activation Pathway | 1.61 | 1.633 | HSP90AB1,IL1R1,JAK2,NFAT5,SOCS3,STAT3 |
| NGF Signaling | 1.59 | 1.134 | AKT3,GRB2,Map3k7,MAPK9,PIK3CA,PIK3CD,RPS6KA1 |
| Bupropion Degradation | 1.58 | NA | CYP2A6 (includes others),CYP2B6,CYP2C8 |
| Role of Tissue Factor in Cancer | 1.56 | NA | AKT3,ITGA3,JAK2,PIK3CA,PIK3CD,RPS6KA1,STAT5A |
| NF-κB Signaling | 1.54 | 0.333 | AKT3,ARAF,FGFR3,IL1R1,IL33,PIK3CA,PIK3CD,TLR1,TNFAIP3 |
| Cell Cycle: G2/M DNA Damage Checkpoint Regulation | 1.53 | 1 | MDM2,MDM4,RPS6KA1,TRIP12 |
| Natural Killer Cell Signaling | 1.52 | NA | AKT3,GRB2,INPPL1,PIK3CA,PIK3CD,SYNJ1,SYNJ2 |
| IL-10 Signaling | 1.51 | NA | CD14,IL1R1,IL33,SOCS3,STAT3 |
| CD28 Signaling in T Helper Cells | 1.51 | 1.89 | AKT3,GRB2,ITPR1,MAPK9,NFAT5,PIK3CA,PIK3CD |
| Role of p14/p19ARF in Tumor Suppression | 1.5 | NA | MDM2,PIK3CA,PIK3CD |
| PAK Signaling | 1.5 | 0.816 | GRB2,ITGA3,MAPK9,PIK3CA,PIK3CD,PXN |
| Molecular Mechanisms of Cancer | 1.49 | NA | AKT3,APC,BID,CAMK2G,CDH1,CDK9,CDKN1B,E2F3,GRB2,GSK3A,ITGA3,JAK2,MAPK9,MDM2,PIK3CA,PIK3CD |
| Renin-Angiotensin Signaling | 1.48 | 1.633 | GRB2,ITPR1,JAK2,MAPK9,PIK3CA,PIK3CD,STAT3 |
| Neuregulin Signaling | 1.46 | 1 | AKT3,CDKN1B,GRB2,HSP90AB1,ITGA3,STAT5A |
| Protein Ubiquitination Pathway | 1.45 | NA | BAG1,DNAJB2,DNAJC12,DNAJC2,DNAJC21,DNAJC5,HSP90AB1,MDM2,NEDD4L,UBE2M,USP24,USP8 |
| Glutathione Redox Reactions I | 1.43 | NA | GPX1,GPX4,MGST1 |
| Angiopoietin Signaling | 1.43 | NA | AKT3,GRB2,PIK3CA,PIK3CD,STAT5A |
| Ceramide Degradation | 1.43 | NA | ACER2,ASAH1 |
| Systemic Lupus Erythematosus In B Cell Signaling Pathway | 1.42 | 1.732 | AKT3,CD72,GRB2,IL33,INPPL1,JAK2,NFAT5,PIK3CA,PIK3CD,STAT3,SYNJ1,SYNJ2 |
| PPAR Signaling | 1.41 | -1.633 | AIP,GRB2,HSP90AB1,IL1R1,IL33,STAT5A |
| Estrogen-Dependent Breast Cancer Signaling | 1.41 | 2 | AKT3,HSD17B10,PIK3CA,PIK3CD,STAT5A |
| VDR/RXR Activation | 1.39 | NA | CD14,CDKN1B,CEBPA,IGFBP1,RXRB |
| GDNF Family Ligand-Receptor Interactions | 1.39 | 1 | GRB2,ITPR1,MAPK9,PIK3CA,PIK3CD |
| 3-phosphoinositide Degradation | 1.38 | 0 | INPP4A,INPPL1,MTM1,PPP1R16B,SIRPA,SOCS3,SYNJ1,SYNJ2 |
| IL-17 Signaling | 1.37 | NA | AKT3,JAK2,MAPK9,PIK3CA,PIK3CD |
| Superpathway of Inositol Phosphate Compounds | 1.37 | 1.265 | INPPL1,PI4KB,PIK3CA,PIK3CD,PPP1R16B,SEC16A,SIRPA,SOCS3,SYNJ1,SYNJ2 |
| PD-1, PD-L1 cancer immunotherapy pathway | 1.36 | -1.633 | AKT3,CDKN1B,JAK2,PIK3CA,PIK3CD,STAT5A |
| Production of Nitric Oxide and Reactive Oxygen Species in Macrophages | 1.35 | 1 | AKT3,APOA2,JAK2,Map3k7,MAPK9,PIK3CA,PIK3CD,PPP1R10,SIRPA |
| Antiproliferative Role of Somatostatin Receptor 2 | 1.35 | NA | CDKN1B,GUCY1B1,GUCY2C,PIK3CA,PIK3CD |
| Role of IL-17A in Arthritis | 1.35 | NA | MAPK9,PIK3CA,PIK3CD,RPS6KA1 |
| HMGB1 Signaling | 1.34 | 1.134 | AKT3,IL1R1,IL33,Kat6b,KAT7,MAPK9,PIK3CA,PIK3CD |
| Th2 Pathway | 1.33 | 2.449 | GRB2,IL33,JAK2,PIK3CA,PIK3CD,SOCS3,STAT5A |
| Sumoylation Pathway | 1.33 | -1 | CDH1,CEBPA,MAPK9,MDM2,NR3C1,PML |
| Colorectal Cancer Metastasis Signaling | 1.32 | 1.897 | AKT3,APC,CDH1,GRB2,JAK2,MAPK9,MSH3,PIK3CA,PIK3CD,STAT3,TLR1 |
| Tec Kinase Signaling | 1.32 | 1.414 | GTF2I,ITGA3,JAK2,MAPK9,PIK3CA,PIK3CD,STAT3,STAT5A |

**Table 16.** Significant pathways for differentially expressed transcripts in ^28^Si vs. non-irradiated control at 9 months analyzed by IPA.

| **Ingenuity Canonical Pathways** | **-log(p-value)** | **z-score** | **Molecules** |
| --- | --- | --- | --- |
| Acute Phase Response Signaling | 6 | -2.53 | C2,C4A/C4B,CP,HP,IL18,ITIH3,MAPK9,NFKB1,NR3C1,PIK3CD,PIK3CG,SAA1,Saa3,SERPINA1,SERPINE1,TCF3,TF |
| LXR/RXR Activation | 5.1 | 0.302 | C4A/C4B,CD36,IL18,LYZ,MLXIPL,NCOR2,NFKB1,NR1H2,RXRA,S100A8,SAA1,SERPINA1,TF |
| Adipogenesis pathway | 4.18 | NA | ERCC3,FGFR2,FGFR3,HDAC6,HDAC7,KDM1A,KMT2B,PPIP5K1,RPS6KA1,SAP130,SETDB1,SIN3A |
| LPS/IL-1 Mediated Inhibition of RXR Function | 4.08 | -0.707 | ABCB11,ACSL1,ACSL4,ALAS1,CYP2B6,CYP2C8,GSTM4,GSTM5,Gstm6,IL18,LIPC,MAPK9,NR1H2,PPARGC1B,RXRA,SLC27A1 |
| PPARα/RXRα Activation | 3.76 | 1.265 | ADCY4,ADIPOR2,CD36,CKAP5,CYP2C8,HELZ2,HSP90AB1,MED12,MED23,NCOR2,NFKB1,PRKAA1,RXRA,SLC27A1 |
| Germ Cell-Sertoli Cell Junction Signaling | 3.68 | NA | ACTN4,CDH1,CTNND1,EPN2,FNBP1,MAP3K13,MAPK9,MYO7A,PIK3C3,PIK3CD,PIK3CG,PXN,RHOT1 |
| Role of IL-17A in Arthritis | 3.55 | NA | ATF2,MAPK9,NFKB1,PIK3C3,PIK3CD,PIK3CG,RPS6KA1 |
| Small Cell Lung Cancer Signaling | 3.54 | 0.447 | APAF1,MYC,NFKB1,PIK3C3,PIK3CD,PIK3CG,RXRA,SIN3A |
| IL-12 Signaling and Production in Macrophages | 3.51 | NA | IL18,LYZ,MAPK9,NFKB1,PIK3C3,PIK3CD,PIK3CG,PRKD3,RXRA,S100A8,SERPINA1 |
| FXR/RXR Activation | 3.49 | NA | ABCB11,C4A/C4B,FETUB,IL18,LIPC,MAPK9,MLXIPL,RXRA,SAA1,SERPINA1,TF |
| Type II Diabetes Mellitus Signaling | 3.41 | 0.333 | ACSL1,ACSL4,ADIPOR2,CD36,MAPK9,NFKB1,PIK3C3,PIK3CD,PIK3CG,PRKAA1,PRKD3,SLC27A1 |
| Reelin Signaling in Neurons | 3.38 | NA | ARHGEF2,ARHGEF3,ITGAL,MAPK8IP3,MAPK9,PIK3C3,PIK3CD,PIK3CG |
| Xenobiotic Metabolism Signaling | 3.38 | NA | ARNT,CYP2B6,CYP2C8,ESD,GSTM4,GSTM5,Gstm6,HSP90AB1,MAP3K13,MAPK9,NCOR2,NFKB1,PIK3C3,PIK3CD,PIK3CG,PRKD3,RXRA |
| ILK Signaling | 3.27 | -1.387 | ACTN4,ATF2,CDH1,FNBP1,MAPK9,MYC,MYH10,NFKB1,PIK3C3,PIK3CD,PIK3CG,PXN,RHOT1 |
| Apelin Pancreas Signaling Pathway | 3.27 | 0 | MAPK9,NFKB1,PIK3C3,PIK3CD,PIK3CG,PRKAA1 |
| Apelin Endothelial Signaling Pathway | 3.26 | 0.632 | ADCY4,ARNT,Calm1 (includes others),MAPK9,NFKB1,PIK3C3,PIK3CD,PIK3CG,PRKAA1,PRKD3 |
| Production of Nitric Oxide and Reactive Oxygen Species in Macrophages | 3.25 | -1.387 | FNBP1,LYZ,MAP3K13,MAPK9,NFKB1,PIK3C3,PIK3CD,PIK3CG,PRKD3,RHOT1,S100A8,SERPINA1,TYK2 |
| Chronic Myeloid Leukemia Signaling | 3.22 | NA | HDAC6,HDAC7,MECOM,MYC,NFKB1,PIK3C3,PIK3CD,PIK3CG,SIN3A |
| SAPK/JNK Signaling | 3.22 | 0 | ATF2,GNB1,MAP3K13,MAPK8IP3,MAPK9,MINK1,PIK3C3,PIK3CD,PIK3CG |
| Clathrin-mediated Endocytosis Signaling | 3.21 | NA | AP1G2,CD2AP,DNM2,EPS15,LYZ,MYO6,PICALM,PIK3C3,PIK3CD,PIK3CG,S100A8,SERPINA1,TF |
| Aryl Hydrocarbon Receptor Signaling | 3.18 | -1.134 | APAF1,ARNT,GSTM4,GSTM5,Gstm6,HSP90AB1,MYC,NCOR2,NFIX,NFKB1,RXRA |
| IL-8 Signaling | 3.06 | -1.508 | CDH1,FNBP1,GNB1,IRAK3,ITGAV,MAPK9,NFKB1,PIK3C3,PIK3CD,PIK3CG,PLD1,PRKD3,RHOT1 |
| Leukocyte Extravasation Signaling | 3.05 | 0 | ACTN4,CLDN12,CTNND1,ITGAL,MAPK9,PIK3C3,PIK3CD,PIK3CG,PRKD3,PXN,RAPGEF4,SIPA1,TEC |
| Adrenomedullin signaling pathway | 3.03 | 0.277 | ADCY4,ARNT,Calm1 (includes others),CFH,GUCY2C,IL18,MAPK9,NFKB1,PIK3C3,PIK3CD,PIK3CG,RXRA,SHF |
| Pancreatic Adenocarcinoma Signaling | 2.98 | -1.134 | BRCA2,MAPK9,NFKB1,PIK3C3,PIK3CD,PIK3CG,PLD1,SIN3A,TYK2 |
| Nitric Oxide Signaling in the Cardiovascular System | 2.95 | 0.333 | Calm1 (includes others),GUCY2C,HSP90AB1,PDE1A,PIK3C3,PIK3CD,PIK3CG,PRKAA1,PRKD3 |
| TR/RXR Activation | 2.94 | NA | COL6A3,DIO1,HP,NCOR2,PIK3C3,PIK3CD,PIK3CG,RXRA |
| Phospholipase C Signaling | 2.9 | 1.941 | ADCY4,ARHGEF2,ARHGEF3,ATF2,Calm1 (includes others),FCGR2A,FNBP1,GNB1,HDAC6,HDAC7,NFAT5,NFKB1,PLD1,PRKD3,RHOT1 |
| mTOR Signaling | 2.82 | -1.897 | ATG13,EIF4G1,EIF4G3,FNBP1,PIK3C3,PIK3CD,PIK3CG,PLD1,PRKAA1,PRKD3,RHOT1,RPS19,RPS6KA1 |
| Tec Kinase Signaling | 2.77 | -0.632 | FNBP1,GNB1,MAPK9,NFKB1,PIK3C3,PIK3CD,PIK3CG,PRKD3,RHOT1,TEC,TYK2 |
| GP6 Signaling Pathway | 2.65 | 1 | ADAM10,Calm1 (includes others),COL4A1,COL4A5,COL6A3,PIK3C3,PIK3CD,PIK3CG,PRKD3 |
| Molecular Mechanisms of Cancer | 2.62 | NA | ADCY4,APAF1,ARHGEF2,ARHGEF3,CDH1,CDK9,CTNND1,FNBP1,MAPK9,MYC,NFKB1,PIK3C3,PIK3CD,PIK3CG,PRKD3,RHOT1,SIN3A,TCF3,TYK2 |
| Endocannabinoid Cancer Inhibition Pathway | 2.61 | 0 | ADCY4,ATF2,CDH1,MYC,PIK3C3,PIK3CD,PIK3CG,PRKAA1,TCF3,TCF7L2 |
| Renin-Angiotensin Signaling | 2.58 | 0.707 | ADCY4,ATF2,MAPK9,NFKB1,PIK3C3,PIK3CD,PIK3CG,PRKD3,SHF |
| Colorectal Cancer Metastasis Signaling | 2.58 | -0.535 | ADCY4,CDH1,FNBP1,GNB1,MAPK9,MYC,NFKB1,PIK3C3,PIK3CD,PIK3CG,RHOT1,TCF3,TCF7L2,TYK2 |
| LPS-stimulated MAPK Signaling | 2.56 | -0.378 | ATF2,MAPK9,NFKB1,PIK3C3,PIK3CD,PIK3CG,PRKD3 |
| NF-κB Activation by Viruses | 2.53 | -0.378 | ITGAL,ITGAV,NFKB1,PIK3C3,PIK3CD,PIK3CG,PRKD3 |
| IL-23 Signaling Pathway | 2.49 | -0.447 | NFKB1,PIK3C3,PIK3CD,PIK3CG,TYK2 |
| Paxillin Signaling | 2.44 | -0.816 | ACTN4,ITGAL,ITGAV,MAPK9,PIK3C3,PIK3CD,PIK3CG,PXN |
| CD40 Signaling | 2.43 | 0 | MAPK9,NFKB1,PIK3C3,PIK3CD,PIK3CG,TNFAIP3 |
| Myc Mediated Apoptosis Signaling | 2.43 | NA | APAF1,MAPK9,MYC,PIK3C3,PIK3CD,PIK3CG |
| IL-4 Signaling | 2.42 | NA | HLA-A,NFAT5,NR3C1,PIK3C3,PIK3CD,PIK3CG,TYK2 |
| IL-17A Signaling in Airway Cells | 2.4 | -0.816 | MAPK9,NFKB1,PIK3C3,PIK3CD,PIK3CG,TYK2 |
| HOTAIR Regulatory Pathway | 2.39 | -0.632 | CDH1,KDM1A,MYC,NFKB1,PIK3C3,PIK3CD,PIK3CG,SETDB1,TCF3,TCF7L2 |
| Role of p14/p19ARF in Tumor Suppression | 2.39 | 0 | PIK3C3,PIK3CD,PIK3CG,UBTF |
| Huntington's Disease Signaling | 2.38 | 1.134 | APAF1,ATF2,DNM2,GNB1,HDAC6,HDAC7,MAPK9,NCOR2,PIK3C3,PIK3CD,PIK3CG,PRKD3,SIN3A |
| RANK Signaling in Osteoclasts | 2.36 | 0.378 | Calm1 (includes others),MAP3K13,MAPK9,NFKB1,PIK3C3,PIK3CD,PIK3CG |
| P2Y Purigenic Receptor Signaling Pathway | 2.36 | 1 | ADCY4,ATF2,GNB1,MYC,NFKB1,PIK3C3,PIK3CD,PIK3CG,PRKD3 |
| Signaling by Rho Family GTPases | 2.35 | -1.508 | ARHGEF2,ARHGEF3,CDH1,FNBP1,GNB1,MAPK9,NFKB1,PARD3,PIK3C3,PIK3CD,PIK3CG,PLD1,RHOT1 |
| Virus Entry via Endocytic Pathways | 2.34 | NA | AP1G2,DNM2,HLA-A,ITGAL,PIK3C3,PIK3CD,PIK3CG,PRKD3 |
| B Cell Receptor Signaling | 2.34 | 1 | ATF2,Calm1 (includes others),FCGR2A,MAP3K13,MAPK9,NFAT5,NFKB1,PIK3C3,PIK3CD,PIK3CG,TCF3 |
| HGF Signaling | 2.32 | 0 | ATF2,MAP3K13,MAPK9,PIK3C3,PIK3CD,PIK3CG,PRKD3,PXN |
| Gαq Signaling | 2.31 | 0 | Calm1 (includes others),FNBP1,GNB1,NFKB1,PIK3C3,PIK3CD,PIK3CG,PLD1,PRKD3,RHOT1 |
| Acute Myeloid Leukemia Signaling | 2.28 | -0.378 | MYC,NFKB1,PIK3C3,PIK3CD,PIK3CG,TCF3,TCF7L2 |
| PXR/RXR Activation | 2.27 | NA | ABCB11,ALAS1,CYP2B6,CYP2C8,NR3C1,RXRA |
| Systemic Lupus Erythematosus In B Cell Signaling Pathway | 2.26 | 0 | Calm1 (includes others),FCGR2A,IL18,MCL1,MYC,NFAT5,NFKB1,PIK3C3,PIK3CD,PIK3CG,PRKD3,SHF,TBK1,TYK2 |
| Prostate Cancer Signaling | 2.21 | NA | ATF2,HSP90AB1,NFKB1,PIK3C3,PIK3CD,PIK3CG,SIN3A |
| NGF Signaling | 2.19 | 0 | ATF2,MAP3K13,MAPK9,NFKB1,PIK3C3,PIK3CD,PIK3CG,RPS6KA1 |
| eNOS Signaling | 2.19 | 0.378 | ADCY4,AQP8,Calm1 (includes others),DNM2,HSP90AB1,PIK3C3,PIK3CD,PIK3CG,PRKAA1,PRKD3 |
| IL-9 Signaling | 2.18 | 0 | NFKB1,PIK3C3,PIK3CD,PIK3CG |
| Thyroid Cancer Signaling | 2.18 | NA | CDH1,MYC,RXRA,TCF3,TCF7L2 |
| UVB-Induced MAPK Signaling | 2.18 | -0.447 | MAPK9,PIK3C3,PIK3CD,PIK3CG,PRKD3 |
| CXCR4 Signaling | 2.14 | -1.265 | ADCY4,FNBP1,GNB1,MAPK9,PIK3C3,PIK3CD,PIK3CG,PRKD3,PXN,RHOT1 |
| Caveolar-mediated Endocytosis Signaling | 2.13 | NA | DNM2,FLOT1,FLOT2,HLA-A,ITGAL,ITGAV |
| Glioma Invasiveness Signaling | 2.13 | -1.633 | FNBP1,ITGAV,PIK3C3,PIK3CD,PIK3CG,RHOT1 |
| fMLP Signaling in Neutrophils | 2.13 | 1.414 | Calm1 (includes others),GNB1,NFAT5,NFKB1,PIK3C3,PIK3CD,PIK3CG,PRKD3 |
| Macropinocytosis Signaling | 2.1 | 0.447 | ABI1,ACTN4,PIK3C3,PIK3CD,PIK3CG,PRKD3 |
| CD28 Signaling in T Helper Cells | 2.09 | 0.378 | Calm1 (includes others),HLA-A,MAPK9,NFAT5,NFKB1,PIK3C3,PIK3CD,PIK3CG |
| Phagosome Formation | 2.09 | NA | FCGR2A,FNBP1,MARCO,PIK3C3,PIK3CD,PIK3CG,PRKD3,RHOT1 |
| Lymphotoxin β Receptor Signaling | 2.08 | -0.447 | APAF1,NFKB1,PIK3C3,PIK3CD,PIK3CG |
| NF-κB Signaling | 2.07 | 0 | FGFR2,FGFR3,IL18,IRAK3,NFKB1,PIK3C3,PIK3CD,PIK3CG,TBK1,TNFAIP3 |
| Fatty Acid Activation | 2.05 | NA | ACSL1,ACSL4,SLC27A1 |
| Mouse Embryonic Stem Cell Pluripotency | 2.02 | -1.134 | MYC,PIK3C3,PIK3CD,PIK3CG,TCF3,TCF7L2,TYK2 |
| Epithelial Adherens Junction Signaling | 2.02 | NA | ACTN4,CDH1,CTNND1,EPN2,MYH10,MYO7A,PARD3,TCF3,TCF7L2 |
| IL-17 Signaling | 2 | NA | ATF2,MAPK9,NFKB1,PIK3C3,PIK3CD,PIK3CG |
| Thrombin Signaling | 1.97 | -0.632 | ADCY4,ARHGEF2,ARHGEF3,FNBP1,GNB1,NFKB1,PIK3C3,PIK3CD,PIK3CG,PRKD3,RHOT1 |
| Gα12/13 Signaling | 1.97 | 0 | CDH1,MAPK9,NFKB1,PIK3C3,PIK3CD,PIK3CG,PXN,TEC |
| Complement System | 1.96 | -1 | C2,C4A/C4B,CFH,MASP2 |
| Stearate Biosynthesis I (Animals) | 1.95 | 1.342 | ACOT9,ACSL1,ACSL4,ELOVL1,SLC27A1 |
| Role of NFAT in Regulation of the Immune Response | 1.95 | 1.667 | ATF2,Calm1 (includes others),FCGR2A,GNB1,HLA-A,NFAT5,NFKB1,PIK3C3,PIK3CD,PIK3CG |
| Cardiac Hypertrophy Signaling (Enhanced) | 1.94 | 0.447 | ADCY4,ATF2,Calm1 (includes others),DIAPH1,FGFR2,FGFR3,GNB1,HDAC6,HDAC7,IL18,MAP3K13,MAPK9,MYC,NFAT5,NFKB1,PDE1A,PIK3C3,PIK3CD,PIK3CG,PRKD3 |
| Telomerase Signaling | 1.94 | -0.447 | HDAC6,HDAC7,HSP90AB1,MYC,PIK3C3,PIK3CD,PIK3CG |
| Mitochondrial L-carnitine Shuttle Pathway | 1.93 | NA | ACSL1,ACSL4,SLC27A1 |
| CNTF Signaling | 1.92 | -1.342 | PIK3C3,PIK3CD,PIK3CG,RPS6KA1,TYK2 |
| Nur77 Signaling in T Lymphocytes | 1.92 | NA | APAF1,Calm1 (includes others),HLA-A,RXRA,SIN3A |
| Cardiac Hypertrophy Signaling | 1.9 | 0 | ADCY4,ATF2,Calm1 (includes others),FNBP1,GNB1,MAP3K13,MAPK9,PIK3C3,PIK3CD,PIK3CG,RHOT1,RPS6KA1 |
| Prolactin Signaling | 1.9 | -0.447 | MYC,NR3C1,PIK3C3,PIK3CD,PIK3CG,PRKD3 |
| Sumoylation Pathway | 1.89 | -0.447 | CDH1,FNBP1,KDM1A,MAPK9,NFKB1,NR3C1,RHOT1 |
| Osteoarthritis Pathway | 1.89 | 0.333 | ACVRL1,ATF2,FGFR3,MYBBP1A,NFKB1,PRKAA1,S100A8,SIK3,SLC39A8,TCF3,TCF7L2 |
| Proline Degradation | 1.87 | NA | LOC102724788/PRODH,PRODH2 |
| T Cell Receptor Signaling | 1.87 | NA | Calm1 (includes others),NFAT5,NFKB1,PIK3C3,PIK3CD,PIK3CG,TEC |
| Endometrial Cancer Signaling | 1.86 | -0.447 | CDH1,MYC,PIK3C3,PIK3CD,PIK3CG |
| Regulation of the Epithelial-Mesenchymal Transition Pathway | 1.86 | NA | CDH1,FGFR2,FGFR3,NFKB1,PIK3C3,PIK3CD,PIK3CG,TCF3,TCF7L2,TYK2 |
| HMGB1 Signaling | 1.85 | -1.414 | FNBP1,IL18,MAPK9,NFKB1,PIK3C3,PIK3CD,PIK3CG,RHOT1,SERPINE1 |
| Protein Kinase A Signaling | 1.84 | 2.309 | ADCY4,AKAP1,ANAPC5,ATF2,Calm1 (includes others),DUSP3,EYA3,GNB1,MYH10,NFAT5,NFKB1,PDE1A,PRKD3,PTPN21,PXN,TCF3,TCF7L2 |
| Activation of IRF by Cytosolic Pattern Recognition Receptors | 1.83 | -0.447 | ATF2,DDX58,MAPK9,NFKB1,TBK1 |
| Role of Macrophages, Fibroblasts and Endothelial Cells in Rheumatoid Arthritis | 1.81 | NA | ATF2,Calm1 (includes others),IL18,IRAK3,MAPK9,MYC,NFAT5,NFKB1,PIK3C3,PIK3CD,PIK3CG,PRKD3,TCF3,TCF7L2 |
| Glutamate Receptor Signaling | 1.81 | NA | Calm1 (includes others),GNB1,GRIK5,SLC1A4,SLC38A1 |
| Aldosterone Signaling in Epithelial Cells | 1.8 | 0 | DNAJB2,DNAJC12,DNAJC2,HSP90AB1,HSPA4L,PIK3C3,PIK3CD,PIK3CG,PRKD3 |
| FGF Signaling | 1.8 | 0 | ATF2,FGFR2,FGFR3,PIK3C3,PIK3CD,PIK3CG |
| ErbB2-ErbB3 Signaling | 1.78 | -1.342 | MYC,PIK3C3,PIK3CD,PIK3CG,TYK2 |
| γ-linolenate Biosynthesis II (Animals) | 1.77 | NA | ACSL1,ACSL4,SLC27A1 |
| Choline Biosynthesis III | 1.77 | NA | CHPT1,PCYT1A,PLD1 |
| Opioid Signaling Pathway | 1.76 | 0.577 | ADCY4,AP1G2,ATF2,Calm1 (includes others),GNB1,GRK6,MYC,NFKB1,PDE1A,PIK3CG,PRKD3,RPS6KA1 |
| iCOS-iCOSL Signaling in T Helper Cells | 1.76 | 1.134 | Calm1 (includes others),HLA-A,NFAT5,NFKB1,PIK3C3,PIK3CD,PIK3CG |
| Endocannabinoid Developing Neuron Pathway | 1.76 | -0.378 | ADCY4,ATF2,GNB1,MAPK9,PIK3C3,PIK3CD,PIK3CG |
| RAR Activation | 1.75 | NA | ADCY4,ERCC3,MAPK9,NCOR2,NFKB1,PIK3CD,PIK3CG,PRKD3,RXRA,TRIM24 |
| Endothelin-1 Signaling | 1.75 | -0.632 | ADCY4,GUCY2C,MAPK9,MYC,PIK3C3,PIK3CD,PIK3CG,PLD1,PRKD3,SHF |
| Docosahexaenoic Acid (DHA) Signaling | 1.74 | NA | APAF1,PIK3C3,PIK3CD,PIK3CG |
| Sirtuin Signaling Pathway | 1.74 | 0.302 | ACSS2,ADAM10,ATG13,CDH1,CYC1,GLUD1,MYC,NDUFAF1,NDUFV1,NFKB1,NR1H2,PFKM,PRKAA1,SIRT7 |
| Thrombopoietin Signaling | 1.73 | -0.447 | MYC,PIK3C3,PIK3CD,PIK3CG,PRKD3 |
| Fatty Acid β-oxidation I | 1.71 | 1 | ACSL1,ACSL4,SCP2,SLC27A1 |
| Acetate Conversion to Acetyl-CoA | 1.68 | NA | ACSL1,ACSS2 |
| Remodeling of Epithelial Adherens Junctions | 1.68 | NA | ACTN4,CBLL1,CDH1,CTNND1,DNM2 |
| SPINK1 General Cancer Pathway | 1.68 | -0.447 | Mt2,PIK3C3,PIK3CD,PIK3CG,TYK2 |
| IL-15 Production | 1.67 | NA | AATK,DYRK1A,FGFR2,FGFR3,NFKB1,TEC,TYK2 |
| GNRH Signaling | 1.65 | 0.707 | ADCY4,ATF2,Calm1 (includes others),GNB1,MAP3K13,MAPK9,NFKB1,PRKD3,PXN |
| Melanocyte Development and Pigmentation Signaling | 1.64 | 0 | ADCY4,ATF2,PIK3C3,PIK3CD,PIK3CG,RPS6KA1 |
| Bupropion Degradation | 1.63 | NA | CYP2B6,CYP2C8,POR |
| Th1 Pathway | 1.63 | -0.378 | HLA-A,IL18,NFKB1,PIK3C3,PIK3CD,PIK3CG,TYK2 |
| iNOS Signaling | 1.62 | NA | Calm1 (includes others),IRAK3,NFKB1,TYK2 |
| Iron homeostasis signaling pathway | 1.62 | NA | ARNT,ATP6AP1,CP,FLVCR1,HP,TCIRG1,TF,TYK2 |
| Cholecystokinin/Gastrin-mediated Signaling | 1.62 | -1.134 | ATF2,FNBP1,IL18,MAPK9,PRKD3,PXN,RHOT1 |
| IL-15 Signaling | 1.61 | NA | NFKB1,PIK3C3,PIK3CD,PIK3CG,TYK2 |
| IL-6 Signaling | 1.6 | -0.378 | IL18,MAPK9,MCL1,NFKB1,PIK3C3,PIK3CD,PIK3CG |
| p53 Signaling | 1.6 | 0.816 | APAF1,COQ8A,PIK3C3,PIK3CD,PIK3CG,STAG1 |
| Ephrin A Signaling | 1.59 | NA | ADAM10,PIK3C3,PIK3CD,PIK3CG |
| Role of Pattern Recognition Receptors in Recognition of Bacteria and Viruses | 1.59 | 0.378 | DDX58,IL18,MAPK9,NFKB1,PIK3C3,PIK3CD,PIK3CG,PRKD3 |
| Dendritic Cell Maturation | 1.58 | 0.333 | ATF2,FCGR2A,HLA-A,IL18,MAPK9,NFKB1,PIK3C3,PIK3CD,PIK3CG |
| Apoptosis Signaling | 1.58 | 0 | APAF1,LMNA,MCL1,NFKB1,RPS6KA1,SPTAN1 |
| Glucocorticoid Receptor Signaling | 1.57 | NA | BAG1,ERCC3,HSP90AB1,MAPK9,NCOR2,NFAT5,NFKB1,NR3C1,PIK3C3,PIK3CD,PIK3CG,PRKAA1,SERPINE1,TAF1 |
| CREB Signaling in Neurons | 1.57 | 1 | ADCY4,ATF2,Calm1 (includes others),GNB1,GRIK5,PIK3C3,PIK3CD,PIK3CG,PRKD3,RPS6KA1 |
| Integrin Signaling | 1.57 | -1.265 | ACTN4,ARF4,FNBP1,ITGAL,ITGAV,PIK3C3,PIK3CD,PIK3CG,PXN,RHOT1 |
| Growth Hormone Signaling | 1.54 | -0.447 | PIK3C3,PIK3CD,PIK3CG,PRKD3,RPS6KA1 |
| Pyridoxal 5'-phosphate Salvage Pathway | 1.54 | 0.447 | DAPK1,DYRK1A,GRK6,MAPK9,PRKAA1 |
| Relaxin Signaling | 1.52 | 0.378 | ADCY4,GNB1,GUCY2C,NFKB1,PDE1A,PIK3C3,PIK3CD,PIK3CG |
| Neuroinflammation Signaling Pathway | 1.51 | -0.577 | ATF2,BACE1,HLA-A,IL18,IRAK3,MAPK9,NFAT5,NFKB1,PIK3C3,PIK3CD,PIK3CG,TBK1,TYK2 |
| TNFR2 Signaling | 1.51 | NA | NFKB1,TBK1,TNFAIP3 |
| Melanoma Signaling | 1.51 | NA | CDH1,PIK3C3,PIK3CD,PIK3CG |
| NRF2-mediated Oxidative Stress Response | 1.5 | NA | DNAJB2,GSTM4,GSTM5,Gstm6,MAPK9,PIK3C3,PIK3CD,PIK3CG,PRKD3 |
| Role of NFAT in Cardiac Hypertrophy | 1.5 | 0.707 | ADCY4,Calm1 (includes others),GNB1,HDAC6,HDAC7,MAPK9,PIK3C3,PIK3CD,PIK3CG,PRKD3 |
| Apelin Cardiomyocyte Signaling Pathway | 1.49 | -0.447 | ARNT,MAPK9,PIK3C3,PIK3CD,PIK3CG,PRKD3 |
| CD27 Signaling in Lymphocytes | 1.48 | 1 | APAF1,MAP3K13,MAPK9,NFKB1 |
| Estrogen-Dependent Breast Cancer Signaling | 1.48 | 0.447 | ATF2,NFKB1,PIK3C3,PIK3CD,PIK3CG |
| Assembly of RNA Polymerase III Complex | 1.46 | NA | GTF3C1,GTF3C5 |
| Erythropoietin Signaling | 1.46 | NA | NFKB1,PIK3C3,PIK3CD,PIK3CG,PRKD3 |
| Non-Small Cell Lung Cancer Signaling | 1.46 | NA | PIK3C3,PIK3CD,PIK3CG,RXRA,SIN3A |
| Amyotrophic Lateral Sclerosis Signaling | 1.45 | 0 | ALS2,APAF1,GRIK5,PIK3C3,PIK3CD,PIK3CG |
| VEGF Signaling | 1.45 | -0.447 | ACTN4,ARNT,PIK3C3,PIK3CD,PIK3CG,PXN |
| Sertoli Cell-Sertoli Cell Junction Signaling | 1.45 | NA | ACTN4,ATF2,CDH1,CLDN12,EPN2,MAP3K13,MAPK9,MYO7A,SPTAN1 |
| Role of Osteoblasts, Osteoclasts and Chondrocytes in Rheumatoid Arthritis | 1.44 | NA | Calm1 (includes others),IL18,MAPK9,NFAT5,NFKB1,PIK3C3,PIK3CD,PIK3CG,TCF3,TCF7L2 |
| 4-1BB Signaling in T Lymphocytes | 1.44 | NA | ATF2,MAPK9,NFKB1 |
| PD-1, PD-L1 cancer immunotherapy pathway | 1.44 | 0.816 | HLA-A,PDCD4,PIK3C3,PIK3CD,PIK3CG,TYK2 |
| JAK/Stat Signaling | 1.44 | -0.447 | NFKB1,PIK3C3,PIK3CD,PIK3CG,TYK2 |
| IL-7 Signaling Pathway | 1.44 | -0.447 | MCL1,MYC,PIK3C3,PIK3CD,PIK3CG |
| EIF2 Signaling | 1.43 | -0.378 | ATF5,EIF2AK4,EIF4G1,EIF4G3,MYC,PIK3C3,PIK3CD,PIK3CG,RPL18A,RPS19 |
| Antiproliferative Role of Somatostatin Receptor 2 | 1.42 | 0 | GNB1,GUCY2C,PIK3C3,PIK3CD,PIK3CG |
| Neurotrophin/TRK Signaling | 1.42 | -0.447 | ATF2,PIK3C3,PIK3CD,PIK3CG,RPS6KA1 |
| Estrogen Receptor Signaling | 1.41 | NA | ERCC3,MED12,MED23,NCOR2,NR3C1,TAF1,TRRAP |
| PEDF Signaling | 1.4 | -0.447 | NFKB1,PIK3C3,PIK3CD,PIK3CG,TCF7L2 |
| FLT3 Signaling in Hematopoietic Progenitor Cells | 1.38 | -0.447 | ATF2,PIK3C3,PIK3CD,PIK3CG,RPS6KA1 |
| DNA Methylation and Transcriptional Repression Signaling | 1.37 | NA | MTA1,SAP130,SIN3A |
| Human Embryonic Stem Cell Pluripotency | 1.37 | NA | FGFR2,FGFR3,PIK3C3,PIK3CD,PIK3CG,TCF3,TCF7L2 |
| HER-2 Signaling in Breast Cancer | 1.36 | NA | PARD3,PIK3C3,PIK3CD,PIK3CG,PRKD3 |
| Ovarian Cancer Signaling | 1.36 | NA | BRCA2,PIK3C3,PIK3CD,PIK3CG,SIN3A,TCF3,TCF7L2 |
| PI3K Signaling in B Lymphocytes | 1.33 | 1.134 | ATF2,ATF5,Calm1 (includes others),NFAT5,NFKB1,PIK3CD,PIK3CG |
| Rac Signaling | 1.32 | -0.447 | NFKB1,PARD3,PIK3C3,PIK3CD,PIK3CG,PLD1 |
| Salvage Pathways of Pyrimidine Ribonucleotides | 1.32 | 0 | AK4,DAPK1,DYRK1A,GRK6,MAPK9,PRKAA1 |
| Breast Cancer Regulation by Stathmin1 | 1.31 | NA | ADCY4,ARHGEF2,ARHGEF3,Calm1 (includes others),GNB1,PIK3C3,PIK3CD,PIK3CG,PRKD3 |
| MIF-mediated Glucocorticoid Regulation | 1.31 | NA | CD74,NFKB1,NR3C1 |

**Table 17.** Significant pathways for differentially expressed transcripts in ^28^Si vs. non-irradiated control at 12 months analyzed by IPA.

| **^28^Si, 1 month** | | **^28^Si, 2 months** | | **^28^Si, 4 months** | | **^28^Si, 9 months** | | **^28^Si, 12 months** | |
| --- | --- | --- | --- | --- | --- | --- | --- | --- | --- |
| **Module** | **Transcript Ensemble ID** | **Module** | **Transcript Ensemble ID** | **Module** | **Transcript Ensemble ID** | **Module** | **Transcript Ensemble ID** | **Module** | **Transcript Ensemble ID** |
| 10 | ENSMUST00000238749 | 29 | ENSMUST00000235620 | 16 | ENSMUST00000237125 | 19 | ENSMUST00000237305 | 11 | ENSMUST00000238098 |
| 11 | ENSMUST00000237098 | 33 | ENSMUST00000238125 | 1 | ENSMUST00000237749 | 26 | ENSMUST00000237358 | 13 | ENSMUST00000235207 |
| 11 | ENSMUST00000236403 | 35 | ENSMUST00000237742 | 1 | ENSMUST00000236336 | 27 | ENSMUST00000236687 | 17 | ENSMUST00000236414 |
| 26 | ENSMUST00000236171 | 35 | ENSMUST00000236925 | 20 | ENSMUST00000237305 | 2 | ENSMUST00000236504 | 1 | ENSMUST00000238267 |
| 27 | ENSMUST00000236591 | 37 | ENSMUST00000237337 | 28 | ENSMUST00000236046 | 2 | ENSMUST00000238267 | 21 | ENSMUST00000235620 |
| 28 | ENSMUST00000235620 | 37 | ENSMUST00000237529 | 30 | ENSMUST00000236414 | 30 | ENSMUST00000235318 | 22 | ENSMUST00000237798 |
| 29 | ENSMUST00000235647 | 37 | ENSMUST00000237854 | 34 | ENSMUST00000237854 | 30 | ENSMUST00000236414 | 23 | ENSMUST00000236171 |
| 34 | ENSMUST00000237472 | 38 | ENSMUST00000236006 | 36 | ENSMUST00000235620 | 34 | ENSMUST00000235927 | 29 | ENSMUST00000235915 |
| 36 | ENSMUST00000236546 | 3 | ENSMUST00000236950 | 36 | ENSMUST00000237499 | 35 | ENSMUST00000238729 | 29 | ENSMUST00000237823 |
| 3 | ENSMUST00000236292 | 44 | ENSMUST00000235929 | 36 | ENSMUST00000238729 | 36 | ENSMUST00000238021 | 29 | ENSMUST00000238513 |
| 40 | ENSMUST00000236873 | 45 | ENSMUST00000235335 | 36 | ENSMUST00000235411 | 47 | ENSMUST00000236898 | 2 | ENSMUST00000238288 |
| 43 | ENSMUST00000238729 | 45 | ENSMUST00000237478 | 38 | ENSMUST00000237602 | 9 | ENSMUST00000236950 | 2 | ENSMUST00000235318 |
| 44 | ENSMUST00000237337 | 45 | ENSMUST00000237364 | 38 | ENSMUST00000235332 |  |  | 34 | ENSMUST00000235135 |
| 44 | ENSMUST00000236215 | 6 | ENSMUST00000238731 | 44 | ENSMUST00000238021 |  |  | 36 | ENSMUST00000236006 |
| 44 | ENSMUST00000238271 |  |  | 44 | ENSMUST00000238267 |  |  | 40 | ENSMUST00000235648 |
| 47 | ENSMUST00000238288 |  |  | 45 | ENSMUST00000236794 |  |  | 41 | ENSMUST00000238368 |
| 8 | ENSMUST00000237749 |  |  | 48 | ENSMUST00000236850 |  |  | 44 | ENSMUST00000235411 |
| 8 | ENSMUST00000237832 |  |  |  |  |  |  | 45 | ENSMUST00000238021 |
| 9 | ENSMUST00000236824 |  |  |  |  |  |  | 5 | ENSMUST00000238677 |

**Table 18.** Unmapped differentially expressed transcripts (by IPA) in ^28^Si experiments at all time points. Each unmapped ensemble transcript ID is listed with the corresponding module number in SOM Figure 6.

| **Canonical Pathways** | **^56^Fe 1 mo** | **^56^Fe 2 mo** | **^56^Fe 4 mo** | **^56^Fe 9 mo** | **^56^Fe 12 mo** | **^16^O 1 mo** | **^16^O 2 mo** | **^16^O 4 mo** | **^16^O 9 mo** | **^16^O 12 mo** | **^28^Si 1 mo** | **^28^Si 2 mo** | **^28^Si 4 mo** | **^28^Si 9 mo** | **^28^Si 12 mo** |
| --- | --- | --- | --- | --- | --- | --- | --- | --- | --- | --- | --- | --- | --- | --- | --- |
| PI3K Signaling in B Lymphocytes | -1.34 | -0.71 | -2.33 | -1.13 | 1.89 | -0.38 | -2.24 | 1.00 | -1.00 | 2.12 | 0.82 | 0.00 | -0.38 | 1.89 | 1.13 |
| Acute Phase Response Signaling | 0.33 | -1.67 | -1.41 | -1.07 | -1.27 | -1.34 | -1.00 | 0.38 | N/A | 2.20 | -0.82 | -0.45 | -1.50 | 1.81 | -2.53 |
| Synaptogenesis Signaling Pathway | -1.27 | -0.91 | -1.73 | -0.78 | 1.34 | -0.82 | -1.13 | 0.71 | -1.00 | 3.16 | 0.58 | -1.27 | 0.00 | 1.90 | 0.91 |
| Thrombin Signaling | -1.89 | -1.63 | -1.67 | -0.91 | 1.13 | -1.34 | -1.00 | 1.34 | -0.45 | 0.33 | 0.71 | -0.82 | 1.51 | 1.63 | -0.63 |
| Protein Kinase A Signaling | -2.31 | -1.94 | -0.50 | -0.30 | 0.00 | -1.07 | -1.67 | 0.54 | -2.31 | 0.91 | -1.07 | -0.30 | -0.24 | -1.41 | 2.31 |
| 3-phosphoinositide Biosynthesis | -1.41 | -1.00 | 1.63 | 0.00 | 2.65 | 0.00 | 0.00 | 1.89 | 1.34 | 2.12 | 1.63 | 0.45 | 0.30 | 1.89 | 0.45 |
| fMLP Signaling in Neutrophils | -1.00 | -1.63 | -0.82 | -1.13 | 2.24 | -1.00 | -2.24 | N/A | N/A | 1.34 | 1.00 | N/A | 0.71 | 2.00 | 1.41 |
| Gαq Signaling | N/A | -1.63 | -0.33 | 0.00 | 2.12 | -1.13 | -2.24 | 2.24 | N/A | 2.12 | 2.24 | N/A | 1.00 | 1.34 | 0.00 |
| Role of NFAT in Regulation of the Immune Response | N/A | -1.00 | -0.91 | -0.63 | 2.12 | -0.71 | -1.34 | 2.65 | -0.45 | 2.11 | 0.45 | N/A | 0.00 | 1.63 | 1.67 |
| CREB Signaling in Neurons | -0.82 | -0.45 | -1.41 | -0.30 | 2.24 | -1.63 | -2.00 | N/A | -1.13 | 2.65 | 0.00 | -0.82 | 0.00 | 1.13 | 1.00 |
| Opioid Signaling Pathway | -1.27 | 1.41 | -1.94 | -0.83 | 1.67 | -0.38 | -0.38 | 0.38 | -1.41 | 1.63 | 1.00 | 1.13 | 0.58 | -0.82 | 0.58 |
| Superpathway of Inositol Phosphate Compounds | -1.00 | -1.51 | 1.13 | 0.00 | 2.65 | 0.00 | 0.82 | 1.41 | 1.34 | 2.12 | 1.00 | 0.33 | -0.28 | 1.27 | 0.45 |
| D-myo-inositol-5-phosphate Metabolism | -1.34 | -1.00 | 2.00 | 0.82 | 2.24 | -0.30 | 0.00 | 1.34 | N/A | 2.24 | 1.63 | 0.45 | 0.71 | 1.00 | N/A |
| Phospholipase C Signaling | 0.38 | 0.00 | -0.63 | 0.00 | 1.67 | -1.27 | -1.90 | 2.24 | 0.00 | 0.58 | 1.00 | 0.63 | -0.30 | 2.24 | 1.94 |
| Apelin Endothelial Signaling Pathway | -2.00 | -0.45 | -1.63 | -0.58 | 1.41 | -2.00 | -2.12 | N/A | 0.82 | 1.90 | N/A | N/A | 0.71 | 0.45 | 0.63 |
| IL-8 Signaling | N/A | -1.41 | -0.33 | -1.67 | 1.00 | -0.45 | -0.38 | 1.63 | -1.00 | 2.11 | 1.63 | N/A | 1.27 | 0.00 | -1.51 |
| CD28 Signaling in T Helper Cells | N/A | -0.71 | -1.13 | -1.41 | 1.41 | -0.82 | -1.34 | 1.34 | N/A | 2.12 | 1.34 | 0.00 | -0.38 | 1.89 | 0.38 |
| iCOS-iCOSL Signaling in T Helper Cells | N/A | -0.82 | -0.82 | -0.82 | 1.63 | -0.82 | N/A | 2.24 | -1.34 | 1.89 | 0.00 | -1.00 | 0.00 | 1.63 | 1.13 |
| D-myo-inositol (1,4,5,6)-Tetrakisphosphate Biosynthesis | -1.34 | -0.71 | 2.00 | 0.82 | 2.24 | 0.00 | 0.00 | 1.34 | N/A | 2.24 | 1.13 | 0.45 | 0.71 | 1.00 | N/A |
| D-myo-inositol (3,4,5,6)-tetrakisphosphate Biosynthesis | -1.34 | -0.71 | 2.00 | 0.82 | 2.24 | 0.00 | 0.00 | 1.34 | N/A | 2.24 | 1.13 | 0.45 | 0.71 | 1.00 | N/A |
| B Cell Receptor Signaling | -1.63 | 0.00 | -1.21 | -1.39 | 0.63 | 0.00 | -1.13 | 0.71 | -1.27 | 1.94 | -0.63 | 0.33 | -0.91 | 1.16 | 1.00 |
| Role of NFAT in Cardiac Hypertrophy | -1.13 | -1.34 | -0.33 | -0.78 | 1.89 | -1.89 | -0.45 | 0.82 | -0.71 | 1.67 | 0.00 | -0.45 | 0.30 | 1.13 | 0.71 |
| Colorectal Cancer Metastasis Signaling | 1.41 | 0.82 | 0.28 | 0.00 | 0.58 | 0.82 | 1.41 | 0.38 | 0.00 | 2.32 | 2.12 | 0.38 | 0.63 | 1.90 | -0.54 |
| Type II Diabetes Mellitus Signaling | N/A | -0.45 | -0.45 | -2.12 | 0.71 | -1.00 | 0.45 | 2.00 | -1.00 | 1.89 | N/A | 1.00 | 1.34 | 0.45 | 0.33 |
| Adrenomedullin signaling pathway | -0.45 | -1.89 | -0.63 | 0.00 | 1.63 | -2.00 | -1.34 | -0.38 | 0.38 | 2.14 | 0.00 | N/A | -0.63 | 1.39 | 0.28 |
| mTOR Signaling | -1.00 | -0.45 | 0.00 | -1.67 | 0.38 | -0.45 | -1.00 | 2.00 | -1.89 | 0.38 | 0.38 | 0.45 | 0.82 | 0.38 | -1.90 |
| Neuroinflammation Signaling Pathway | 0.91 | -0.73 | -1.41 | -0.71 | -0.91 | 0.63 | -1.00 | 0.30 | 0.38 | 2.36 | 1.94 | 0.00 | 0.28 | 1.00 | -0.58 |
| Cardiac Hypertrophy Signaling (Enhanced) | -1.07 | -0.66 | 0.00 | -0.85 | 1.15 | -0.47 | -0.23 | 0.50 | -2.00 | 1.96 | 1.16 | 0.83 | 0.96 | 0.73 | 0.45 |
| AMPK Signaling | 0.38 | 0.00 | -1.13 | 0.63 | 0.82 | 0.38 | -1.34 | -1.34 | 1.60 | 1.41 | 0.00 | -0.82 | -1.63 | 1.13 | -0.38 |
| Glioblastoma Multiforme Signaling | -0.82 | -1.63 | -0.33 | -0.38 | -0.45 | -0.45 | 0.45 | 1.63 | -0.45 | -0.38 | 1.00 | N/A | 0.71 | 2.33 | -1.89 |
| Aryl Hydrocarbon Receptor Signaling | -1.34 | 0.71 | 0.45 | -1.41 | N/A | -0.82 | -0.71 | 0.82 | 0.00 | 0.30 | 1.34 | -0.71 | -1.13 | -1.89 | -1.13 |
| Integrin Signaling | -1.34 | -1.41 | 0.30 | -1.16 | -0.38 | -0.33 | 0.45 | 0.00 | -0.82 | 0.45 | -0.33 | -1.67 | 0.63 | 2.12 | -1.27 |
| P2Y Purigenic Receptor Signaling Pathway | -0.45 | -1.00 | -0.71 | -1.00 | 2.45 | N/A | N/A | 1.00 | -0.45 | 3.16 | 1.34 | N/A | 0.00 | N/A | 1.00 |
| Nitric Oxide Signaling in the Cardiovascular System | N/A | N/A | -0.82 | 1.00 | 2.00 | -1.00 | 0.00 | 0.45 | 1.63 | 2.00 | N/A | 0.00 | 0.63 | 2.65 | 0.33 |
| 3-phosphoinositide Degradation | -1.63 | -0.33 | 1.63 | 0.00 | 1.89 | -0.58 | 0.82 | 0.38 | 2.00 | 2.24 | 0.71 | 0.00 | 0.30 | 0.00 | N/A |
| White Adipose Tissue Browning Pathway | 1.00 | -1.63 | -1.00 | 0.63 | 1.34 | 1.00 | N/A | N/A | 0.00 | 1.41 | 0.82 | 0.00 | -1.00 | 1.00 | 1.63 |
| Production of Nitric Oxide and Reactive Oxygen Species in Macrophages | -0.38 | -1.39 | 0.00 | -0.63 | 0.30 | 1.00 | -0.38 | 0.00 | 1.13 | 1.73 | 2.31 | 0.33 | 0.33 | 1.00 | -1.39 |
| Dendritic Cell Maturation | -1.34 | -0.33 | -0.91 | -1.89 | 0.71 | 0.45 | 0.00 | 1.63 | 0.00 | 2.32 | 0.82 | 0.00 | 0.71 | 0.82 | 0.33 |
| PI3K/AKT Signaling | -0.45 | -0.30 | 0.00 | 0.38 | -0.63 | 0.38 | 1.13 | 1.13 | 0.82 | 1.27 | 1.67 | 0.00 | 0.83 | 2.67 | -0.45 |
| Apelin Cardiomyocyte Signaling Pathway | -1.63 | -1.41 | -1.34 | -1.41 | 1.00 | N/A | N/A | N/A | N/A | 2.00 | N/A | N/A | 1.41 | 1.34 | -0.45 |
| Mouse Embryonic Stem Cell Pluripotency | 1.13 | -0.82 | -1.67 | 0.00 | 1.13 | 0.00 | N/A | N/A | 1.00 | 1.13 | 1.34 | N/A | -0.45 | 2.12 | -1.13 |
| Systemic Lupus Erythematosus In B Cell Signaling Pathway | -0.38 | 0.00 | -1.29 | -1.16 | 0.63 | 0.63 | -1.13 | -0.30 | -1.27 | 0.00 | 1.00 | -1.13 | -1.21 | 1.73 | 0.00 |
| NF-κB Activation by Viruses | N/A | -1.34 | -0.38 | -1.89 | 0.71 | -0.45 | N/A | N/A | N/A | 2.24 | 1.00 | N/A | 1.34 | 2.00 | -0.38 |
| ILK Signaling | 1.34 | 0.71 | -0.63 | -0.63 | 0.45 | 0.00 | 0.00 | 0.45 | 1.34 | 2.11 | 0.63 | 0.38 | -0.38 | 1.27 | -1.39 |
| Endocannabinoid Cancer Inhibition Pathway | -2.83 | -0.38 | 0.00 | 0.30 | -1.63 | 0.00 | 0.38 | -0.38 | 1.13 | -1.51 | -0.38 | -0.82 | 1.41 | -0.45 | 0.00 |
| Tec Kinase Signaling | N/A | N/A | 1.89 | -0.33 | 0.38 | N/A | 0.45 | 1.00 | 0.45 | 0.71 | 2.24 | -1.00 | 1.00 | 1.41 | -0.63 |
| LXR/RXR Activation | 1.73 | -0.71 | -1.51 | 1.16 | 0.00 | 2.53 | 0.00 | 0.38 | 1.00 | -0.50 | 0.26 | 0.00 | -0.58 | 0.83 | 0.30 |
| Sirtuin Signaling Pathway | -2.00 | 0.83 | 0.00 | 1.00 | -0.63 | -0.38 | -1.67 | 0.00 | 0.91 | -1.73 | 0.28 | -0.28 | 0.00 | 0.91 | 0.30 |
| ERK/MAPK Signaling | -0.82 | -0.38 | -1.00 | -0.28 | -0.45 | -0.38 | 1.00 | -1.00 | 0.00 | 2.00 | 0.33 | -1.00 | 0.00 | 1.51 | -0.71 |
| Osteoarthritis Pathway | 0.45 | 0.71 | 1.41 | 0.82 | 0.38 | 0.71 | 1.41 | -0.63 | N/A | 0.50 | 0.82 | N/A | 0.38 | 2.24 | 0.33 |
| Relaxin Signaling | N/A | N/A | 0.00 | 0.38 | 2.24 | N/A | N/A | 0.82 | 0.00 | 2.33 | 2.00 | N/A | 0.38 | 2.24 | 0.38 |
| 14-3-3-mediated Signaling | -1.34 | -0.71 | -0.45 | -1.27 | 0.45 | -1.34 | 0.00 | N/A | -1.63 | 1.89 | 0.00 | 0.45 | -0.38 | 0.00 | -0.82 |
| cAMP-mediated signaling | 0.45 | N/A | -1.00 | 0.71 | N/A | 0.00 | -2.00 | 0.45 | -1.67 | 2.24 | N/A | -0.45 | -0.33 | -0.45 | 0.82 |
| PPARα/RXRα Activation | 0.33 | -1.67 | -0.33 | -0.69 | 0.71 | 0.58 | 0.45 | 0.00 | 0.91 | 0.28 | -1.00 | 0.00 | 0.00 | -2.33 | 1.27 |
| Ephrin Receptor Signaling | 0.00 | 0.45 | -0.30 | -0.91 | -0.45 | 1.00 | 0.00 | 1.00 | -2.00 | 0.45 | 1.13 | -0.33 | 0.91 | 1.13 | 0.45 |
| RANK Signaling in Osteoclasts | N/A | -1.00 | -1.00 | -0.82 | 0.71 | 0.00 | -0.82 | 1.00 | N/A | 1.90 | 1.00 | 0.00 | -0.45 | 1.34 | 0.38 |
| PKCθ Signaling in T Lymphocytes | N/A | -0.38 | 0.00 | -1.00 | 1.63 | 0.00 | N/A | 1.63 | -0.82 | 2.12 | N/A | N/A | 0.00 | 1.63 | 1.13 |
| Growth Hormone Signaling | N/A | N/A | 1.13 | -1.27 | -0.45 | -1.00 | N/A | 2.24 | -0.38 | 0.00 | 0.00 | 0.00 | 2.12 | 1.27 | -0.45 |
| Signaling by Rho Family GTPases | N/A | -0.71 | 0.38 | -0.82 | 0.82 | N/A | 0.00 | 2.00 | 0.00 | 1.00 | 1.89 | 0.33 | 0.00 | 0.82 | -1.51 |
| Oxidative Phosphorylation | 2.65 | 0.00 | N/A | N/A | N/A | N/A | -0.82 | N/A | 1.63 | N/A | -1.89 | -1.13 | -2.12 | N/A | N/A |
| PEDF Signaling | N/A | -1.34 | -0.71 | -1.63 | 1.63 | 1.00 | N/A | 1.34 | N/A | 1.13 | 0.00 | N/A | 1.00 | N/A | -0.45 |
| eNOS Signaling | N/A | N/A | -0.82 | -0.71 | 1.34 | -0.45 | -1.00 | 0.00 | -0.82 | 1.63 | 0.45 | N/A | -0.38 | 2.00 | 0.38 |
| CXCR4 Signaling | N/A | 0.00 | 0.33 | -0.91 | 0.38 | N/A | N/A | 1.34 | 0.45 | 1.00 | N/A | 1.34 | 1.67 | 1.13 | -1.27 |
| Cardiac Hypertrophy Signaling | -0.45 | 0.00 | 0.00 | 0.00 | 1.34 | -0.91 | -1.13 | 0.71 | -0.33 | 2.11 | 0.82 | 0.71 | -0.30 | 0.82 | 0.00 |
| Huntington's Disease Signaling | -1.00 | 0.00 | 0.00 | -0.38 | 1.13 | N/A | 0.00 | 0.82 | -1.34 | 1.41 | N/A | -0.45 | 0.00 | 1.89 | 1.13 |
| Actin Cytoskeleton Signaling | 0.00 | -0.71 | 0.00 | 0.00 | 0.82 | -0.38 | 0.00 | 0.82 | 1.00 | 0.71 | 0.91 | -1.27 | -0.91 | 1.67 | -0.38 |
| Apelin Adipocyte Signaling Pathway | -1.34 | 0.82 | -2.00 | -0.33 | -1.00 | N/A | N/A | 0.00 | 1.34 | 0.00 | -0.82 | -0.45 | 0.45 | -1.00 | N/A |
| FGF Signaling | N/A | -1.63 | -1.67 | -1.00 | N/A | 0.00 | N/A | N/A | -1.00 | 0.38 | -0.82 | N/A | -1.13 | 1.89 | 0.00 |
| Endothelin-1 Signaling | -1.13 | -0.38 | -1.34 | -1.16 | 0.45 | -0.45 | N/A | 0.45 | -1.41 | 1.13 | 0.00 | N/A | 0.58 | -0.33 | -0.63 |
| Leukocyte Extravasation Signaling | -0.45 | 0.71 | -0.71 | -1.94 | 0.00 | 0.00 | 1.13 | -1.34 | N/A | 1.89 | 0.00 | 0.45 | 0.00 | 0.82 | 0.00 |
| T Cell Exhaustion Signaling Pathway | N/A | 1.00 | 0.33 | 1.00 | -1.34 | 0.45 | N/A | 0.00 | 1.00 | -2.45 | 1.00 | N/A | 0.00 | 0.38 | 0.45 |
| PTEN Signaling | N/A | 1.51 | 0.28 | 1.90 | -0.33 | 0.00 | -0.38 | -1.13 | 1.34 | 0.30 | -0.82 | 0.38 | 0.30 | -0.28 | 0.45 |
| LPS-stimulated MAPK Signaling | -1.00 | -0.38 | -1.13 | -1.89 | 0.00 | 0.00 | 0.00 | 1.00 | N/A | 1.67 | -0.45 | N/A | 0.45 | 1.00 | -0.38 |
| IL-7 Signaling Pathway | N/A | -2.45 | -0.38 | -0.63 | N/A | N/A | N/A | N/A | -0.45 | 0.00 | -1.00 | -2.00 | -0.82 | 1.13 | -0.45 |
| NGF Signaling | 0.00 | 0.71 | -0.71 | -0.38 | 0.71 | 0.38 | 0.00 | 1.63 | -0.45 | 2.83 | 0.33 | 0.00 | 0.00 | 1.13 | 0.00 |
| STAT3 Pathway | N/A | -1.00 | -0.71 | -2.53 | -1.34 | N/A | N/A | N/A | N/A | -1.13 | 0.00 | N/A | 0.82 | -0.38 | -1.34 |
| LPS/IL-1 Mediated Inhibition of RXR Function | 0.63 | 0.38 | 0.00 | 0.45 | 0.00 | 0.33 | 2.00 | -1.13 | 0.45 | 1.16 | 0.00 | 1.13 | 0.00 | 0.82 | -0.71 |
| Role of Pattern Recognition Receptors in Recognition of Bacteria and Viruses | -1.00 | N/A | -1.63 | -1.34 | 1.34 | N/A | 0.00 | N/A | N/A | 1.89 | N/A | N/A | -0.45 | 1.00 | 0.38 |
| Fc Epsilon RI Signaling | -1.00 | 0.00 | -1.67 | -1.90 | 0.45 | N/A | N/A | 0.00 | -1.34 | 0.82 | 0.00 | 0.00 | 0.00 | 1.41 | -0.45 |
| Insulin Receptor Signaling | N/A | -0.38 | -0.33 | -2.11 | -0.38 | 0.00 | 0.00 | 1.13 | -1.41 | -0.38 | -0.45 | -0.71 | -0.30 | 1.39 | N/A |
| Acute Myeloid Leukemia Signaling | N/A | N/A | -0.71 | 0.00 | 2.24 | 1.34 | N/A | -0.82 | -0.45 | 1.13 | N/A | N/A | -0.82 | 1.00 | -0.38 |
| Pancreatic Adenocarcinoma Signaling | N/A | N/A | -0.71 | -0.38 | 0.38 | N/A | 0.45 | N/A | -0.45 | 2.33 | 1.63 | N/A | -0.38 | 1.00 | -1.13 |
| IL-17A Signaling in Airway Cells | N/A | -1.63 | -1.13 | -0.82 | -0.38 | N/A | N/A | N/A | N/A | 1.41 | 0.00 | N/A | 1.00 | 1.63 | -0.82 |
| Th2 Pathway | N/A | N/A | 1.41 | 0.82 | 1.34 | N/A | N/A | N/A | 1.00 | 1.34 | N/A | N/A | 0.00 | 2.45 | -0.45 |
| IL-3 Signaling | N/A | N/A | 0.38 | 0.00 | 1.00 | 0.00 | N/A | N/A | N/A | 1.63 | N/A | -2.00 | 1.13 | 2.65 | 0.00 |
| GP6 Signaling Pathway | N/A | N/A | -0.82 | -1.13 | 1.63 | -0.45 | N/A | N/A | 0.00 | 2.12 | N/A | N/A | 0.00 | 1.63 | 1.00 |
| Cdc42 Signaling | 0.00 | 0.71 | 0.38 | -2.00 | 0.00 | 0.00 | 0.00 | N/A | N/A | 2.00 | -1.00 | 0.00 | 1.63 | -1.00 | N/A |
| Amyotrophic Lateral Sclerosis Signaling | N/A | N/A | 1.34 | -1.00 | 1.63 | N/A | N/A | N/A | -1.00 | 0.82 | -1.00 | 1.34 | -0.45 | 0.00 | 0.00 |
| Cholecystokinin/Gastrin-mediated Signaling | -1.34 | 0.00 | -0.82 | -1.41 | N/A | -1.00 | 0.00 | N/A | N/A | 0.71 | -0.45 | 0.45 | 0.45 | -0.82 | -1.13 |
| PPAR Signaling | 1.00 | -0.38 | 0.00 | -0.71 | -1.00 | 0.82 | 0.00 | 0.00 | N/A | -1.00 | N/A | 0.45 | 1.13 | -1.63 | 0.45 |
| Gα12/13 Signaling | N/A | 0.38 | 0.00 | -1.41 | 0.71 | 0.00 | 0.00 | N/A | -0.45 | 2.33 | 2.24 | N/A | 1.00 | 0.00 | 0.00 |
| Renin-Angiotensin Signaling | N/A | 0.00 | -0.82 | -1.00 | 0.45 | N/A | -1.00 | N/A | 0.00 | 1.89 | -1.00 | N/A | 0.00 | 1.63 | 0.71 |
| EIF2 Signaling | 0.00 | 0.00 | -1.63 | 0.38 | 0.45 | N/A | 0.00 | N/A | N/A | 0.38 | -1.00 | N/A | -2.11 | 2.12 | -0.38 |
| NF-κB Signaling | N/A | -1.51 | -0.91 | -1.39 | 1.13 | -1.00 | 0.38 | 0.00 | -0.38 | 0.78 | N/A | N/A | -0.63 | 0.33 | 0.00 |
| NRF2-mediated Oxidative Stress Response | -0.45 | 0.38 | -0.82 | -1.13 | -1.00 | 0.00 | 0.45 | N/A | N/A | 1.63 | -1.41 | -1.00 | N/A | N/A | N/A |
| CDK5 Signaling | 1.34 | 0.45 | 1.34 | 1.13 | N/A | N/A | N/A | -1.00 | N/A | N/A | 1.00 | -2.00 | N/A | N/A | N/A |
| Endocannabinoid Developing Neuron Pathway | 0.38 | 0.33 | -1.67 | -0.63 | 0.45 | N/A | -0.45 | 0.45 | -0.45 | 0.63 | 0.38 | 1.34 | 0.00 | 0.38 | -0.38 |
| GDNF Family Ligand-Receptor Interactions | N/A | N/A | -1.34 | -1.13 | N/A | N/A | N/A | N/A | -1.00 | 1.34 | -1.00 | N/A | N/A | 1.00 | -1.00 |
| HMGB1 Signaling | N/A | -0.45 | -0.82 | -1.00 | 1.00 | N/A | N/A | 0.45 | 0.45 | 0.63 | N/A | N/A | 0.45 | 1.13 | -1.41 |
| Melanocyte Development and Pigmentation Signaling | 1.00 | 0.00 | -0.82 | -0.33 | N/A | 0.00 | N/A | 1.00 | -0.45 | 2.24 | 0.00 | 0.00 | -0.82 | 1.00 | 0.00 |
| Apoptosis Signaling | N/A | 0.82 | 2.00 | N/A | 0.38 | 1.63 | N/A | -0.82 | -1.00 | 0.00 | N/A | N/A | -1.00 | N/A | 0.00 |
| Neurotrophin/TRK Signaling | N/A | N/A | -1.34 | -0.45 | N/A | N/A | N/A | N/A | -1.00 | 1.34 | -1.00 | 0.00 | -1.00 | 1.00 | -0.45 |
| Type I Diabetes Mellitus Signaling | N/A | -0.82 | N/A | -1.63 | -1.13 | 1.00 | N/A | -0.38 | N/A | 0.00 | 0.45 | N/A | 1.34 | -0.82 | N/A |
| HOTAIR Regulatory Pathway | 1.00 | 0.00 | -0.82 | -0.63 | 0.00 | 0.00 | -0.71 | 0.00 | -1.13 | -0.83 | -0.38 | 0.00 | -1.27 | 0.00 | -0.63 |
| ATM Signaling | N/A | 0.45 | -0.45 | -0.45 | N/A | N/A | 0.00 | N/A | N/A | 2.00 | 0.00 | 1.34 | -1.00 | -1.63 | N/A |
| CD40 Signaling | N/A | -1.63 | -2.12 | -1.13 | -0.82 | N/A | N/A | -0.45 | N/A | 0.71 | N/A | N/A | 0.00 | 0.45 | 0.00 |
| PD-1, PD-L1 cancer immunotherapy pathway | N/A | N/A | -0.38 | 1.13 | 0.00 | N/A | N/A | -1.34 | 0.00 | -1.13 | N/A | N/A | -0.82 | -1.63 | 0.82 |
| Small Cell Lung Cancer Signaling | N/A | -1.34 | -0.82 | 0.00 | 0.38 | N/A | N/A | 1.00 | 0.00 | 1.89 | N/A | N/A | 0.00 | 1.34 | 0.45 |
| Pyridoxal 5'-phosphate Salvage Pathway | N/A | 0.00 | 1.00 | 1.34 | 0.00 | N/A | 1.34 | 1.00 | N/A | N/A | 1.00 | 1.00 | N/A | N/A | 0.45 |
| Fcγ Receptor-mediated Phagocytosis in Macrophages and Monocytes | N/A | N/A | 0.00 | -1.63 | 1.63 | N/A | -1.00 | N/A | N/A | N/A | 2.00 | N/A | -0.38 | 0.00 | -0.45 |
| Role of NANOG in Mammalian Embryonic Stem Cell Pluripotency | 1.00 | N/A | -0.45 | 0.00 | N/A | N/A | N/A | N/A | 0.45 | 1.00 | 0.45 | N/A | 0.00 | 2.65 | -1.00 |
| Prolactin Signaling | N/A | N/A | 0.82 | -1.39 | -0.45 | N/A | N/A | N/A | 0.00 | 0.00 | N/A | -1.00 | 1.41 | 1.41 | -0.45 |
| FLT3 Signaling in Hematopoietic Progenitor Cells | N/A | 0.00 | -1.00 | 0.00 | N/A | 0.00 | N/A | N/A | 0.00 | 2.00 | -0.45 | -1.13 | 0.00 | 1.89 | -0.45 |
| BMP signaling pathway | 0.45 | -0.45 | -2.00 | -0.82 | N/A | N/A | -1.00 | N/A | N/A | 1.34 | 0.82 | N/A | N/A | N/A | N/A |
| PAK Signaling | N/A | N/A | 0.82 | -1.41 | -1.00 | N/A | N/A | N/A | N/A | 1.00 | N/A | N/A | 0.45 | 0.82 | -1.34 |
| GNRH Signaling | -0.38 | 0.00 | 0.00 | -0.58 | 0.38 | 0.00 | 0.38 | -0.45 | -0.82 | 1.67 | 0.38 | 0.38 | 0.71 | 0.00 | 0.71 |
| tRNA Charging | N/A | N/A | N/A | N/A | 1.34 | N/A | N/A | N/A | N/A | N/A | -2.45 | -2.00 | N/A | N/A | -1.00 |
| Sphingosine-1-phosphate Signaling | -2.00 | N/A | -0.82 | 0.00 | 0.00 | N/A | N/A | 1.13 | N/A | 0.00 | N/A | N/A | 1.13 | N/A | -1.63 |
| Gαs Signaling | N/A | N/A | -0.82 | 0.00 | N/A | N/A | N/A | 0.00 | N/A | 2.45 | 1.00 | -0.45 | 0.00 | N/A | 2.00 |
| SAPK/JNK Signaling | N/A | 0.45 | 0.00 | -1.13 | 0.00 | 0.00 | N/A | 0.00 | -1.34 | 1.67 | 0.38 | 0.82 | -0.38 | 0.45 | 0.00 |
| JAK/Stat Signaling | N/A | N/A | 0.00 | -0.63 | -0.71 | N/A | N/A | 1.00 | 0.45 | 0.30 | 0.45 | N/A | 0.71 | 1.90 | -0.45 |
| Wnt/β-catenin Signaling | N/A | 0.82 | 0.00 | N/A | 0.00 | 1.41 | 2.00 | -1.34 | N/A | 0.00 | N/A | N/A | N/A | 0.00 | -1.00 |
| PDGF Signaling | N/A | N/A | 0.38 | 0.00 | N/A | N/A | N/A | 0.00 | 1.00 | 1.34 | 0.00 | -1.00 | 0.00 | 1.41 | -1.34 |
| EGF Signaling | N/A | N/A | -1.63 | -0.38 | N/A | N/A | N/A | N/A | N/A | 2.00 | N/A | N/A | 0.00 | 2.45 | N/A |
| Antioxidant Action of Vitamin C | 1.00 | 0.38 | -0.82 | N/A | 0.00 | 0.00 | N/A | N/A | N/A | -1.63 | -1.63 | N/A | -1.00 | 0.00 | N/A |
| Calcium Signaling | -1.00 | 0.00 | -2.45 | 0.45 | N/A | -0.45 | N/A | N/A | -0.45 | N/A | N/A | 0.82 | -0.82 | N/A | N/A |
| Estrogen-Dependent Breast Cancer Signaling | N/A | N/A | -1.00 | -0.45 | N/A | N/A | N/A | N/A | N/A | 2.45 | N/A | N/A | 0.00 | 2.00 | 0.45 |
| Rac Signaling | N/A | -1.00 | 0.00 | -1.34 | 1.00 | N/A | -0.45 | N/A | N/A | 1.63 | N/A | N/A | 0.00 | 0.45 | -0.45 |
| Lymphotoxin β Receptor Signaling | N/A | -1.00 | -0.82 | N/A | 0.82 | N/A | N/A | 1.00 | N/A | 2.24 | N/A | N/A | N/A | N/A | -0.45 |
| Sumoylation Pathway | -0.82 | N/A | N/A | N/A | N/A | N/A | N/A | -2.00 | N/A | 0.00 | 0.00 | 0.00 | -1.89 | -1.00 | -0.45 |
| p53 Signaling | N/A | N/A | 0.82 | 1.34 | 0.38 | N/A | N/A | 0.45 | 0.45 | -1.13 | N/A | N/A | 0.38 | -0.38 | 0.82 |
| Th17 Activation Pathway | N/A | -1.00 | N/A | 1.00 | 1.00 | N/A | N/A | N/A | N/A | 0.00 | N/A | N/A | -1.00 | 1.63 | -0.45 |
| Glioma Signaling | N/A | N/A | -1.63 | -1.13 | N/A | N/A | N/A | N/A | -1.00 | N/A | N/A | N/A | -0.45 | 1.34 | 0.45 |
| IL-6 Signaling | N/A | -0.82 | -0.38 | -0.83 | -0.33 | N/A | N/A | 0.00 | N/A | 1.50 | 0.00 | N/A | 0.00 | 1.51 | -0.38 |
| RhoA Signaling | 0.00 | -0.38 | N/A | N/A | N/A | 0.45 | -0.38 | -0.45 | N/A | N/A | 1.34 | -0.63 | -2.12 | N/A | N/A |
| Salvage Pathways of Pyrimidine Ribonucleotides | N/A | -0.38 | 0.45 | 0.38 | 0.00 | N/A | 0.82 | 1.34 | N/A | 1.34 | 1.00 | 0.00 | N/A | N/A | 0.00 |
| Endometrial Cancer Signaling | N/A | N/A | -1.00 | -0.82 | N/A | N/A | N/A | N/A | N/A | 2.00 | N/A | N/A | N/A | 1.34 | -0.45 |
| Thrombopoietin Signaling | N/A | N/A | 0.82 | 0.00 | N/A | N/A | N/A | N/A | N/A | 1.00 | N/A | N/A | 0.82 | 2.45 | -0.45 |
| VEGF Signaling | N/A | N/A | -0.45 | 0.00 | 0.00 | N/A | N/A | N/A | N/A | 2.00 | N/A | N/A | -1.00 | 1.63 | -0.45 |
| ErbB Signaling | N/A | N/A | -0.82 | -1.41 | N/A | N/A | N/A | N/A | N/A | 1.34 | N/A | N/A | 0.45 | 1.00 | -0.45 |
| Telomerase Signaling | N/A | N/A | -1.34 | 0.00 | N/A | N/A | N/A | N/A | 1.00 | N/A | N/A | N/A | 0.00 | 2.65 | -0.45 |
| UVB-Induced MAPK Signaling | N/A | N/A | -1.34 | -1.63 | N/A | N/A | N/A | N/A | N/A | 2.00 | N/A | N/A | N/A | N/A | -0.45 |
| Corticotropin Releasing Hormone Signaling | -0.45 | N/A | -0.82 | 0.33 | N/A | N/A | N/A | 0.00 | 1.00 | 1.00 | -0.45 | N/A | 0.38 | N/A | 1.00 |
| GM-CSF Signaling | N/A | N/A | 0.00 | -0.71 | 0.00 | N/A | N/A | N/A | -0.45 | 1.63 | N/A | N/A | -0.45 | 2.12 | N/A |
| Superpathway of Cholesterol Biosynthesis | -2.45 | N/A | N/A | N/A | N/A | -1.89 | N/A | N/A | N/A | N/A | -1.00 | N/A | N/A | N/A | N/A |
| Death Receptor Signaling | N/A | -1.34 | 1.00 | N/A | 0.00 | 0.45 | N/A | -0.38 | N/A | 0.82 | N/A | N/A | 0.82 | N/A | 0.45 |
| Nicotine Degradation II | -1.34 | N/A | 0.00 | -0.45 | -1.00 | 0.00 | N/A | N/A | 0.00 | N/A | -0.38 | N/A | N/A | -1.00 | 1.00 |
| p38 MAPK Signaling | 0.00 | N/A | -1.00 | 0.00 | N/A | 0.00 | N/A | -1.00 | N/A | 0.71 | -0.38 | N/A | -1.00 | N/A | -1.00 |
| HGF Signaling | N/A | 0.00 | 0.00 | -0.30 | 0.38 | 0.00 | 0.00 | N/A | 0.00 | 2.12 | N/A | 0.00 | -0.38 | 1.90 | 0.00 |
| UVC-Induced MAPK Signaling | -2.00 | N/A | N/A | -2.00 | N/A | N/A | 1.00 | N/A | N/A | N/A | N/A | N/A | N/A | N/A | N/A |
| Triacylglycerol Biosynthesis | -2.00 | N/A | N/A | N/A | N/A | 0.00 | N/A | N/A | -2.00 | N/A | -1.00 | N/A | N/A | N/A | N/A |
| Melanoma Signaling | N/A | N/A | -0.45 | -0.45 | N/A | N/A | N/A | N/A | 1.00 | 1.00 | N/A | N/A | N/A | 2.00 | N/A |
| IL-23 Signaling Pathway | N/A | N/A | 0.00 | -0.45 | 0.45 | N/A | N/A | N/A | N/A | 0.82 | N/A | N/A | 1.00 | 1.63 | -0.45 |
| Acetone Degradation I (to Methylglyoxal) | -2.00 | N/A | N/A | -1.34 | N/A | -1.00 | N/A | N/A | N/A | N/A | -0.45 | N/A | N/A | N/A | N/A |
| Ephrin B Signaling | 0.00 | N/A | -1.00 | -0.45 | N/A | 0.00 | N/A | N/A | N/A | N/A | 1.34 | N/A | 2.00 | N/A | N/A |
| IGF-1 Signaling | N/A | N/A | 0.00 | -0.91 | -0.82 | N/A | N/A | N/A | 0.00 | 0.38 | N/A | N/A | 0.38 | 1.27 | -1.00 |
| SPINK1 General Cancer Pathway | -1.00 | N/A | 0.00 | -0.82 | N/A | N/A | N/A | N/A | N/A | 0.00 | N/A | N/A | 1.34 | 1.13 | -0.45 |
| April Mediated Signaling | N/A | -0.38 | -1.00 | N/A | 0.00 | -1.00 | N/A | 1.00 | N/A | 1.34 | 0.00 | N/A | N/A | N/A | N/A |
| B Cell Activating Factor Signaling | N/A | -0.38 | -1.00 | N/A | 0.00 | -1.00 | N/A | 1.00 | N/A | 1.34 | 0.00 | N/A | N/A | N/A | N/A |
| PCP pathway | N/A | N/A | -2.24 | N/A | N/A | N/A | N/A | N/A | N/A | 2.00 | 0.45 | N/A | N/A | N/A | N/A |
| Wnt/Ca+ pathway | N/A | 0.00 | -2.24 | N/A | N/A | -1.00 | N/A | N/A | N/A | 1.34 | 0.00 | N/A | N/A | N/A | N/A |
| Role of RIG1-like Receptors in Antiviral Innate Immunity | N/A | -1.34 | 1.00 | N/A | 0.45 | 0.45 | N/A | 0.82 | N/A | 0.45 | N/A | N/A | N/A | N/A | N/A |
| ErbB2-ErbB3 Signaling | N/A | N/A | 0.45 | 0.00 | N/A | N/A | N/A | N/A | 1.00 | N/A | N/A | N/A | 0.00 | 1.63 | -1.34 |
| Cardiac β-adrenergic Signaling | N/A | N/A | 1.00 | 1.00 | N/A | N/A | N/A | -0.38 | -1.00 | N/A | N/A | -1.00 | 0.00 | N/A | N/A |
| Cell Cycle: G1/S Checkpoint Regulation | N/A | N/A | N/A | -1.00 | N/A | N/A | 0.00 | N/A | -0.45 | 0.45 | N/A | -0.82 | 1.63 | 0.00 | N/A |
| IL-9 Signaling | N/A | N/A | 1.00 | 0.00 | N/A | N/A | N/A | N/A | N/A | 1.00 | N/A | N/A | 1.00 | 1.34 | 0.00 |
| Paxillin Signaling | N/A | N/A | 0.45 | -0.63 | -1.00 | N/A | N/A | N/A | N/A | N/A | N/A | N/A | -0.45 | 1.00 | -0.82 |
| Inhibition of Angiogenesis by TSP1 | N/A | N/A | N/A | -2.24 | N/A | N/A | N/A | N/A | N/A | 1.00 | N/A | N/A | N/A | -1.00 | N/A |
| Activation of IRF by Cytosolic Pattern Recognition Receptors | N/A | 0.38 | 0.45 | N/A | -0.82 | 0.45 | 0.00 | 0.45 | N/A | 0.71 | 0.45 | 0.00 | N/A | N/A | -0.45 |
| Agrin Interactions at Neuromuscular Junction | N/A | N/A | 0.45 | -1.34 | -1.34 | N/A | N/A | N/A | N/A | N/A | N/A | N/A | N/A | N/A | -1.00 |
| Non-Small Cell Lung Cancer Signaling | N/A | N/A | -1.34 | -0.45 | N/A | N/A | N/A | N/A | N/A | N/A | N/A | N/A | 0.00 | 2.24 | N/A |
| Oncostatin M Signaling | N/A | N/A | 1.00 | 1.00 | N/A | N/A | N/A | N/A | N/A | N/A | N/A | N/A | N/A | 2.00 | N/A |
| IL-2 Signaling | N/A | N/A | -0.38 | -0.38 | N/A | N/A | N/A | N/A | 0.00 | 1.00 | N/A | N/A | 0.00 | 2.24 | N/A |
| Th1 Pathway | N/A | N/A | N/A | -0.71 | -0.45 | N/A | N/A | N/A | 0.00 | 0.00 | N/A | N/A | 0.82 | 1.63 | -0.38 |
| VEGF Family Ligand-Receptor Interactions | N/A | N/A | -1.00 | -0.82 | N/A | N/A | N/A | N/A | N/A | N/A | N/A | N/A | 0.00 | 2.00 | 0.00 |
| Stearate Biosynthesis I (Animals) | N/A | N/A | N/A | -1.63 | N/A | N/A | N/A | N/A | -0.82 | N/A | N/A | N/A | N/A | N/A | 1.34 |
| TNFR2 Signaling | N/A | -0.45 | 1.00 | N/A | 1.00 | N/A | N/A | N/A | N/A | 1.34 | N/A | N/A | N/A | N/A | N/A |
| CNTF Signaling | N/A | N/A | 0.00 | 0.00 | N/A | 0.00 | N/A | N/A | 0.45 | N/A | N/A | N/A | 0.00 | 1.89 | -1.34 |
| TGF-β Signaling | N/A | N/A | -1.00 | -1.00 | N/A | N/A | N/A | N/A | N/A | 1.63 | N/A | N/A | N/A | N/A | N/A |
| CCR3 Signaling in Eosinophils | -1.00 | N/A | -1.00 | -1.63 | N/A | N/A | N/A | N/A | N/A | N/A | N/A | N/A | N/A | N/A | 0.00 |
| Chemokine Signaling | -1.00 | N/A | -1.00 | -1.63 | N/A | N/A | N/A | N/A | N/A | N/A | N/A | N/A | N/A | N/A | N/A |
| Superpathway of Geranylgeranyldiphosphate Biosynthesis I (via Mevalonate) | -2.24 | N/A | N/A | N/A | N/A | -1.34 | N/A | N/A | N/A | N/A | N/A | N/A | N/A | N/A | N/A |
| CD27 Signaling in Lymphocytes | N/A | 0.45 | N/A | N/A | 0.00 | 0.00 | 0.00 | 0.45 | N/A | 1.63 | N/A | N/A | N/A | N/A | 1.00 |
| iNOS Signaling | N/A | -1.34 | N/A | N/A | 0.00 | N/A | N/A | N/A | N/A | 0.82 | 1.34 | N/A | N/A | N/A | N/A |
| Toll-like Receptor Signaling | N/A | -0.82 | -1.00 | N/A | N/A | N/A | N/A | 0.00 | N/A | 0.63 | N/A | N/A | -1.00 | N/A | N/A |
| TNFR1 Signaling | N/A | 0.45 | 0.00 | N/A | 1.00 | 0.00 | N/A | 0.00 | N/A | 2.00 | 0.00 | N/A | N/A | N/A | N/A |
| TWEAK Signaling | N/A | -1.00 | N/A | N/A | -0.45 | -1.00 | N/A | -1.00 | N/A | N/A | N/A | N/A | N/A | N/A | N/A |
| Angiopoietin Signaling | N/A | 0.45 | 0.00 | 1.00 | 1.00 | -1.00 | N/A | N/A | N/A | N/A | N/A | N/A | 0.00 | N/A | N/A |
| Glioma Invasiveness Signaling | N/A | N/A | -0.45 | -1.34 | N/A | N/A | N/A | N/A | N/A | N/A | N/A | N/A | N/A | N/A | -1.63 |
| FcγRIIB Signaling in B Lymphocytes | N/A | N/A | N/A | -0.38 | N/A | N/A | N/A | N/A | N/A | N/A | N/A | -1.00 | 0.00 | 1.00 | -1.00 |
| Mevalonate Pathway I | -2.00 | N/A | N/A | N/A | N/A | -1.34 | N/A | N/A | N/A | N/A | N/A | N/A | N/A | N/A | N/A |
| TREM1 Signaling | N/A | N/A | 0.00 | N/A | N/A | N/A | N/A | N/A | N/A | 0.82 | N/A | N/A | 0.00 | 2.45 | N/A |
| 4-1BB Signaling in T Lymphocytes | N/A | 0.00 | -1.00 | N/A | 0.00 | N/A | N/A | N/A | N/A | 2.24 | 0.00 | N/A | N/A | N/A | N/A |
| Leptin Signaling in Obesity | N/A | N/A | -0.45 | 0.00 | 0.00 | N/A | N/A | N/A | N/A | 0.45 | N/A | N/A | 0.45 | 1.89 | N/A |
| Estrogen Biosynthesis | N/A | N/A | N/A | -1.00 | N/A | 0.00 | N/A | N/A | N/A | N/A | -0.82 | N/A | N/A | -1.34 | N/A |
| Regulation of eIF4 and p70S6K Signaling | N/A | N/A | -0.45 | -0.45 | N/A | N/A | N/A | N/A | N/A | N/A | N/A | N/A | 0.00 | 2.24 | N/A |
| ERK5 Signaling | N/A | 1.00 | -1.00 | N/A | N/A | N/A | 0.00 | N/A | -1.00 | N/A | 0.00 | N/A | N/A | N/A | N/A |
| UVA-Induced MAPK Signaling | N/A | N/A | -1.00 | -1.00 | N/A | N/A | N/A | N/A | N/A | N/A | N/A | N/A | N/A | N/A | -1.00 |
| Neuregulin Signaling | -0.45 | N/A | 0.00 | -0.45 | N/A | N/A | N/A | N/A | N/A | N/A | N/A | N/A | 0.82 | 1.00 | N/A |
| Macropinocytosis Signaling | N/A | N/A | N/A | -1.63 | N/A | N/A | N/A | N/A | N/A | N/A | N/A | N/A | 0.45 | N/A | 0.45 |
| Induction of Apoptosis by HIV1 | N/A | -0.45 | N/A | N/A | 0.00 | N/A | 1.00 | -1.00 | N/A | 0.00 | N/A | N/A | N/A | N/A | N/A |
| Nicotine Degradation III | N/A | N/A | N/A | -1.00 | N/A | 0.00 | N/A | N/A | N/A | N/A | -0.45 | N/A | N/A | N/A | 1.00 |
| Aldosterone Signaling in Epithelial Cells | N/A | -1.00 | N/A | -1.00 | N/A | N/A | N/A | N/A | N/A | N/A | N/A | N/A | 0.45 | N/A | 0.00 |
| HIPPO signaling | 1.00 | N/A | N/A | N/A | N/A | 0.00 | N/A | N/A | 1.00 | 0.00 | 0.45 | N/A | N/A | 0.00 | N/A |
| Antiproliferative Role of Somatostatin Receptor 2 | N/A | N/A | 0.00 | -1.00 | N/A | N/A | N/A | N/A | -1.00 | 0.45 | N/A | N/A | 0.00 | N/A | 0.00 |
| MIF Regulation of Innate Immunity | N/A | N/A | N/A | N/A | N/A | N/A | N/A | N/A | N/A | 2.24 | N/A | N/A | N/A | N/A | N/A |
| ErbB4 Signaling | N/A | N/A | -0.45 | -1.13 | N/A | N/A | N/A | N/A | N/A | N/A | N/A | N/A | 0.45 | N/A | 0.00 |
| Cell Cycle: G2/M DNA Damage Checkpoint Regulation | N/A | N/A | N/A | N/A | N/A | N/A | N/A | 0.00 | N/A | 1.00 | N/A | N/A | N/A | 1.00 | N/A |
| Role of p14/p19ARF in Tumor Suppression | N/A | N/A | N/A | N/A | N/A | N/A | N/A | N/A | N/A | -2.00 | N/A | N/A | N/A | N/A | 0.00 |
| Remodeling of Epithelial Adherens Junctions | N/A | -1.00 | N/A | N/A | N/A | N/A | N/A | N/A | N/A | N/A | -1.00 | N/A | N/A | N/A | N/A |
| Triacylglycerol Degradation | N/A | N/A | N/A | -0.45 | N/A | N/A | N/A | N/A | 0.45 | N/A | N/A | -1.00 | N/A | N/A | N/A |
| Sperm Motility | N/A | N/A | N/A | N/A | N/A | -1.00 | N/A | N/A | N/A | N/A | N/A | N/A | 0.82 | N/A | N/A |
| Ovarian Cancer Signaling | N/A | N/A | -0.82 | 0.00 | N/A | N/A | N/A | N/A | 1.00 | N/A | N/A | N/A | N/A | N/A | N/A |
| IL-1 Signaling | N/A | -0.38 | N/A | 0.00 | 0.00 | N/A | N/A | 0.00 | N/A | 1.13 | N/A | N/A | N/A | N/A | N/A |
| Bupropion Degradation | N/A | N/A | N/A | -1.00 | N/A | N/A | N/A | N/A | N/A | N/A | -0.45 | N/A | N/A | N/A | N/A |
| Apelin Pancreas Signaling Pathway | N/A | N/A | N/A | 0.00 | 1.00 | N/A | N/A | N/A | 0.00 | 0.45 | N/A | N/A | N/A | N/A | 0.00 |
| VDR/RXR Activation | N/A | N/A | N/A | N/A | N/A | N/A | 0.00 | N/A | N/A | 1.34 | N/A | N/A | N/A | N/A | N/A |
| IL-22 Signaling | N/A | N/A | -0.45 | -0.45 | N/A | N/A | N/A | N/A | N/A | N/A | N/A | N/A | N/A | 0.45 | N/A |
| α-Adrenergic Signaling | N/A | N/A | N/A | N/A | N/A | N/A | N/A | N/A | N/A | N/A | N/A | N/A | 1.00 | N/A | N/A |
| Phosphatidylcholine Biosynthesis I | N/A | 1.00 | N/A | N/A | N/A | N/A | N/A | N/A | N/A | N/A | N/A | N/A | N/A | N/A | N/A |
| Fatty Acid β-oxidation I | N/A | 0.00 | N/A | N/A | N/A | N/A | N/A | N/A | N/A | N/A | N/A | N/A | N/A | N/A | 1.00 |
| Glycolysis I | N/A | 1.00 | N/A | N/A | N/A | N/A | N/A | N/A | N/A | N/A | N/A | N/A | N/A | N/A | N/A |
| Complement System | N/A | N/A | N/A | N/A | N/A | N/A | N/A | N/A | N/A | N/A | N/A | N/A | N/A | N/A | -1.00 |
| Amyloid Processing | N/A | N/A | -1.00 | N/A | N/A | N/A | N/A | N/A | N/A | N/A | N/A | N/A | N/A | N/A | N/A |
| Regulation of Cellular Mechanics by Calpain Protease | N/A | N/A | 0.00 | N/A | N/A | N/A | 1.00 | N/A | N/A | N/A | N/A | N/A | 0.00 | N/A | N/A |
| Retinol Biosynthesis | N/A | N/A | N/A | 0.45 | N/A | N/A | N/A | N/A | 0.45 | N/A | N/A | N/A | N/A | N/A | N/A |
| Calcium-induced T Lymphocyte Apoptosis | N/A | N/A | N/A | N/A | N/A | N/A | N/A | N/A | N/A | N/A | N/A | N/A | 0.82 | N/A | N/A |
| FAT10 Cancer Signaling Pathway | N/A | 0.00 | 0.00 | N/A | 0.45 | 0.00 | N/A | N/A | N/A | N/A | N/A | N/A | N/A | N/A | N/A |
| GPCR-Mediated Nutrient Sensing in Enteroendocrine Cells | N/A | N/A | N/A | -0.38 | N/A | N/A | N/A | N/A | N/A | N/A | N/A | N/A | 0.00 | N/A | N/A |
| Regulation of the Epithelial-Mesenchymal Transition Pathway | N/A | N/A | N/A | N/A | N/A | N/A | N/A | N/A | N/A | N/A | N/A | N/A | N/A | N/A | N/A |
| CDP-diacylglycerol Biosynthesis I | N/A | N/A | N/A | N/A | N/A | N/A | N/A | N/A | N/A | N/A | N/A | N/A | N/A | N/A | N/A |
| Pregnenolone Biosynthesis | N/A | N/A | N/A | N/A | N/A | N/A | N/A | N/A | N/A | N/A | N/A | N/A | N/A | N/A | N/A |
| Cyclins and Cell Cycle Regulation | N/A | N/A | N/A | N/A | N/A | N/A | N/A | N/A | N/A | N/A | N/A | N/A | N/A | N/A | N/A |
| Xenobiotic Metabolism Signaling | N/A | N/A | N/A | N/A | N/A | N/A | N/A | N/A | N/A | N/A | N/A | N/A | N/A | N/A | N/A |
| Ephrin A Signaling | N/A | N/A | N/A | N/A | N/A | N/A | N/A | N/A | N/A | N/A | N/A | N/A | N/A | N/A | N/A |
| Sertoli Cell-Sertoli Cell Junction Signaling | N/A | N/A | N/A | N/A | N/A | N/A | N/A | N/A | N/A | N/A | N/A | N/A | N/A | N/A | N/A |
| D-myo-inositol (1,4,5)-trisphosphate Degradation | N/A | N/A | N/A | N/A | N/A | N/A | N/A | N/A | N/A | N/A | N/A | 0.00 | 0.00 | 0.00 | N/A |
| Protein Ubiquitination Pathway | N/A | N/A | N/A | N/A | N/A | N/A | N/A | N/A | N/A | N/A | N/A | N/A | N/A | N/A | N/A |
| Altered T Cell and B Cell Signaling in Rheumatoid Arthritis | N/A | N/A | N/A | N/A | N/A | N/A | N/A | N/A | N/A | N/A | N/A | N/A | N/A | N/A | N/A |
| IL-15 Signaling | N/A | N/A | N/A | N/A | N/A | N/A | N/A | N/A | N/A | N/A | N/A | N/A | N/A | N/A | N/A |
| Hepatic Cholestasis | N/A | N/A | N/A | N/A | N/A | N/A | N/A | N/A | N/A | N/A | N/A | N/A | N/A | N/A | N/A |
| DNA Double-Strand Break Repair by Homologous Recombination | N/A | N/A | N/A | N/A | N/A | N/A | N/A | N/A | N/A | N/A | N/A | N/A | N/A | N/A | N/A |
| Maturity Onset Diabetes of Young (MODY) Signaling | N/A | N/A | N/A | N/A | N/A | N/A | N/A | N/A | N/A | N/A | N/A | N/A | N/A | N/A | N/A |
| Role of PKR in Interferon Induction and Antiviral Response | N/A | N/A | N/A | N/A | N/A | N/A | N/A | N/A | N/A | N/A | N/A | N/A | N/A | N/A | N/A |
| Cancer Drug Resistance By Drug Efflux | N/A | N/A | N/A | N/A | N/A | N/A | N/A | N/A | N/A | N/A | N/A | N/A | N/A | N/A | N/A |
| Systemic Lupus Erythematosus Signaling | N/A | N/A | N/A | N/A | N/A | N/A | N/A | N/A | N/A | N/A | N/A | N/A | N/A | N/A | N/A |
| MIF-mediated Glucocorticoid Regulation | N/A | N/A | N/A | N/A | N/A | N/A | N/A | N/A | N/A | N/A | N/A | N/A | N/A | N/A | N/A |
| Glycerol Degradation I | N/A | N/A | N/A | N/A | N/A | N/A | N/A | N/A | N/A | N/A | N/A | N/A | N/A | N/A | N/A |
| Factors Promoting Cardiogenesis in Vertebrates | N/A | N/A | N/A | N/A | N/A | N/A | N/A | N/A | N/A | N/A | N/A | N/A | N/A | N/A | N/A |
| Reelin Signaling in Neurons | N/A | N/A | N/A | N/A | N/A | N/A | N/A | N/A | N/A | N/A | N/A | N/A | N/A | N/A | N/A |
| α-tocopherol Degradation | N/A | N/A | N/A | N/A | N/A | N/A | N/A | N/A | N/A | N/A | N/A | N/A | N/A | N/A | N/A |
| Fatty Acid Activation | N/A | N/A | N/A | N/A | N/A | N/A | N/A | N/A | N/A | N/A | N/A | N/A | N/A | N/A | N/A |
| G Protein Signaling Mediated by Tubby | N/A | N/A | N/A | N/A | N/A | N/A | N/A | N/A | N/A | N/A | N/A | N/A | N/A | N/A | N/A |
| TR/RXR Activation | N/A | N/A | N/A | N/A | N/A | N/A | N/A | N/A | N/A | N/A | N/A | N/A | N/A | N/A | N/A |
| Ubiquinol-10 Biosynthesis (Eukaryotic) | N/A | N/A | N/A | N/A | N/A | N/A | N/A | N/A | N/A | N/A | N/A | N/A | N/A | N/A | N/A |
| FAK Signaling | N/A | N/A | N/A | N/A | N/A | N/A | N/A | N/A | N/A | N/A | N/A | N/A | N/A | N/A | N/A |
| Bladder Cancer Signaling | N/A | N/A | N/A | N/A | N/A | N/A | N/A | N/A | N/A | N/A | N/A | N/A | N/A | N/A | N/A |
| Phenylalanine Degradation I (Aerobic) | N/A | N/A | N/A | N/A | N/A | N/A | N/A | N/A | N/A | N/A | N/A | N/A | N/A | N/A | N/A |
| CTLA4 Signaling in Cytotoxic T Lymphocytes | N/A | N/A | N/A | N/A | N/A | N/A | N/A | N/A | N/A | N/A | N/A | N/A | N/A | N/A | N/A |
| Chronic Myeloid Leukemia Signaling | N/A | N/A | N/A | N/A | N/A | N/A | N/A | N/A | N/A | N/A | N/A | N/A | N/A | N/A | N/A |
| Role of JAK1 and JAK3 in γc Cytokine Signaling | N/A | N/A | N/A | N/A | N/A | N/A | N/A | N/A | N/A | N/A | N/A | N/A | N/A | N/A | N/A |
| Glutamate Receptor Signaling | N/A | N/A | N/A | N/A | N/A | N/A | N/A | N/A | N/A | N/A | N/A | N/A | N/A | N/A | N/A |
| Tight Junction Signaling | N/A | N/A | N/A | N/A | N/A | N/A | N/A | N/A | N/A | N/A | N/A | N/A | N/A | N/A | N/A |
| Natural Killer Cell Signaling | N/A | N/A | N/A | N/A | N/A | N/A | N/A | N/A | N/A | N/A | N/A | N/A | N/A | N/A | N/A |
| RAR Activation | N/A | N/A | N/A | N/A | N/A | N/A | N/A | N/A | N/A | N/A | N/A | N/A | N/A | N/A | N/A |
| Autophagy | N/A | N/A | N/A | N/A | N/A | N/A | N/A | N/A | N/A | N/A | N/A | N/A | N/A | N/A | N/A |
| Estrogen Receptor Signaling | N/A | N/A | N/A | N/A | N/A | N/A | N/A | N/A | N/A | N/A | N/A | N/A | N/A | N/A | N/A |
| Adipogenesis pathway | N/A | N/A | N/A | N/A | N/A | N/A | N/A | N/A | N/A | N/A | N/A | N/A | N/A | N/A | N/A |
| Cell Cycle Regulation by BTG Family Proteins | N/A | N/A | N/A | N/A | N/A | N/A | N/A | N/A | N/A | N/A | N/A | N/A | N/A | N/A | N/A |
| Lysine Degradation II | N/A | N/A | N/A | N/A | N/A | N/A | N/A | N/A | N/A | N/A | N/A | N/A | N/A | N/A | N/A |
| Regulation of IL-2 Expression in Activated and Anergic T Lymphocytes | N/A | N/A | N/A | N/A | N/A | N/A | N/A | N/A | N/A | N/A | N/A | N/A | N/A | N/A | N/A |
| Th1 and Th2 Activation Pathway | N/A | N/A | N/A | N/A | N/A | N/A | N/A | N/A | N/A | N/A | N/A | N/A | N/A | N/A | N/A |
| Proline Degradation | N/A | N/A | N/A | N/A | N/A | N/A | N/A | N/A | N/A | N/A | N/A | N/A | N/A | N/A | N/A |
| Tetrahydrofolate Salvage from 5,10-methenyltetrahydrofolate | N/A | N/A | N/A | N/A | N/A | N/A | N/A | N/A | N/A | N/A | N/A | N/A | N/A | N/A | N/A |
| DNA Methylation and Transcriptional Repression Signaling | N/A | N/A | N/A | N/A | N/A | N/A | N/A | N/A | N/A | N/A | N/A | N/A | N/A | N/A | N/A |
| D-myo-inositol (1,3,4)-trisphosphate Biosynthesis | N/A | N/A | N/A | N/A | N/A | N/A | N/A | N/A | N/A | N/A | N/A | 0.00 | 0.00 | 0.00 | N/A |
| IL-12 Signaling and Production in Macrophages | N/A | N/A | N/A | N/A | N/A | N/A | N/A | N/A | N/A | N/A | N/A | N/A | N/A | N/A | N/A |
| IL-17A Signaling in Fibroblasts | N/A | N/A | N/A | N/A | N/A | N/A | N/A | N/A | N/A | N/A | N/A | N/A | N/A | N/A | N/A |
| Tetrapyrrole Biosynthesis II | N/A | N/A | N/A | N/A | N/A | N/A | N/A | N/A | N/A | N/A | N/A | N/A | N/A | N/A | N/A |
| Assembly of RNA Polymerase III Complex | N/A | N/A | N/A | N/A | N/A | N/A | N/A | N/A | N/A | N/A | N/A | N/A | N/A | N/A | N/A |
| G-Protein Coupled Receptor Signaling | N/A | N/A | N/A | N/A | N/A | N/A | N/A | N/A | N/A | N/A | N/A | N/A | N/A | N/A | N/A |
| γ-linolenate Biosynthesis II (Animals) | N/A | N/A | N/A | N/A | N/A | N/A | N/A | N/A | N/A | N/A | N/A | N/A | N/A | N/A | N/A |
| Thyroid Cancer Signaling | N/A | N/A | N/A | N/A | N/A | N/A | N/A | N/A | N/A | N/A | N/A | N/A | N/A | N/A | N/A |
| T Cell Receptor Signaling | N/A | N/A | N/A | N/A | N/A | N/A | N/A | N/A | N/A | N/A | N/A | N/A | N/A | N/A | N/A |
| Axonal Guidance Signaling | N/A | N/A | N/A | N/A | N/A | N/A | N/A | N/A | N/A | N/A | N/A | N/A | N/A | N/A | N/A |
| Ketogenesis | N/A | N/A | N/A | N/A | N/A | N/A | N/A | N/A | N/A | N/A | N/A | N/A | N/A | N/A | N/A |
| Oxidative Ethanol Degradation III | N/A | N/A | N/A | N/A | N/A | N/A | N/A | N/A | N/A | N/A | N/A | N/A | N/A | N/A | N/A |
| MSP-RON Signaling Pathway | N/A | N/A | N/A | N/A | N/A | N/A | N/A | N/A | N/A | N/A | N/A | N/A | N/A | N/A | N/A |
| Lysine Degradation V | N/A | N/A | N/A | N/A | N/A | N/A | N/A | N/A | N/A | N/A | N/A | N/A | N/A | N/A | N/A |
| Apelin Cardiac Fibroblast Signaling Pathway | N/A | N/A | N/A | N/A | N/A | N/A | N/A | N/A | N/A | N/A | N/A | N/A | N/A | N/A | N/A |
| IL-17A Signaling in Gastric Cells | N/A | N/A | N/A | N/A | N/A | N/A | N/A | N/A | N/A | N/A | N/A | N/A | N/A | N/A | N/A |
| Nur77 Signaling in T Lymphocytes | N/A | N/A | N/A | N/A | N/A | N/A | N/A | N/A | N/A | N/A | N/A | N/A | N/A | N/A | N/A |
| Cleavage and Polyadenylation of Pre-mRNA | N/A | N/A | N/A | N/A | N/A | N/A | N/A | N/A | N/A | N/A | N/A | N/A | N/A | N/A | N/A |
| Lipid Antigen Presentation by CD1 | N/A | N/A | N/A | N/A | N/A | N/A | N/A | N/A | N/A | N/A | N/A | N/A | N/A | N/A | N/A |
| Formaldehyde Oxidation II (Glutathione-dependent) | N/A | N/A | N/A | N/A | N/A | N/A | N/A | N/A | N/A | N/A | N/A | N/A | N/A | N/A | N/A |
| Semaphorin Signaling in Neurons | N/A | N/A | N/A | N/A | N/A | N/A | N/A | N/A | N/A | N/A | N/A | N/A | N/A | N/A | N/A |
| Circadian Rhythm Signaling | N/A | N/A | N/A | N/A | N/A | N/A | N/A | N/A | N/A | N/A | N/A | N/A | N/A | N/A | N/A |
| Iron homeostasis signaling pathway | N/A | N/A | N/A | N/A | N/A | N/A | N/A | N/A | N/A | N/A | N/A | N/A | N/A | N/A | N/A |
| Atherosclerosis Signaling | N/A | N/A | N/A | N/A | N/A | N/A | N/A | N/A | N/A | N/A | N/A | N/A | N/A | N/A | N/A |
| 2-ketoglutarate Dehydrogenase Complex | N/A | N/A | N/A | N/A | N/A | N/A | N/A | N/A | N/A | N/A | N/A | N/A | N/A | N/A | N/A |
| Role of BRCA1 in DNA Damage Response | N/A | N/A | N/A | N/A | N/A | N/A | N/A | N/A | N/A | N/A | N/A | N/A | N/A | N/A | N/A |
| Ceramide Degradation | N/A | N/A | N/A | N/A | N/A | N/A | N/A | N/A | N/A | N/A | N/A | N/A | N/A | N/A | N/A |
| Coagulation System | N/A | N/A | N/A | N/A | N/A | N/A | N/A | N/A | N/A | 0.00 | N/A | N/A | N/A | N/A | N/A |
| BAG2 Signaling Pathway | N/A | N/A | N/A | N/A | N/A | N/A | N/A | N/A | N/A | N/A | 0.00 | N/A | N/A | N/A | N/A |
| Hereditary Breast Cancer Signaling | N/A | N/A | N/A | N/A | N/A | N/A | N/A | N/A | N/A | N/A | N/A | N/A | N/A | N/A | N/A |
| Assembly of RNA Polymerase II Complex | N/A | N/A | N/A | N/A | N/A | N/A | N/A | N/A | N/A | N/A | N/A | N/A | N/A | N/A | N/A |
| Myc Mediated Apoptosis Signaling | N/A | N/A | N/A | N/A | N/A | N/A | N/A | N/A | N/A | N/A | N/A | N/A | N/A | N/A | N/A |
| IL-17 Signaling | N/A | N/A | N/A | N/A | N/A | N/A | N/A | N/A | N/A | N/A | N/A | N/A | N/A | N/A | N/A |
| Glutathione Redox Reactions I | N/A | N/A | N/A | N/A | N/A | N/A | N/A | N/A | N/A | N/A | N/A | N/A | N/A | N/A | N/A |
| Telomere Extension by Telomerase | N/A | N/A | N/A | N/A | N/A | N/A | N/A | N/A | N/A | N/A | N/A | N/A | N/A | N/A | N/A |
| HER-2 Signaling in Breast Cancer | N/A | N/A | N/A | N/A | N/A | N/A | N/A | N/A | N/A | N/A | N/A | N/A | N/A | N/A | N/A |
| Valine Degradation I | N/A | N/A | N/A | N/A | N/A | N/A | N/A | N/A | N/A | N/A | N/A | N/A | N/A | N/A | N/A |
| Human Embryonic Stem Cell Pluripotency | N/A | N/A | N/A | N/A | N/A | N/A | N/A | N/A | N/A | N/A | N/A | N/A | N/A | N/A | N/A |
| Gap Junction Signaling | N/A | N/A | N/A | N/A | N/A | N/A | N/A | N/A | N/A | N/A | N/A | N/A | N/A | N/A | N/A |
| FXR/RXR Activation | N/A | N/A | N/A | N/A | N/A | N/A | N/A | N/A | N/A | N/A | N/A | N/A | N/A | N/A | N/A |
| Caveolar-mediated Endocytosis Signaling | N/A | N/A | N/A | N/A | N/A | N/A | N/A | N/A | N/A | N/A | N/A | N/A | N/A | N/A | N/A |
| Ethanol Degradation IV | N/A | N/A | N/A | N/A | N/A | N/A | N/A | N/A | N/A | N/A | N/A | N/A | N/A | N/A | N/A |
| BER pathway | N/A | N/A | N/A | N/A | N/A | N/A | N/A | N/A | N/A | N/A | N/A | N/A | N/A | N/A | N/A |
| Dopamine Receptor Signaling | N/A | N/A | N/A | N/A | N/A | N/A | N/A | N/A | N/A | N/A | N/A | N/A | N/A | N/A | N/A |
| IL-4 Signaling | N/A | N/A | N/A | N/A | N/A | N/A | N/A | N/A | N/A | N/A | N/A | N/A | N/A | N/A | N/A |
| Transcriptional Regulatory Network in Embryonic Stem Cells | N/A | N/A | N/A | N/A | N/A | N/A | N/A | N/A | N/A | N/A | N/A | N/A | N/A | N/A | N/A |
| Mitochondrial L-carnitine Shuttle Pathway | N/A | N/A | N/A | N/A | N/A | N/A | N/A | N/A | N/A | N/A | N/A | N/A | N/A | N/A | N/A |
| Glutamine Degradation I | N/A | N/A | N/A | N/A | N/A | N/A | N/A | N/A | N/A | N/A | N/A | N/A | N/A | N/A | N/A |
| Role of Oct4 in Mammalian Embryonic Stem Cell Pluripotency | N/A | N/A | N/A | N/A | N/A | N/A | N/A | N/A | N/A | N/A | N/A | N/A | N/A | N/A | N/A |
| IL-10 Signaling | N/A | N/A | N/A | N/A | N/A | N/A | N/A | N/A | N/A | N/A | N/A | N/A | N/A | N/A | N/A |
| Germ Cell-Sertoli Cell Junction Signaling | N/A | N/A | N/A | N/A | N/A | N/A | N/A | N/A | N/A | N/A | N/A | N/A | N/A | N/A | N/A |
| Prostate Cancer Signaling | N/A | N/A | N/A | N/A | N/A | N/A | N/A | N/A | N/A | N/A | N/A | N/A | N/A | N/A | N/A |
| Phosphatidylglycerol Biosynthesis II (Non-plastidic) | N/A | N/A | N/A | N/A | N/A | N/A | N/A | N/A | N/A | N/A | N/A | N/A | N/A | N/A | N/A |
| Epithelial Adherens Junction Signaling | N/A | N/A | N/A | N/A | N/A | N/A | N/A | N/A | N/A | N/A | N/A | N/A | N/A | N/A | N/A |
| Phagosome Maturation | N/A | N/A | N/A | N/A | N/A | N/A | N/A | N/A | N/A | N/A | N/A | N/A | N/A | N/A | N/A |
| Clathrin-mediated Endocytosis Signaling | N/A | N/A | N/A | N/A | N/A | N/A | N/A | N/A | N/A | N/A | N/A | N/A | N/A | N/A | N/A |
| Superpathway of D-myo-inositol (1,4,5)-trisphosphate Metabolism | N/A | N/A | N/A | N/A | N/A | N/A | N/A | N/A | N/A | N/A | N/A | 0.00 | 0.00 | 0.00 | N/A |
| Histidine Degradation VI | N/A | N/A | N/A | N/A | N/A | N/A | N/A | N/A | N/A | N/A | N/A | N/A | N/A | N/A | N/A |
| Unfolded protein response | N/A | N/A | N/A | N/A | N/A | N/A | N/A | N/A | N/A | N/A | N/A | N/A | N/A | N/A | N/A |
| Role of JAK1, JAK2 and TYK2 in Interferon Signaling | N/A | N/A | N/A | N/A | N/A | N/A | N/A | N/A | N/A | N/A | N/A | N/A | N/A | N/A | N/A |
| Antiproliferative Role of TOB in T Cell Signaling | N/A | N/A | N/A | N/A | N/A | N/A | N/A | N/A | N/A | N/A | N/A | N/A | N/A | N/A | N/A |
| PXR/RXR Activation | N/A | N/A | N/A | N/A | N/A | N/A | N/A | N/A | N/A | N/A | N/A | N/A | N/A | N/A | N/A |
| Cellular Effects of Sildenafil (Viagra) | N/A | N/A | N/A | N/A | N/A | N/A | N/A | N/A | N/A | N/A | N/A | N/A | N/A | N/A | N/A |
| Mitochondrial Dysfunction | N/A | N/A | N/A | N/A | N/A | N/A | N/A | N/A | N/A | N/A | N/A | N/A | N/A | N/A | N/A |
| Breast Cancer Regulation by Stathmin1 | N/A | N/A | N/A | N/A | N/A | N/A | N/A | N/A | N/A | N/A | N/A | N/A | N/A | N/A | N/A |
| Role of Macrophages, Fibroblasts and Endothelial Cells in Rheumatoid Arthritis | N/A | N/A | N/A | N/A | N/A | N/A | N/A | N/A | N/A | N/A | N/A | N/A | N/A | N/A | N/A |
| Role of JAK2 in Hormone-like Cytokine Signaling | N/A | N/A | N/A | N/A | N/A | N/A | N/A | N/A | N/A | N/A | N/A | N/A | N/A | N/A | N/A |
| Role of IL-17A in Arthritis | N/A | N/A | N/A | N/A | N/A | N/A | N/A | N/A | N/A | N/A | N/A | N/A | N/A | N/A | N/A |
| Calcium Transport I | N/A | N/A | N/A | N/A | N/A | N/A | N/A | N/A | N/A | N/A | N/A | N/A | N/A | N/A | N/A |
| Role of Tissue Factor in Cancer | N/A | N/A | N/A | N/A | N/A | N/A | N/A | N/A | N/A | N/A | N/A | N/A | N/A | N/A | N/A |
| Erythropoietin Signaling | N/A | N/A | N/A | N/A | N/A | N/A | N/A | N/A | N/A | N/A | N/A | N/A | N/A | N/A | N/A |
| Docosahexaenoic Acid (DHA) Signaling | N/A | N/A | N/A | N/A | N/A | N/A | N/A | N/A | N/A | N/A | N/A | N/A | N/A | N/A | N/A |
| Choline Biosynthesis III | N/A | N/A | N/A | N/A | N/A | N/A | N/A | N/A | N/A | 0.00 | N/A | N/A | N/A | N/A | N/A |
| Phagosome Formation | N/A | N/A | N/A | N/A | N/A | N/A | N/A | N/A | N/A | N/A | N/A | N/A | N/A | N/A | N/A |
| Role of JAK family kinases in IL-6-type Cytokine Signaling | N/A | N/A | N/A | N/A | N/A | N/A | N/A | N/A | N/A | N/A | N/A | N/A | N/A | N/A | N/A |
| HIF1α Signaling | N/A | N/A | N/A | N/A | N/A | N/A | N/A | N/A | N/A | N/A | N/A | N/A | N/A | N/A | N/A |
| Role of Osteoblasts, Osteoclasts and Chondrocytes in Rheumatoid Arthritis | N/A | N/A | N/A | N/A | N/A | N/A | N/A | N/A | N/A | N/A | N/A | N/A | N/A | N/A | N/A |
| 1D-myo-inositol Hexakisphosphate Biosynthesis II (Mammalian) | N/A | N/A | N/A | N/A | N/A | N/A | N/A | N/A | N/A | N/A | N/A | 0.00 | 0.00 | 0.00 | N/A |
| GADD45 Signaling | N/A | N/A | N/A | N/A | N/A | N/A | N/A | N/A | N/A | N/A | N/A | N/A | N/A | N/A | N/A |
| Hypoxia Signaling in the Cardiovascular System | N/A | N/A | N/A | N/A | N/A | N/A | N/A | N/A | N/A | N/A | N/A | N/A | N/A | N/A | N/A |
| Molecular Mechanisms of Cancer | N/A | N/A | N/A | N/A | N/A | N/A | N/A | N/A | N/A | N/A | N/A | N/A | N/A | N/A | N/A |
| IL-15 Production | N/A | N/A | N/A | N/A | N/A | N/A | N/A | N/A | N/A | N/A | N/A | N/A | N/A | N/A | N/A |
| Virus Entry via Endocytic Pathways | N/A | N/A | N/A | N/A | N/A | N/A | N/A | N/A | N/A | N/A | N/A | N/A | N/A | N/A | N/A |
| Graft-versus-Host Disease Signaling | N/A | N/A | N/A | N/A | N/A | N/A | N/A | N/A | N/A | N/A | N/A | N/A | N/A | N/A | N/A |
| Hepatic Fibrosis / Hepatic Stellate Cell Activation | N/A | N/A | N/A | N/A | N/A | N/A | N/A | N/A | N/A | N/A | N/A | N/A | N/A | N/A | N/A |
| Glucocorticoid Receptor Signaling | N/A | N/A | N/A | N/A | N/A | N/A | N/A | N/A | N/A | N/A | N/A | N/A | N/A | N/A | N/A |
| Biotin-carboxyl Carrier Protein Assembly | N/A | N/A | N/A | N/A | N/A | N/A | N/A | N/A | N/A | N/A | N/A | N/A | N/A | N/A | N/A |
| Acetate Conversion to Acetyl-CoA | N/A | N/A | N/A | N/A | N/A | N/A | N/A | N/A | N/A | N/A | N/A | N/A | N/A | N/A | N/A |

**Table 19.** A complete list of predicted z-scores for significantly altered pathways in all HZE irradiated mice across all time points. NA indicates that the directionality for the significant pathway could not be predicted based on the available literature and observed data (by IPA).

| **Sample Name** | **Mouse Strain** | **Treatment** | **Time Point (month)** | **Number of Reads Before Trimming** | **Number of Reads After Trimming** |
| --- | --- | --- | --- | --- | --- |
| B1 | C3H/HeNCrl | Control | 1 | 27,803,741 | 27,593,600 |
| B2 | C3H/HeNCrl | Control | 1 | 38,763,374 | 38,434,949 |
| B3 | C3H/HeNCrl | Control | 1 | 28,647,253 | 28,446,327 |
| B6 | C3H/HeNCrl | Control | 2 | 33,847,556 | 33,597,495 |
| B7 | C3H/HeNCrl | Control | 2 | 29,461,708 | 29,284,111 |
| B8 | C3H/HeNCrl | Control | 2 | 26,203,039 | 26,043,238 |
| B11 | C3H/HeNCrl | Control | 4 | 25,414,881 | 25,244,281 |
| B12 | C3H/HeNCrl | Control | 4 | 27,215,741 | 27,074,514 |
| B13 | C3H/HeNCrl | Control | 4 | 16,715,026 | 16,620,010 |
| B16 | C3H/HeNCrl | Control | 9 | 28,872,035 | 28,674,962 |
| B17 | C3H/HeNCrl | Control | 9 | 18,012,784 | 17,922,314 |
| B18 | C3H/HeNCrl | Control | 9 | 25,465,333 | 25,310,404 |
| B21 | C3H/HeNCrl | Control | 12 | 21,304,222 | 21,204,760 |
| B22 | C3H/HeNCrl | Control | 12 | 21,714,727 | 21,593,313 |
| B25 | C3H/HeNCrl | Control | 12 | 18,375,391 | 18,252,464 |
| E1 | C3H/HeNCrl | ^56^Fe | 1 | 34,592,008 | 34,438,044 |
| E2 | C3H/HeNCrl | ^56^Fe | 1 | 34,515,771 | 34,332,528 |
| E3 | C3H/HeNCrl | ^56^Fe | 1 | 29,559,903 | 29,429,351 |
| E6 | C3H/HeNCrl | ^56^Fe | 2 | 35,947,917 | 35,794,507 |
| E7 | C3H/HeNCrl | ^56^Fe | 2 | 31,131,612 | 30,924,067 |
| E8 | C3H/HeNCrl | ^56^Fe | 2 | 30,078,715 | 29,907,978 |
| E11 | C3H/HeNCrl | ^56^Fe | 4 | 27,971,374 | 27,880,383 |
| E12 | C3H/HeNCrl | ^56^Fe | 4 | 28,131,345 | 28,050,475 |
| E13 | C3H/HeNCrl | ^56^Fe | 4 | 27,783,844 | 27,693,338 |
| E17 | C3H/HeNCrl | ^56^Fe | 9 | 29,265,273 | 29,165,791 |
| E18 | C3H/HeNCrl | ^56^Fe | 9 | 28,164,125 | 28,072,094 |
| E19 | C3H/HeNCrl | ^56^Fe | 9 | 24,831,462 | 24,609,209 |
| E21 | C3H/HeNCrl | ^56^Fe | 12 | 31,486,613 | 31,311,847 |
| E22 | C3H/HeNCrl | ^56^Fe | 12 | 20,517,771 | 20,428,583 |
| E23 | C3H/HeNCrl | ^56^Fe | 12 | 24,249,471 | 24,096,544 |
| F3 | C3H/HeNCrl | ^16^O | 1 | 34,033,641 | 33,940,904 |
| F4 | C3H/HeNCrl | ^16^O | 1 | 37,051,878 | 36,906,215 |
| F5 | C3H/HeNCrl | ^16^O | 1 | 39,026,140 | 38,877,220 |
| F6 | C3H/HeNCrl | ^16^O | 2 | 29,096,350 | 28,991,895 |
| F7 | C3H/HeNCrl | ^16^O | 2 | 32,159,425 | 32,069,211 |
| F8 | C3H/HeNCrl | ^16^O | 2 | 30,669,033 | 30,547,830 |
| F11 | C3H/HeNCrl | ^16^O | 4 | 57,549,867 | 57,397,802 |
| F12 | C3H/HeNCrl | ^16^O | 4 | 40,188,169 | 40,073,955 |
| F13 | C3H/HeNCrl | ^16^O | 4 | 24,642,593 | 24,562,846 |
| F18 | C3H/HeNCrl | ^16^O | 9 | 23,183,122 | 23,115,690 |
| F19 | C3H/HeNCrl | ^16^O | 9 | 22,468,806 | 22,404,440 |
| F20 | C3H/HeNCrl | ^16^O | 9 | 24,030,796 | 23,952,028 |
| F23 | C3H/HeNCrl | ^16^O | 12 | 26,614,330 | 26,470,972 |
| F24 | C3H/HeNCrl | ^16^O | 12 | 28,651,764 | 28,448,632 |
| F25 | C3H/HeNCrl | ^16^O | 12 | 22,993,406 | 22,857,177 |
| A1 | C3H/HeNCrl | ^28^Si | 1 | 29,448,912 | 29,337,599 |
| A2 | C3H/HeNCrl | ^28^Si | 1 | 35,982,178 | 35,803,291 |
| A3 | C3H/HeNCrl | ^28^Si | 1 | 34,578,327 | 34,357,243 |
| A6 | C3H/HeNCrl | ^28^Si | 2 | 23,769,566 | 23,636,132 |
| A7 | C3H/HeNCrl | ^28^Si | 2 | 39,750,054 | 39,502,602 |
| A8 | C3H/HeNCrl | ^28^Si | 2 | 38,280,772 | 37,980,706 |
| A11 | C3H/HeNCrl | ^28^Si | 4 | 35,824,821 | 35,612,832 |
| A12 | C3H/HeNCrl | ^28^Si | 4 | 28,437,483 | 28,194,876 |
| A13 | C3H/HeNCrl | ^28^Si | 4 | 32,749,059 | 32,566,960 |
| A16 | C3H/HeNCrl | ^28^Si | 9 | 26,257,484 | 26,082,882 |
| A17 | C3H/HeNCrl | ^28^Si | 9 | 18,513,436 | 18,429,411 |
| A20 | C3H/HeNCrl | ^28^Si | 9 | 34,911,295 | 34,691,357 |
| A21 | C3H/HeNCrl | ^28^Si | 12 | 33,510,740 | 33,239,692 |
| A23 | C3H/HeNCrl | ^28^Si | 12 | 27,988,794 | 27,809,886 |
| A25 | C3H/HeNCrl | ^28^Si | 12 | 32,278,179 | 32,036,538 |

**Table 20.** Sample List and Total Reads.

| **Ion** | **1 month** | **2 months** | **4 months** | **9 months** | **12 months** | **Total** |
| --- | --- | --- | --- | --- | --- | --- |
| **^56^Fe** | 465 | 454 | 469 | 466 | 385 | 2239 |
| **^16^O** | 468 | 417 | 400 | 386 | 555 | 2226 |
| **^28^Si** | 584 | 480 | 609 | 446 | 419 | 2538 |

**Table 21.** Number of unannotated transcripts analyzed by DAVID (<https://david.ncifcrf.gov/>) provided in KEGG database.

| **Ion** | **1 month** | **2 months** | **4 months** | **9 months** | **12 months** | **Total** |
| --- | --- | --- | --- | --- | --- | --- |
| **^56^Fe** | 650 | 601 | 624 | 651 | 513 | 3039 |
| **^16^O** | 646 | 567 | 534 | 542 | 727 | 3016 |
| **^28^Si** | 788 | 644 | 831 | 621 | 568 | 3452 |

**Table 22.** Number of unannotated transcripts analyzed by DAVID (<https://david.ncifcrf.gov/>) provided in BIOCARTA database.

| **Database** | **Pathway** | **Count** | **%** | ***p*-value** | **Transcripts** |
| --- | --- | --- | --- | --- | --- |
| KEGG | mmu01100:Metabolic pathways | 64 | 11.5732369 | 1.40E-05 | ENSMUST00000109194, ENSMUST00000078912, ENSMUST00000035488, ENSMUST00000155749, ENSMUST00000082421, ENSMUST00000177087, ENSMUST00000092498, ENSMUST00000135441, ENSMUST00000082396, ENSMUST00000058785, ENSMUST00000200953, ENSMUST00000185612, ENSMUST00000199857, ENSMUST00000109457, ENSMUST00000022176, ENSMUST00000044721, ENSMUST00000206170, ENSMUST00000200541, ENSMUST00000184932, ENSMUST00000066540, ENSMUST00000193235, ENSMUST00000081848, ENSMUST00000165234, ENSMUST00000006692, ENSMUST00000026841, ENSMUST00000163476, ENSMUST00000115137, ENSMUST00000125476, ENSMUST00000163201, ENSMUST00000179869, ENSMUST00000159096, ENSMUST00000082407, ENSMUST00000082408, ENSMUST00000200259, ENSMUST00000206589, ENSMUST00000106146, ENSMUST00000159784, ENSMUST00000055655, ENSMUST00000137222, ENSMUST00000190637, ENSMUST00000072357, ENSMUST00000143108, ENSMUST00000152945, ENSMUST00000166036, ENSMUST00000047816, ENSMUST00000106945, ENSMUST00000113290, ENSMUST00000054963, ENSMUST00000170464, ENSMUST00000156713, ENSMUST00000168536, ENSMUST00000198239, ENSMUST00000092802, ENSMUST00000082418, ENSMUST00000082419, ENSMUST00000114171, ENSMUST00000082414, ENSMUST00000105390, ENSMUST00000082411, ENSMUST00000031082, ENSMUST00000025946, ENSMUST00000001720, ENSMUST00000074082, ENSMUST00000106188, ENSMUST00000170287, ENSMUST00000005477, ENSMUST00000113978, ENSMUST00000111067, ENSMUST00000084013, ENSMUST00000168546 |
| KEGG | mmu00564:Glycerophospholipid metabolism | 10 | 1.80831826 | 0.00185381 | ENSMUST00000206170, ENSMUST00000106188, ENSMUST00000148833, ENSMUST00000112618, ENSMUST00000047816, ENSMUST00000105390, ENSMUST00000111067, ENSMUST00000109457, ENSMUST00000115137, ENSMUST00000143108 |
| KEGG | mmu00190:Oxidative phosphorylation | 12 | 2.16998192 | 0.00281441 | ENSMUST00000082407, ENSMUST00000082408, ENSMUST00000044721, ENSMUST00000082418, ENSMUST00000114171, ENSMUST00000082419, ENSMUST00000082421, ENSMUST00000082414, ENSMUST00000084013, ENSMUST00000193235, ENSMUST00000082411, ENSMUST00000082396 |
| BIOCARTA | m_nuclearRsPathway:Nuclear Receptors in Lipid Metabolism and Toxicity | 6 | 1.08499096 | 0.003723981 | ENSMUST00000105296, ENSMUST00000042706, ENSMUST00000199857, ENSMUST00000136234, ENSMUST00000025946, ENSMUST00000102709 |
| KEGG | mmu01130:Biosynthesis of antibiotics | 15 | 2.7124774 | 0.004642973 | ENSMUST00000078912, ENSMUST00000200541, ENSMUST00000155749, ENSMUST00000066540, ENSMUST00000190637, ENSMUST00000072357, ENSMUST00000081848, ENSMUST00000001720, ENSMUST00000006692, ENSMUST00000026841, ENSMUST00000152945, ENSMUST00000170287, ENSMUST00000106945, ENSMUST00000054963, ENSMUST00000156713, ENSMUST00000022176, ENSMUST00000198239, ENSMUST00000163201, ENSMUST00000179869, ENSMUST00000159096 |
| KEGG | mmu04976:Bile secretion | 7 | 1.26582278 | 0.018679976 | ENSMUST00000166036, ENSMUST00000170287, ENSMUST00000105296, ENSMUST00000042706, ENSMUST00000005719, ENSMUST00000206106, ENSMUST00000022176, ENSMUST00000163201, ENSMUST00000102709 |
| BIOCARTA | m_fxrPathway:FXR and LXR Regulation of Cholesterol Metabolism | 3 | 0.54249548 | 0.021449748 | ENSMUST00000105296, ENSMUST00000042706, ENSMUST00000136234 |
| KEGG | mmu00900:Terpenoid backbone biosynthesis | 4 | 0.72332731 | 0.029736325 | ENSMUST00000170287, ENSMUST00000022176, ENSMUST00000081848, ENSMUST00000198239, ENSMUST00000006692, ENSMUST00000163201, ENSMUST00000179869 |
| KEGG | mmu05012:Parkinson's disease | 10 | 1.80831826 | 0.033562219 | ENSMUST00000082407, ENSMUST00000082408, ENSMUST00000082418, ENSMUST00000082419, ENSMUST00000082421, ENSMUST00000082414, ENSMUST00000084013, ENSMUST00000193235, ENSMUST00000082411, ENSMUST00000082396 |
| KEGG | mmu03040:Spliceosome | 9 | 1.62748644 | 0.044662195 | ENSMUST00000094693, ENSMUST00000114730, ENSMUST00000172549, ENSMUST00000102510, ENSMUST00000204189, ENSMUST00000136980, ENSMUST00000021306, ENSMUST00000145482, ENSMUST00000175847, ENSMUST00000043269, ENSMUST00000131283, ENSMUST00000174366 |

**Table 23.** Significant pathways for differentially expressed transcripts in ^56^Fe vs. non-irradiated control at 1 month analyzed by DAVID (<https://david.ncifcrf.gov/>).

| **Database** | **Pathway** | **Count** | **%** | ***p*-value** | **Transcripts** |
| --- | --- | --- | --- | --- | --- |
| KEGG | mmu01100:Metabolic pathways | 54 | 10.4854369 | 4.77E-04 | ENSMUST00000205318, ENSMUST00000168419, ENSMUST00000114783, ENSMUST00000185466, ENSMUST00000022858, ENSMUST00000033634, ENSMUST00000097912, ENSMUST00000134104, ENSMUST00000184371, ENSMUST00000137540, ENSMUST00000133320, ENSMUST00000118390, ENSMUST00000169273, ENSMUST00000099149, ENSMUST00000112907, ENSMUST00000165111, ENSMUST00000130229, ENSMUST00000165234, ENSMUST00000105470, ENSMUST00000205983, ENSMUST00000163602, ENSMUST00000026039, ENSMUST00000177400, ENSMUST00000147165, ENSMUST00000199940, ENSMUST00000115137, ENSMUST00000149290, ENSMUST00000135379, ENSMUST00000200259, ENSMUST00000159784, ENSMUST00000030830, ENSMUST00000190637, ENSMUST00000075630, ENSMUST00000025546, ENSMUST00000143741, ENSMUST00000143108, ENSMUST00000072841, ENSMUST00000166036, ENSMUST00000127439, ENSMUST00000159644, ENSMUST00000106945, ENSMUST00000170464, ENSMUST00000072055, ENSMUST00000172533, ENSMUST00000026887, ENSMUST00000026462, ENSMUST00000044038, ENSMUST00000143827, ENSMUST00000135949, ENSMUST00000084771, ENSMUST00000130698, ENSMUST00000180358, ENSMUST00000134053, ENSMUST00000089449, ENSMUST00000151505, ENSMUST00000025391, ENSMUST00000131186, ENSMUST00000189437 |
| KEGG | mmu04622:RIG-I-like receptor signaling pathway | 8 | 1.55339806 | 0.00222918 | ENSMUST00000000284, ENSMUST00000026217, ENSMUST00000108184, ENSMUST00000180230, ENSMUST00000114130, ENSMUST00000133580, ENSMUST00000168499, ENSMUST00000178543, ENSMUST00000164101, ENSMUST00000125314 |
| KEGG | mmu05231:Choline metabolism in cancer | 9 | 1.74757282 | 0.00570754 | ENSMUST00000177654, ENSMUST00000163602, ENSMUST00000149854, ENSMUST00000028822, ENSMUST00000060955, ENSMUST00000115137, ENSMUST00000110136, ENSMUST00000072055, ENSMUST00000178543, ENSMUST00000173399, ENSMUST00000113870, ENSMUST00000143108 |
| KEGG | mmu04620:Toll-like receptor signaling pathway | 9 | 1.74757282 | 0.00570754 | ENSMUST00000149871, ENSMUST00000177654, ENSMUST00000026217, ENSMUST00000108184, ENSMUST00000180230, ENSMUST00000114130, ENSMUST00000151890, ENSMUST00000178543, ENSMUST00000164101, ENSMUST00000034056, ENSMUST00000125314 |
| KEGG | mmu04146:Peroxisome | 8 | 1.55339806 | 0.00680875 | ENSMUST00000135379, ENSMUST00000166036, ENSMUST00000033634, ENSMUST00000102855, ENSMUST00000066514, ENSMUST00000088561, ENSMUST00000112907, ENSMUST00000130229, ENSMUST00000111168 |
| KEGG | mmu05222:Small cell lung cancer | 8 | 1.55339806 | 0.00726275 | ENSMUST00000177654, ENSMUST00000026217, ENSMUST00000111740, ENSMUST00000187938, ENSMUST00000180230, ENSMUST00000114130, ENSMUST00000204807, ENSMUST00000188674, ENSMUST00000164101, ENSMUST00000145065, ENSMUST00000125314 |
| KEGG | mmu05166:HTLV-I infection | 16 | 3.10679612 | 0.0074346 | ENSMUST00000100001, ENSMUST00000177654, ENSMUST00000029865, ENSMUST00000180230, ENSMUST00000114130, ENSMUST00000164101, ENSMUST00000103118, ENSMUST00000125314, ENSMUST00000154356, ENSMUST00000058801, ENSMUST00000090802, ENSMUST00000132925, ENSMUST00000105343, ENSMUST00000094120, ENSMUST00000026217, ENSMUST00000038980, ENSMUST00000005719, ENSMUST00000196640, ENSMUST00000167977, ENSMUST00000145065 |
| KEGG | mmu05215:Prostate cancer | 8 | 1.55339806 | 0.00930561 | ENSMUST00000177654, ENSMUST00000026217, ENSMUST00000166469, ENSMUST00000122054, ENSMUST00000180230, ENSMUST00000114130, ENSMUST00000204807, ENSMUST00000164101, ENSMUST00000124096, ENSMUST00000125314, ENSMUST00000154356 |
| KEGG | mmu04068:FoxO signaling pathway | 10 | 1.94174757 | 0.00999039 | ENSMUST00000177654, ENSMUST00000026217, ENSMUST00000100036, ENSMUST00000105371, ENSMUST00000204807, ENSMUST00000204956, ENSMUST00000178543, ENSMUST00000147730, ENSMUST00000125314, ENSMUST00000111168 |
| KEGG | mmu05169:Epstein-Barr virus infection | 10 | 1.94174757 | 0.01094967 | ENSMUST00000177654, ENSMUST00000108184, ENSMUST00000180230, ENSMUST00000114130, ENSMUST00000111393, ENSMUST00000164101, ENSMUST00000125314, ENSMUST00000058801, ENSMUST00000149871, ENSMUST00000026217, ENSMUST00000055256, ENSMUST00000204807, ENSMUST00000178543 |
| KEGG | mmu04520:Adherens junction | 7 | 1.3592233 | 0.01264176 | ENSMUST00000162602, ENSMUST00000108184, ENSMUST00000111493, ENSMUST00000161355, ENSMUST00000103021, ENSMUST00000162456, ENSMUST00000173399, ENSMUST00000154356, ENSMUST00000115442 |
| KEGG | mmu04151:PI3K-Akt signaling pathway | 18 | 3.49514563 | 0.01346728 | ENSMUST00000177654, ENSMUST00000100036, ENSMUST00000111740, ENSMUST00000122054, ENSMUST00000105371, ENSMUST00000166469, ENSMUST00000164207, ENSMUST00000180230, ENSMUST00000114130, ENSMUST00000164101, ENSMUST00000124096, ENSMUST00000132525, ENSMUST00000125314, ENSMUST00000113870, ENSMUST00000090802, ENSMUST00000132945, ENSMUST00000153628, ENSMUST00000026217, ENSMUST00000187938, ENSMUST00000204807, ENSMUST00000188674, ENSMUST00000115442 |
| KEGG | mmu04010:MAPK signaling pathway | 14 | 2.7184466 | 0.01790802 | ENSMUST00000124556, ENSMUST00000003741, ENSMUST00000017435, ENSMUST00000122054, ENSMUST00000164207, ENSMUST00000108184, ENSMUST00000180230, ENSMUST00000114130, ENSMUST00000117509, ENSMUST00000178969, ENSMUST00000164101, ENSMUST00000124096, ENSMUST00000125314, ENSMUST00000090802, ENSMUST00000101454, ENSMUST00000026217, ENSMUST00000114299, ENSMUST00000167977, ENSMUST00000178543 |
| KEGG | mmu04621:NOD-like receptor signaling pathway | 6 | 1.16504854 | 0.01814352 | ENSMUST00000026217, ENSMUST00000166469, ENSMUST00000108184, ENSMUST00000180230, ENSMUST00000114130, ENSMUST00000178543, ENSMUST00000164101, ENSMUST00000125314 |
| KEGG | mmu04380:Osteoclast differentiation | 9 | 1.74757282 | 0.02012909 | ENSMUST00000177654, ENSMUST00000026217, ENSMUST00000049262, ENSMUST00000108184, ENSMUST00000180230, ENSMUST00000114130, ENSMUST00000167977, ENSMUST00000178543, ENSMUST00000164101, ENSMUST00000143379, ENSMUST00000125314 |
| BIOCARTA | m_nfkbpathway:NF-kB Signaling Pathway | 5 | 0.97087379 | 0.02240371 | ENSMUST00000149871, ENSMUST00000026217, ENSMUST00000108184, ENSMUST00000180230, ENSMUST00000114130, ENSMUST00000164101, ENSMUST00000125314 |
| KEGG | mmu04144:Endocytosis | 14 | 2.7184466 | 0.02383074 | ENSMUST00000162602, ENSMUST00000112605, ENSMUST00000106305, ENSMUST00000164207, ENSMUST00000122054, ENSMUST00000068054, ENSMUST00000154686, ENSMUST00000058033, ENSMUST00000085399, ENSMUST00000162456, ENSMUST00000124096, ENSMUST00000173399, ENSMUST00000058801, ENSMUST00000100215, ENSMUST00000161355, ENSMUST00000110115, ENSMUST00000172482 |
| KEGG | mmu01200:Carbon metabolism | 8 | 1.55339806 | 0.03663101 | ENSMUST00000205983, ENSMUST00000135949, ENSMUST00000159784, ENSMUST00000151505, ENSMUST00000030830, ENSMUST00000175887, ENSMUST00000072841, ENSMUST00000111168 |
| KEGG | mmu05200:Pathways in cancer | 18 | 3.49514563 | 0.03712511 | ENSMUST00000177654, ENSMUST00000111740, ENSMUST00000122054, ENSMUST00000166469, ENSMUST00000164207, ENSMUST00000180230, ENSMUST00000114130, ENSMUST00000164101, ENSMUST00000124096, ENSMUST00000132525, ENSMUST00000125314, ENSMUST00000154356, ENSMUST00000072767, ENSMUST00000026217, ENSMUST00000187938, ENSMUST00000005719, ENSMUST00000184371, ENSMUST00000204807, ENSMUST00000188674, ENSMUST00000178543, ENSMUST00000145065, ENSMUST00000115442 |
| KEGG | mmu05144:Malaria | 5 | 0.97087379 | 0.03947306 | ENSMUST00000161883, ENSMUST00000106796, ENSMUST00000183610, ENSMUST00000023934, ENSMUST00000141954, ENSMUST00000115442 |
| KEGG | mmu04662:B cell receptor signaling pathway | 6 | 1.16504854 | 0.03974436 | ENSMUST00000177654, ENSMUST00000026217, ENSMUST00000141954, ENSMUST00000180230, ENSMUST00000114130, ENSMUST00000167977, ENSMUST00000164101, ENSMUST00000125314 |
| KEGG | mmu05164:Influenza A | 10 | 1.94174757 | 0.04102996 | ENSMUST00000000284, ENSMUST00000177654, ENSMUST00000090802, ENSMUST00000098924, ENSMUST00000168499, ENSMUST00000178543, ENSMUST00000190052, ENSMUST00000122232, ENSMUST00000034056, ENSMUST00000125314 |
| KEGG | mmu05161:Hepatitis B | 9 | 1.74757282 | 0.04308117 | ENSMUST00000177654, ENSMUST00000090802, ENSMUST00000026217, ENSMUST00000180230, ENSMUST00000114130, ENSMUST00000167977, ENSMUST00000204807, ENSMUST00000178543, ENSMUST00000164101, ENSMUST00000034056, ENSMUST00000125314 |
| KEGG | mmu04920:Adipocytokine signaling pathway | 6 | 1.16504854 | 0.04403816 | ENSMUST00000033634, ENSMUST00000026217, ENSMUST00000105371, ENSMUST00000180230, ENSMUST00000112907, ENSMUST00000114130, ENSMUST00000178543, ENSMUST00000164101, ENSMUST00000125314 |
| KEGG | mmu04064:NF-kappa B signaling pathway | 7 | 1.3592233 | 0.04631091 | ENSMUST00000149871, ENSMUST00000000284, ENSMUST00000026217, ENSMUST00000108184, ENSMUST00000180230, ENSMUST00000114130, ENSMUST00000164101, ENSMUST00000145065, ENSMUST00000125314 |
| KEGG | mmu05205:Proteoglycans in cancer | 11 | 2.13592233 | 0.04715136 | ENSMUST00000161883, ENSMUST00000177654, ENSMUST00000111740, ENSMUST00000163153, ENSMUST00000154356, ENSMUST00000072767, ENSMUST00000101454, ENSMUST00000153628, ENSMUST00000114299, ENSMUST00000187938, ENSMUST00000188674, ENSMUST00000182795, ENSMUST00000115442 |
| KEGG | mmu01212:Fatty acid metabolism | 5 | 0.97087379 | 0.04767419 | ENSMUST00000133757, ENSMUST00000033634, ENSMUST00000151505, ENSMUST00000112907, ENSMUST00000114783, ENSMUST00000130229 |

**Table 24.** Significant pathways for differentially expressed transcripts in ^56^Fe vs. non-irradiated control at 2 months analyzed by DAVID (<https://david.ncifcrf.gov/>).

| **Database** | **Pathway** | **Count** | **%** | ***p*-value** | **Transcripts** |
| --- | --- | --- | --- | --- | --- |
| KEGG | mmu05200:Pathways in cancer | 25 | 4.72589792 | 1.68E-04 | ENSMUST00000107357, ENSMUST00000107358, ENSMUST00000074246, ENSMUST00000060274, ENSMUST00000079362, ENSMUST00000019362, ENSMUST00000113934, ENSMUST00000138518, ENSMUST00000174299, ENSMUST00000166775, ENSMUST00000135310, ENSMUST00000067150, ENSMUST00000108242, ENSMUST00000126690, ENSMUST00000091328, ENSMUST00000186940, ENSMUST00000105263, ENSMUST00000165335, ENSMUST00000114133, ENSMUST00000111740, ENSMUST00000179165, ENSMUST00000170293, ENSMUST00000044234, ENSMUST00000120375, ENSMUST00000111160, ENSMUST00000205391, ENSMUST00000171509, ENSMUST00000033099 |
| KEGG | mmu05222:Small cell lung cancer | 10 | 1.89035917 | 4.28E-04 | ENSMUST00000120375, ENSMUST00000074246, ENSMUST00000166775, ENSMUST00000114133, ENSMUST00000060274, ENSMUST00000111740, ENSMUST00000108242, ENSMUST00000111160, ENSMUST00000113934, ENSMUST00000186940, ENSMUST00000174299 |
| KEGG | mmu05221:Acute myeloid leukemia | 8 | 1.51228733 | 7.41E-04 | ENSMUST00000107357, ENSMUST00000107358, ENSMUST00000114133, ENSMUST00000135310, ENSMUST00000108242, ENSMUST00000126690, ENSMUST00000111160, ENSMUST00000091328, ENSMUST00000205391 |
| KEGG | mmu04068:FoxO signaling pathway | 12 | 2.268431 | 0.00101185 | ENSMUST00000209056, ENSMUST00000023151, ENSMUST00000019469, ENSMUST00000021903, ENSMUST00000208312, ENSMUST00000097826, ENSMUST00000108242, ENSMUST00000111160, ENSMUST00000121127, ENSMUST00000091328, ENSMUST00000138518, ENSMUST00000105263, ENSMUST00000044234 |
| KEGG | mmu04550:Signaling pathways regulating pluripotency of stem cells | 12 | 2.268431 | 0.00128836 | ENSMUST00000056006, ENSMUST00000035093, ENSMUST00000179165, ENSMUST00000079362, ENSMUST00000019362, ENSMUST00000025705, ENSMUST00000044608, ENSMUST00000165164, ENSMUST00000067150, ENSMUST00000108242, ENSMUST00000111160, ENSMUST00000091328, ENSMUST00000171509 |
| KEGG | mmu04668:TNF signaling pathway | 10 | 1.89035917 | 0.00276684 | ENSMUST00000187811, ENSMUST00000074246, ENSMUST00000060274, ENSMUST00000114133, ENSMUST00000031327, ENSMUST00000105527, ENSMUST00000112016, ENSMUST00000108242, ENSMUST00000112017, ENSMUST00000111160, ENSMUST00000091328, ENSMUST00000112007, ENSMUST00000201245 |
| KEGG | mmu04151:PI3K-Akt signaling pathway | 20 | 3.78071834 | 0.00313239 | ENSMUST00000165335, ENSMUST00000187811, ENSMUST00000019469, ENSMUST00000114133, ENSMUST00000111740, ENSMUST00000097826, ENSMUST00000170293, ENSMUST00000113934, ENSMUST00000138518, ENSMUST00000025705, ENSMUST00000120375, ENSMUST00000166775, ENSMUST00000055087, ENSMUST00000067150, ENSMUST00000108242, ENSMUST00000112016, ENSMUST00000112017, ENSMUST00000111160, ENSMUST00000091328, ENSMUST00000186940, ENSMUST00000112007, ENSMUST00000171509, ENSMUST00000033099, ENSMUST00000105263 |
| KEGG | mmu04920:Adipocytokine signaling pathway | 8 | 1.51228733 | 0.00326487 | ENSMUST00000019469, ENSMUST00000166775, ENSMUST00000114133, ENSMUST00000112903, ENSMUST00000110371, ENSMUST00000111160, ENSMUST00000113934, ENSMUST00000174299, ENSMUST00000025705 |
| KEGG | mmu05220:Chronic myeloid leukemia | 8 | 1.51228733 | 0.00326487 | ENSMUST00000107357, ENSMUST00000107358, ENSMUST00000114133, ENSMUST00000108242, ENSMUST00000111160, ENSMUST00000091328, ENSMUST00000105263, ENSMUST00000044234 |
| KEGG | mmu04062:Chemokine signaling pathway | 13 | 2.45746692 | 0.00706105 | ENSMUST00000107358, ENSMUST00000165335, ENSMUST00000179755, ENSMUST00000114133, ENSMUST00000152156, ENSMUST00000031327, ENSMUST00000025791, ENSMUST00000170293, ENSMUST00000025705, ENSMUST00000108242, ENSMUST00000111160, ENSMUST00000091328, ENSMUST00000161355, ENSMUST00000201245 |
| KEGG | mmu04380:Osteoclast differentiation | 10 | 1.89035917 | 0.0072004 | ENSMUST00000136936, ENSMUST00000187811, ENSMUST00000055087, ENSMUST00000114133, ENSMUST00000108242, ENSMUST00000111160, ENSMUST00000091328, ENSMUST00000169017, ENSMUST00000103203, ENSMUST00000044234 |
| KEGG | mmu05230:Central carbon metabolism in cancer | 7 | 1.32325142 | 0.00757407 | ENSMUST00000151358, ENSMUST00000067150, ENSMUST00000108242, ENSMUST00000111160, ENSMUST00000134104, ENSMUST00000091328, ENSMUST00000138518, ENSMUST00000171509 |
| KEGG | mmu04919:Thyroid hormone signaling pathway | 9 | 1.70132325 | 0.01221116 | ENSMUST00000168177, ENSMUST00000124072, ENSMUST00000166775, ENSMUST00000111740, ENSMUST00000108242, ENSMUST00000111160, ENSMUST00000091328, ENSMUST00000113934, ENSMUST00000174299, ENSMUST00000105263 |
| KEGG | mmu04810:Regulation of actin cytoskeleton | 13 | 2.45746692 | 0.01312862 | ENSMUST00000026436, ENSMUST00000152156, ENSMUST00000111740, ENSMUST00000079362, ENSMUST00000085255, ENSMUST00000138518, ENSMUST00000037285, ENSMUST00000120375, ENSMUST00000067150, ENSMUST00000108242, ENSMUST00000091328, ENSMUST00000186940, ENSMUST00000171509, ENSMUST00000033099 |
| KEGG | mmu05223:Non-small cell lung cancer | 6 | 1.1342155 | 0.01755914 | ENSMUST00000166775, ENSMUST00000108242, ENSMUST00000111160, ENSMUST00000091328, ENSMUST00000113934, ENSMUST00000138518, ENSMUST00000174299 |
| KEGG | mmu05161:Hepatitis B | 10 | 1.89035917 | 0.01779435 | ENSMUST00000107357, ENSMUST00000107358, ENSMUST00000187811, ENSMUST00000114133, ENSMUST00000108242, ENSMUST00000112016, ENSMUST00000111160, ENSMUST00000112017, ENSMUST00000167106, ENSMUST00000091328, ENSMUST00000112007, ENSMUST00000044234 |
| BIOCARTA | m_gleevecPathway:Inhibition of Cellular Proliferation by Gleevec | 5 | 0.94517958 | 0.0180808 | ENSMUST00000107357, ENSMUST00000107358, ENSMUST00000108242, ENSMUST00000091328, ENSMUST00000025705 |
| KEGG | mmu03320:PPAR signaling pathway | 7 | 1.32325142 | 0.02127614 | ENSMUST00000166775, ENSMUST00000112903, ENSMUST00000041331, ENSMUST00000110371, ENSMUST00000130031, ENSMUST00000029905, ENSMUST00000113934, ENSMUST00000174299 |
| KEGG | mmu00260:Glycine, serine and threonine metabolism | 5 | 0.94517958 | 0.0225571 | ENSMUST00000112524, ENSMUST00000074082, ENSMUST00000123115, ENSMUST00000123601, ENSMUST00000118917, ENSMUST00000128351, ENSMUST00000133617, ENSMUST00000124193, ENSMUST00000174093, ENSMUST00000143125, ENSMUST00000134053 |
| BIOCARTA | m_tpoPathway:TPO Signaling Pathway | 5 | 0.94517958 | 0.02357416 | ENSMUST00000107357, ENSMUST00000107358, ENSMUST00000108242, ENSMUST00000091328, ENSMUST00000025705 |
| KEGG | mmu04144:Endocytosis | 14 | 2.64650284 | 0.02581976 | ENSMUST00000179755, ENSMUST00000025791, ENSMUST00000080418, ENSMUST00000138518, ENSMUST00000037285, ENSMUST00000178202, ENSMUST00000044234, ENSMUST00000177731, ENSMUST00000176384, ENSMUST00000110416, ENSMUST00000135310, ENSMUST00000067150, ENSMUST00000126690, ENSMUST00000161355, ENSMUST00000171509, ENSMUST00000105263 |
| KEGG | mmu04012:ErbB signaling pathway | 7 | 1.32325142 | 0.03063656 | ENSMUST00000107357, ENSMUST00000107358, ENSMUST00000108242, ENSMUST00000111160, ENSMUST00000091328, ENSMUST00000119142, ENSMUST00000138518 |
| BIOCARTA | m_il3Pathway:IL 3 signaling pathway | 4 | 0.75614367 | 0.03116527 | ENSMUST00000107357, ENSMUST00000107358, ENSMUST00000091328, ENSMUST00000025705 |
| BIOCARTA | :Role of ï¿½-arrestins in the activation and targeting of MAP kinases | 4 | 0.75614367 | 0.03116527 | ENSMUST00000165335, ENSMUST00000179755, ENSMUST00000025791, ENSMUST00000091328 |
| KEGG | mmu05212:Pancreatic cancer | 6 | 1.1342155 | 0.0312662 | ENSMUST00000114133, ENSMUST00000108242, ENSMUST00000111160, ENSMUST00000091328, ENSMUST00000138518, ENSMUST00000044234 |
| BIOCARTA | m_fxrPathway:FXR and LXR Regulation of Cholesterol Metabolism | 3 | 0.56710775 | 0.03160656 | ENSMUST00000166775, ENSMUST00000105296, ENSMUST00000130031, ENSMUST00000113934 |
| KEGG | mmu05160:Hepatitis C | 9 | 1.70132325 | 0.0316352 | ENSMUST00000166775, ENSMUST00000114133, ENSMUST00000060274, ENSMUST00000108242, ENSMUST00000111160, ENSMUST00000130031, ENSMUST00000167106, ENSMUST00000091328, ENSMUST00000113934, ENSMUST00000138518 |
| KEGG | mmu05215:Prostate cancer | 7 | 1.32325142 | 0.03216244 | ENSMUST00000187811, ENSMUST00000114133, ENSMUST00000108242, ENSMUST00000111160, ENSMUST00000091328, ENSMUST00000138518, ENSMUST00000105263 |
| BIOCARTA | m_ghPathway:Growth Hormone Signaling Pathway | 5 | 0.94517958 | 0.03354609 | ENSMUST00000107357, ENSMUST00000107358, ENSMUST00000108242, ENSMUST00000091328, ENSMUST00000025705 |
| KEGG | mmu04725:Cholinergic synapse | 8 | 1.51228733 | 0.03390624 | ENSMUST00000165335, ENSMUST00000187811, ENSMUST00000199372, ENSMUST00000108242, ENSMUST00000111160, ENSMUST00000170293, ENSMUST00000091328, ENSMUST00000025705 |
| KEGG | mmu05166:HTLV-I infection | 14 | 2.64650284 | 0.03762513 | ENSMUST00000107357, ENSMUST00000107358, ENSMUST00000187811, ENSMUST00000114133, ENSMUST00000179165, ENSMUST00000079362, ENSMUST00000019362, ENSMUST00000197074, ENSMUST00000044234, ENSMUST00000038980, ENSMUST00000108242, ENSMUST00000112016, ENSMUST00000111160, ENSMUST00000112017, ENSMUST00000112007, ENSMUST00000165033 |
| KEGG | mmu04120:Ubiquitin mediated proteolysis | 9 | 1.70132325 | 0.03936105 | ENSMUST00000074246, ENSMUST00000067908, ENSMUST00000135310, ENSMUST00000080418, ENSMUST00000035973, ENSMUST00000126690, ENSMUST00000064900, ENSMUST00000197074, ENSMUST00000076383, ENSMUST00000105263 |
| KEGG | mmu00562:Inositol phosphate metabolism | 6 | 1.1342155 | 0.04115133 | ENSMUST00000118390, ENSMUST00000092802, ENSMUST00000184454, ENSMUST00000109943, ENSMUST00000108242, ENSMUST00000115790 |
| KEGG | mmu05218:Melanoma | 6 | 1.1342155 | 0.043331 | ENSMUST00000108242, ENSMUST00000111160, ENSMUST00000091328, ENSMUST00000138518, ENSMUST00000033099, ENSMUST00000105263 |
| BIOCARTA | m_tnfr2Pathway:TNFR2 Signaling Pathway | 4 | 0.75614367 | 0.04782603 | ENSMUST00000105527, ENSMUST00000114133, ENSMUST00000060274, ENSMUST00000078074 |
| KEGG | mmu04917:Prolactin signaling pathway | 6 | 1.1342155 | 0.04789543 | ENSMUST00000107357, ENSMUST00000107358, ENSMUST00000108242, ENSMUST00000111160, ENSMUST00000091328, ENSMUST00000025705 |
| KEGG | mmu05203:Viral carcinogenesis | 12 | 2.268431 | 0.04983417 | ENSMUST00000107357, ENSMUST00000107358, ENSMUST00000187811, ENSMUST00000114133, ENSMUST00000152156, ENSMUST00000060274, ENSMUST00000129949, ENSMUST00000055087, ENSMUST00000165860, ENSMUST00000112016, ENSMUST00000108242, ENSMUST00000112017, ENSMUST00000091328, ENSMUST00000112007, ENSMUST00000105263 |
| KEGG | mmu05205:Proteoglycans in cancer | 11 | 2.07939509 | 0.0499587 | ENSMUST00000169061, ENSMUST00000152156, ENSMUST00000111740, ENSMUST00000179165, ENSMUST00000108242, ENSMUST00000111160, ENSMUST00000091328, ENSMUST00000119142, ENSMUST00000138518, ENSMUST00000186940, ENSMUST00000105263 |

**Table 25.** Significant pathways for differentially expressed transcripts in ^56^Fe vs. non-irradiated control at 4 months analyzed by DAVID (<https://david.ncifcrf.gov/>).

| **Database** | **Pathway** | **Count** | **%** | ***p*-value** | **Transcripts** |
| --- | --- | --- | --- | --- | --- |
| KEGG | mmu04917:Prolactin signaling pathway | 10 | 1.73310225 | 5.69E-04 | ENSMUST00000170690, ENSMUST00000107357, ENSMUST00000038859, ENSMUST00000106497, ENSMUST00000114752, ENSMUST00000138438, ENSMUST00000102778, ENSMUST00000128450, ENSMUST00000054002, ENSMUST00000124971 |
| KEGG | mmu00830:Retinol metabolism | 11 | 1.90641248 | 6.03E-04 | ENSMUST00000058785, ENSMUST00000071646, ENSMUST00000035488, ENSMUST00000098657, ENSMUST00000025968, ENSMUST00000176364, ENSMUST00000005669, ENSMUST00000176624, ENSMUST00000026462, ENSMUST00000025966, ENSMUST00000082214 |
| KEGG | mmu04750:Inflammatory mediator regulation of TRP channels | 13 | 2.25303293 | 8.04E-04 | ENSMUST00000058785, ENSMUST00000071816, ENSMUST00000038859, ENSMUST00000035488, ENSMUST00000114752, ENSMUST00000102778, ENSMUST00000025968, ENSMUST00000064921, ENSMUST00000152065, ENSMUST00000168545, ENSMUST00000027241, ENSMUST00000176624, ENSMUST00000025966 |
| KEGG | mmu04910:Insulin signaling pathway | 13 | 2.25303293 | 0.00201255 | ENSMUST00000107482, ENSMUST00000106497, ENSMUST00000055655, ENSMUST00000019631, ENSMUST00000124971, ENSMUST00000103201, ENSMUST00000113870, ENSMUST00000170690, ENSMUST00000113869, ENSMUST00000038859, ENSMUST00000102778, ENSMUST00000054002, ENSMUST00000051186, ENSMUST00000052949 |
| KEGG | mmu05223:Non-small cell lung cancer | 8 | 1.3864818 | 0.00214584 | ENSMUST00000142558, ENSMUST00000038859, ENSMUST00000166775, ENSMUST00000106497, ENSMUST00000064921, ENSMUST00000124971, ENSMUST00000174299, ENSMUST00000049503 |
| KEGG | mmu04920:Adipocytokine signaling pathway | 9 | 1.55979203 | 0.002264 | ENSMUST00000105370, ENSMUST00000165232, ENSMUST00000166775, ENSMUST00000110371, ENSMUST00000138438, ENSMUST00000102778, ENSMUST00000054002, ENSMUST00000170051, ENSMUST00000169095, ENSMUST00000174299, ENSMUST00000051186, ENSMUST00000197890 |
| KEGG | mmu04015:Rap1 signaling pathway | 16 | 2.7729636 | 0.00426763 | ENSMUST00000119260, ENSMUST00000028525, ENSMUST00000193835, ENSMUST00000000312, ENSMUST00000093769, ENSMUST00000144270, ENSMUST00000202138, ENSMUST00000064921, ENSMUST00000124971, ENSMUST00000160766, ENSMUST00000038859, ENSMUST00000114752, ENSMUST00000152065, ENSMUST00000161355, ENSMUST00000168545, ENSMUST00000153541, ENSMUST00000108775 |
| KEGG | mmu03320:PPAR signaling pathway | 9 | 1.55979203 | 0.0043862 | ENSMUST00000174299, ENSMUST00000169095, ENSMUST00000197890, ENSMUST00000058785, ENSMUST00000165232, ENSMUST00000166775, ENSMUST00000041331, ENSMUST00000113978, ENSMUST00000110371, ENSMUST00000170051, ENSMUST00000023820, ENSMUST00000168401, ENSMUST00000015712 |
| KEGG | mmu00310:Lysine degradation | 7 | 1.21317158 | 0.00658732 | ENSMUST00000114432, ENSMUST00000114689, ENSMUST00000178486, ENSMUST00000074127, ENSMUST00000108154, ENSMUST00000093350, ENSMUST00000023741, ENSMUST00000015841 |
| KEGG | mmu04120:Ubiquitin mediated proteolysis | 12 | 2.0797227 | 0.00660087 | ENSMUST00000205303, ENSMUST00000165394, ENSMUST00000035973, ENSMUST00000172836, ENSMUST00000197074, ENSMUST00000186894, ENSMUST00000123497, ENSMUST00000164095, ENSMUST00000135310, ENSMUST00000075082, ENSMUST00000020101, ENSMUST00000182845, ENSMUST00000054002 |
| BIOCARTA | m_biopeptidesPathway:Bioactive Peptide Induced Signaling Pathway | 7 | 1.21317158 | 0.0082795 | ENSMUST00000107357, ENSMUST00000106497, ENSMUST00000114752, ENSMUST00000138438, ENSMUST00000064921, ENSMUST00000063091, ENSMUST00000124971 |
| KEGG | mmu05221:Acute myeloid leukemia | 7 | 1.21317158 | 0.00943156 | ENSMUST00000107357, ENSMUST00000038859, ENSMUST00000106497, ENSMUST00000135310, ENSMUST00000138438, ENSMUST00000144270, ENSMUST00000124971 |
| KEGG | mmu01040:Biosynthesis of unsaturated fatty acids | 5 | 0.86655113 | 0.0107606 | ENSMUST00000034904, ENSMUST00000071402, ENSMUST00000021649, ENSMUST00000041331, ENSMUST00000021653, ENSMUST00000120927, ENSMUST00000197070 |
| KEGG | mmu04070:Phosphatidylinositol signaling system | 9 | 1.55979203 | 0.01364313 | ENSMUST00000118390, ENSMUST00000038859, ENSMUST00000131405, ENSMUST00000064921, ENSMUST00000125476, ENSMUST00000089461, ENSMUST00000040448, ENSMUST00000028291, ENSMUST00000087657 |
| KEGG | mmu05231:Choline metabolism in cancer | 9 | 1.55979203 | 0.01708303 | ENSMUST00000113869, ENSMUST00000038859, ENSMUST00000106497, ENSMUST00000102778, ENSMUST00000064921, ENSMUST00000124971, ENSMUST00000128694, ENSMUST00000127712, ENSMUST00000113870, ENSMUST00000143108 |
| KEGG | mmu04660:T cell receptor signaling pathway | 9 | 1.55979203 | 0.01708303 | ENSMUST00000142558, ENSMUST00000038859, ENSMUST00000106497, ENSMUST00000080933, ENSMUST00000114752, ENSMUST00000102534, ENSMUST00000182755, ENSMUST00000063091, ENSMUST00000124971 |
| KEGG | mmu05202:Transcriptional misregulation in cancer | 12 | 2.0797227 | 0.01897059 | ENSMUST00000051446, ENSMUST00000052368, ENSMUST00000020377, ENSMUST00000114689, ENSMUST00000115677, ENSMUST00000174299, ENSMUST00000103016, ENSMUST00000005722, ENSMUST00000185355, ENSMUST00000128184, ENSMUST00000105345, ENSMUST00000166775, ENSMUST00000105343, ENSMUST00000135310 |
| KEGG | mmu04380:Osteoclast differentiation | 10 | 1.73310225 | 0.02178533 | ENSMUST00000038859, ENSMUST00000136936, ENSMUST00000106497, ENSMUST00000080933, ENSMUST00000114752, ENSMUST00000102778, ENSMUST00000054002, ENSMUST00000027241, ENSMUST00000063091, ENSMUST00000103202 |
| KEGG | mmu04930:Type II diabetes mellitus | 6 | 1.03986135 | 0.02280926 | ENSMUST00000170690, ENSMUST00000038859, ENSMUST00000107482, ENSMUST00000102778, ENSMUST00000054002, ENSMUST00000052949 |
| KEGG | mmu04664:Fc epsilon RI signaling pathway | 7 | 1.21317158 | 0.02304701 | ENSMUST00000038859, ENSMUST00000106497, ENSMUST00000114752, ENSMUST00000102778, ENSMUST00000064921, ENSMUST00000063091, ENSMUST00000124971 |
| KEGG | mmu04914:Progesterone-mediated oocyte maturation | 8 | 1.3864818 | 0.02312571 | ENSMUST00000038859, ENSMUST00000163966, ENSMUST00000193835, ENSMUST00000114752, ENSMUST00000102778, ENSMUST00000152065, ENSMUST00000168545, ENSMUST00000197074 |
| KEGG | mmu04145:Phagosome | 12 | 2.0797227 | 0.02401272 | ENSMUST00000027639, ENSMUST00000111740, ENSMUST00000048417, ENSMUST00000145791, ENSMUST00000105041, ENSMUST00000169095, ENSMUST00000197890, ENSMUST00000165232, ENSMUST00000172994, ENSMUST00000131405, ENSMUST00000100028, ENSMUST00000170051, ENSMUST00000153541, ENSMUST00000112610, ENSMUST00000146979, ENSMUST00000056427 |
| KEGG | mmu05164:Influenza A | 12 | 2.0797227 | 0.02401272 | ENSMUST00000038859, ENSMUST00000114752, ENSMUST00000135310, ENSMUST00000098924, ENSMUST00000109551, ENSMUST00000102778, ENSMUST00000064921, ENSMUST00000054002, ENSMUST00000168499, ENSMUST00000107362, ENSMUST00000190052, ENSMUST00000025724 |
| KEGG | mmu04912:GnRH signaling pathway | 8 | 1.3864818 | 0.02445136 | ENSMUST00000071816, ENSMUST00000106497, ENSMUST00000114752, ENSMUST00000102778, ENSMUST00000064921, ENSMUST00000152065, ENSMUST00000168545, ENSMUST00000124971 |
| KEGG | mmu01212:Fatty acid metabolism | 6 | 1.03986135 | 0.0246378 | ENSMUST00000034904, ENSMUST00000071402, ENSMUST00000041331, ENSMUST00000110371, ENSMUST00000055655, ENSMUST00000197070, ENSMUST00000103201 |
| KEGG | mmu04360:Axon guidance | 10 | 1.73310225 | 0.02494288 | ENSMUST00000201960, ENSMUST00000191642, ENSMUST00000193835, ENSMUST00000111559, ENSMUST00000166239, ENSMUST00000205822, ENSMUST00000076335, ENSMUST00000063091, ENSMUST00000124971, ENSMUST00000072093 |
| KEGG | mmu00590:Arachidonic acid metabolism | 8 | 1.3864818 | 0.02582835 | ENSMUST00000058785, ENSMUST00000193987, ENSMUST00000035488, ENSMUST00000025968, ENSMUST00000005669, ENSMUST00000176624, ENSMUST00000025966, ENSMUST00000082214 |
| KEGG | mmu00562:Inositol phosphate metabolism | 7 | 1.21317158 | 0.02617404 | ENSMUST00000118390, ENSMUST00000038859, ENSMUST00000131405, ENSMUST00000125476, ENSMUST00000040448, ENSMUST00000028291, ENSMUST00000087657 |
| KEGG | mmu05204:Chemical carcinogenesis | 8 | 1.3864818 | 0.03027529 | ENSMUST00000035488, ENSMUST00000140932, ENSMUST00000025968, ENSMUST00000005669, ENSMUST00000176624, ENSMUST00000126593, ENSMUST00000025966, ENSMUST00000082214 |
| KEGG | mmu01100:Metabolic pathways | 54 | 9.35875217 | 0.03043731 | ENSMUST00000156434, ENSMUST00000035488, ENSMUST00000098657, ENSMUST00000145791, ENSMUST00000136162, ENSMUST00000185466, ENSMUST00000005669, ENSMUST00000040448, ENSMUST00000058785, ENSMUST00000131405, ENSMUST00000128351, ENSMUST00000007131, ENSMUST00000163620, ENSMUST00000142920, ENSMUST00000110371, ENSMUST00000136776, ENSMUST00000089461, ENSMUST00000082214, ENSMUST00000028291, ENSMUST00000118390, ENSMUST00000164763, ENSMUST00000025968, ENSMUST00000165111, ENSMUST00000025966, ENSMUST00000087657, ENSMUST00000071646, ENSMUST00000165067, ENSMUST00000021653, ENSMUST00000120927, ENSMUST00000128694, ENSMUST00000125476, ENSMUST00000021649, ENSMUST00000034992, ENSMUST00000055655, ENSMUST00000074127, ENSMUST00000143108, ENSMUST00000000430, ENSMUST00000113825, ENSMUST00000093350, ENSMUST00000029464, ENSMUST00000034989, ENSMUST00000176624, ENSMUST00000052949, ENSMUST00000026462, ENSMUST00000107482, ENSMUST00000107389, ENSMUST00000105390, ENSMUST00000031766, ENSMUST00000134053, ENSMUST00000103201, ENSMUST00000118917, ENSMUST00000149122, ENSMUST00000113978, ENSMUST00000204714, ENSMUST00000174093, ENSMUST00000159572, ENSMUST00000023393 |
| KEGG | mmu04725:Cholinergic synapse | 9 | 1.55979203 | 0.03109427 | ENSMUST00000067120, ENSMUST00000071816, ENSMUST00000038859, ENSMUST00000193835, ENSMUST00000064921, ENSMUST00000152065, ENSMUST00000168545, ENSMUST00000063091, ENSMUST00000124971 |
| KEGG | mmu04932:Non-alcoholic fatty liver disease (NAFLD) | 11 | 1.90641248 | 0.03248038 | ENSMUST00000123497, ENSMUST00000038859, ENSMUST00000107482, ENSMUST00000166775, ENSMUST00000149122, ENSMUST00000102778, ENSMUST00000054002, ENSMUST00000185466, ENSMUST00000204714, ENSMUST00000051186, ENSMUST00000108411 |
| KEGG | mmu05200:Pathways in cancer | 21 | 3.63951473 | 0.0332308 | ENSMUST00000107357, ENSMUST00000142558, ENSMUST00000119260, ENSMUST00000000312, ENSMUST00000193835, ENSMUST00000106497, ENSMUST00000111740, ENSMUST00000102534, ENSMUST00000144270, ENSMUST00000202138, ENSMUST00000064921, ENSMUST00000124971, ENSMUST00000174299, ENSMUST00000038859, ENSMUST00000166775, ENSMUST00000163966, ENSMUST00000135310, ENSMUST00000138438, ENSMUST00000102778, ENSMUST00000152065, ENSMUST00000168545 |
| KEGG | mmu05169:Epstein-Barr virus infection | 10 | 1.73310225 | 0.03355069 | ENSMUST00000038859, ENSMUST00000105527, ENSMUST00000151644, ENSMUST00000172994, ENSMUST00000080933, ENSMUST00000114752, ENSMUST00000138438, ENSMUST00000102778, ENSMUST00000044078, ENSMUST00000105041, ENSMUST00000172207 |
| KEGG | mmu04010:MAPK signaling pathway | 15 | 2.59965338 | 0.03499906 | ENSMUST00000115228, ENSMUST00000119260, ENSMUST00000021903, ENSMUST00000106497, ENSMUST00000080933, ENSMUST00000102534, ENSMUST00000017435, ENSMUST00000202138, ENSMUST00000064921, ENSMUST00000027241, ENSMUST00000124971, ENSMUST00000005722, ENSMUST00000114752, ENSMUST00000102778, ENSMUST00000130007 |
| KEGG | mmu01200:Carbon metabolism | 9 | 1.55979203 | 0.03555716 | ENSMUST00000177283, ENSMUST00000107482, ENSMUST00000142920, ENSMUST00000113825, ENSMUST00000093350, ENSMUST00000174093, ENSMUST00000029464, ENSMUST00000034989, ENSMUST00000177137, ENSMUST00000052949 |
| KEGG | mmu00620:Pyruvate metabolism | 5 | 0.86655113 | 0.03726042 | ENSMUST00000107482, ENSMUST00000074127, ENSMUST00000113825, ENSMUST00000103201, ENSMUST00000034989 |
| KEGG | mmu04151:PI3K-Akt signaling pathway | 19 | 3.29289428 | 0.0379417 | ENSMUST00000105370, ENSMUST00000142558, ENSMUST00000119260, ENSMUST00000173913, ENSMUST00000106497, ENSMUST00000111740, ENSMUST00000144270, ENSMUST00000128450, ENSMUST00000202138, ENSMUST00000124971, ENSMUST00000178686, ENSMUST00000113870, ENSMUST00000113869, ENSMUST00000038859, ENSMUST00000056925, ENSMUST00000166775, ENSMUST00000163966, ENSMUST00000110697, ENSMUST00000173984, ENSMUST00000051186 |
| KEGG | mmu00260:Glycine, serine and threonine metabolism | 5 | 0.86655113 | 0.04037761 | ENSMUST00000156434, ENSMUST00000118917, ENSMUST00000128351, ENSMUST00000174093, ENSMUST00000134053 |
| KEGG | mmu04915:Estrogen signaling pathway | 8 | 1.3864818 | 0.04065481 | ENSMUST00000038859, ENSMUST00000163966, ENSMUST00000106497, ENSMUST00000193835, ENSMUST00000152065, ENSMUST00000168545, ENSMUST00000124971, ENSMUST00000173984 |
| KEGG | mmu05132:Salmonella infection | 7 | 1.21317158 | 0.04145445 | ENSMUST00000173913, ENSMUST00000114752, ENSMUST00000048417, ENSMUST00000102778, ENSMUST00000100028, ENSMUST00000127694, ENSMUST00000130007 |
| BIOCARTA | m_tpoPathway:TPO Signaling Pathway | 5 | 0.86655113 | 0.04520905 | ENSMUST00000107357, ENSMUST00000106497, ENSMUST00000138438, ENSMUST00000064921, ENSMUST00000124971 |
| BIOCARTA | m_tsp1Pathway:TSP-1 Induced Apoptosis in Microvascular Endothelial Cell | 3 | 0.51993068 | 0.04590473 | ENSMUST00000165232, ENSMUST00000114752, ENSMUST00000170051, ENSMUST00000063091, ENSMUST00000169095, ENSMUST00000197890 |
| KEGG | mmu04141:Protein processing in endoplasmic reticulum | 11 | 1.90641248 | 0.04797805 | ENSMUST00000197291, ENSMUST00000120461, ENSMUST00000163966, ENSMUST00000188346, ENSMUST00000102778, ENSMUST00000172836, ENSMUST00000161085, ENSMUST00000009236, ENSMUST00000146979, ENSMUST00000173984, ENSMUST00000038210 |

**Table 26.** Significant pathways for differentially expressed transcripts in ^56^Fe vs. non-irradiated control at 9 months analyzed by DAVID (<https://david.ncifcrf.gov/>).

| **Database** | **Pathway** | **Count** | **%** | ***p*-value** | **Transcripts** |
| --- | --- | --- | --- | --- | --- |
| KEGG | mmu04931:Insulin resistance | 12 | 2.60303688 | 4.37E-05 | ENSMUST00000038859, ENSMUST00000196469, ENSMUST00000109423, ENSMUST00000102778, ENSMUST00000111160, ENSMUST00000054002, ENSMUST00000107911, ENSMUST00000151705, ENSMUST00000063307, ENSMUST00000051186, ENSMUST00000119299, ENSMUST00000125314 |
| KEGG | mmu05222:Small cell lung cancer | 10 | 2.1691974 | 1.29E-04 | ENSMUST00000120375, ENSMUST00000038859, ENSMUST00000060274, ENSMUST00000196469, ENSMUST00000140436, ENSMUST00000111160, ENSMUST00000114130, ENSMUST00000112930, ENSMUST00000145065, ENSMUST00000125314 |
| KEGG | mmu04380:Osteoclast differentiation | 12 | 2.60303688 | 1.51E-04 | ENSMUST00000115315, ENSMUST00000038859, ENSMUST00000196469, ENSMUST00000113594, ENSMUST00000102778, ENSMUST00000111160, ENSMUST00000043526, ENSMUST00000114130, ENSMUST00000054002, ENSMUST00000038099, ENSMUST00000169017, ENSMUST00000125314 |
| KEGG | mmu04920:Adipocytokine signaling pathway | 9 | 1.95227766 | 2.38E-04 | ENSMUST00000196469, ENSMUST00000109423, ENSMUST00000102778, ENSMUST00000111160, ENSMUST00000114130, ENSMUST00000054002, ENSMUST00000051186, ENSMUST00000025705, ENSMUST00000125314 |
| KEGG | mmu04210:Apoptosis | 8 | 1.73535792 | 4.34E-04 | ENSMUST00000038859, ENSMUST00000156795, ENSMUST00000196469, ENSMUST00000140436, ENSMUST00000111160, ENSMUST00000114130, ENSMUST00000145065, ENSMUST00000125314 |
| KEGG | mmu04668:TNF signaling pathway | 10 | 2.1691974 | 9.10E-04 | ENSMUST00000038859, ENSMUST00000060274, ENSMUST00000196469, ENSMUST00000031327, ENSMUST00000102778, ENSMUST00000111160, ENSMUST00000114130, ENSMUST00000054002, ENSMUST00000056157, ENSMUST00000201245, ENSMUST00000125314 |
| KEGG | mmu05160:Hepatitis C | 11 | 2.38611714 | 0.00117444 | ENSMUST00000038859, ENSMUST00000110874, ENSMUST00000060274, ENSMUST00000196469, ENSMUST00000109423, ENSMUST00000142055, ENSMUST00000102778, ENSMUST00000111160, ENSMUST00000114130, ENSMUST00000054002, ENSMUST00000125314 |
| KEGG | mmu05145:Toxoplasmosis | 9 | 1.95227766 | 0.00290434 | ENSMUST00000196469, ENSMUST00000102778, ENSMUST00000140436, ENSMUST00000111160, ENSMUST00000114130, ENSMUST00000038099, ENSMUST00000145065, ENSMUST00000025705, ENSMUST00000125314 |
| KEGG | mmu05212:Pancreatic cancer | 7 | 1.51843818 | 0.00376615 | ENSMUST00000038859, ENSMUST00000196469, ENSMUST00000102778, ENSMUST00000140436, ENSMUST00000111160, ENSMUST00000114130, ENSMUST00000125314 |
| KEGG | mmu04550:Signaling pathways regulating pluripotency of stem cells | 10 | 2.1691974 | 0.00461854 | ENSMUST00000038859, ENSMUST00000105343, ENSMUST00000020377, ENSMUST00000165164, ENSMUST00000079362, ENSMUST00000122054, ENSMUST00000111160, ENSMUST00000112601, ENSMUST00000105340, ENSMUST00000163421, ENSMUST00000107171, ENSMUST00000025705 |
| KEGG | mmu04622:RIG-I-like receptor signaling pathway | 7 | 1.51843818 | 0.00471462 | ENSMUST00000060274, ENSMUST00000196469, ENSMUST00000142055, ENSMUST00000102778, ENSMUST00000043526, ENSMUST00000114130, ENSMUST00000125314 |
| KEGG | mmu04910:Insulin signaling pathway | 10 | 2.1691974 | 0.00507438 | ENSMUST00000174080, ENSMUST00000113869, ENSMUST00000038859, ENSMUST00000102778, ENSMUST00000111160, ENSMUST00000054002, ENSMUST00000151705, ENSMUST00000038099, ENSMUST00000051186, ENSMUST00000125314 |
| KEGG | mmu04917:Prolactin signaling pathway | 7 | 1.51843818 | 0.00667036 | ENSMUST00000038859, ENSMUST00000196469, ENSMUST00000102778, ENSMUST00000111160, ENSMUST00000054002, ENSMUST00000038099, ENSMUST00000025705 |
| KEGG | mmu04144:Endocytosis | 14 | 3.03687636 | 0.00741146 | ENSMUST00000174050, ENSMUST00000106658, ENSMUST00000112605, ENSMUST00000179755, ENSMUST00000080418, ENSMUST00000001063, ENSMUST00000122054, ENSMUST00000142820, ENSMUST00000108557, ENSMUST00000110416, ENSMUST00000111729, ENSMUST00000113620, ENSMUST00000107236, ENSMUST00000126690, ENSMUST00000179936 |
| KEGG | mmu05231:Choline metabolism in cancer | 8 | 1.73535792 | 0.00875451 | ENSMUST00000113869, ENSMUST00000124107, ENSMUST00000108557, ENSMUST00000038859, ENSMUST00000148833, ENSMUST00000104893, ENSMUST00000102778, ENSMUST00000111160, ENSMUST00000107236 |
| KEGG | mmu05168:Herpes simplex infection | 12 | 2.60303688 | 0.00876547 | ENSMUST00000110874, ENSMUST00000073161, ENSMUST00000060274, ENSMUST00000196469, ENSMUST00000167610, ENSMUST00000142055, ENSMUST00000102778, ENSMUST00000126690, ENSMUST00000114130, ENSMUST00000054002, ENSMUST00000025705, ENSMUST00000125314 |
| KEGG | mmu05221:Acute myeloid leukemia | 6 | 1.30151844 | 0.00924323 | ENSMUST00000038859, ENSMUST00000196469, ENSMUST00000126690, ENSMUST00000111160, ENSMUST00000114130, ENSMUST00000125314 |
| KEGG | mmu05200:Pathways in cancer | 18 | 3.90455531 | 0.00981039 | ENSMUST00000165335, ENSMUST00000060274, ENSMUST00000079362, ENSMUST00000140436, ENSMUST00000122054, ENSMUST00000114130, ENSMUST00000112930, ENSMUST00000107161, ENSMUST00000170223, ENSMUST00000163421, ENSMUST00000125314, ENSMUST00000038859, ENSMUST00000120375, ENSMUST00000196469, ENSMUST00000111160, ENSMUST00000126690, ENSMUST00000102778, ENSMUST00000145065 |
| KEGG | mmu04932:Non-alcoholic fatty liver disease (NAFLD) | 10 | 2.1691974 | 0.01050219 | ENSMUST00000038859, ENSMUST00000196469, ENSMUST00000109423, ENSMUST00000102778, ENSMUST00000111160, ENSMUST00000155377, ENSMUST00000054002, ENSMUST00000051186, ENSMUST00000134479, ENSMUST00000125314 |
| KEGG | mmu05169:Epstein-Barr virus infection | 9 | 1.95227766 | 0.01342362 | ENSMUST00000038859, ENSMUST00000060274, ENSMUST00000196469, ENSMUST00000142055, ENSMUST00000102778, ENSMUST00000111160, ENSMUST00000114130, ENSMUST00000170971, ENSMUST00000125314 |
| KEGG | mmu04062:Chemokine signaling pathway | 11 | 2.38611714 | 0.0154158 | ENSMUST00000038859, ENSMUST00000165335, ENSMUST00000179755, ENSMUST00000152156, ENSMUST00000196469, ENSMUST00000031327, ENSMUST00000111160, ENSMUST00000114130, ENSMUST00000170223, ENSMUST00000201245, ENSMUST00000025705, ENSMUST00000125314 |
| KEGG | mmu05164:Influenza A | 10 | 2.1691974 | 0.01757522 | ENSMUST00000038859, ENSMUST00000110874, ENSMUST00000196469, ENSMUST00000142055, ENSMUST00000102778, ENSMUST00000126690, ENSMUST00000111160, ENSMUST00000054002, ENSMUST00000025705, ENSMUST00000125314 |
| BIOCARTA | m_cd40Pathway:CD40L Signaling Pathway | 4 | 0.86767896 | 0.01798419 | ENSMUST00000060274, ENSMUST00000196469, ENSMUST00000114130, ENSMUST00000125314 |
| KEGG | mmu04064:NF-kappa B signaling pathway | 7 | 1.51843818 | 0.02465598 | ENSMUST00000060274, ENSMUST00000196469, ENSMUST00000142055, ENSMUST00000140436, ENSMUST00000114130, ENSMUST00000145065, ENSMUST00000125314 |
| KEGG | mmu04070:Phosphatidylinositol signaling system | 7 | 1.51843818 | 0.02465598 | ENSMUST00000118390, ENSMUST00000038859, ENSMUST00000110627, ENSMUST00000107236, ENSMUST00000170853, ENSMUST00000137266, ENSMUST00000114621, ENSMUST00000089461, ENSMUST00000033700 |
| KEGG | mmu05220:Chronic myeloid leukemia | 6 | 1.30151844 | 0.02523611 | ENSMUST00000038859, ENSMUST00000196469, ENSMUST00000140436, ENSMUST00000111160, ENSMUST00000114130, ENSMUST00000125314 |
| KEGG | mmu05166:HTLV-I infection | 13 | 2.81995662 | 0.02625485 | ENSMUST00000020377, ENSMUST00000079362, ENSMUST00000140436, ENSMUST00000114130, ENSMUST00000170223, ENSMUST00000125314, ENSMUST00000038859, ENSMUST00000105343, ENSMUST00000196469, ENSMUST00000111160, ENSMUST00000127128, ENSMUST00000170971, ENSMUST00000105340, ENSMUST00000145065, ENSMUST00000146979 |
| KEGG | mmu04930:Type II diabetes mellitus | 5 | 1.0845987 | 0.02795566 | ENSMUST00000038859, ENSMUST00000102778, ENSMUST00000054002, ENSMUST00000038099, ENSMUST00000125314 |
| KEGG | mmu04660:T cell receptor signaling pathway | 7 | 1.51843818 | 0.02934478 | ENSMUST00000038859, ENSMUST00000196469, ENSMUST00000113594, ENSMUST00000111160, ENSMUST00000182755, ENSMUST00000114130, ENSMUST00000125314 |
| KEGG | mmu04620:Toll-like receptor signaling pathway | 7 | 1.51843818 | 0.02934478 | ENSMUST00000038859, ENSMUST00000060274, ENSMUST00000196469, ENSMUST00000102778, ENSMUST00000111160, ENSMUST00000114130, ENSMUST00000125314 |
| BIOCARTA | m_freePathway:Free Radical Induced Apoptosis | 3 | 0.65075922 | 0.03556194 | ENSMUST00000193987, ENSMUST00000196469, ENSMUST00000130881 |
| KEGG | mmu05202:Transcriptional misregulation in cancer | 9 | 1.95227766 | 0.03742878 | ENSMUST00000066854, ENSMUST00000105343, ENSMUST00000115680, ENSMUST00000173689, ENSMUST00000196469, ENSMUST00000020377, ENSMUST00000154666, ENSMUST00000140436, ENSMUST00000126690, ENSMUST00000078137, ENSMUST00000105340, ENSMUST00000113503 |
| BIOCARTA | m_tnfr2Pathway:TNFR2 Signaling Pathway | 4 | 0.86767896 | 0.03931957 | ENSMUST00000060274, ENSMUST00000196469, ENSMUST00000114130, ENSMUST00000125314 |
| KEGG | mmu04621:NOD-like receptor signaling pathway | 5 | 1.0845987 | 0.04239143 | ENSMUST00000196469, ENSMUST00000031327, ENSMUST00000102778, ENSMUST00000114130, ENSMUST00000201245, ENSMUST00000125314 |
| KEGG | mmu04150:mTOR signaling pathway | 5 | 1.0845987 | 0.04716014 | ENSMUST00000113869, ENSMUST00000038859, ENSMUST00000111160, ENSMUST00000051186, ENSMUST00000125314 |

**Table 27.** Significant pathways for differentially expressed transcripts in ^56^Fe vs. non-irradiated control at 12 months analyzed by DAVID (<https://david.ncifcrf.gov/>).

| **Database** | **Pathway** | **Count** | **%** | ***p*-value** | **Transcripts** |
| --- | --- | --- | --- | --- | --- |
| KEGG | mmu01100:Metabolic pathways | 66 | 12.1996303 | 2.05E-05 | ENSMUST00000109194, ENSMUST00000078912, ENSMUST00000164351, ENSMUST00000109943, ENSMUST00000113995, ENSMUST00000007005, ENSMUST00000155749, ENSMUST00000177087, ENSMUST00000114783, ENSMUST00000182267, ENSMUST00000058785, ENSMUST00000007131, ENSMUST00000128351, ENSMUST00000185612, ENSMUST00000199857, ENSMUST00000133320, ENSMUST00000022176, ENSMUST00000142231, ENSMUST00000189503, ENSMUST00000130813, ENSMUST00000118390, ENSMUST00000164763, ENSMUST00000165111, ENSMUST00000193235, ENSMUST00000165234, ENSMUST00000006692, ENSMUST00000139387, ENSMUST00000205983, ENSMUST00000104893, ENSMUST00000107236, ENSMUST00000115137, ENSMUST00000147720, ENSMUST00000137266, ENSMUST00000179869, ENSMUST00000163201, ENSMUST00000123115, ENSMUST00000034785, ENSMUST00000109771, ENSMUST00000200259, ENSMUST00000206589, ENSMUST00000159784, ENSMUST00000156113, ENSMUST00000072818, ENSMUST00000137222, ENSMUST00000143108, ENSMUST00000113290, ENSMUST00000054963, ENSMUST00000170464, ENSMUST00000156713, ENSMUST00000033754, ENSMUST00000077705, ENSMUST00000106348, ENSMUST00000026887, ENSMUST00000052949, ENSMUST00000043735, ENSMUST00000161090, ENSMUST00000030487, ENSMUST00000105390, ENSMUST00000124193, ENSMUST00000006053, ENSMUST00000031082, ENSMUST00000010795, ENSMUST00000025946, ENSMUST00000153808, ENSMUST00000103201, ENSMUST00000001720, ENSMUST00000005685, ENSMUST00000112346, ENSMUST00000067124, ENSMUST00000023805 |
| KEGG | mmu01130:Biosynthesis of antibiotics | 19 | 3.51201479 | 1.24E-04 | ENSMUST00000078912, ENSMUST00000161090, ENSMUST00000113995, ENSMUST00000007005, ENSMUST00000155749, ENSMUST00000165111, ENSMUST00000114783, ENSMUST00000010795, ENSMUST00000001720, ENSMUST00000006692, ENSMUST00000205983, ENSMUST00000128351, ENSMUST00000007131, ENSMUST00000054963, ENSMUST00000156713, ENSMUST00000072585, ENSMUST00000022176, ENSMUST00000106348, ENSMUST00000163201, ENSMUST00000052949, ENSMUST00000179869 |
| KEGG | mmu00830:Retinol metabolism | 11 | 2.03327172 | 4.38E-04 | ENSMUST00000005685, ENSMUST00000058785, ENSMUST00000164351, ENSMUST00000030487, ENSMUST00000072818, ENSMUST00000199857, ENSMUST00000177087, ENSMUST00000025946, ENSMUST00000153808, ENSMUST00000077705, ENSMUST00000070597 |
| KEGG | mmu03320:PPAR signaling pathway | 9 | 1.66358595 | 0.00342764 | ENSMUST00000058785, ENSMUST00000165232, ENSMUST00000030487, ENSMUST00000111319, ENSMUST00000132118, ENSMUST00000041331, ENSMUST00000136234, ENSMUST00000010795, ENSMUST00000109317 |
| KEGG | mmu01212:Fatty acid metabolism | 7 | 1.29390018 | 0.00491995 | ENSMUST00000007005, ENSMUST00000206589, ENSMUST00000041331, ENSMUST00000163837, ENSMUST00000114783, ENSMUST00000010795, ENSMUST00000103201 |
| KEGG | mmu04910:Insulin signaling pathway | 11 | 2.03327172 | 0.01234419 | ENSMUST00000043596, ENSMUST00000113869, ENSMUST00000132555, ENSMUST00000206589, ENSMUST00000150096, ENSMUST00000196397, ENSMUST00000019631, ENSMUST00000106495, ENSMUST00000103201, ENSMUST00000052949, ENSMUST00000125314 |
| KEGG | mmu04144:Endocytosis | 16 | 2.95748614 | 0.01701261 | ENSMUST00000162602, ENSMUST00000106658, ENSMUST00000112605, ENSMUST00000169841, ENSMUST00000167930, ENSMUST00000177083, ENSMUST00000169212, ENSMUST00000001063, ENSMUST00000154686, ENSMUST00000058033, ENSMUST00000124096, ENSMUST00000044234, ENSMUST00000204983, ENSMUST00000135310, ENSMUST00000142147, ENSMUST00000107236, ENSMUST00000161355 |
| BIOCARTA | m_nuclearRsPathway:Nuclear Receptors in Lipid Metabolism and Toxicity | 6 | 1.1090573 | 0.01720257 | ENSMUST00000164351, ENSMUST00000005820, ENSMUST00000042706, ENSMUST00000199857, ENSMUST00000136234, ENSMUST00000025946 |
| KEGG | mmu04622:RIG-I-like receptor signaling pathway | 7 | 1.29390018 | 0.01928593 | ENSMUST00000000284, ENSMUST00000060274, ENSMUST00000190213, ENSMUST00000117269, ENSMUST00000133580, ENSMUST00000164101, ENSMUST00000118375, ENSMUST00000125314 |
| KEGG | mmu00564:Glycerophospholipid metabolism | 8 | 1.47874307 | 0.02769022 | ENSMUST00000149854, ENSMUST00000148833, ENSMUST00000104893, ENSMUST00000112618, ENSMUST00000067124, ENSMUST00000028822, ENSMUST00000105390, ENSMUST00000115137, ENSMUST00000142231, ENSMUST00000127712, ENSMUST00000134936, ENSMUST00000143108 |
| KEGG | mmu05221:Acute myeloid leukemia | 6 | 1.1090573 | 0.03026267 | ENSMUST00000135310, ENSMUST00000005815, ENSMUST00000106495, ENSMUST00000205391, ENSMUST00000164101, ENSMUST00000125314 |
| KEGG | mmu04070:Phosphatidylinositol signaling system | 8 | 1.47874307 | 0.03214802 | ENSMUST00000118390, ENSMUST00000109943, ENSMUST00000107236, ENSMUST00000110626, ENSMUST00000031082, ENSMUST00000142231, ENSMUST00000137266, ENSMUST00000182267, ENSMUST00000130813 |
| KEGG | mmu00900:Terpenoid backbone biosynthesis | 4 | 0.73937153 | 0.0338868 | ENSMUST00000007005, ENSMUST00000022176, ENSMUST00000006692, ENSMUST00000163201, ENSMUST00000179869 |
| KEGG | mmu05202:Transcriptional misregulation in cancer | 11 | 2.03327172 | 0.03429416 | ENSMUST00000051446, ENSMUST00000101695, ENSMUST00000106159, ENSMUST00000079327, ENSMUST00000052368, ENSMUST00000077680, ENSMUST00000079949, ENSMUST00000135352, ENSMUST00000025944, ENSMUST00000115679, ENSMUST00000173689, ENSMUST00000135310, ENSMUST00000205391, ENSMUST00000105340 |
| KEGG | mmu05200:Pathways in cancer | 20 | 3.69685767 | 0.04095968 | ENSMUST00000060274, ENSMUST00000169212, ENSMUST00000111740, ENSMUST00000186057, ENSMUST00000169088, ENSMUST00000170293, ENSMUST00000117269, ENSMUST00000112930, ENSMUST00000005815, ENSMUST00000107161, ENSMUST00000106495, ENSMUST00000164101, ENSMUST00000163421, ENSMUST00000124096, ENSMUST00000125314, ENSMUST00000154356, ENSMUST00000044234, ENSMUST00000072767, ENSMUST00000135310, ENSMUST00000190213, ENSMUST00000205391 |

**Table 28.** Significant pathways for differentially expressed transcripts in ^16^O vs. non-irradiated control at 1 month analyzed by DAVID (<https://david.ncifcrf.gov/>).

| **Database** | **Pathway** | **Count** | **%** | ***p*-value** | **Transcripts** |
| --- | --- | --- | --- | --- | --- |
| KEGG | mmu04066:HIF-1 signaling pathway | 8 | 1.70575693 | 0.01548997 | ENSMUST00000029812, ENSMUST00000073939, ENSMUST00000144235, ENSMUST00000107161, ENSMUST00000179584, ENSMUST00000204807, ENSMUST00000139722, ENSMUST00000072841 |
| KEGG | mmu05202:Transcriptional misregulation in cancer | 10 | 2.13219616 | 0.02566931 | ENSMUST00000018645, ENSMUST00000115680, ENSMUST00000077680, ENSMUST00000115677, ENSMUST00000127481, ENSMUST00000139365, ENSMUST00000168177, ENSMUST00000029812, ENSMUST00000185355, ENSMUST00000105343, ENSMUST00000115679, ENSMUST00000173354, ENSMUST00000204807, ENSMUST00000105340 |
| KEGG | mmu04910:Insulin signaling pathway | 9 | 1.91897655 | 0.02710208 | ENSMUST00000119229, ENSMUST00000113869, ENSMUST00000147781, ENSMUST00000028155, ENSMUST00000033652, ENSMUST00000109822, ENSMUST00000136353, ENSMUST00000196397, ENSMUST00000102778, ENSMUST00000144235, ENSMUST00000178543, ENSMUST00000113870 |
| KEGG | mmu05144:Malaria | 5 | 1.06609808 | 0.03381643 | ENSMUST00000161883, ENSMUST00000165232, ENSMUST00000183610, ENSMUST00000023934, ENSMUST00000174000 |
| KEGG | mmu04151:PI3K-Akt signaling pathway | 16 | 3.41151386 | 0.03548459 | ENSMUST00000073939, ENSMUST00000100036, ENSMUST00000122054, ENSMUST00000055833, ENSMUST00000041097, ENSMUST00000166469, ENSMUST00000188894, ENSMUST00000120141, ENSMUST00000117872, ENSMUST00000139722, ENSMUST00000132525, ENSMUST00000113870, ENSMUST00000113869, ENSMUST00000029812, ENSMUST00000147781, ENSMUST00000120375, ENSMUST00000172521, ENSMUST00000028155, ENSMUST00000132945, ENSMUST00000168746, ENSMUST00000204807 |
| BIOCARTA | M_p38mapkPathway:p38 MAPK Signaling Pathway | 5 | 1.06609808 | 0.04614151 | ENSMUST00000076532, ENSMUST00000144235, ENSMUST00000108184, ENSMUST00000055833, ENSMUST00000076325 |
| KEGG | mmu05132:Salmonella infection | 6 | 1.2793177 | 0.04922208 | ENSMUST00000029812, ENSMUST00000172521, ENSMUST00000132945, ENSMUST00000101454, ENSMUST00000102778, ENSMUST00000100028, ENSMUST00000178543, ENSMUST00000144429 |

**Table 29.** Significant pathways for differentially expressed transcripts in ^16^O vs. non-irradiated control at 2 months analyzed by DAVID (<https://david.ncifcrf.gov/>).

| **Database** | **Pathway** | **Count** | **%** | ***p*-value** | **Transcripts** |
| --- | --- | --- | --- | --- | --- |
| KEGG | mmu04910:Insulin signaling pathway | 13 | 2.78969957 | 1.45E-04 | ENSMUST00000047111, ENSMUST00000134660, ENSMUST00000107482, ENSMUST00000206589, ENSMUST00000019469, ENSMUST00000122022, ENSMUST00000055655, ENSMUST00000124758, ENSMUST00000113611, ENSMUST00000172070, ENSMUST00000087321, ENSMUST00000125314, ENSMUST00000113869, ENSMUST00000031583, ENSMUST00000102582, ENSMUST00000144235, ENSMUST00000100784, ENSMUST00000106513 |
| KEGG | mmu05221:Acute myeloid leukemia | 6 | 1.28755365 | 0.01129083 | ENSMUST00000107357, ENSMUST00000114133, ENSMUST00000135310, ENSMUST00000205391, ENSMUST00000107403, ENSMUST00000125314 |
| KEGG | mmu04380:Osteoclast differentiation | 9 | 1.93133047 | 0.01151523 | ENSMUST00000114133, ENSMUST00000080933, ENSMUST00000164591, ENSMUST00000105238, ENSMUST00000114795, ENSMUST00000169017, ENSMUST00000103203, ENSMUST00000125314, ENSMUST00000044234 |
| KEGG | mmu04621:NOD-like receptor signaling pathway | 6 | 1.28755365 | 0.01213891 | ENSMUST00000094361, ENSMUST00000114133, ENSMUST00000080933, ENSMUST00000031327, ENSMUST00000190213, ENSMUST00000201245, ENSMUST00000125314 |
| KEGG | mmu05200:Pathways in cancer | 18 | 3.86266094 | 0.01550749 | ENSMUST00000107357, ENSMUST00000080469, ENSMUST00000165335, ENSMUST00000094361, ENSMUST00000161317, ENSMUST00000155285, ENSMUST00000114133, ENSMUST00000060274, ENSMUST00000174299, ENSMUST00000125314, ENSMUST00000044234, ENSMUST00000135310, ENSMUST00000190213, ENSMUST00000005719, ENSMUST00000186940, ENSMUST00000205391, ENSMUST00000168162, ENSMUST00000107403, ENSMUST00000105263 |
| BIOCARTA | m_nthiPathway:NFkB activation by Nontypeable Hemophilus influenzae | 5 | 1.07296137 | 0.01696356 | ENSMUST00000025300, ENSMUST00000080933, ENSMUST00000130269, ENSMUST00000125314, ENSMUST00000044234 |
| KEGG | mmu04931:Insulin resistance | 8 | 1.7167382 | 0.01745689 | ENSMUST00000031583, ENSMUST00000134660, ENSMUST00000019469, ENSMUST00000102582, ENSMUST00000159951, ENSMUST00000124758, ENSMUST00000107911, ENSMUST00000125314, ENSMUST00000087321 |
| KEGG | mmu04622:RIG-I-like receptor signaling pathway | 6 | 1.28755365 | 0.02443817 | ENSMUST00000000284, ENSMUST00000114133, ENSMUST00000060274, ENSMUST00000080933, ENSMUST00000190213, ENSMUST00000125314 |
| KEGG | mmu00564:Glycerophospholipid metabolism | 7 | 1.50214592 | 0.0266039 | ENSMUST00000166187, ENSMUST00000105390, ENSMUST00000061856, ENSMUST00000128694, ENSMUST00000142957, ENSMUST00000153238, ENSMUST00000138194 |
| KEGG | mmu05168:Herpes simplex infection | 11 | 2.36051502 | 0.03016996 | ENSMUST00000114133, ENSMUST00000060274, ENSMUST00000080933, ENSMUST00000135310, ENSMUST00000190213, ENSMUST00000164591, ENSMUST00000114311, ENSMUST00000105238, ENSMUST00000167084, ENSMUST00000166748, ENSMUST00000125314 |
| KEGG | mmu04520:Adherens junction | 6 | 1.28755365 | 0.03037534 | ENSMUST00000026921, ENSMUST00000080469, ENSMUST00000080933, ENSMUST00000108752, ENSMUST00000150848, ENSMUST00000124758, ENSMUST00000161355, ENSMUST00000044234 |
| KEGG | mmu04070:Phosphatidylinositol signaling system | 7 | 1.50214592 | 0.0304398 | ENSMUST00000118390, ENSMUST00000092802, ENSMUST00000150857, ENSMUST00000161854, ENSMUST00000114621, ENSMUST00000153238, ENSMUST00000138194 |
| KEGG | mmu04064:NF-kappa B signaling pathway | 7 | 1.50214592 | 0.0304398 | ENSMUST00000000284, ENSMUST00000114133, ENSMUST00000060274, ENSMUST00000080933, ENSMUST00000184864, ENSMUST00000114795, ENSMUST00000125314 |
| KEGG | mmu00310:Lysine degradation | 5 | 1.07296137 | 0.03703967 | ENSMUST00000114432, ENSMUST00000066854, ENSMUST00000178486, ENSMUST00000094962, ENSMUST00000107171 |
| KEGG | mmu05169:Epstein-Barr virus infection | 8 | 1.7167382 | 0.04764571 | ENSMUST00000155285, ENSMUST00000114133, ENSMUST00000060274, ENSMUST00000080933, ENSMUST00000044078, ENSMUST00000114311, ENSMUST00000130269, ENSMUST00000125314, ENSMUST00000105263 |
| KEGG | mmu05202:Transcriptional misregulation in cancer | 9 | 1.93133047 | 0.0477343 | ENSMUST00000066854, ENSMUST00000105342, ENSMUST00000080469, ENSMUST00000155285, ENSMUST00000135310, ENSMUST00000052368, ENSMUST00000174299, ENSMUST00000205391, ENSMUST00000107403, ENSMUST00000105263 |
| KEGG | mmu04668:TNF signaling pathway | 7 | 1.50214592 | 0.04932603 | ENSMUST00000114133, ENSMUST00000060274, ENSMUST00000080933, ENSMUST00000031327, ENSMUST00000190213, ENSMUST00000201245, ENSMUST00000130269, ENSMUST00000125314 |

**Table 30.** Significant pathways for differentially expressed transcripts in ^16^O vs. non-irradiated control at 4 months analyzed by DAVID (<https://david.ncifcrf.gov/>).

| **Database** | **Pathway** | **Count** | **%** | ***p*-value** | **Transcripts** |
| --- | --- | --- | --- | --- | --- |
| KEGG | mmu00830:Retinol metabolism | 12 | 2.51572327 | 3.40E-05 | ENSMUST00000058785, ENSMUST00000075858, ENSMUST00000071646, ENSMUST00000094886, ENSMUST00000035488, ENSMUST00000072818, ENSMUST00000098657, ENSMUST00000025968, ENSMUST00000176364, ENSMUST00000005669, ENSMUST00000176624, ENSMUST00000026462, ENSMUST00000082214 |
| KEGG | mmu01100:Metabolic pathways | 56 | 11.7400419 | 3.85E-04 | ENSMUST00000156434, ENSMUST00000035488, ENSMUST00000098657, ENSMUST00000185466, ENSMUST00000135441, ENSMUST00000005669, ENSMUST00000130881, ENSMUST00000058785, ENSMUST00000102582, ENSMUST00000128351, ENSMUST00000007131, ENSMUST00000163620, ENSMUST00000110371, ENSMUST00000136776, ENSMUST00000089461, ENSMUST00000082214, ENSMUST00000118390, ENSMUST00000025968, ENSMUST00000165111, ENSMUST00000087657, ENSMUST00000071646, ENSMUST00000075858, ENSMUST00000163602, ENSMUST00000165067, ENSMUST00000021653, ENSMUST00000120927, ENSMUST00000049207, ENSMUST00000202779, ENSMUST00000199519, ENSMUST00000082407, ENSMUST00000082408, ENSMUST00000200259, ENSMUST00000094886, ENSMUST00000206589, ENSMUST00000020253, ENSMUST00000034992, ENSMUST00000072818, ENSMUST00000055655, ENSMUST00000145216, ENSMUST00000074127, ENSMUST00000126751, ENSMUST00000143108, ENSMUST00000170464, ENSMUST00000146103, ENSMUST00000029464, ENSMUST00000176624, ENSMUST00000034989, ENSMUST00000026462, ENSMUST00000045289, ENSMUST00000107389, ENSMUST00000105390, ENSMUST00000031766, ENSMUST00000124832, ENSMUST00000134053, ENSMUST00000103201, ENSMUST00000074082, ENSMUST00000031583, ENSMUST00000151952, ENSMUST00000025391, ENSMUST00000168120, ENSMUST00000204714, ENSMUST00000174093, ENSMUST00000023684, ENSMUST00000159572 |
| KEGG | mmu05204:Chemical carcinogenesis | 10 | 2.09643606 | 0.00102486 | ENSMUST00000075858, ENSMUST00000035488, ENSMUST00000072818, ENSMUST00000169613, ENSMUST00000025968, ENSMUST00000199519, ENSMUST00000111068, ENSMUST00000005669, ENSMUST00000176624, ENSMUST00000082214 |
| KEGG | mmu00140:Steroid hormone biosynthesis | 9 | 1.88679245 | 0.002874 | ENSMUST00000075858, ENSMUST00000035488, ENSMUST00000072818, ENSMUST00000025968, ENSMUST00000035625, ENSMUST00000005669, ENSMUST00000176624, ENSMUST00000026462, ENSMUST00000082214 |
| KEGG | mmu00062:Fatty acid elongation | 5 | 1.04821803 | 0.00547533 | ENSMUST00000034904, ENSMUST00000071402, ENSMUST00000021653, ENSMUST00000120927, ENSMUST00000168120, ENSMUST00000006557 |
| KEGG | mmu00061:Fatty acid biosynthesis | 4 | 0.83857442 | 0.0062429 | ENSMUST00000031583, ENSMUST00000206589, ENSMUST00000102582, ENSMUST00000110371, ENSMUST00000055655, ENSMUST00000103201 |
| KEGG | mmu01040:Biosynthesis of unsaturated fatty acids | 5 | 1.04821803 | 0.00628915 | ENSMUST00000034904, ENSMUST00000071402, ENSMUST00000041331, ENSMUST00000021653, ENSMUST00000120927, ENSMUST00000168120 |
| KEGG | mmu04152:AMPK signaling pathway | 10 | 2.09643606 | 0.00949587 | ENSMUST00000105370, ENSMUST00000206589, ENSMUST00000080933, ENSMUST00000055655, ENSMUST00000169095, ENSMUST00000103201, ENSMUST00000197890, ENSMUST00000031583, ENSMUST00000165232, ENSMUST00000102582, ENSMUST00000041331, ENSMUST00000108242, ENSMUST00000170051, ENSMUST00000179584, ENSMUST00000051186 |
| KEGG | mmu01212:Fatty acid metabolism | 6 | 1.25786164 | 0.01347837 | ENSMUST00000034904, ENSMUST00000071402, ENSMUST00000206589, ENSMUST00000041331, ENSMUST00000110371, ENSMUST00000055655, ENSMUST00000103201 |
| KEGG | mmu05202:Transcriptional misregulation in cancer | 11 | 2.30607966 | 0.01712307 | ENSMUST00000128184, ENSMUST00000185355, ENSMUST00000080469, ENSMUST00000023151, ENSMUST00000135310, ENSMUST00000114689, ENSMUST00000204807, ENSMUST00000205391, ENSMUST00000005722, ENSMUST00000105263, ENSMUST00000120105 |
| KEGG | mmu00590:Arachidonic acid metabolism | 7 | 1.46750524 | 0.03794645 | ENSMUST00000058785, ENSMUST00000193987, ENSMUST00000094886, ENSMUST00000035488, ENSMUST00000025968, ENSMUST00000005669, ENSMUST00000176624, ENSMUST00000082214 |

**Table 31.** Significant pathways for differentially expressed transcripts in ^16^O vs. non-irradiated control at 9 months analyzed by DAVID (<https://david.ncifcrf.gov/>).

| **Database** | **Pathway** | **Count** | **%** | ***p*-value** | **Transcripts** |
| --- | --- | --- | --- | --- | --- |
| KEGG | mmu04380:Osteoclast differentiation | 14 | 2.2875817 | 1.43E-04 | ENSMUST00000146084, ENSMUST00000064922, ENSMUST00000043526, ENSMUST00000114130, ENSMUST00000027241, ENSMUST00000038099, ENSMUST00000114795, ENSMUST00000038859, ENSMUST00000196469, ENSMUST00000113594, ENSMUST00000111160, ENSMUST00000054002, ENSMUST00000178543, ENSMUST00000004330, ENSMUST00000107094, ENSMUST00000169017 |
| KEGG | mmu05161:Hepatitis B | 15 | 2.45098039 | 1.75E-04 | ENSMUST00000114171, ENSMUST00000114130, ENSMUST00000041362, ENSMUST00000027009, ENSMUST00000038859, ENSMUST00000023829, ENSMUST00000196469, ENSMUST00000110361, ENSMUST00000029623, ENSMUST00000142055, ENSMUST00000111160, ENSMUST00000204807, ENSMUST00000100009, ENSMUST00000178543, ENSMUST00000004330, ENSMUST00000107094 |
| KEGG | mmu05164:Influenza A | 16 | 2.61437908 | 2.77E-04 | ENSMUST00000146084, ENSMUST00000112916, ENSMUST00000041362, ENSMUST00000025705, ENSMUST00000025724, ENSMUST00000038859, ENSMUST00000172549, ENSMUST00000110874, ENSMUST00000196469, ENSMUST00000142055, ENSMUST00000111160, ENSMUST00000054002, ENSMUST00000100009, ENSMUST00000178543, ENSMUST00000180021, ENSMUST00000107094 |
| KEGG | mmu04668:TNF signaling pathway | 12 | 1.96078431 | 5.61E-04 | ENSMUST00000146084, ENSMUST00000031327, ENSMUST00000064922, ENSMUST00000114130, ENSMUST00000038859, ENSMUST00000105527, ENSMUST00000196469, ENSMUST00000111160, ENSMUST00000054002, ENSMUST00000100009, ENSMUST00000178543, ENSMUST00000201245, ENSMUST00000004330, ENSMUST00000107094 |
| KEGG | mmu05202:Transcriptional misregulation in cancer | 15 | 2.45098039 | 6.19E-04 | ENSMUST00000018645, ENSMUST00000020377, ENSMUST00000154666, ENSMUST00000114689, ENSMUST00000174408, ENSMUST00000113500, ENSMUST00000120105, ENSMUST00000105344, ENSMUST00000105342, ENSMUST00000093852, ENSMUST00000105343, ENSMUST00000166775, ENSMUST00000023829, ENSMUST00000196469, ENSMUST00000109820, ENSMUST00000061829, ENSMUST00000204807, ENSMUST00000105340, ENSMUST00000031256 |
| KEGG | mmu05169:Epstein-Barr virus infection | 13 | 2.12418301 | 0.00106911 | ENSMUST00000146084, ENSMUST00000038859, ENSMUST00000105527, ENSMUST00000023829, ENSMUST00000196469, ENSMUST00000142055, ENSMUST00000111160, ENSMUST00000044078, ENSMUST00000114130, ENSMUST00000204807, ENSMUST00000178543, ENSMUST00000025181, ENSMUST00000004330, ENSMUST00000107094 |
| KEGG | mmu05222:Small cell lung cancer | 10 | 1.63398693 | 0.00115654 | ENSMUST00000038859, ENSMUST00000166775, ENSMUST00000196469, ENSMUST00000109820, ENSMUST00000111160, ENSMUST00000114130, ENSMUST00000112930, ENSMUST00000186940, ENSMUST00000204807, ENSMUST00000145065, ENSMUST00000004330 |
| KEGG | mmu05152:Tuberculosis | 15 | 2.45098039 | 0.0011693 | ENSMUST00000114171, ENSMUST00000089022, ENSMUST00000167610, ENSMUST00000109491, ENSMUST00000145791, ENSMUST00000025705, ENSMUST00000152541, ENSMUST00000016168, ENSMUST00000131405, ENSMUST00000196469, ENSMUST00000029623, ENSMUST00000111160, ENSMUST00000061829, ENSMUST00000040259, ENSMUST00000178543, ENSMUST00000180021, ENSMUST00000169017 |
| KEGG | mmu04066:HIF-1 signaling pathway | 11 | 1.79738562 | 0.00124608 | ENSMUST00000153665, ENSMUST00000114846, ENSMUST00000107161, ENSMUST00000192949, ENSMUST00000041388, ENSMUST00000199832, ENSMUST00000038859, ENSMUST00000023829, ENSMUST00000196469, ENSMUST00000005548, ENSMUST00000111160, ENSMUST00000112645, ENSMUST00000204807, ENSMUST00000179584, ENSMUST00000192844, ENSMUST00000077523 |
| KEGG | mmu05168:Herpes simplex infection | 16 | 2.61437908 | 0.00210874 | ENSMUST00000073161, ENSMUST00000167610, ENSMUST00000111261, ENSMUST00000114130, ENSMUST00000025181, ENSMUST00000041362, ENSMUST00000025705, ENSMUST00000059042, ENSMUST00000110874, ENSMUST00000200944, ENSMUST00000196469, ENSMUST00000029623, ENSMUST00000142055, ENSMUST00000054002, ENSMUST00000110352, ENSMUST00000178543, ENSMUST00000166748, ENSMUST00000004330, ENSMUST00000107094 |
| KEGG | mmu05132:Salmonella infection | 9 | 1.47058824 | 0.00285078 | ENSMUST00000152541, ENSMUST00000016168, ENSMUST00000114299, ENSMUST00000196469, ENSMUST00000031327, ENSMUST00000109491, ENSMUST00000061829, ENSMUST00000178543, ENSMUST00000201245, ENSMUST00000180021, ENSMUST00000107094, ENSMUST00000030751, ENSMUST00000144429 |
| KEGG | mmu05160:Hepatitis C | 12 | 1.96078431 | 0.00340846 | ENSMUST00000177648, ENSMUST00000169391, ENSMUST00000114130, ENSMUST00000041362, ENSMUST00000038859, ENSMUST00000110874, ENSMUST00000023829, ENSMUST00000166775, ENSMUST00000196469, ENSMUST00000142055, ENSMUST00000111160, ENSMUST00000054002, ENSMUST00000178543, ENSMUST00000004330 |
| KEGG | mmu04620:Toll-like receptor signaling pathway | 10 | 1.63398693 | 0.00415173 | ENSMUST00000152541, ENSMUST00000146084, ENSMUST00000038859, ENSMUST00000016168, ENSMUST00000196469, ENSMUST00000029623, ENSMUST00000109491, ENSMUST00000111160, ENSMUST00000114130, ENSMUST00000061829, ENSMUST00000178543, ENSMUST00000004330, ENSMUST00000107094 |
| KEGG | mmu05145:Toxoplasmosis | 10 | 1.63398693 | 0.00536639 | ENSMUST00000146084, ENSMUST00000196469, ENSMUST00000029623, ENSMUST00000109820, ENSMUST00000111160, ENSMUST00000114130, ENSMUST00000038099, ENSMUST00000178543, ENSMUST00000145065, ENSMUST00000004330, ENSMUST00000025705 |
| KEGG | mmu05200:Pathways in cancer | 23 | 3.75816993 | 0.00580974 | ENSMUST00000135398, ENSMUST00000122054, ENSMUST00000112930, ENSMUST00000170223, ENSMUST00000163421, ENSMUST00000020706, ENSMUST00000024967, ENSMUST00000038859, ENSMUST00000093852, ENSMUST00000166775, ENSMUST00000204807, ENSMUST00000186940, ENSMUST00000098683, ENSMUST00000107094, ENSMUST00000165335, ENSMUST00000119260, ENSMUST00000107161, ENSMUST00000114130, ENSMUST00000023829, ENSMUST00000196469, ENSMUST00000111160, ENSMUST00000109820, ENSMUST00000178543, ENSMUST00000171509, ENSMUST00000145065, ENSMUST00000004330 |
| KEGG | mmu04151:PI3K-Akt signaling pathway | 21 | 3.43137255 | 0.00659584 | ENSMUST00000119260, ENSMUST00000165335, ENSMUST00000122054, ENSMUST00000114130, ENSMUST00000022746, ENSMUST00000112930, ENSMUST00000025705, ENSMUST00000038859, ENSMUST00000056925, ENSMUST00000166775, ENSMUST00000023829, ENSMUST00000196469, ENSMUST00000110361, ENSMUST00000029623, ENSMUST00000109820, ENSMUST00000111160, ENSMUST00000020308, ENSMUST00000186940, ENSMUST00000204807, ENSMUST00000100009, ENSMUST00000171509, ENSMUST00000051186, ENSMUST00000004330 |
| KEGG | mmu04920:Adipocytokine signaling pathway | 8 | 1.30718954 | 0.00692871 | ENSMUST00000166775, ENSMUST00000196469, ENSMUST00000111160, ENSMUST00000114130, ENSMUST00000054002, ENSMUST00000178543, ENSMUST00000051186, ENSMUST00000004330, ENSMUST00000025705 |
| KEGG | mmu04931:Insulin resistance | 10 | 1.63398693 | 0.00725264 | ENSMUST00000038859, ENSMUST00000196469, ENSMUST00000034267, ENSMUST00000111160, ENSMUST00000054002, ENSMUST00000151705, ENSMUST00000029053, ENSMUST00000063307, ENSMUST00000178543, ENSMUST00000167197, ENSMUST00000051186 |
| KEGG | mmu05134:Legionellosis | 7 | 1.14379085 | 0.00832296 | ENSMUST00000072838, ENSMUST00000196469, ENSMUST00000031327, ENSMUST00000029623, ENSMUST00000061829, ENSMUST00000201245, ENSMUST00000020153, ENSMUST00000180021 |
| KEGG | mmu04064:NF-kappa B signaling pathway | 9 | 1.47058824 | 0.01061151 | ENSMUST00000152541, ENSMUST00000016168, ENSMUST00000105527, ENSMUST00000196469, ENSMUST00000142055, ENSMUST00000109491, ENSMUST00000109820, ENSMUST00000114130, ENSMUST00000061829, ENSMUST00000027241, ENSMUST00000145065, ENSMUST00000004330, ENSMUST00000114795 |
| KEGG | mmu04210:Apoptosis | 7 | 1.14379085 | 0.01063024 | ENSMUST00000038859, ENSMUST00000196469, ENSMUST00000109820, ENSMUST00000111160, ENSMUST00000114130, ENSMUST00000145065, ENSMUST00000004330, ENSMUST00000027009 |
| KEGG | mmu04010:MAPK signaling pathway | 16 | 2.61437908 | 0.0120121 | ENSMUST00000088345, ENSMUST00000146084, ENSMUST00000119260, ENSMUST00000021903, ENSMUST00000039926, ENSMUST00000122054, ENSMUST00000114130, ENSMUST00000027241, ENSMUST00000114795, ENSMUST00000114299, ENSMUST00000196469, ENSMUST00000151678, ENSMUST00000111160, ENSMUST00000061829, ENSMUST00000100009, ENSMUST00000178543, ENSMUST00000171509, ENSMUST00000004330, ENSMUST00000107094, ENSMUST00000132480, ENSMUST00000144429 |
| KEGG | mmu04910:Insulin signaling pathway | 11 | 1.79738562 | 0.01201422 | ENSMUST00000174080, ENSMUST00000153665, ENSMUST00000038859, ENSMUST00000122022, ENSMUST00000111160, ENSMUST00000054002, ENSMUST00000151705, ENSMUST00000021147, ENSMUST00000029053, ENSMUST00000038099, ENSMUST00000178543, ENSMUST00000051186 |
| KEGG | mmu04623:Cytosolic DNA-sensing pathway | 7 | 1.14379085 | 0.01436736 | ENSMUST00000196469, ENSMUST00000142055, ENSMUST00000180358, ENSMUST00000114130, ENSMUST00000041362, ENSMUST00000004330, ENSMUST00000180021, ENSMUST00000025724 |
| KEGG | mmu05142:Chagas disease (American trypanosomiasis) | 9 | 1.47058824 | 0.01490008 | ENSMUST00000020706, ENSMUST00000199832, ENSMUST00000038859, ENSMUST00000196469, ENSMUST00000029623, ENSMUST00000135398, ENSMUST00000111160, ENSMUST00000114130, ENSMUST00000178543, ENSMUST00000004330, ENSMUST00000041388, ENSMUST00000077523, ENSMUST00000107094 |
| BIOCARTA | m_eponfkbPathway:Erythropoietin mediated neuroprotection through NF-kB | 4 | 0.65359477 | 0.01708398 | ENSMUST00000023829, ENSMUST00000196469, ENSMUST00000107161, ENSMUST00000025705 |
| KEGG | mmu05220:Chronic myeloid leukemia | 7 | 1.14379085 | 0.02440666 | ENSMUST00000038859, ENSMUST00000023829, ENSMUST00000196469, ENSMUST00000109820, ENSMUST00000111160, ENSMUST00000114130, ENSMUST00000204807, ENSMUST00000004330 |
| KEGG | mmu04917:Prolactin signaling pathway | 7 | 1.14379085 | 0.02592378 | ENSMUST00000038859, ENSMUST00000196469, ENSMUST00000111160, ENSMUST00000054002, ENSMUST00000038099, ENSMUST00000178543, ENSMUST00000025705 |
| KEGG | mmu05162:Measles | 10 | 1.63398693 | 0.02615592 | ENSMUST00000038859, ENSMUST00000105527, ENSMUST00000110874, ENSMUST00000196469, ENSMUST00000142055, ENSMUST00000029623, ENSMUST00000111160, ENSMUST00000204807, ENSMUST00000041362, ENSMUST00000025705 |
| BIOCARTA | M_il1rPathway:Signal transduction through IL1R | 6 | 0.98039216 | 0.02641275 | ENSMUST00000146084, ENSMUST00000114482, ENSMUST00000089022, ENSMUST00000196469, ENSMUST00000027241, ENSMUST00000114795, ENSMUST00000107094 |
| KEGG | mmu04550:Signaling pathways regulating pluripotency of stem cells | 10 | 1.63398693 | 0.02840286 | ENSMUST00000119260, ENSMUST00000020377, ENSMUST00000038765, ENSMUST00000122054, ENSMUST00000118470, ENSMUST00000163421, ENSMUST00000025705, ENSMUST00000105344, ENSMUST00000038859, ENSMUST00000105342, ENSMUST00000105343, ENSMUST00000111160, ENSMUST00000105340, ENSMUST00000015841, ENSMUST00000171509 |
| KEGG | mmu04621:NOD-like receptor signaling pathway | 6 | 0.98039216 | 0.03186125 | ENSMUST00000105527, ENSMUST00000196469, ENSMUST00000031327, ENSMUST00000114130, ENSMUST00000178543, ENSMUST00000201245, ENSMUST00000004330, ENSMUST00000180021 |
| KEGG | mmu04978:Mineral absorption | 5 | 0.81699346 | 0.03236426 | ENSMUST00000028843, ENSMUST00000034214, ENSMUST00000034215, ENSMUST00000005548, ENSMUST00000112645 |
| KEGG | mmu05231:Choline metabolism in cancer | 8 | 1.30718954 | 0.03807534 | ENSMUST00000126074, ENSMUST00000038859, ENSMUST00000104893, ENSMUST00000074729, ENSMUST00000161854, ENSMUST00000111160, ENSMUST00000178543, ENSMUST00000107094, ENSMUST00000143108 |
| KEGG | mmu05210:Colorectal cancer | 6 | 0.98039216 | 0.04880085 | ENSMUST00000024967, ENSMUST00000038859, ENSMUST00000111160, ENSMUST00000178543, ENSMUST00000163421, ENSMUST00000107094 |
| KEGG | mmu05166:HTLV-I infection | 15 | 2.45098039 | 0.04997839 | ENSMUST00000020377, ENSMUST00000135398, ENSMUST00000114130, ENSMUST00000170223, ENSMUST00000027241, ENSMUST00000025181, ENSMUST00000114795, ENSMUST00000020706, ENSMUST00000105344, ENSMUST00000038859, ENSMUST00000105342, ENSMUST00000105343, ENSMUST00000023829, ENSMUST00000196469, ENSMUST00000109820, ENSMUST00000111160, ENSMUST00000100009, ENSMUST00000105340, ENSMUST00000145065, ENSMUST00000146979, ENSMUST00000004330, ENSMUST00000107094 |

**Table 32.** Significant pathways for differentially expressed transcripts in ^16^O vs. non-irradiated control at 12 months analyzed by DAVID (<https://david.ncifcrf.gov/>).

| **Database** | **Pathway** | **Count** | **%** | ***p*-value** | **Transcripts** |
| --- | --- | --- | --- | --- | --- |
| KEGG | mmu01100:Metabolic pathways | 69 | 10.6481481 | 6.54E-05 | ENSMUST00000109194, ENSMUST00000078912, ENSMUST00000156434, ENSMUST00000113995, ENSMUST00000045376, ENSMUST00000168419, ENSMUST00000177087, ENSMUST00000114783, ENSMUST00000019986, ENSMUST00000031637, ENSMUST00000126440, ENSMUST00000022858, ENSMUST00000136776, ENSMUST00000135642, ENSMUST00000169202, ENSMUST00000134104, ENSMUST00000106701, ENSMUST00000146818, ENSMUST00000137540, ENSMUST00000133320, ENSMUST00000115242, ENSMUST00000142231, ENSMUST00000145091, ENSMUST00000169273, ENSMUST00000006838, ENSMUST00000047490, ENSMUST00000164763, ENSMUST00000189211, ENSMUST00000165111, ENSMUST00000080283, ENSMUST00000025760, ENSMUST00000207851, ENSMUST00000135772, ENSMUST00000163602, ENSMUST00000177400, ENSMUST00000010191, ENSMUST00000049207, ENSMUST00000147720, ENSMUST00000078155, ENSMUST00000163201, ENSMUST00000159096, ENSMUST00000135379, ENSMUST00000149290, ENSMUST00000139353, ENSMUST00000034785, ENSMUST00000200259, ENSMUST00000173409, ENSMUST00000206637, ENSMUST00000137222, ENSMUST00000075630, ENSMUST00000169966, ENSMUST00000143741, ENSMUST00000072841, ENSMUST00000108557, ENSMUST00000127439, ENSMUST00000047816, ENSMUST00000113290, ENSMUST00000170464, ENSMUST00000147277, ENSMUST00000033754, ENSMUST00000174214, ENSMUST00000077705, ENSMUST00000029905, ENSMUST00000006053, ENSMUST00000153808, ENSMUST00000205865, ENSMUST00000135943, ENSMUST00000124834, ENSMUST00000166804, ENSMUST00000074082, ENSMUST00000170287, ENSMUST00000146580, ENSMUST00000149122, ENSMUST00000005477, ENSMUST00000070334, ENSMUST00000168546, ENSMUST00000023393, ENSMUST00000126248, ENSMUST00000189437 |
| KEGG | mmu05168:Herpes simplex infection | 17 | 2.62345679 | 0.00206814 | ENSMUST00000132555, ENSMUST00000163506, ENSMUST00000111261, ENSMUST00000186057, ENSMUST00000107920, ENSMUST00000164101, ENSMUST00000173888, ENSMUST00000103204, ENSMUST00000047321, ENSMUST00000069620, ENSMUST00000101004, ENSMUST00000160405, ENSMUST00000107834, ENSMUST00000054556, ENSMUST00000081491, ENSMUST00000174513, ENSMUST00000166603, ENSMUST00000110937, ENSMUST00000020550, ENSMUST00000184550, ENSMUST00000166748 |
| KEGG | mmu01130:Biosynthesis of antibiotics | 17 | 2.62345679 | 0.0027604 | ENSMUST00000078912, ENSMUST00000145091, ENSMUST00000113995, ENSMUST00000173409, ENSMUST00000165111, ENSMUST00000114783, ENSMUST00000205865, ENSMUST00000135943, ENSMUST00000126440, ENSMUST00000143741, ENSMUST00000169966, ENSMUST00000072841, ENSMUST00000170287, ENSMUST00000177400, ENSMUST00000136776, ENSMUST00000169202, ENSMUST00000174214, ENSMUST00000163201, ENSMUST00000159096, ENSMUST00000111168 |
| KEGG | mmu05202:Transcriptional misregulation in cancer | 13 | 2.00617284 | 0.0111392 | ENSMUST00000051446, ENSMUST00000080469, ENSMUST00000115680, ENSMUST00000106159, ENSMUST00000079327, ENSMUST00000052368, ENSMUST00000079949, ENSMUST00000135352, ENSMUST00000137191, ENSMUST00000069620, ENSMUST00000173689, ENSMUST00000159789, ENSMUST00000205391, ENSMUST00000184550 |
| KEGG | mmu03320:PPAR signaling pathway | 8 | 1.2345679 | 0.0195428 | ENSMUST00000123744, ENSMUST00000135379, ENSMUST00000111319, ENSMUST00000128408, ENSMUST00000029905, ENSMUST00000135642, ENSMUST00000106701, ENSMUST00000197890, ENSMUST00000109317 |
| KEGG | mmu04710:Circadian rhythm | 5 | 0.77160494 | 0.02059686 | ENSMUST00000069620, ENSMUST00000101004, ENSMUST00000190348, ENSMUST00000103204, ENSMUST00000166748, ENSMUST00000047321 |
| BIOCARTA | m_malatexPathway:Shuttle for transfer of acetyl groups from mitochondria to the cytosol | 3 | 0.46296296 | 0.04979421 | ENSMUST00000142022, ENSMUST00000165111, ENSMUST00000126440 |

**Table 33.** Significant pathways for differentially expressed transcripts in ^28^Si vs. non-irradiated control at 1 month analyzed by DAVID (<https://david.ncifcrf.gov/>).

| **Database** | **Pathway** | **Count** | **%** | ***p*-value** | **Transcripts** |
| --- | --- | --- | --- | --- | --- |
| KEGG | mmu05202:Transcriptional misregulation in cancer | 13 | 2.45283019 | 0.00247223 | ENSMUST00000018645, ENSMUST00000051446, ENSMUST00000101695, ENSMUST00000114136, ENSMUST00000168502, ENSMUST00000020377, ENSMUST00000115677, ENSMUST00000120105, ENSMUST00000139365, ENSMUST00000105343, ENSMUST00000115679, ENSMUST00000142080, ENSMUST00000177595, ENSMUST00000139965, ENSMUST00000204807, ENSMUST00000205391, ENSMUST00000105340 |
| KEGG | mmu01100:Metabolic pathways | 53 | 10 | 0.00291357 | ENSMUST00000156434, ENSMUST00000168419, ENSMUST00000163856, ENSMUST00000019986, ENSMUST00000033634, ENSMUST00000142920, ENSMUST00000177595, ENSMUST00000135642, ENSMUST00000133320, ENSMUST00000115242, ENSMUST00000142231, ENSMUST00000102540, ENSMUST00000099149, ENSMUST00000164763, ENSMUST00000165111, ENSMUST00000156390, ENSMUST00000080283, ENSMUST00000193235, ENSMUST00000148016, ENSMUST00000207851, ENSMUST00000105470, ENSMUST00000163476, ENSMUST00000127128, ENSMUST00000115137, ENSMUST00000202779, ENSMUST00000137266, ENSMUST00000082407, ENSMUST00000047111, ENSMUST00000139353, ENSMUST00000159784, ENSMUST00000163507, ENSMUST00000172281, ENSMUST00000097975, ENSMUST00000173409, ENSMUST00000030939, ENSMUST00000025546, ENSMUST00000143741, ENSMUST00000000430, ENSMUST00000152945, ENSMUST00000166036, ENSMUST00000127439, ENSMUST00000159644, ENSMUST00000106945, ENSMUST00000084433, ENSMUST00000026462, ENSMUST00000114171, ENSMUST00000105390, ENSMUST00000133474, ENSMUST00000180358, ENSMUST00000006053, ENSMUST00000153808, ENSMUST00000164412, ENSMUST00000074082, ENSMUST00000151505, ENSMUST00000113978, ENSMUST00000068004, ENSMUST00000084013, ENSMUST00000131186 |
| BIOCARTA | m_ptenpathway:PTEN dependent cell cycle arrest and apoptosis | 5 | 0.94339623 | 0.00809234 | ENSMUST00000054556, ENSMUST00000115409, ENSMUST00000166232, ENSMUST00000204807, ENSMUST00000191485 |
| KEGG | mmu04146:Peroxisome | 8 | 1.50943396 | 0.00894715 | ENSMUST00000166036, ENSMUST00000033634, ENSMUST00000155891, ENSMUST00000118234, ENSMUST00000135642, ENSMUST00000112531, ENSMUST00000164412, ENSMUST00000111168 |
| KEGG | mmu00310:Lysine degradation | 6 | 1.13207547 | 0.01540409 | ENSMUST00000152945, ENSMUST00000142080, ENSMUST00000151505, ENSMUST00000178486, ENSMUST00000108154, ENSMUST00000098853 |
| KEGG | mmu01200:Carbon metabolism | 9 | 1.69811321 | 0.01698051 | ENSMUST00000047111, ENSMUST00000177181, ENSMUST00000159784, ENSMUST00000151505, ENSMUST00000163507, ENSMUST00000142920, ENSMUST00000068004, ENSMUST00000173409, ENSMUST00000175712, ENSMUST00000111168 |
| KEGG | mmu04722:Neurotrophin signaling pathway | 9 | 1.69811321 | 0.02231057 | ENSMUST00000040308, ENSMUST00000095087, ENSMUST00000205340, ENSMUST00000003741, ENSMUST00000196397, ENSMUST00000102778, ENSMUST00000115409, ENSMUST00000205497, ENSMUST00000135636, ENSMUST00000005188, ENSMUST00000191485 |
| KEGG | mmu03320:PPAR signaling pathway | 7 | 1.32075472 | 0.02554751 | ENSMUST00000165232, ENSMUST00000033634, ENSMUST00000111319, ENSMUST00000113978, ENSMUST00000115409, ENSMUST00000135642, ENSMUST00000156390, ENSMUST00000164412 |
| BIOCARTA | m_rhoPathway:Rho cell motility signaling pathway | 5 | 0.94339623 | 0.02666366 | ENSMUST00000115631, ENSMUST00000108549, ENSMUST00000098683, ENSMUST00000103021, ENSMUST00000027579 |
| KEGG | mmu04141:Protein processing in endoplasmic reticulum | 10 | 1.88679245 | 0.04909959 | ENSMUST00000105470, ENSMUST00000197291, ENSMUST00000102778, ENSMUST00000166469, ENSMUST00000009236, ENSMUST00000128210, ENSMUST00000154774, ENSMUST00000202590, ENSMUST00000160653, ENSMUST00000197357 |

**Table 34.** Significant pathways for differentially expressed transcripts in ^28^Si vs. non-irradiated control at 2 months analyzed by DAVID (<https://david.ncifcrf.gov/>).

| **Database** | **Pathway** | **Count** | **%** | ***p*-value** | **Transcripts** |
| --- | --- | --- | --- | --- | --- |
| KEGG | mmu05010:Alzheimer's disease | 16 | 2.33236152 | 0.00291978 | ENSMUST00000082407, ENSMUST00000177974, ENSMUST00000082408, ENSMUST00000127300, ENSMUST00000021142, ENSMUST00000155377, ENSMUST00000193235, ENSMUST00000135602, ENSMUST00000031637, ENSMUST00000081180, ENSMUST00000140205, ENSMUST00000128787, ENSMUST00000032192, ENSMUST00000050580, ENSMUST00000204714, ENSMUST00000015712, ENSMUST00000208838, ENSMUST00000146103 |
| KEGG | mmu04070:Phosphatidylinositol signaling system | 11 | 1.60349854 | 0.00361487 | ENSMUST00000118390, ENSMUST00000092802, ENSMUST00000045469, ENSMUST00000184454, ENSMUST00000177654, ENSMUST00000110628, ENSMUST00000127300, ENSMUST00000042275, ENSMUST00000032192, ENSMUST00000115790, ENSMUST00000033700 |
| KEGG | mmu04120:Ubiquitin mediated proteolysis | 13 | 1.89504373 | 0.00760671 | ENSMUST00000161317, ENSMUST00000164034, ENSMUST00000131837, ENSMUST00000192762, ENSMUST00000164095, ENSMUST00000135310, ENSMUST00000126690, ENSMUST00000125514, ENSMUST00000054002, ENSMUST00000064900, ENSMUST00000145065, ENSMUST00000145567, ENSMUST00000105263, ENSMUST00000112622 |
| KEGG | mmu04144:Endocytosis | 19 | 2.7696793 | 0.01026865 | ENSMUST00000045469, ENSMUST00000179755, ENSMUST00000079777, ENSMUST00000108752, ENSMUST00000114311, ENSMUST00000037285, ENSMUST00000178202, ENSMUST00000148827, ENSMUST00000044234, ENSMUST00000172444, ENSMUST00000177731, ENSMUST00000135310, ENSMUST00000126690, ENSMUST00000098444, ENSMUST00000136590, ENSMUST00000091967, ENSMUST00000042185, ENSMUST00000120071, ENSMUST00000171509, ENSMUST00000131556, ENSMUST00000105263 |
| KEGG | mmu04932:Non-alcoholic fatty liver disease (NAFLD) | 13 | 1.89504373 | 0.01611833 | ENSMUST00000047111, ENSMUST00000177654, ENSMUST00000130031, ENSMUST00000155377, ENSMUST00000193235, ENSMUST00000135602, ENSMUST00000125314, ENSMUST00000031637, ENSMUST00000081180, ENSMUST00000128787, ENSMUST00000054002, ENSMUST00000050580, ENSMUST00000204714, ENSMUST00000208838, ENSMUST00000205391 |
| KEGG | mmu00562:Inositol phosphate metabolism | 8 | 1.16618076 | 0.01642783 | ENSMUST00000118390, ENSMUST00000092802, ENSMUST00000045469, ENSMUST00000184454, ENSMUST00000177654, ENSMUST00000042275, ENSMUST00000115790, ENSMUST00000033700 |
| KEGG | mmu01100:Metabolic pathways | 63 | 9.18367347 | 0.01835021 | ENSMUST00000156434, ENSMUST00000168419, ENSMUST00000174483, ENSMUST00000031637, ENSMUST00000092663, ENSMUST00000126074, ENSMUST00000103142, ENSMUST00000081180, ENSMUST00000128351, ENSMUST00000136776, ENSMUST00000128787, ENSMUST00000185612, ENSMUST00000205823, ENSMUST00000118390, ENSMUST00000184454, ENSMUST00000170534, ENSMUST00000115790, ENSMUST00000165111, ENSMUST00000193235, ENSMUST00000161737, ENSMUST00000033700, ENSMUST00000199489, ENSMUST00000163476, ENSMUST00000198126, ENSMUST00000050580, ENSMUST00000177447, ENSMUST00000202779, ENSMUST00000208838, ENSMUST00000159096, ENSMUST00000082407, ENSMUST00000082408, ENSMUST00000047111, ENSMUST00000149930, ENSMUST00000155272, ENSMUST00000159784, ENSMUST00000151089, ENSMUST00000145216, ENSMUST00000155377, ENSMUST00000030830, ENSMUST00000120541, ENSMUST00000148827, ENSMUST00000030939, ENSMUST00000143741, ENSMUST00000143108, ENSMUST00000034808, ENSMUST00000152945, ENSMUST00000201838, ENSMUST00000154045, ENSMUST00000112255, ENSMUST00000093350, ENSMUST00000146103, ENSMUST00000174214, ENSMUST00000172533, ENSMUST00000026462, ENSMUST00000045469, ENSMUST00000092802, ENSMUST00000069902, ENSMUST00000105390, ENSMUST00000135602, ENSMUST00000118917, ENSMUST00000031053, ENSMUST00000068004, ENSMUST00000204714, ENSMUST00000106501, ENSMUST00000189968, ENSMUST00000142957 |
| KEGG | mmu04022:cGMP-PKG signaling pathway | 13 | 1.89504373 | 0.0210429 | ENSMUST00000177974, ENSMUST00000193835, ENSMUST00000200389, ENSMUST00000127300, ENSMUST00000021142, ENSMUST00000174155, ENSMUST00000173456, ENSMUST00000125758, ENSMUST00000097274, ENSMUST00000112016, ENSMUST00000032192, ENSMUST00000152065, ENSMUST00000112007, ENSMUST00000107874 |
| KEGG | mmu05012:Parkinson's disease | 12 | 1.74927114 | 0.02606941 | ENSMUST00000082407, ENSMUST00000082408, ENSMUST00000193835, ENSMUST00000173456, ENSMUST00000155377, ENSMUST00000193235, ENSMUST00000135602, ENSMUST00000031637, ENSMUST00000081180, ENSMUST00000128787, ENSMUST00000050580, ENSMUST00000204714, ENSMUST00000146103, ENSMUST00000208838 |
| KEGG | mmu02010:ABC transporters | 6 | 0.87463557 | 0.0293033 | ENSMUST00000170725, ENSMUST00000047970, ENSMUST00000087527, ENSMUST00000115547, ENSMUST00000197662, ENSMUST00000117337 |
| KEGG | mmu01200:Carbon metabolism | 10 | 1.45772595 | 0.03191738 | ENSMUST00000047111, ENSMUST00000103142, ENSMUST00000177283, ENSMUST00000159784, ENSMUST00000198126, ENSMUST00000068004, ENSMUST00000030830, ENSMUST00000093350, ENSMUST00000174214, ENSMUST00000189968, ENSMUST00000177137 |
| KEGG | mmu01130:Biosynthesis of antibiotics | 15 | 2.18658892 | 0.03297454 | ENSMUST00000152945, ENSMUST00000047111, ENSMUST00000103142, ENSMUST00000155272, ENSMUST00000128351, ENSMUST00000136776, ENSMUST00000198126, ENSMUST00000145216, ENSMUST00000177447, ENSMUST00000165111, ENSMUST00000093350, ENSMUST00000174214, ENSMUST00000120541, ENSMUST00000143741, ENSMUST00000159096 |
| KEGG | mmu04062:Chemokine signaling pathway | 14 | 2.04081633 | 0.03551363 | ENSMUST00000177654, ENSMUST00000165335, ENSMUST00000179755, ENSMUST00000099080, ENSMUST00000079777, ENSMUST00000193835, ENSMUST00000031327, ENSMUST00000108752, ENSMUST00000106499, ENSMUST00000025705, ENSMUST00000101365, ENSMUST00000125314, ENSMUST00000136131, ENSMUST00000114225, ENSMUST00000152065, ENSMUST00000201245 |
| KEGG | mmu00190:Oxidative phosphorylation | 11 | 1.60349854 | 0.03839948 | ENSMUST00000082407, ENSMUST00000082408, ENSMUST00000155377, ENSMUST00000193235, ENSMUST00000135602, ENSMUST00000031637, ENSMUST00000092663, ENSMUST00000081180, ENSMUST00000128787, ENSMUST00000050580, ENSMUST00000204714, ENSMUST00000146103, ENSMUST00000208838 |
| KEGG | mmu04930:Type II diabetes mellitus | 6 | 0.87463557 | 0.04012106 | ENSMUST00000177654, ENSMUST00000047111, ENSMUST00000097274, ENSMUST00000054002, ENSMUST00000172070, ENSMUST00000125314 |
| BIOCARTA | m_biopeptidesPathway:Bioactive Peptide Induced Signaling Pathway | 6 | 0.87463557 | 0.041389 | ENSMUST00000107357, ENSMUST00000165335, ENSMUST00000106499, ENSMUST00000127300, ENSMUST00000025705, ENSMUST00000111335 |
| KEGG | mmu00310:Lysine degradation | 6 | 0.87463557 | 0.0463287 | ENSMUST00000152945, ENSMUST00000142080, ENSMUST00000114689, ENSMUST00000178486, ENSMUST00000093350, ENSMUST00000015841, ENSMUST00000107171 |

**Table 35.** Significant pathways for differentially expressed transcripts in ^28^Si vs. non-irradiated control at 4 months analyzed by DAVID (<https://david.ncifcrf.gov/>).

| **Database** | **Pathway** | **Count** | **%** | ***p*-value** | **Transcripts** |
| --- | --- | --- | --- | --- | --- |
| KEGG | mmu04917:Prolactin signaling pathway | 11 | 2.07156309 | 6.02E-05 | ENSMUST00000170690, ENSMUST00000107357, ENSMUST00000038859, ENSMUST00000106497, ENSMUST00000108242, ENSMUST00000138438, ENSMUST00000102778, ENSMUST00000111160, ENSMUST00000165664, ENSMUST00000054002, ENSMUST00000025705 |
| KEGG | mmu05221:Acute myeloid leukemia | 9 | 1.69491525 | 2.47E-04 | ENSMUST00000107357, ENSMUST00000136451, ENSMUST00000038859, ENSMUST00000106497, ENSMUST00000135310, ENSMUST00000108242, ENSMUST00000138438, ENSMUST00000111160, ENSMUST00000205391 |
| KEGG | mmu00562:Inositol phosphate metabolism | 9 | 1.69491525 | 0.00114307 | ENSMUST00000118390, ENSMUST00000019283, ENSMUST00000038859, ENSMUST00000035836, ENSMUST00000108242, ENSMUST00000115790, ENSMUST00000025391, ENSMUST00000137266, ENSMUST00000125476 |
| KEGG | mmu04920:Adipocytokine signaling pathway | 9 | 1.69491525 | 0.00137634 | ENSMUST00000105370, ENSMUST00000165232, ENSMUST00000110371, ENSMUST00000138438, ENSMUST00000102778, ENSMUST00000111160, ENSMUST00000054002, ENSMUST00000169095, ENSMUST00000174299, ENSMUST00000025705 |
| KEGG | mmu04070:Phosphatidylinositol signaling system | 10 | 1.88323917 | 0.00251909 | ENSMUST00000118390, ENSMUST00000038859, ENSMUST00000035836, ENSMUST00000108242, ENSMUST00000032192, ENSMUST00000115790, ENSMUST00000025391, ENSMUST00000137266, ENSMUST00000125476, ENSMUST00000089461 |
| KEGG | mmu00471:D-Glutamine and D-glutamate metabolism | 3 | 0.56497175 | 0.00266418 | ENSMUST00000159784, ENSMUST00000159440, ENSMUST00000135441, ENSMUST00000155587 |
| KEGG | mmu04932:Non-alcoholic fatty liver disease (NAFLD) | 13 | 2.44821092 | 0.00273542 | ENSMUST00000160684, ENSMUST00000155377, ENSMUST00000185466, ENSMUST00000193235, ENSMUST00000123497, ENSMUST00000038859, ENSMUST00000081180, ENSMUST00000108242, ENSMUST00000111160, ENSMUST00000102778, ENSMUST00000054002, ENSMUST00000208838, ENSMUST00000205391, ENSMUST00000108411 |
| KEGG | mmu05230:Central carbon metabolism in cancer | 8 | 1.50659134 | 0.00298555 | ENSMUST00000038859, ENSMUST00000087820, ENSMUST00000151358, ENSMUST00000108242, ENSMUST00000111160, ENSMUST00000159440, ENSMUST00000135441, ENSMUST00000155587, ENSMUST00000171509, ENSMUST00000052949 |
| KEGG | mmu04066:HIF-1 signaling pathway | 10 | 1.88323917 | 0.00354594 | ENSMUST00000071816, ENSMUST00000038859, ENSMUST00000165296, ENSMUST00000151358, ENSMUST00000108242, ENSMUST00000138438, ENSMUST00000111160, ENSMUST00000112645, ENSMUST00000179584, ENSMUST00000204807, ENSMUST00000052949 |
| KEGG | mmu05213:Endometrial cancer | 7 | 1.31826742 | 0.00450562 | ENSMUST00000136451, ENSMUST00000038859, ENSMUST00000106497, ENSMUST00000000312, ENSMUST00000108242, ENSMUST00000079362, ENSMUST00000111160 |
| KEGG | mmu04012:ErbB signaling pathway | 9 | 1.69491525 | 0.00456781 | ENSMUST00000071816, ENSMUST00000107357, ENSMUST00000136451, ENSMUST00000038859, ENSMUST00000106497, ENSMUST00000108242, ENSMUST00000102778, ENSMUST00000111160, ENSMUST00000204807 |
| KEGG | mmu04668:TNF signaling pathway | 10 | 1.88323917 | 0.00550592 | ENSMUST00000123497, ENSMUST00000038859, ENSMUST00000105527, ENSMUST00000080933, ENSMUST00000031327, ENSMUST00000108242, ENSMUST00000102778, ENSMUST00000111160, ENSMUST00000054002, ENSMUST00000201245, ENSMUST00000173984 |
| KEGG | mmu05220:Chronic myeloid leukemia | 8 | 1.50659134 | 0.00577954 | ENSMUST00000107357, ENSMUST00000136451, ENSMUST00000038859, ENSMUST00000106497, ENSMUST00000108242, ENSMUST00000111160, ENSMUST00000204807, ENSMUST00000105263 |
| KEGG | mmu04931:Insulin resistance | 10 | 1.88323917 | 0.0058434 | ENSMUST00000165232, ENSMUST00000038859, ENSMUST00000168974, ENSMUST00000108242, ENSMUST00000034267, ENSMUST00000138438, ENSMUST00000102778, ENSMUST00000111160, ENSMUST00000054002, ENSMUST00000169095, ENSMUST00000167197 |
| KEGG | mmu01100:Metabolic pathways | 54 | 10.1694915 | 0.00683251 | ENSMUST00000035488, ENSMUST00000185466, ENSMUST00000136162, ENSMUST00000135441, ENSMUST00000114952, ENSMUST00000092663, ENSMUST00000081180, ENSMUST00000128351, ENSMUST00000110371, ENSMUST00000098862, ENSMUST00000089461, ENSMUST00000082214, ENSMUST00000118390, ENSMUST00000006838, ENSMUST00000025968, ENSMUST00000115790, ENSMUST00000156390, ENSMUST00000165111, ENSMUST00000193235, ENSMUST00000155587, ENSMUST00000161737, ENSMUST00000165234, ENSMUST00000126561, ENSMUST00000199489, ENSMUST00000202779, ENSMUST00000208838, ENSMUST00000137266, ENSMUST00000125476, ENSMUST00000159096, ENSMUST00000159784, ENSMUST00000145216, ENSMUST00000074127, ENSMUST00000155377, ENSMUST00000208043, ENSMUST00000148827, ENSMUST00000143741, ENSMUST00000030939, ENSMUST00000143108, ENSMUST00000034808, ENSMUST00000019283, ENSMUST00000093350, ENSMUST00000159440, ENSMUST00000029464, ENSMUST00000052949, ENSMUST00000084433, ENSMUST00000161090, ENSMUST00000031766, ENSMUST00000174595, ENSMUST00000005685, ENSMUST00000005477, ENSMUST00000151200, ENSMUST00000025391, ENSMUST00000159572, ENSMUST00000106501, ENSMUST00000023684, ENSMUST00000142957 |
| KEGG | mmu04068:FoxO signaling pathway | 11 | 2.07156309 | 0.00713999 | ENSMUST00000105370, ENSMUST00000136451, ENSMUST00000038859, ENSMUST00000106497, ENSMUST00000108242, ENSMUST00000138438, ENSMUST00000102778, ENSMUST00000111160, ENSMUST00000204807, ENSMUST00000147730, ENSMUST00000105263 |
| KEGG | mmu04910:Insulin signaling pathway | 11 | 2.07156309 | 0.00960973 | ENSMUST00000170690, ENSMUST00000113869, ENSMUST00000136451, ENSMUST00000038859, ENSMUST00000106497, ENSMUST00000035836, ENSMUST00000108242, ENSMUST00000102778, ENSMUST00000111160, ENSMUST00000054002, ENSMUST00000052949, ENSMUST00000113870 |
| BIOCARTA | m_il22bpPathway:IL22 Soluble Receptor Signaling Pathway | 4 | 0.75329567 | 0.0105103 | ENSMUST00000107357, ENSMUST00000138438, ENSMUST00000054002, ENSMUST00000025705 |
| KEGG | mmu04142:Lysosome | 10 | 1.88323917 | 0.01126013 | ENSMUST00000126561, ENSMUST00000092663, ENSMUST00000156742, ENSMUST00000163739, ENSMUST00000131108, ENSMUST00000132718, ENSMUST00000117645, ENSMUST00000098862, ENSMUST00000112610, ENSMUST00000177779 |
| KEGG | mmu04630:Jak-STAT signaling pathway | 11 | 2.07156309 | 0.0121272 | ENSMUST00000170690, ENSMUST00000107357, ENSMUST00000038859, ENSMUST00000106497, ENSMUST00000108242, ENSMUST00000138438, ENSMUST00000111160, ENSMUST00000165664, ENSMUST00000164529, ENSMUST00000054002, ENSMUST00000025705 |
| KEGG | mmu05210:Colorectal cancer | 7 | 1.31826742 | 0.01231322 | ENSMUST00000136451, ENSMUST00000038859, ENSMUST00000022220, ENSMUST00000108242, ENSMUST00000079362, ENSMUST00000102778, ENSMUST00000111160 |
| KEGG | mmu05214:Glioma | 7 | 1.31826742 | 0.01323369 | ENSMUST00000071816, ENSMUST00000136451, ENSMUST00000038859, ENSMUST00000106497, ENSMUST00000108242, ENSMUST00000111160, ENSMUST00000105263 |
| KEGG | mmu04914:Progesterone-mediated oocyte maturation | 8 | 1.50659134 | 0.01570398 | ENSMUST00000136451, ENSMUST00000038859, ENSMUST00000166713, ENSMUST00000163966, ENSMUST00000168974, ENSMUST00000108242, ENSMUST00000102778, ENSMUST00000166469, ENSMUST00000111160, ENSMUST00000169035 |
| KEGG | mmu05215:Prostate cancer | 8 | 1.50659134 | 0.01663789 | ENSMUST00000136451, ENSMUST00000038859, ENSMUST00000163966, ENSMUST00000106497, ENSMUST00000108242, ENSMUST00000166469, ENSMUST00000111160, ENSMUST00000204807, ENSMUST00000105263 |
| KEGG | mmu04930:Type II diabetes mellitus | 6 | 1.1299435 | 0.01681077 | ENSMUST00000170690, ENSMUST00000038859, ENSMUST00000108242, ENSMUST00000102778, ENSMUST00000054002, ENSMUST00000052949 |
| KEGG | mmu00220:Arginine biosynthesis | 4 | 0.75329567 | 0.01837028 | ENSMUST00000159784, ENSMUST00000159440, ENSMUST00000135441, ENSMUST00000155587, ENSMUST00000159096 |
| KEGG | mmu04550:Signaling pathways regulating pluripotency of stem cells | 10 | 1.88323917 | 0.02345674 | ENSMUST00000038859, ENSMUST00000087820, ENSMUST00000106497, ENSMUST00000108242, ENSMUST00000079362, ENSMUST00000138438, ENSMUST00000111160, ENSMUST00000164529, ENSMUST00000015841, ENSMUST00000171509, ENSMUST00000025705 |
| KEGG | mmu00250:Alanine, aspartate and glutamate metabolism | 5 | 0.94161959 | 0.0244414 | ENSMUST00000159784, ENSMUST00000031766, ENSMUST00000159440, ENSMUST00000135441, ENSMUST00000155587, ENSMUST00000159096 |
| KEGG | mmu05223:Non-small cell lung cancer | 6 | 1.1299435 | 0.02623189 | ENSMUST00000136451, ENSMUST00000038859, ENSMUST00000106497, ENSMUST00000108242, ENSMUST00000111160, ENSMUST00000174299 |
| KEGG | mmu05202:Transcriptional misregulation in cancer | 11 | 2.07156309 | 0.02736986 | ENSMUST00000051446, ENSMUST00000185355, ENSMUST00000135310, ENSMUST00000114689, ENSMUST00000043396, ENSMUST00000061829, ENSMUST00000204807, ENSMUST00000174299, ENSMUST00000205391, ENSMUST00000105263, ENSMUST00000120105 |
| BIOCARTA | m_tpoPathway:TPO Signaling Pathway | 5 | 0.94161959 | 0.02820091 | ENSMUST00000107357, ENSMUST00000106497, ENSMUST00000108242, ENSMUST00000138438, ENSMUST00000025705 |
| KEGG | mmu01130:Biosynthesis of antibiotics | 13 | 2.44821092 | 0.02828194 | ENSMUST00000019283, ENSMUST00000161090, ENSMUST00000128351, ENSMUST00000145216, ENSMUST00000074127, ENSMUST00000151200, ENSMUST00000165111, ENSMUST00000093350, ENSMUST00000023684, ENSMUST00000029464, ENSMUST00000052949, ENSMUST00000159096, ENSMUST00000143741 |
| KEGG | mmu04978:Mineral absorption | 5 | 0.94161959 | 0.02905876 | ENSMUST00000028843, ENSMUST00000034214, ENSMUST00000165296, ENSMUST00000034215, ENSMUST00000112645, ENSMUST00000164219 |
| KEGG | mmu05200:Pathways in cancer | 20 | 3.76647834 | 0.02936838 | ENSMUST00000136451, ENSMUST00000107357, ENSMUST00000160684, ENSMUST00000000312, ENSMUST00000106497, ENSMUST00000079362, ENSMUST00000022220, ENSMUST00000166469, ENSMUST00000174299, ENSMUST00000120375, ENSMUST00000038859, ENSMUST00000087820, ENSMUST00000163966, ENSMUST00000135310, ENSMUST00000108242, ENSMUST00000138438, ENSMUST00000102778, ENSMUST00000111160, ENSMUST00000204807, ENSMUST00000205391, ENSMUST00000171509, ENSMUST00000105263 |
| KEGG | mmu04141:Protein processing in endoplasmic reticulum | 11 | 2.07156309 | 0.03048941 | ENSMUST00000197291, ENSMUST00000120461, ENSMUST00000163966, ENSMUST00000188346, ENSMUST00000102778, ENSMUST00000166469, ENSMUST00000124613, ENSMUST00000009236, ENSMUST00000128210, ENSMUST00000146979, ENSMUST00000173984, ENSMUST00000108796 |
| KEGG | mmu04150:mTOR signaling pathway | 6 | 1.1299435 | 0.03199445 | ENSMUST00000113869, ENSMUST00000105370, ENSMUST00000038859, ENSMUST00000168974, ENSMUST00000108242, ENSMUST00000111160, ENSMUST00000113870 |
| KEGG | mmu05231:Choline metabolism in cancer | 8 | 1.50659134 | 0.03255957 | ENSMUST00000113869, ENSMUST00000038859, ENSMUST00000106497, ENSMUST00000108242, ENSMUST00000102778, ENSMUST00000111160, ENSMUST00000148827, ENSMUST00000113870, ENSMUST00000143108 |
| KEGG | mmu04062:Chemokine signaling pathway | 12 | 2.25988701 | 0.03471766 | ENSMUST00000162602, ENSMUST00000079777, ENSMUST00000106497, ENSMUST00000152156, ENSMUST00000031327, ENSMUST00000023840, ENSMUST00000025705, ENSMUST00000160766, ENSMUST00000038859, ENSMUST00000021970, ENSMUST00000138438, ENSMUST00000108242, ENSMUST00000111160, ENSMUST00000201245, ENSMUST00000108411 |
| KEGG | mmu04380:Osteoclast differentiation | 9 | 1.69491525 | 0.03638332 | ENSMUST00000038859, ENSMUST00000106497, ENSMUST00000080933, ENSMUST00000108242, ENSMUST00000102778, ENSMUST00000111160, ENSMUST00000054002, ENSMUST00000027241, ENSMUST00000103202 |
| KEGG | mmu04973:Carbohydrate digestion and absorption | 5 | 0.94161959 | 0.0368931 | ENSMUST00000038859, ENSMUST00000108242, ENSMUST00000111160, ENSMUST00000202779, ENSMUST00000052949 |
| BIOCARTA | m_ghPathway:Growth Hormone Signaling Pathway | 5 | 0.94161959 | 0.03991311 | ENSMUST00000107357, ENSMUST00000106497, ENSMUST00000168974, ENSMUST00000108242, ENSMUST00000025705 |
| KEGG | mmu05212:Pancreatic cancer | 6 | 1.1299435 | 0.04574069 | ENSMUST00000136451, ENSMUST00000038859, ENSMUST00000108242, ENSMUST00000138438, ENSMUST00000102778, ENSMUST00000111160 |
| KEGG | mmu05203:Viral carcinogenesis | 13 | 2.44821092 | 0.04654494 | ENSMUST00000107357, ENSMUST00000152156, ENSMUST00000106497, ENSMUST00000107644, ENSMUST00000145675, ENSMUST00000107150, ENSMUST00000038859, ENSMUST00000144909, ENSMUST00000138438, ENSMUST00000108242, ENSMUST00000204807, ENSMUST00000173984, ENSMUST00000105263 |

**Table 36.** Significant pathways for differentially expressed transcripts in ^28^Si vs. non-irradiated control at 9 months analyzed by DAVID (<https://david.ncifcrf.gov/>).

| **Database** | **Pathway** | **Count** | **%** | ***p*-value** | **Transcripts** |
| --- | --- | --- | --- | --- | --- |
| KEGG | mmu04931:Insulin resistance | 12 | 2.44897959 | 1.78E-04 | ENSMUST00000165232, ENSMUST00000038859, ENSMUST00000177654, ENSMUST00000196469, ENSMUST00000003741, ENSMUST00000034267, ENSMUST00000085469, ENSMUST00000102778, ENSMUST00000128691, ENSMUST00000107911, ENSMUST00000063307, ENSMUST00000051186, ENSMUST00000119299 |
| KEGG | mmu05169:Epstein-Barr virus infection | 12 | 2.44897959 | 0.0010994 | ENSMUST00000177654, ENSMUST00000106306, ENSMUST00000025181, ENSMUST00000038859, ENSMUST00000105527, ENSMUST00000196469, ENSMUST00000085469, ENSMUST00000142055, ENSMUST00000102778, ENSMUST00000044078, ENSMUST00000161976, ENSMUST00000173280, ENSMUST00000086083 |
| KEGG | mmu04920:Adipocytokine signaling pathway | 8 | 1.63265306 | 0.00317893 | ENSMUST00000165232, ENSMUST00000187699, ENSMUST00000166775, ENSMUST00000112903, ENSMUST00000196469, ENSMUST00000110371, ENSMUST00000102778, ENSMUST00000051186 |
| KEGG | mmu04141:Protein processing in endoplasmic reticulum | 12 | 2.44897959 | 0.00575216 | ENSMUST00000197291, ENSMUST00000110874, ENSMUST00000120461, ENSMUST00000102778, ENSMUST00000166469, ENSMUST00000055223, ENSMUST00000128210, ENSMUST00000191070, ENSMUST00000203353, ENSMUST00000146979, ENSMUST00000205279, ENSMUST00000191273 |
| KEGG | mmu05222:Small cell lung cancer | 8 | 1.63265306 | 0.00745052 | ENSMUST00000038859, ENSMUST00000177654, ENSMUST00000166775, ENSMUST00000111740, ENSMUST00000196469, ENSMUST00000085469, ENSMUST00000112930, ENSMUST00000162618, ENSMUST00000161976 |
| BIOCARTA | m_gcrPathway:Corticosteroids and cardioprotection | 5 | 1.02040816 | 0.00749019 | ENSMUST00000153524, ENSMUST00000025300, ENSMUST00000165335, ENSMUST00000196469, ENSMUST00000085469, ENSMUST00000176637, ENSMUST00000105616 |
| KEGG | mmu04914:Progesterone-mediated oocyte maturation | 8 | 1.63265306 | 0.00898317 | ENSMUST00000038859, ENSMUST00000177654, ENSMUST00000084129, ENSMUST00000003741, ENSMUST00000085469, ENSMUST00000102778, ENSMUST00000166469, ENSMUST00000170223, ENSMUST00000197074 |
| KEGG | mmu04932:Non-alcoholic fatty liver disease (NAFLD) | 11 | 2.24489796 | 0.01000398 | ENSMUST00000038859, ENSMUST00000177654, ENSMUST00000187699, ENSMUST00000166775, ENSMUST00000196469, ENSMUST00000085469, ENSMUST00000102778, ENSMUST00000128691, ENSMUST00000155377, ENSMUST00000193235, ENSMUST00000051186, ENSMUST00000134479 |
| KEGG | mmu04015:Rap1 signaling pathway | 13 | 2.65306122 | 0.01311959 | ENSMUST00000153524, ENSMUST00000177654, ENSMUST00000028525, ENSMUST00000079777, ENSMUST00000000312, ENSMUST00000118768, ENSMUST00000106306, ENSMUST00000122054, ENSMUST00000169854, ENSMUST00000111677, ENSMUST00000170223, ENSMUST00000162536, ENSMUST00000038859, ENSMUST00000085469, ENSMUST00000171509 |
| KEGG | mmu05202:Transcriptional misregulation in cancer | 11 | 2.24489796 | 0.01386241 | ENSMUST00000066854, ENSMUST00000168502, ENSMUST00000020377, ENSMUST00000154666, ENSMUST00000078137, ENSMUST00000115677, ENSMUST00000120105, ENSMUST00000105344, ENSMUST00000166775, ENSMUST00000105343, ENSMUST00000196469, ENSMUST00000142080, ENSMUST00000031256, ENSMUST00000161976 |
| KEGG | mmu04621:NOD-like receptor signaling pathway | 6 | 1.2244898 | 0.01848726 | ENSMUST00000105527, ENSMUST00000196469, ENSMUST00000169083, ENSMUST00000102778, ENSMUST00000166469, ENSMUST00000180021 |
| KEGG | mmu05152:Tuberculosis | 11 | 2.24489796 | 0.02087162 | ENSMUST00000153524, ENSMUST00000131405, ENSMUST00000114171, ENSMUST00000196469, ENSMUST00000167610, ENSMUST00000102778, ENSMUST00000145791, ENSMUST00000162618, ENSMUST00000078800, ENSMUST00000180021, ENSMUST00000169017 |
| KEGG | mmu04919:Thyroid hormone signaling pathway | 8 | 1.63265306 | 0.03455138 | ENSMUST00000038859, ENSMUST00000177654, ENSMUST00000166775, ENSMUST00000168502, ENSMUST00000111740, ENSMUST00000085469, ENSMUST00000082426, ENSMUST00000087956, ENSMUST00000161976 |
| KEGG | mmu05204:Chemical carcinogenesis | 7 | 1.42857143 | 0.03799896 | ENSMUST00000106681, ENSMUST00000035488, ENSMUST00000005477, ENSMUST00000072438, ENSMUST00000106670, ENSMUST00000107161, ENSMUST00000126593, ENSMUST00000082214 |
| KEGG | mmu05200:Pathways in cancer | 18 | 3.67346939 | 0.03860082 | ENSMUST00000177654, ENSMUST00000165335, ENSMUST00000000312, ENSMUST00000111740, ENSMUST00000122054, ENSMUST00000166469, ENSMUST00000112930, ENSMUST00000107161, ENSMUST00000108270, ENSMUST00000170223, ENSMUST00000041717, ENSMUST00000105616, ENSMUST00000038859, ENSMUST00000166775, ENSMUST00000196469, ENSMUST00000085469, ENSMUST00000102778, ENSMUST00000176637, ENSMUST00000171509, ENSMUST00000161976, ENSMUST00000044620 |
| KEGG | mmu04670:Leukocyte transendothelial migration | 8 | 1.63265306 | 0.04050485 | ENSMUST00000038859, ENSMUST00000177654, ENSMUST00000028525, ENSMUST00000144909, ENSMUST00000152156, ENSMUST00000085469, ENSMUST00000106306, ENSMUST00000169854, ENSMUST00000111677 |
| KEGG | mmu04145:Phagosome | 10 | 2.04081633 | 0.04209775 | ENSMUST00000165232, ENSMUST00000131405, ENSMUST00000114171, ENSMUST00000027639, ENSMUST00000111740, ENSMUST00000145791, ENSMUST00000025181, ENSMUST00000146979, ENSMUST00000169017, ENSMUST00000173280 |
| KEGG | mmu05216:Thyroid cancer | 4 | 0.81632653 | 0.04251678 | ENSMUST00000166775, ENSMUST00000000312, ENSMUST00000041717, ENSMUST00000161976 |
| KEGG | mmu04976:Bile secretion | 6 | 1.2244898 | 0.04259054 | ENSMUST00000166775, ENSMUST00000033023, ENSMUST00000113218, ENSMUST00000170223, ENSMUST00000032364, ENSMUST00000102709 |
| KEGG | mmu05161:Hepatitis B | 9 | 1.83673469 | 0.04411242 | ENSMUST00000038859, ENSMUST00000177654, ENSMUST00000114171, ENSMUST00000196469, ENSMUST00000142055, ENSMUST00000085469, ENSMUST00000102778, ENSMUST00000162618, ENSMUST00000100009, ENSMUST00000161976 |
| KEGG | mmu05150:Staphylococcus aureus infection | 5 | 1.02040816 | 0.04550913 | ENSMUST00000136647, ENSMUST00000152417, ENSMUST00000106306, ENSMUST00000123238, ENSMUST00000169017 |
| KEGG | mmu04142:Lysosome | 8 | 1.63265306 | 0.04709184 | ENSMUST00000106508, ENSMUST00000128225, ENSMUST00000163739, ENSMUST00000114171, ENSMUST00000170285, ENSMUST00000020928, ENSMUST00000145791, ENSMUST00000132718, ENSMUST00000156173 |
| KEGG | mmu04722:Neurotrophin signaling pathway | 8 | 1.63265306 | 0.04709184 | ENSMUST00000153524, ENSMUST00000038859, ENSMUST00000177654, ENSMUST00000205340, ENSMUST00000020448, ENSMUST00000196469, ENSMUST00000003741, ENSMUST00000085469, ENSMUST00000102778 |
| KEGG | mmu04070:Phosphatidylinositol signaling system | 7 | 1.42857143 | 0.04721801 | ENSMUST00000153524, ENSMUST00000038859, ENSMUST00000177654, ENSMUST00000131405, ENSMUST00000085469, ENSMUST00000110627, ENSMUST00000089461, ENSMUST00000033700 |

**Table 37.** Significant pathways for differentially expressed transcripts in ^28^Si vs. non-irradiated control at 12 months analyzed by DAVID (<https://david.ncifcrf.gov/>).

**
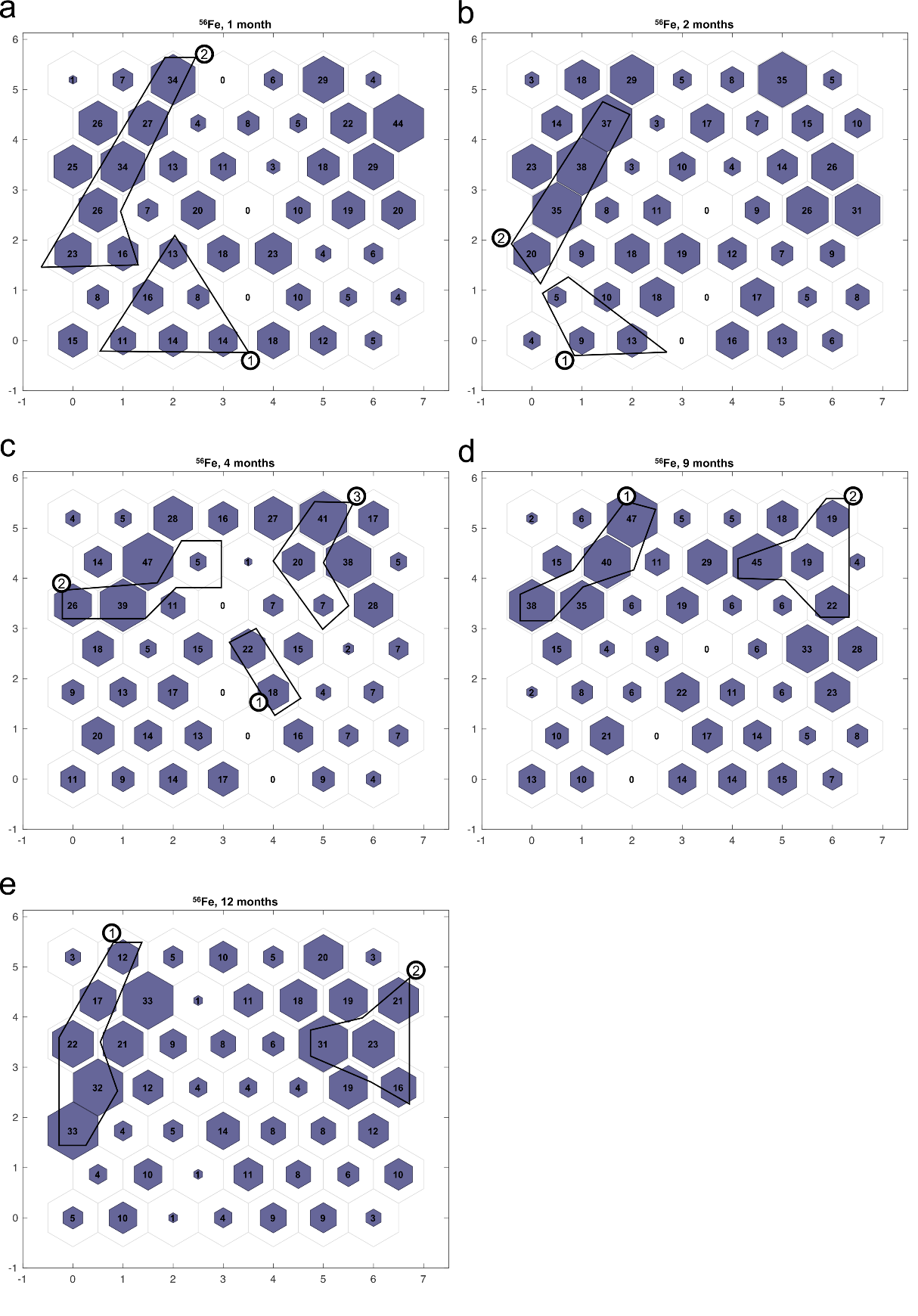
**

**Figure 1. ^56^Fe Analysis of self-organizing maps for each time point. (a,b,c,d,e)** Kohonen Self-Organizing Map (SOM) was applied to the differentially expressed (DE) transcripts obtained from the RNA-Seq data to identify coherent patterns of transcript expression at each time point, as well as patterns within the unmapped transcripts. The mapping clusters transcripts in each unit according to log_2_(fold change) expression values for the transcripts in that unit. SOM clustering analysis demonstrates the distances between correlated transcript groups. The small blue hexagons are modules comprising transcripts with similar log_2_(fold change) expression pattern. The numbers inside hexagons correspond to the number of transcripts in each module.

**
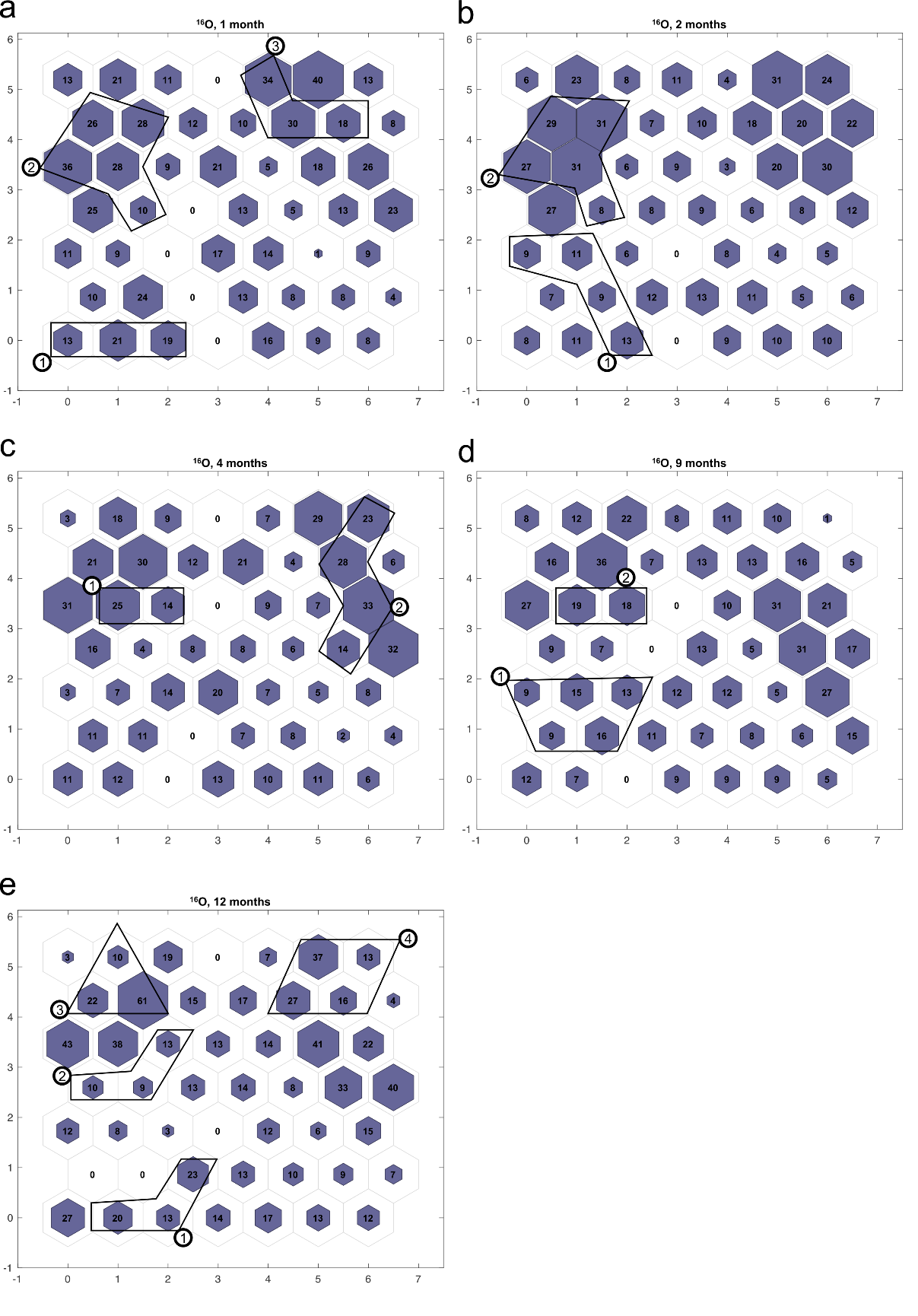
**

**Figure 2:** **^16^O Analysis of self-organizing maps for each time point**. **(a,b,c,d,e)** Kohonen Self-Organizing Map (SOM) was applied to the differentially expressed (DE) transcripts obtained from the RNA-Seq data to identify coherent patterns of transcript expression at each time point, as well as patterns within the unmapped transcripts. The mapping clusters transcripts in each unit according to log_2_(fold change) expression values for the transcripts in that unit. SOM clustering analysis demonstrates the distances between correlated transcript groups. The small blue hexagons are modules comprising transcripts with similar log_2_(fold change) expression pattern. The numbers inside hexagons correspond to the number of transcripts in each module.

**
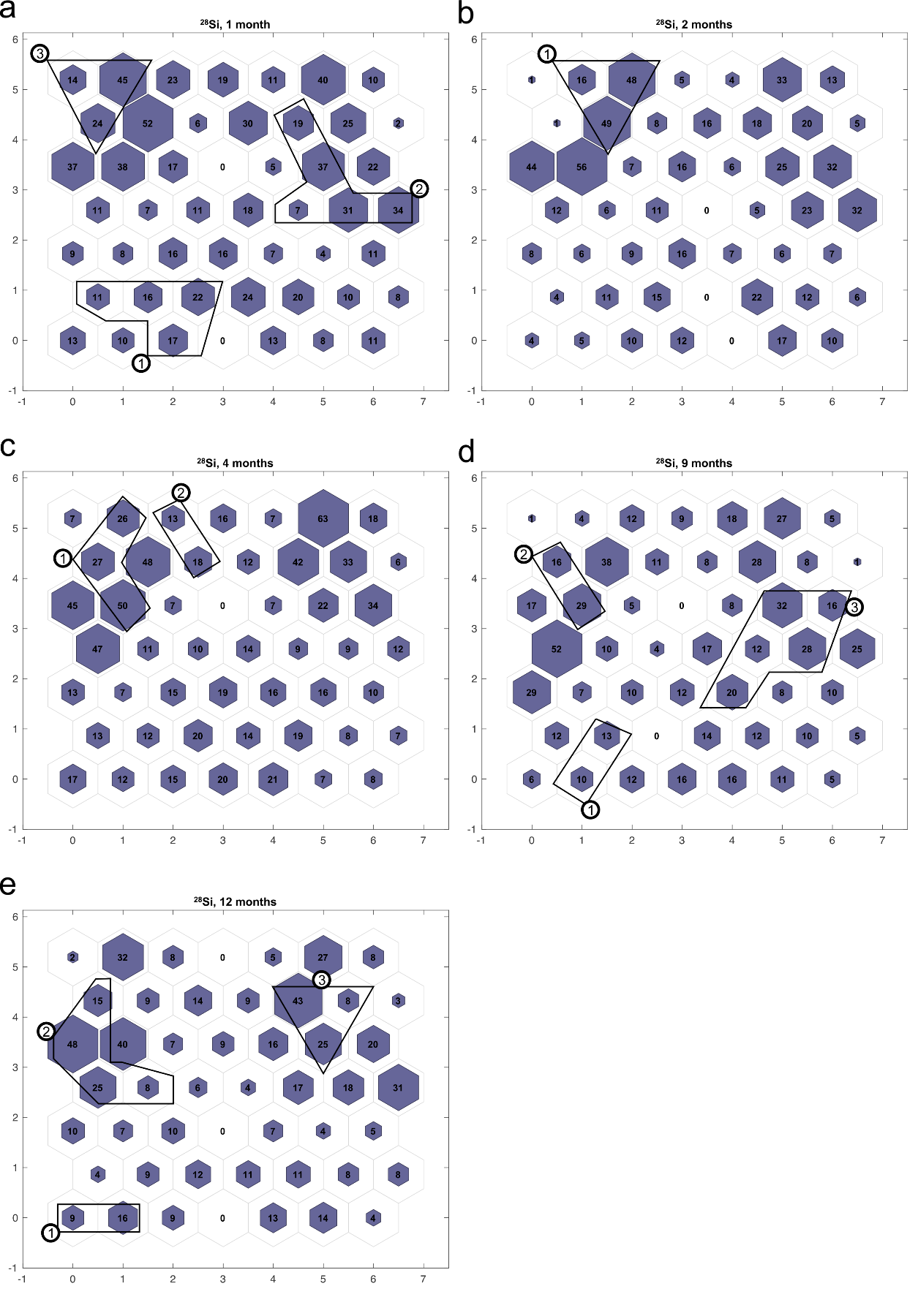
**

**Figure 3:** **^28^Si Analysis of self-organizing maps for each time point**. **(a,b,c,d,e)** Kohonen Self-Organizing Map (SOM) was applied to the differentially expressed (DE) transcripts obtained from the RNA-Seq data to identify coherent patterns of transcript expression at each time point, as well as patterns within the unmapped transcripts. The mapping clusters transcripts in each unit according to log_2_(fold change) expression values for the transcripts in that unit. SOM clustering analysis demonstrates the distances between correlated transcript groups. The small blue hexagons are modules comprising transcripts with similar log_2_(fold change) expression pattern. The numbers inside hexagons correspond to the number of transcripts in each module.
